# Combining Ligand- and Structure-Based Approaches to Develop Haloacetamide-Derived Di(hetero)arylmethanes as Potential Anticancer Agents

**DOI:** 10.3390/ijms27219559

**Published:** 2026-10-26

**Authors:** Karina O. Shibaeva, Andrey V. Smolobochkin, Elena A. Chugunova, Hoang Bao Tran Nguyen, Adel M. Shakirov, Elmira M. Gibadullina, Anna P. Lyubina, Anastasiia S. Sapunova, Alexandra D. Voloshina, Yulia R. Aleksandrova, Margarita E. Neganova, Almir S. Gazizov, Alexander R. Burilov, Michail A. Pudovik, Oleg G. Sinyashin

**Affiliations:** 1 Arbuzov Institute of Organic and Physical Chemistry, FRC Kazan Scientific Center, Russian Academy of Sciences, Arbuzova Str. 8, Kazan 420088, Russia; 2 Kazan National Research Technological University, K. Marksa Str. 68, Kazan 420015, Russia; 3 A.N. Nesmeyanov Institute of Organoelement Compounds of Russian Academy of Sciences, Vavilova Str., 28, Moscow 119334, Russia

**Keywords:** covalent inhibitors, di(hetero)arylmethanes, haloacetamides, GAPDH, GSTO1, anticancer activity, computer-aided drug design

## Abstract

The convergence of ligand-based and structure-based computational strategies provides a robust foundation for hit optimization in anticancer drug discovery. We integrated ligand-based SAR with structure-based covalent docking to guide the optimization of haloacetamide-derived di(hetero)arylmethanes. SAR revealed that the 2-haloacetamide warhead is essential, while the di(hetero)aryl scaffold modulates activity, leading to submicromolar bis(pyrazolyl)methane hits. Rather than seeking to establish a definitive molecular target, we used GAPDH and GSTO1 as experimentally motivated target hypotheses to derive structural constraints for covalent scaffold design. Covalent docking was used to prioritize these hypotheses and guide the design of phosphorylated diarylmethane derivatives, leading to the identification of highly cytotoxic bromoacetamide compound, comparable to doxorubicin. In PANC-1 cells, this lead decreased LDH activity and increased PDH activity, suggesting a shift in cellular metabolism from glycolysis toward oxidative phosphorylation. The results demonstrate the utility of target-hypothesis-guided design under conditions in which molecular target identity remains unresolved.

## 1. Introduction

Structure- and ligand-based drug design are the two general types of computer-aided drug design approaches in existence [1,2]. A structure-based approach implies prior knowledge of the drug target and employs molecular docking to predict novel candidate compounds [3,4]. Though well-known and industry-recognized, this approach is not without obstacles. The most evident limitation is the need for an experimentally elucidated or predicted three-dimensional structure of the target, which is not always available. Several other challenges exist, ranging from the adequacy of the 3D structures of the target and the ligand to the accuracy of the scoring function [3,5,6]. On the other hand, the ligand-based approach is the most frequent choice when the target information is not available [7,8]. Although an attractive alternative to a structure-based approach, it requires a library of structurally diverse compounds sharing the same scaffold, which may be difficult to synthesize. In addition to this, the entire spectrum of activity should be covered to obtain a reliable model, which also often causes complications. While the integration of ligand- and structure-based approaches may be a promising way to guide the search for novel hits, reports of such combined strategies are still rare [9,10].

**Figure 1:**
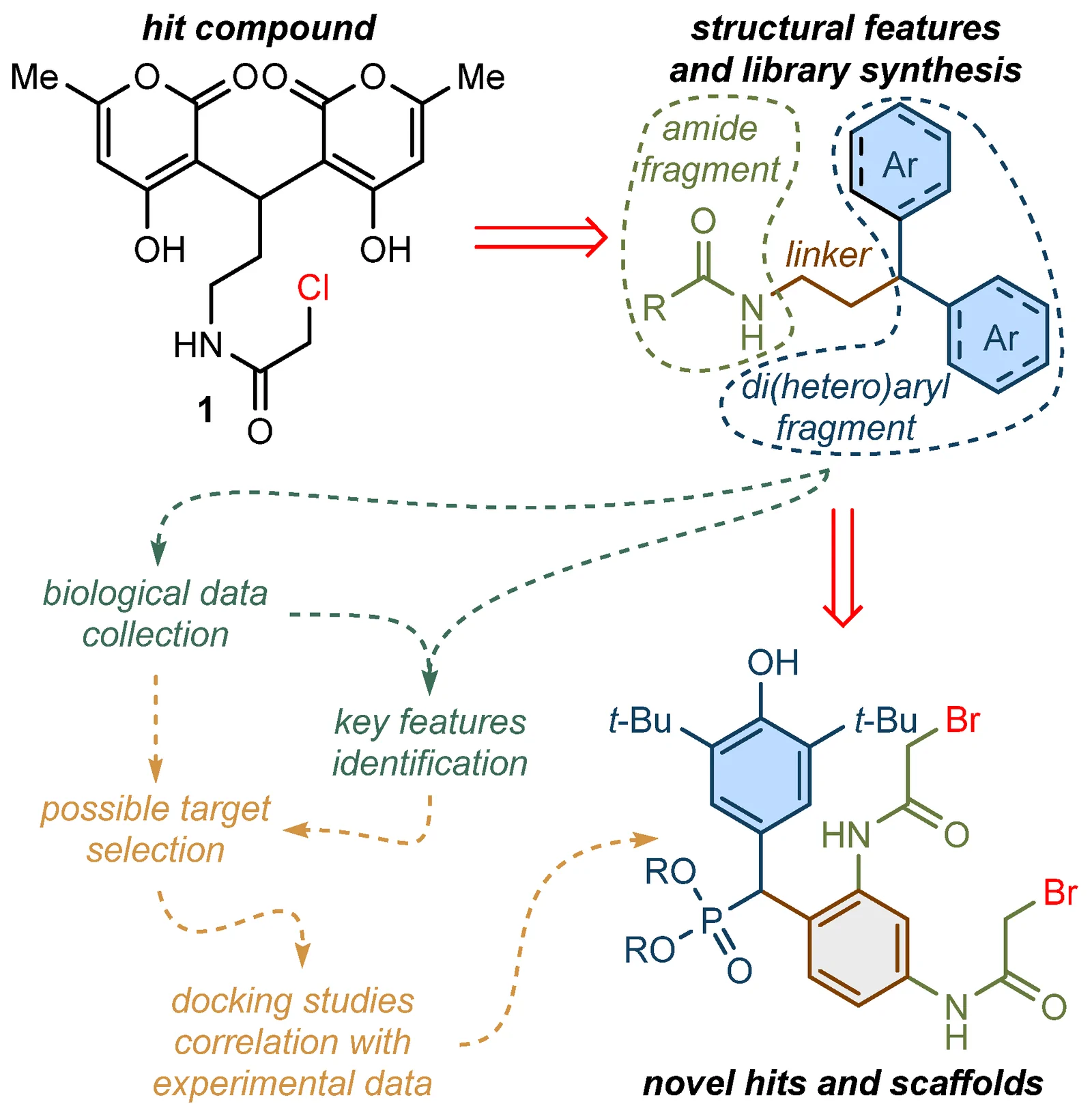
General workflow for ligand-based SAR analysis, formulation of candidate target hypotheses, and target-hypothesis-guided design of novel hit compounds.

Recently, we have reported the synthesis and evaluation of in vitro cytotoxicity against cancer and normal human cell lines for a series of dibenzoxanthene and di(hetero)arylmethane derivatives [11]. Among these, compound **1** (Figure 1) combined excellent efficacy with an IC_50_ of 1.7 µM in the HuTu-80 cancer cell line and a high selectivity index of 73, significantly outperforming the reference drug Doxorubicin. The obtained results suggest that the mechanism of cytotoxic action for the hit compound **1** is associated with the induction of apoptosis via the intrinsic mitochondrial pathway. It was also shown to induce a metabolic crisis through inhibition of glycolysis in human ovarian teratocarcinoma PA-1 cells. These encouraging preliminary results prompted us to further explore the chemical space of di(hetero)arylmethanes to reveal key structural features responsible for their activity and to identify more potent hit compounds.

Figure 1 shows our library design alongside the general workflow of the present study. The amide fragment and the di(hetero)aryl moiety were chosen as the main structural features of hit compound **1**, connected by a (poly)methylene spacer. Systematic modifications of these structural elements, followed by cytotoxicity evaluation, allowed us to clarify the contribution of each fragment to the overall activity, constituting the “ligand-based” part of the work. The data obtained were subsequently used in the “structure-based” part of the study to formulate plausible molecular target hypotheses through careful examination of the literature and to evaluate these hypotheses using covalent docking. The objective was not to establish a definitive molecular target for the haloacetamide derivatives, but rather to determine whether experimentally motivated target hypotheses could be incorporated into the design process and used as structural constraints to guide the exploration of previously untested chemical space. Glyceraldehyde 3-phosphate dehydrogenase (GAPDH) and glutathione S-transferase omega-1 (GSTO1) were considered as candidate targets based on their biological relevance, reported susceptibility to covalent modification, and compatibility with the electrophilic haloacetamide warhead. Covalent docking was therefore used to evaluate these hypotheses at the structural level and to derive design principles for novel diarylmethane scaffolds. The resulting predictions were used to design and select plausible novel hits, which were subsequently synthesized and evaluated experimentally, providing a prospective test of whether the target hypotheses could successfully guide scaffold design.

## 2. Results and Discussion

### 2.1. Library Synthesis

**Scheme 1:**
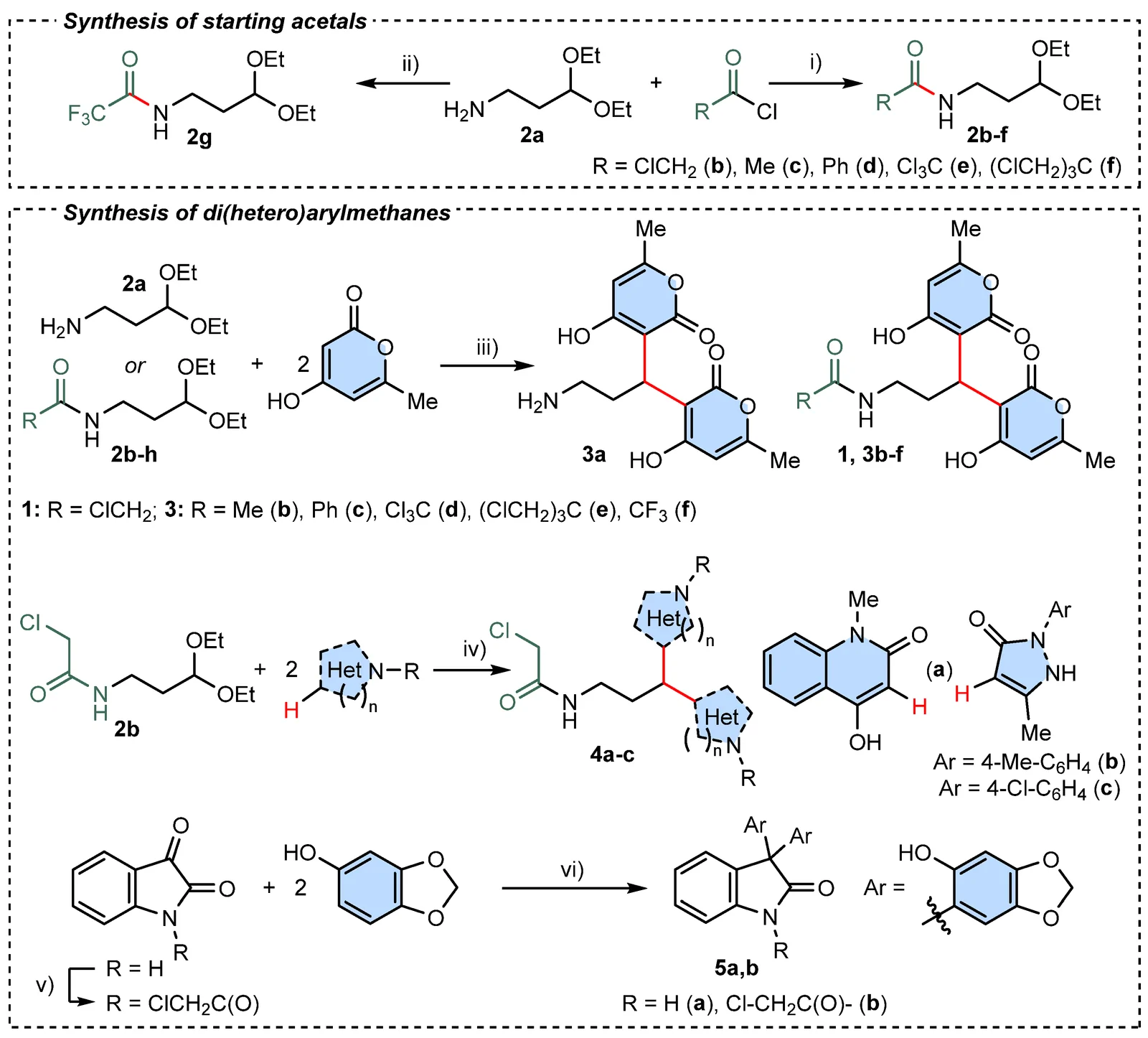
Synthesis of a di(hetero)arylmethane library. (i) Et_3_N (1 eq), CH_2_Cl_2_, −5—−10 °C, 2 h (for **2d–f** Et_3_N (5 eq), C_6_H_6_, rt); (ii) CF_3_COOEt (7 eq), −30 °C, 12 h; (iii) CF_3_COOH (2 mL), CHCl_3_ (10 mL), rt, 48 h; (iv) CF_3_COOH (3 mL), 70 °C, 8–30 h; (v) ClCH_2_C(O)Cl (3.33 eq), 100 °C, 23 h; (vi) CF_3_COOH (3 mL), 70 °C, 24 h.

The syntheses of di(hetero)arylmethane derivatives were carried out following the method previously reported by us (Scheme 1; see the Supplementary Materials, P. S2 for experimental details) [11,12]. Starting from commercially available 3,3-diethoxypropan-1-amine **2a**, the amide derivatives **2b–h** were obtained via reaction with the respective acyl chlorides. The treatment of amine **2a** with ethyl trifluoroacetate afforded the trifluoroacetic acid derivative **2g**.

With the required starting acetals **2** in hand, we carried out the synthesis of the desired compounds **1** and **3a–f** through direct condensation of the acetal with 4-hydroxy-6-methylpyran-2-one in the presence of TFA as a catalyst. The target compounds were obtained in moderate to good yields (41–72%) by simply stirring the reagents in chloroform at room temperature for 48 h.

However, for bis(pyrazol-3-one) derivatives **4b,c**, these reaction conditions proved inappropriate, and no desired product was observed at room temperature regardless of the solvent, catalyst employed, or its amount. Only prolonged refluxing of acetal **2b** with pyrazol-5-ones bearing 4-chlorophenyl and 4-methylphenyl substituents at the nitrogen atom in neat trifluoroacetic acid afforded compounds **4b** and **4c** in 70% and 24% yield, respectively. Notably, other pyrazol-5-one derivatives failed to react even under these harsh conditions. We attribute this failure to the lower nucleophilicity of pyrazol-3-ones, which makes them less prone to electrophilic attack by the intermediate oxonium cation. Not only that, but the protonation of the carbonyl group of pyrazol-3-ones in a strongly acidic medium, similar to that documented for imidazolin-2-ones [13], may render them even less reactive. Thus, the low reactivity of pyrazol-3-ones prevented us from further expanding the scope of bis(pyrazol-3-one)methanes. A similar reactivity problem was encountered in the acid-catalyzed condensation of isatin and *N*-(2-chloroacetyl)isatin **2i** with phenols and electron-rich heterocycles. Only highly nucleophilic sesamol afforded the desired compounds **5a** and **5b** upon refluxing the reagents in neat trifluoroacetic acid; all other tested compounds appeared to be unreactive. Although these synthetic difficulties prevented us from expanding the library of di(hetero)arylmethanes, we considered it sufficient for the intended preliminary cytotoxicity testing.

### 2.2. Activity Screening and SAR Analysis

The in vitro cytotoxicity of newly synthesized di(hetero)arylmethanes was evaluated against the human cancer cell lines HuTu-80 (duodenal adenocarcinoma) and M-HeLa (cervical carcinoma), as well as the normal cell lines WI-38 (fetal lung fibroblast) and Chang liver cells, using the MTT assay after a 48 h treatment. Obtained IC_50_ values (the concentration of the test compound that causes the death of 50% of cells in the experimental population) are summarized in Table 1, with the IC_50_ value of doxorubicin shown for comparison (see Supplementary Materials, P. S24 for details). To get a bigger picture of the influence of a di(hetero)aryl moiety on the activity of compounds under study, we also included IC_50_ values for the previously obtained [11] di(hetero)arylmethanes **4d**–**4g** in the analysis.

We started our SAR investigation by analyzing the effect of the amide moiety on the activity of di(hetero)arylmethanes. For this, the IC_50_ values of the compounds **1** and **3a–g**, which share the common bis(2-oxopyran-3-yl)methane fragment but feature varying amide moieties, were compared. Replacement of the 2-chloroacetamide moiety by acetamide (compound **3b**), benzamide (compound **3c**), trichloro- and trifluoroacetamide (compounds **3d** and **3f**, respectively) resulted in a considerable decrease in cytotoxicity against M-HeLa cells compared to hit compound **1** (IC_50_ = 56.2± 4.7 µM vs. 77–91 µM). In case of the HuTu-80 cell line, the increase in IC_50_ values was much more drastic, rising from 1.9 ± 0.2 µM to 76–106 µM; thus, the activity of modified di(hetero)arylmethanes was 40–50-fold lower than that of the hit compound **1**. The same effect was observed when the amide fragment was completely removed (compound **3a**). Interestingly, the cytotoxicity of amine **3a** did not differ significantly from that of amide derivatives **3b–d** and **3f**, indicating that the amide moiety itself is unlikely to be responsible for the activity of the hit compound **1**. The replacement of the chlorine atom in compound **1** by a triphenylphosphonium group (compound **3g**) resulted in a complete loss of cytotoxicity, suggesting that the chloromethylene group is essential for the di(hetero)arylmethanes to be active. Finally, the tris(chloromethylene)methyl group also rendered compound **3e** inactive. This implies that it is not the amide fragment or the chloromethylene group alone, but a chloroacetamide substituent as a whole that accounts for the activity of the di(hetero)arylmethane **1**. Notably, the cytotoxicity of the above compounds against WI38 and Chang liver cells did not change markedly upon changing the amide substituent. Thus, the 2-chloroacetamide moiety has little effect on the activity against normal cells, while being crucial for the activity against cancer cell lines.

The evaluation of the relationship between the structure of the di(hetero)aryl moiety and the cytotoxicity proved to be much more complex. Replacing the 4-hydroxy-6-methylpyran-2-one fragment in compound **1** with a structurally similar 4-hydroxychromen-2-one moiety (compound **4d**) resulted in a subtle decrease in cytotoxicity against M-HeLa cells (IC_50_ 56.2 ± 4.7 µM vs. 65.3 ± 5.4 µM). At the same time, the activity against HuTu-80 cells showed a 25-fold reduction, with IC_50_ values increasing from 1.9 ± 0.2 μM to 52.1 ± 4.2 μM. Switching from 4-hydroxychromen-2-one (compound **4d**) to 4-hydroxy-1-methylquinolin-2-one (compound **4a**) yielded a considerably more potent derivative, albeit with lower activity than the parent compound **1**. The bis(6-hydroxybenzo[*d*][1,3]dioxol-5-yl)methane derivative **4e** and dibenzoxanthene derivatives **4f,g** also appeared to be less active than the hit compound. Nonetheless, the overall decrease in cytotoxicity was less pronounced in this series of compounds compared to the compounds **3a–g**. This is consistent with our hypothesis about the key role of 2-chloroacetamide moiety in the cytotoxic activity of the di(hetero)arylmethane derivatives. Notably, compound **4a** outperformed the hit compound **1** in cytotoxicity against the M-HeLa cell line (IC_50_ 20.4 ± 1.7 µM vs. 56.2 ± 4.7 µM). Furthermore, bis(3-oxo-2-arylpyrazol-4-yl)methane derivatives **4b,c** were the most potent among the tested compounds, exhibiting *ca* 3-5-fold greater cytotoxicity against both M-HeLa and HuTu-80 cell lines compared to compound **1**. Interestingly, virtually no dependence of cytotoxicity against both cancer and normal cells on the aryl substituent at the nitrogen atom was observed. We have also tested compounds **5a,b**, which possess a conformationally rigid isatin moiety instead of a di(methylene) spacer. Only moderate cytotoxicity against cancer cell lines was observed, thereby indicating the importance of both flexible di(methylene) and 2-chloroacetamide moieties.

Thus, the modification of the di(hetero)aryl scaffold enabled the identification of two novel hit compounds **4b,c** with improved cytotoxic properties. Despite this, no evident correlation between the di(hetero)aryl moiety and cytotoxicity of the studied compounds could be drawn from the data in hand. In principle, several approaches exist, such as ligand-based pharmacophore modeling [14,15] and machine learning methods [16,17], which can be used to relate the structure of a compound to its biological activity. However, these approaches require a sufficiently large compound library, which was inaccessible for us due to several difficulties associated with its synthesis (*vide supra*).

### 2.3. Candidate Target Hypotheses and Covalent Docking

In light of the above, we opted to employ molecular docking methods in the hope of revealing the role of the di(hetero)aryl fragment in the cytotoxicity of the compounds under study. We hypothesized that the 2-chloroacetamide moiety in di(hetero)arylmethanes may act by alkylating a biological target. The well-known ability of the chlorine atom in 2-chloroacetamides to readily undergo nucleophilic substitution upon action of *S*- or *N*-nucleophiles also supports this hypothesis. Thus, we initiated a thorough literature analysis in search of possible targets for such alkylation. As mentioned before, the hit compound **1** was shown to inhibit glycolysis in human ovarian teratocarcinoma PA-1 cells [11]. Taking this into account, we limited our search to targets that are (a) related to the glycolytic pathway, (b) known to have covalent inhibitors, and/or (c) covalently inhibited by compounds structurally related to 2-chloroacetamides.

**Figure 2:**
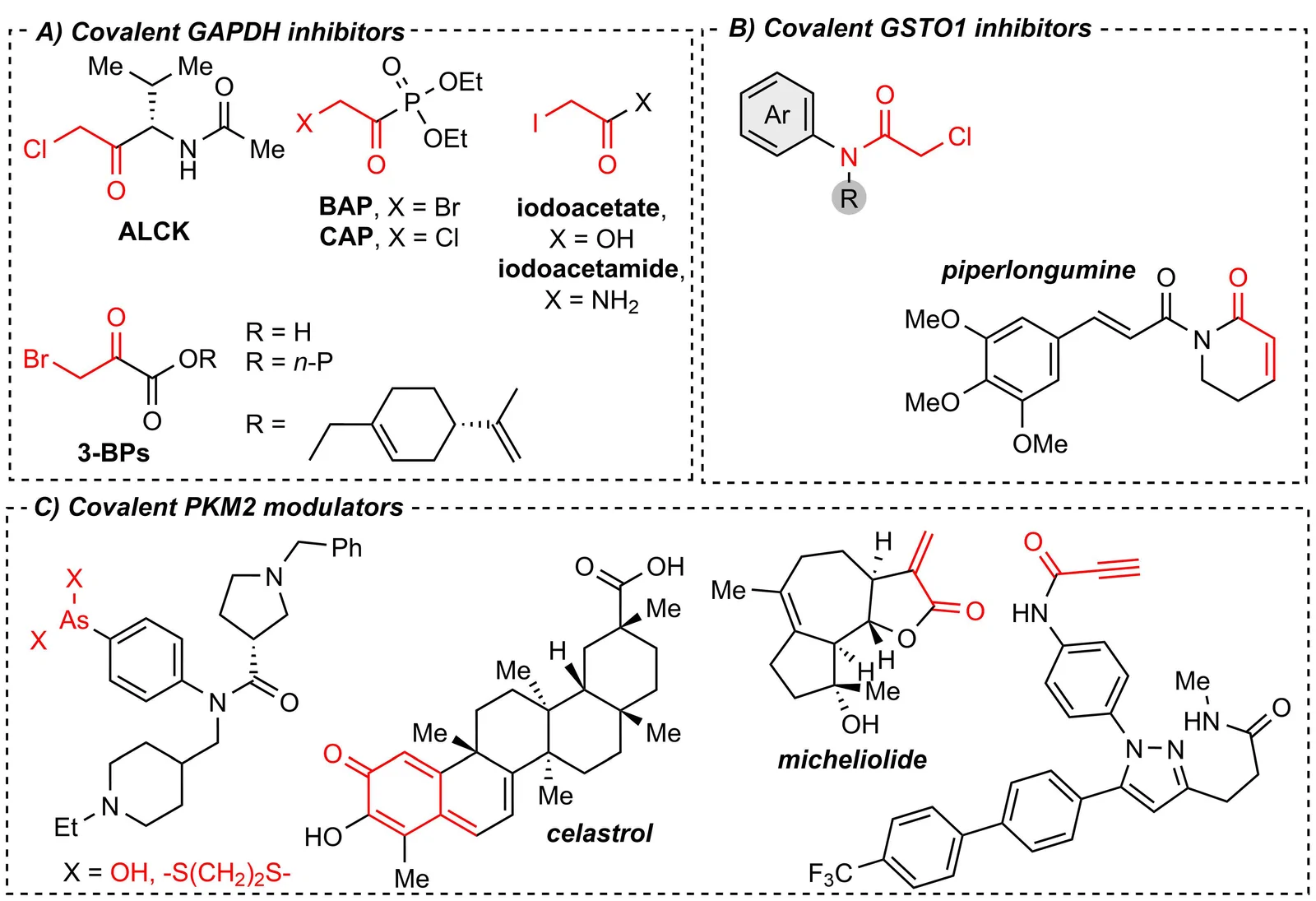
Known covalent GAPDH inhibitors bearing an α-halomethylcarbonyl scaffold [19,20,21,22,23,24,25,26] (**A**), covalent GSTO1 inhibitors [27,28,29] (**B**), and PKM2 modulators acting through irreversible cysteine modification [30,31,32,33,34,35,36] (**C**); the relevant functional groups are highlighted in red.

GAPDH is a key glycolytic enzyme that catalyzes the conversion of glyceraldehyde 3-phosphate to 1,3-bisphosphoglycerate coupled with NAD^+^ reduction to NADH. Several covalent GAPDH inhibitors have been reported [18], with α-halomethyl carbonyl compounds being particularly prominent [19,20,21,22,23,24,25,26]. These compounds are structurally related to our di(hetero)arylmethanes (Figure 2A) and inhibit GAPDH through S_N_2 alkylation of the catalytic cysteine, consistent with our proposed mechanism; notably, they also exhibit significant anticancer activity, supporting GAPDH as a plausible candidate target.

We next considered the M2 splice form of pyruvate kinase (PKM2), which catalyzes the rate-limiting conversion of phosphoenolpyruvate to pyruvate. Several covalent PKM2 modulators have been reported (Figure 2C), most of which irreversibly modify cysteine residues through Michael addition to electrophilic alkenes or alkynes. Micheliolide irreversibly induces PKM2 tetramerization through covalent modification of Cys424 [30]. The pentacyclic triterpenoid celastrol has also been shown to covalently inhibit PKM2 [31,32]. Its anticancer activity is well established [33,34], although not directly attributed to PKM2 inhibition. Synthetic PKM2 inhibitors bearing propiolyl [35] or organoarsenous [36] electrophilic groups have also been reported, further supporting PKM2 as a viable candidate target.

Finally, *N*-aryl-2-chloroacetamides structurally related to our 2-chloroacetamide-derived di(hetero)arylmethanes have been reported as covalent GSTO1 inhibitors [27,28] (Figure 2B). GSTO1 has also been identified as a covalent target of piperlongumine, a naturally occurring compound found in long pepper [29]. In both cases, GSTO1 inhibition proceeds through alkylation of the active-site cysteine via either S_N_2 substitution of the 2-chloroacetamide or Michael addition to the activated double bond. Although GSTO1 is not a glycolytic enzyme, it is highly expressed in aggressive cancer cells [37,38,39] and has been implicated in chemotherapeutic resistance [40,41]. These findings prompted us to consider GSTO1 as an additional plausible candidate target.

**Figure 3:**
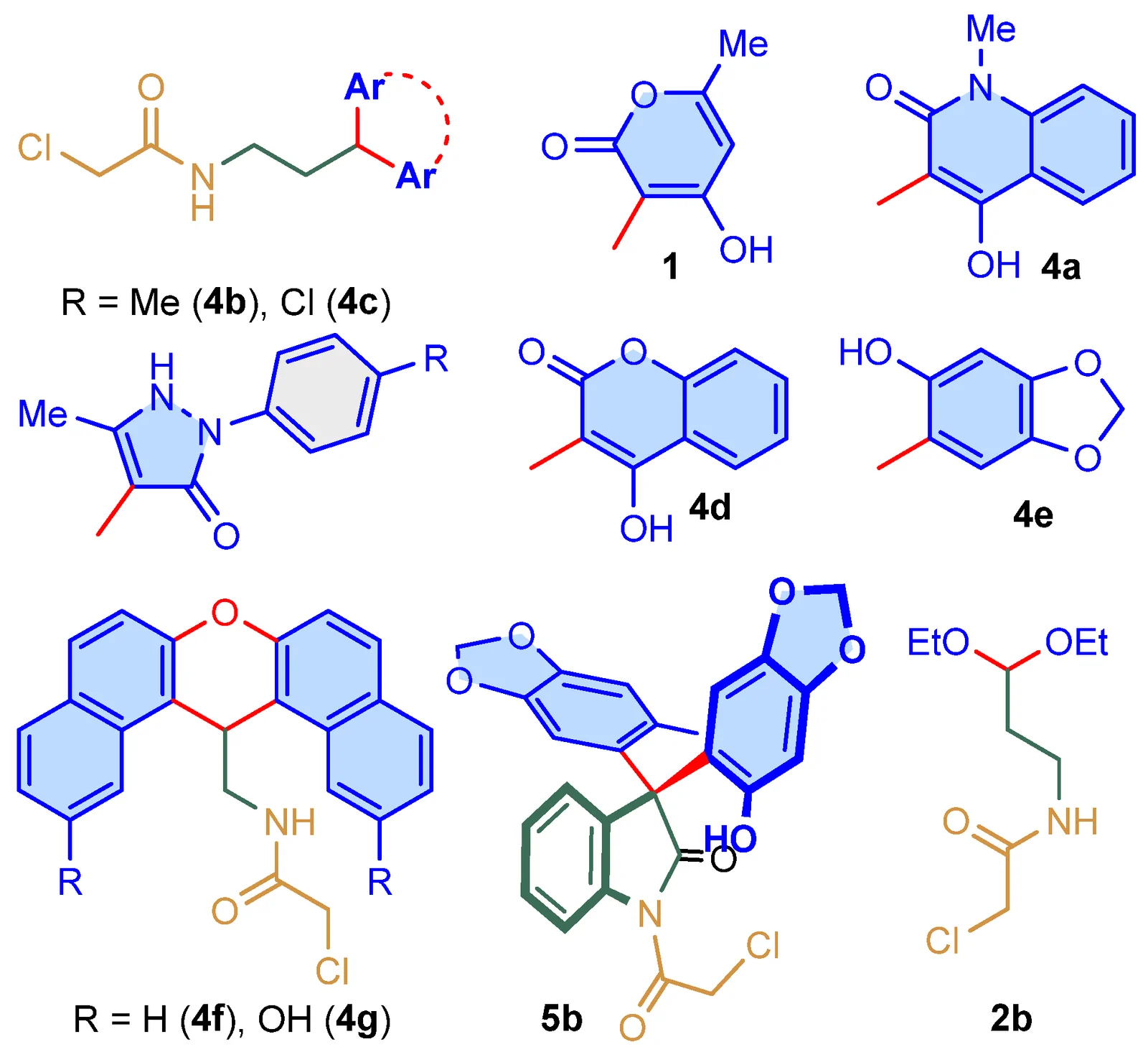
Compounds included in covalent molecular docking studies.

With these candidate target hypotheses formulated, we performed covalent docking studies on a series of di(hetero)arylmethanes to evaluate the influence of di(hetero)aryl moieties on cytotoxicity against the HuTu-80 and M-HeLa cell lines. The purpose of the docking analysis was not to establish the cellular target identity, but to convert a mechanistically plausible target hypothesis into structural constraints for compound design. The ligands for docking were selected from the synthesized library (compounds **4a–c** and **5b**), as well as from compounds previously reported by us (compounds **1** and **4d–g**) [11]. The choice of compounds was driven by the presence of the 2-chloroacetamide moiety and their cytotoxicity against cancer cell lines (IC_50_ < 100 µM). For the compounds **4b,c**, possible tautomers were also accounted for (see Supplementary Materials, P. S15). Additionally, the starting acetal **2b**, which lacks any (hetero)aryl groups, was included in the docking studies as a completely inactive (IC_50_ > 100 µM for all tested cell lines) reference compound (Figure 3). Geometries of the ligands were optimized by the semiempirical GFN2-xTB method [42] using the ORCA 6.0.0 program [43,44]. The optimized structures were further prepared for docking using the Meeko 0.6.1 python package [45].

X-ray structures of a tetramer of PKM2 (PDB code 3GR4), GAPDH (PDB code 6M61) and GSTO1 (PDB code 4YQU) [28] were obtained from the Protein Data Bank. Receptors were prepared for covalent docking by first removing all solvent and ligand molecules, adding hydrogen atoms and charges using ChimeraX 1.11.1 software [46], and processing them with the Meeko 0.6.1 python package. Based on the literature data [18,28,29], the Cys152 and Cys32 residues in the catalytic sites were chosen as the binding sites for GAPDH and GSTO1, respectively. For GAPDH, docking was performed both with NAD^+^ removed from the binding site and with NAD^+^ retained in its native position (hereafter denoted as GAPDH/NAD). In the case of PKM2, several cysteine residues have been reported to undergo covalent modification. The propiolyl-based inhibitor binds to the Cys317 and Cys326 residues near the substrate-binding site of PKM2 [35]. On the contrary, the organoarsenous ligands modify the Cys474 residue located near the allosteric pocket of PKM2 [36]. The Cys31 [32] and Cys424 [31] residues have been reported as attachment sites for celastrol, with the latter also serving as the binding site for micheliolide [30]. Thus, all of these were considered in docking studies. The covalent docking was performed with the AutoDock-GPU 1.6 program [47]. All docking runs were carried out in triplicate to confirm the reproducibility of the predicted binding poses and energies; the mean docking score from the three runs was used in subsequent analysis.

To assess the relationship between predicted binding affinity and activity, docking scores were correlated with cytotoxicity values for 10 compounds across 16 combinations, comprising the GAPDH and GAPDH/NAD, GSTO1, and five PKM2 cysteine sites, evaluated against M-HeLa and HuTu-80 cells. Several strong positive correlations were observed before correction; however, given the number of comparisons, statistical significance was assessed using the Bonferroni correction. After correction, three correlations remained significant: GAPDH/NAD and GAPDH with M-HeLa cytotoxicity (*r* = 0.834, *p*(Bonferroni) = 0.0437; *r* = 0.872, *p*(Bonferroni) = 0.0160, respectively) and GSTO1 with M-HeLa cytotoxicity (*r* = 0.862, *p*(Bonferroni) = 0.0213). None of the PKM2 correlations and none of the GAPDH or GSTO1 correlations with HuTu-80 cytotoxicity remained significant after correction. The three correlations that remained significant after Bonferroni correction were subsequently subjected to leave-one-out sensitivity analysis using Cook’s Distance, with 4/N as the critical threshold. No influential observations were identified, as all data points remained below the corresponding threshold in each analysis. These results were therefore used as a statistical basis for prioritizing GAPDH and GSTO1 as candidate target hypotheses for subsequent structure-based design, rather than as evidence of direct target involvement. The complete correlation and multiple-testing analysis is provided in the Supporting Information, P. S8.

**Figure 4:**
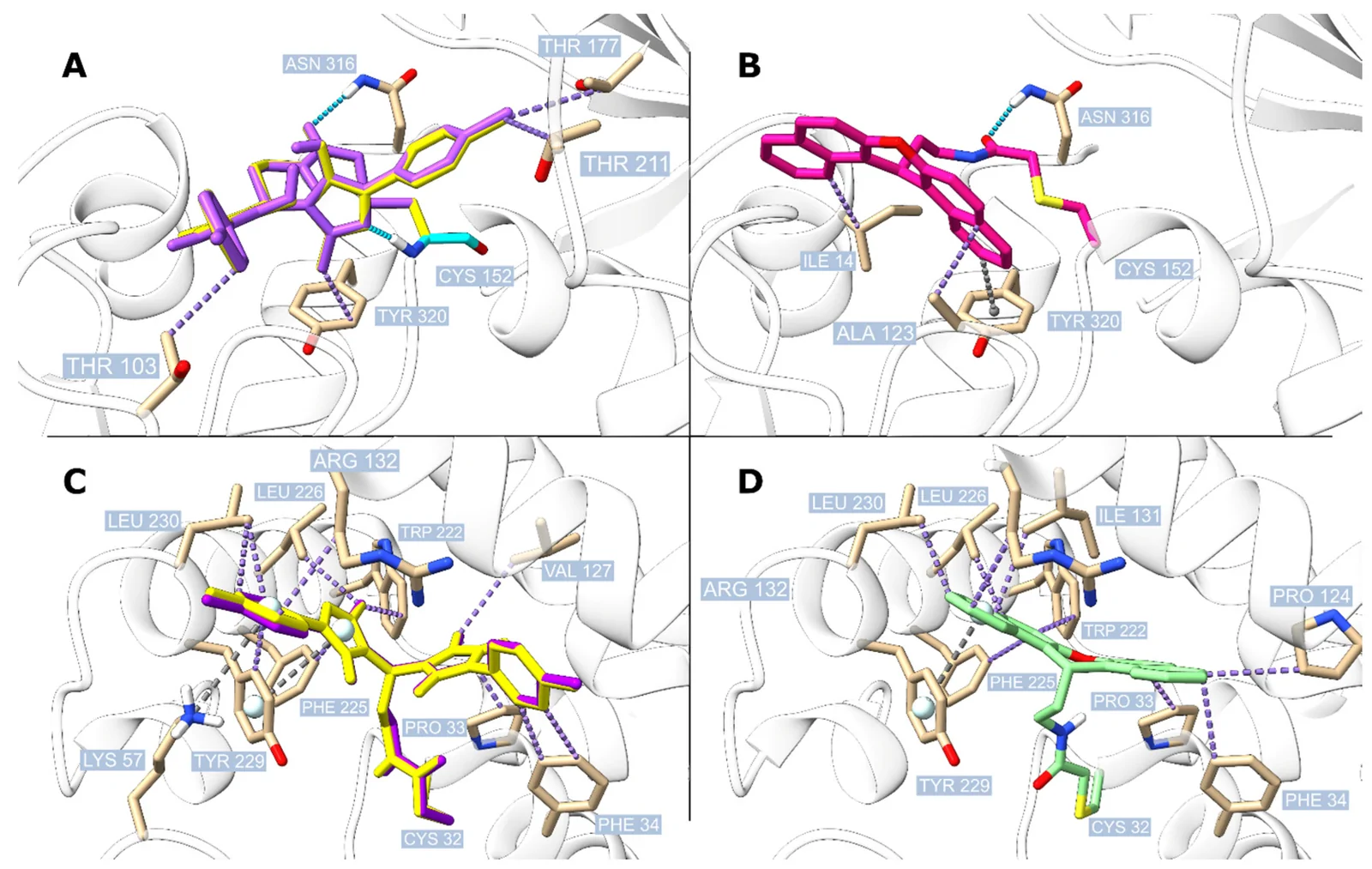
Highest-ranked covalent docking poses (AutoDock-GPU 1.6): compounds **4b** (yellow) and **4c** (magenta) bound to Cys152 of GAPDH (**A**) and Cys32 of GSTO1 (**C**), and compound **4f** bound to Cys152 of GAPDH (**B**) and Cys32 of GSTO1 (**D**). Key hydrogen bonds and hydrophobic interactions are highlighted.

We next analyzed the highest-ranked docking poses of the most potent compounds **4b** and **4c** covalently bound to Cys152 of GAPDH using PLIP v2.4.0 [48] for protein-ligand interaction analysis. Both compounds exhibited nearly identical binding modes, consistent with their highly similar cytotoxicity profiles across the tested cell lines (Figure 4A). The amide carbonyl oxygen atom forms a hydrogen bond with the amide group of Asn316, while the Cys152 residue forms an additional hydrogen bond with the nitrogen atom of one of the pyrazolyl moieties. The two aryl substituents occupy two distinct hydrophobic interaction sites. One aryl substituent establishes hydrophobic interactions with the side chain of Thr103, whereas the other interacts with the side chains of Thr177 and Thr211. In addition, the pyrazolyl moiety forms a hydrophobic contact with Tyr320, reinforcing the first interaction site. For the sake of comparison, the top-ranking pose of the moderately active compound **4f** is depicted in Figure 4B. Only a hydrogen bond with the Asn316 residue is observed in this case, making the binding less effective. Moreover, unlike compounds **4b** and **4c**, compound **4f** engages only one of the two hydrophobic interaction sites, involving Tyr320, Ala123, and Ile14. This suboptimal binding mode leads to a lower docking score, consistent with the lower activity of compound **4f**.

A similar analysis was performed for the covalent docking to the Cys32 residue of GSTO1. As with GAPDH, the highest-ranking poses for compounds **4b** and **4c** were nearly identical (Figure 4C). The two arylpyrazolyl moieties interact with Arg132, Leu230, Tyr229, Trp222, Phe225, Leu226, and Phe34, Pro33, and Val127 residues. In addition, one of the arylpyrazolyl fragments forms π-π stacking and π-cation interactions with Tyr229 and Lys57, respectively. The dibenzoxanthene derivative **4f** also engages in hydrophobic interactions with Arg132, Leu226, Leu230, Ile131, Trp222, Phe225, Pro33, Phe34, and Pro124, as well as π-π stacking with Tyr229 (Figure 4D). However, it lacks π-cation interactions. Consequently, its docking score is lower than those of the bis(pyrazolyl) derivatives **4b,c**, which aligns with its reduced cytotoxicity.

The data above suggest that the pyrazolyl fragments in bis(3-oxo-2-(aryl)pyrazol-4-yl)methanes **4b** and **4c** are responsible for forming hydrogen bonds with GAPDH residues, while the aryl substituents enhance binding both to GAPDH and GSTO1 via hydrophobic and π-stacking interactions. These findings indicate that the di(hetero)aryl moiety plays a crucial role in optimal fitting of the ligand to the binding site. We note that docking studies and observed correlations do not establish or experimentally validate the proposed targets. Rather, they were used to prioritize the candidate target hypotheses and provided a practical structural framework for guiding the rational design of novel scaffolds.

### 2.4. Target-Hypothesis-Guided Design, Synthesis, and Evaluation of Novel Compounds

**Figure 5:**
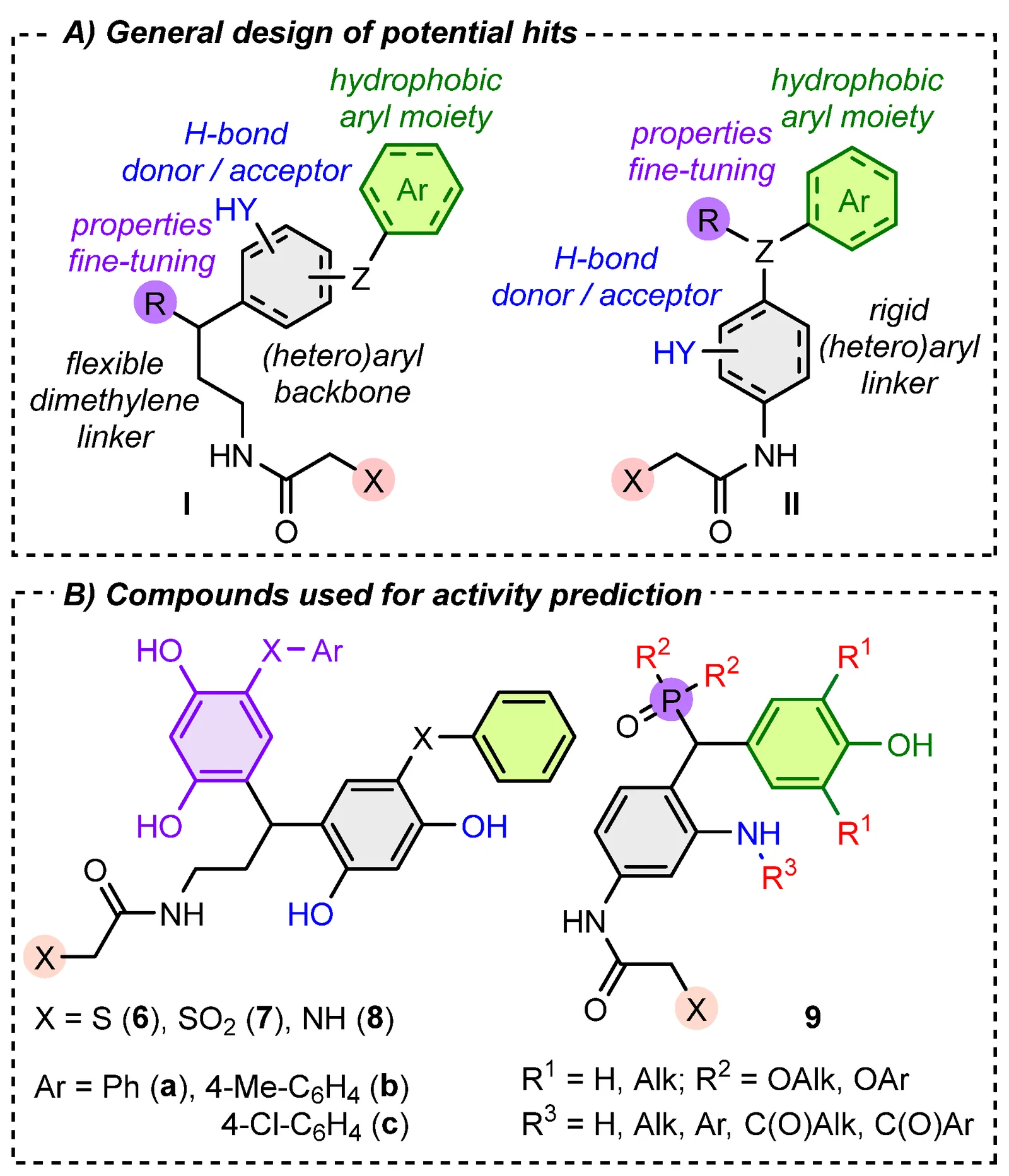
General design of potentially active haloacetamide derivatives **A**) and novel diarylmethanes used in covalent molecular docking studies **B**).

Guided by the above considerations, we proposed a design for novel compounds **I** and **II** with potentially enhanced activity (Figure 5A). The proposed design incorporates a haloacetamide fragment as the reactive warhead for covalent binding to the cysteine residue. Optimal binding and proper orientation of the warhead are ensured by the (hetero)aryl moiety, which features hydrogen bond donor and/or acceptor sites and is connected to a hydrophobic aryl substituent either directly or via a linker. In compounds **I**, the warhead and the affinity anchor are connected via a conformationally flexible dimethylene spacer, chosen on the basis of the data obtained in the present study and our previous results [11]. The design of the compounds **II** was inspired by the covalent GSTO1 inhibitors reported by Cravatt [27] and Neamati [28] (see Figure 2B). We hypothesized that the (hetero)aryl fragment may serve both as the binding motif that provides non-covalent affinity and as a rigid linker for the haloacetamide moiety. Finally, the substituent adjacent to the (hetero)aryl group—which may be a second (hetero)aryl fragment—introduces additional structural diversity and enables fine-tuning of ligand properties (e.g., solubility, lipophilicity, bioavailability, etc.).

We subsequently evaluated how the proposed scaffold modifications affected the predicted covalent binding of the newly designed ligands within the binding sites defined by the leading target hypotheses. Diarylmethane derivatives **6**, **7**, and **8**, bearing aryl sulfide, aryl sulfone, and arylamine substituents, respectively, were selected as representatives of type **I** compounds (Figure 5B). To evaluate the influence of aryl substituents on the docking score, we used unsubstituted phenyl, 4-chlorophenyl, and 4-methylphenyl moieties. We also selected the phosphorylated diarylmethanes **9** to represent type **II** compounds. This choice was guided by their synthetic accessibility [49] and known activity against human cancer cells [50,51,52], with the expectation that incorporation of an additional haloacetamide warhead could enhance cytotoxic activity. The effects of substitution at the phenolic moiety, phosphorus atom, and amino group were investigated by varying the alkyl, aryl, alkoxy, aryloxy, and amide substituents (see Supplementary Materials, P. S21, Figure S16). The designed compounds were modeled and prepared for covalent docking as described above. For compound **9**, both enantiomers were considered. Docking was performed against the Cys152 residue of GAPDH and the Cys32 residue of GSTO1, as these candidate targets showed the highest correlation between experimental cytotoxicity and docking scores.

**Figure 6:**
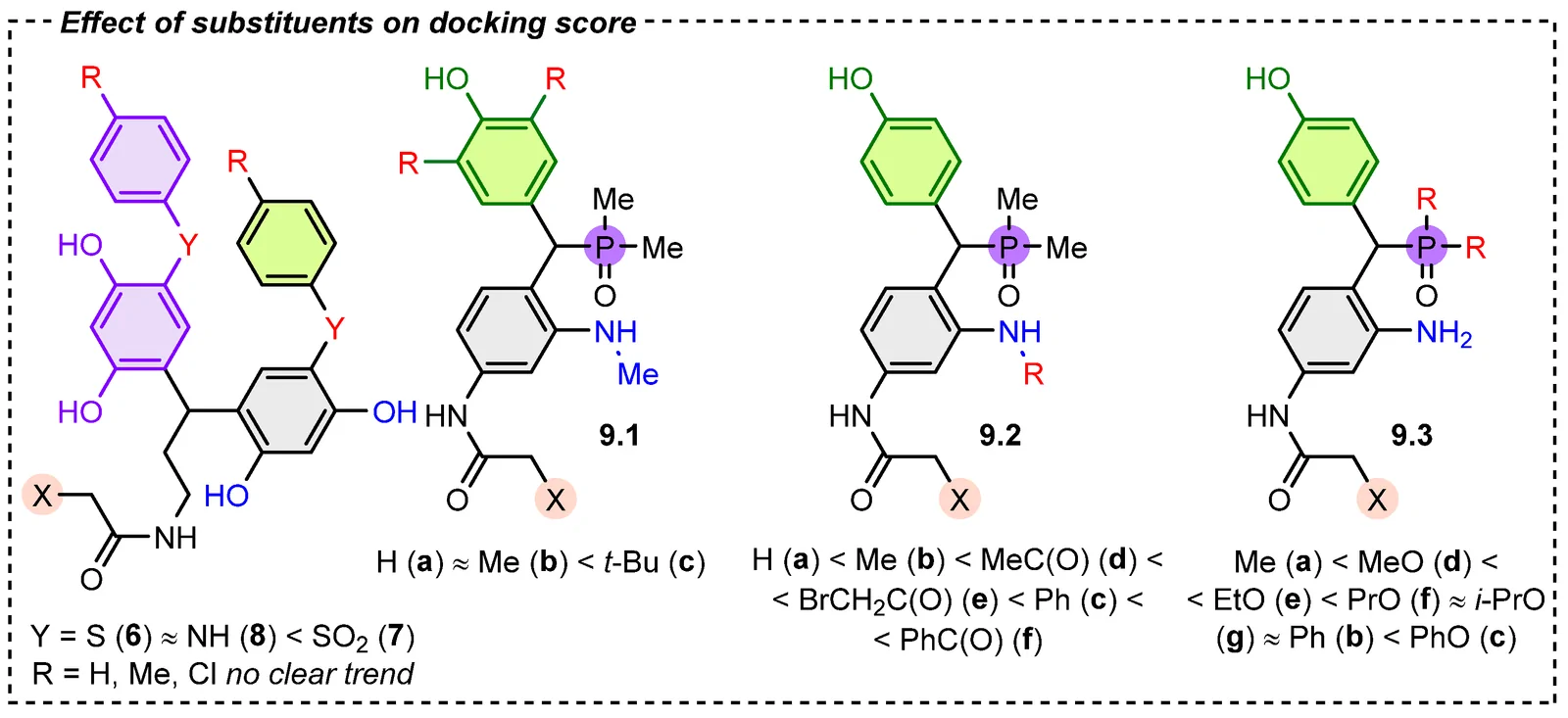
Observed trends in the influence of substituents on the docking scores of diarylmethanes **6–8** and phosphorylated diarylmethanes **9.1–9.3**; varied fragments are highlighted in red.

All diarylmethanes **6–8** outperformed the hit compounds **1** and **4b,c** in terms of docking score, exhibiting scores below −13.70, whereas those of the hit compounds were above –12.9 for both GAPDH and GSTO1. Notably, the structurally related diarylmethane **4e**, bearing two sesamol units, exhibited docking scores above −9.8 (see Supplementary Materials, P. S20, Table S2). This observation is in full agreement with our hypothesis that hydrophobic aryl substituents are important for achieving optimal binding. At the same time, no clear trends were observed for the influence of the *p*-substituent on the aryl fragment on binding affinity (Figure 6). On the contrary, the nature of the linker affected the docking score to some extent, with the sulfonyl group being the most favorable, and sulfur and nitrogen linkers being slightly less effective.

Having established the binding properties of diarylmethanes **6–8**, we subsequently focused on the phosphorylated diarylmethanes **9** to further explore the effects of scaffold modification. Since these compounds possess a stereocenter and thus two enantiomers are possible, both were modeled to assess whether the configuration of the stereocenter affects the predicted binding score. The *S*- and *R*-enantiomers of all compounds **9** exhibited similar docking scores, with differences of *ca* 0.02–1.15 units for GAPDH and GSTO1. Given that the standard error of the AutoDock score is 2–2.5 kcal/mol, these differences were considered negligible and a mean value was used in subsequent analysis as the approximation of the binding affinity of the racemic mixture. Variation in the substituents at the phenolic moiety demonstrated that increasing steric bulk enhances the predicted binding affinity of the compounds **9.1a–c** for both candidate targets (Supplementary Materials, P. S21, Table S3). Introduction of substituents at the nitrogen atom also increased the predicted binding affinity of the modeled compounds **9.2**, following the order H < methyl < acetyl < 2-bromoacetyl < benzoyl < phenyl. Substituents at the phosphorus atom appeared to have the largest effect on the predicted binding affinity. The docking scores for the compounds **9.3** spanned *ca* 4.4–4.6 units, compared with *ca* 2.7–3.1 units for variations in the substituents at the nitrogen atom (compounds **9.2**) and *ca* 2.0–2.1 unit at the phenolic moiety (compounds **9.1**). The phenoxy group afforded the highest affinity, followed by phenyl and the propyloxy and *iso*-propyloxy groups. Further shortening of the alkoxy chain to ethoxy and methoxy progressively weakened the predicted binding. The methyl group produced the highest (least favorable) docking score.

To gain further insight into the combined effects of substituents at the phosphorus and nitrogen atoms on the docking score, we modeled an additional series of phosphorylated diarylmethanes bearing a 4-hydroxy-3,5-di-*tert*-butyl moiety and varying substituents at both positions (compounds **9.4–9.6**, Supplementary Materials, P. S23, Figure S17 and Table S4). The same trends observed for the parent series were reproduced, suggesting that the effects of the substituents are additive and largely independent of the core scaffold. Phosphorus substituents exerted the strongest influence, with phenoxy derivatives affording the most favorable docking scores (**9.4d**; mean score for both enantiomers, −15.02 for GAPDH and −14.14 for GSTO1), followed by propyloxy/iso-propyloxy, ethoxy, and methoxy derivatives (**9.4a**; −11.57/−10.51 for GAPDH/GSTO1), corresponding to a range of *ca* 3.5 score units. Nitrogen substitution produced a smaller but consistent effect of *ca* 1.5–2.5 score units. Among these derivatives, the methyl-substituted compound (**9.4e**) gave the least favorable scores (−12.85/−11.79 for GAPDH/GSTO1), whereas the bromoacetyl (**9.5c**) and benzoyl (**9.5b**) derivatives gave the most favorable scores (−14.39/−13.18 and −14.86/−14.15 for GAPDH/GSTO1, respectively). Compared with the reference compounds **4b** and **4c** (−12.92/−12.48 and −12.94/−12.54 for GAPDH/GSTO1, respectively), most compounds in the **9.4–9.6** series showed more favorable docking scores, with the best combinations, such as **9.6d**, yielding scores below −16 kcal/mol for both plausible targets.

Based on these structural predictions, compounds **9.5c,d,f** and **9.6c,e**, bearing alkoxy and phenoxy substituents at the phosphorus atom, respectively, were selected for prospective experimental evaluation of the target-hypothesis-guided design. Importantly, these compounds were selected and synthesized on the basis of the predicted structural properties of the candidate-target complexes, before their cytotoxicity was determined. Their subsequent biological evaluation therefore provided a prospective test of whether the target-hypothesis-derived structural criteria could successfully guide the discovery of potent compounds.

**Scheme 2:**
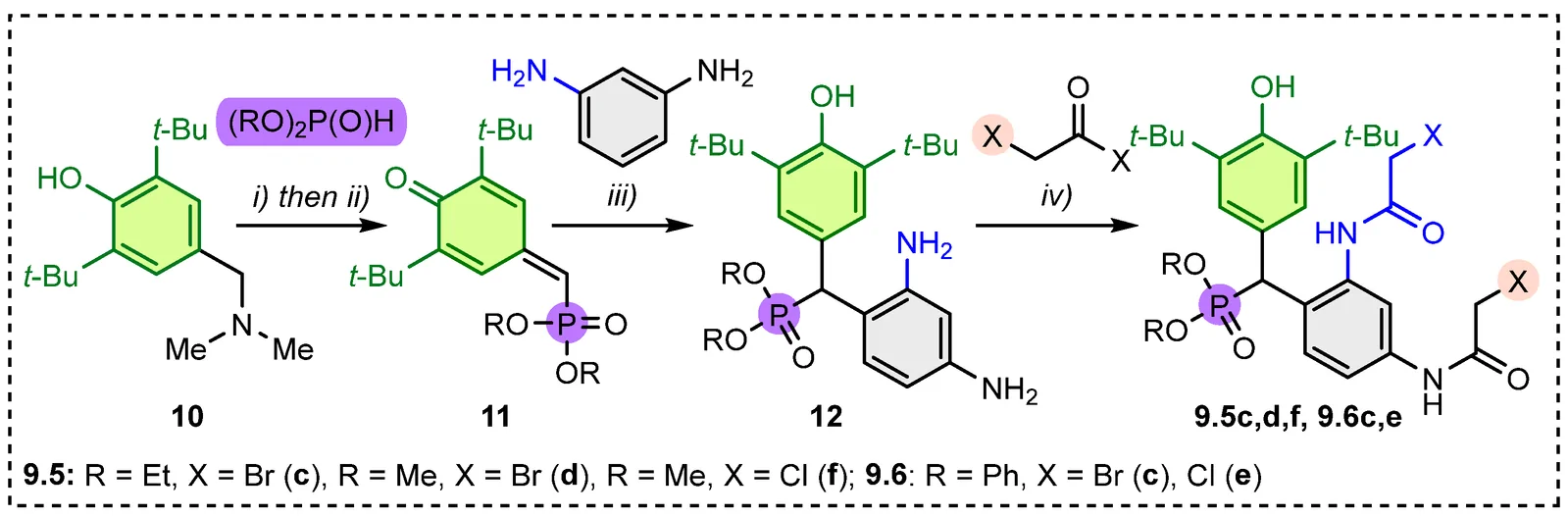
Synthesis of the selected potential hit compounds. (*i*) HP(O)(OR)_2_: R = Me (90–110 °C, 30 min); R = Ph (90–110 °C, 16 h); R = Et (Na, toluene, 110 °C, 1.5 h); (*ii*) K_3_[Fe(CN)_6_], 2N KOH, PhH/H_2_O, rt, 3 h; (*iii*) dioxane, rt, 6h; (*iv*) K_2_CO_3_ (8 equiv.), DCM, rt, 3 h.

The synthetic route was planned around late-stage acylation of intermediates **12** to install the terminal amide groups (Scheme 2). Although benzoyl substituent on the nitrogen atom afforded more favorable docking scores, we opted for haloacetyl moieties to avoid potential selectivity issues during the acylation step. We also introduced both chloroacetamide and the more reactive bromoacetamide moieties to evaluate the effect of this modification on the activity of the resulting compounds.

Because the presence of two haloacetamide fragments gives rise to two possible binding modes, we performed additional docking studies. Interestingly, the regioisomers **9.5c′d′**,**f′**, and **9.6c′,e′**, in which the candidate target is engaged through the haloacetamide group *ortho* to the benzylic phosphonate, consistently showed more favorable docking scores than their *para*-binding counterparts **9.5c,d,f**, and **9.6c,e**, with the difference being particularly pronounced for GSTO1.

With this in hand, the selected compounds were synthesized via phosphorylated quinone methides **11** [53], which are readily accessible from 2,6-di-*tert*-butyl-4-((dimethylamino)methyl)phenol **10** and dialkyl H-phosphonates. Reaction of **11** with *m*-phenylenediamine afforded the key intermediates **12** [54], which were subsequently acylated with haloacetyl halides to furnish the target compounds in 68–75% yield.

We next evaluated the cytotoxicity of the synthesized racemic compounds against a panel of cancer and normal cell lines (Table 2; see Supplementary Materials, p. S26, Table S5 for full data). The chloroacetamide derivatives **9.5f** and **9.6e** were more active against M-HeLa cells than compound **1**, but remained less potent than compounds **4b,c**. By contrast, the bromoacetamide analogues **9.5c,d** and **9.6c** displayed *ca* 24–60-fold higher activity against M-HeLa cells, although their activity against HuTu-80 cells was somewhat lower. Nevertheless, compounds **9.5c,d** and **9.6c** showed cytotoxicity in the same range as doxorubicin against both cancer cell lines, with **9.5d** exhibiting the highest potency. The increased activity of the bromoacetamides was accompanied by greater effects on normal cells, indicating a concomitant reduction in the separation between antitumor activity and general cytotoxicity.

The pronounced activity of the bromoacetamide derivatives is consistent with their greater electrophilic reactivity and with the proposed covalent mode of action. At the same time, the prospective compounds differ from the single-warhead compounds used to establish the docking–cytotoxicity relationships by incorporating two haloacetamide warheads. Importantly, these compounds were docked considering both covalent attachment sites, thereby allowing the potential contribution of either warhead to the predicted binding modes to be assessed. Thus, the observed potency is likely to reflect the combined influence of the scaffold features identified through the structure-based analysis and the intrinsic properties of the prospective molecules, including warhead number and electrophilicity. The latter factors may also increase nonspecific protein modification and therefore preclude assigning the enhanced cytotoxicity to any single structural feature. This consideration is particularly relevant to **9.5d**, which showed high potency against M-HeLa cells (IC_50_ = 0.9 µM) but only a limited difference in activity toward WI-38 cells (IC_50_ = 1.7 µM). Taken together, the cytotoxicity data are consistent with the proposed design rationale and support further exploration of these scaffolds, while the relative contributions of scaffold architecture, warhead multiplicity, and electrophilicity remain to be established experimentally.

### 2.5. Additional Biological Studies of the Hit Compound ***9.5d***

To complement the in silico target hypotheses and to characterize the functional metabolic consequences of treatment, we evaluated the impact of compound **9.5d** on lactate dehydrogenase (LDH) and pyruvate dehydrogenase (PDH) in PANC-1 pancreatic ductal adenocarcinoma cells. LDH activity ensures NAD^+^ regeneration and completes anaerobic glycolysis, and its activity correlates with tumor invasiveness and metastasis [55]. In contrast, the mitochondrial PDH complex catalyzes the irreversible oxidative decarboxylation of pyruvate, directing it into the tricarboxylic acid (TCA) cycle. Suppression of LDH activity and activation of PDH are associated with reduced tumor potential and reversal of the Warburg effect [56], making both enzymes relevant phenotypic reporters of glycolytic perturbation.

**Figure 7:**
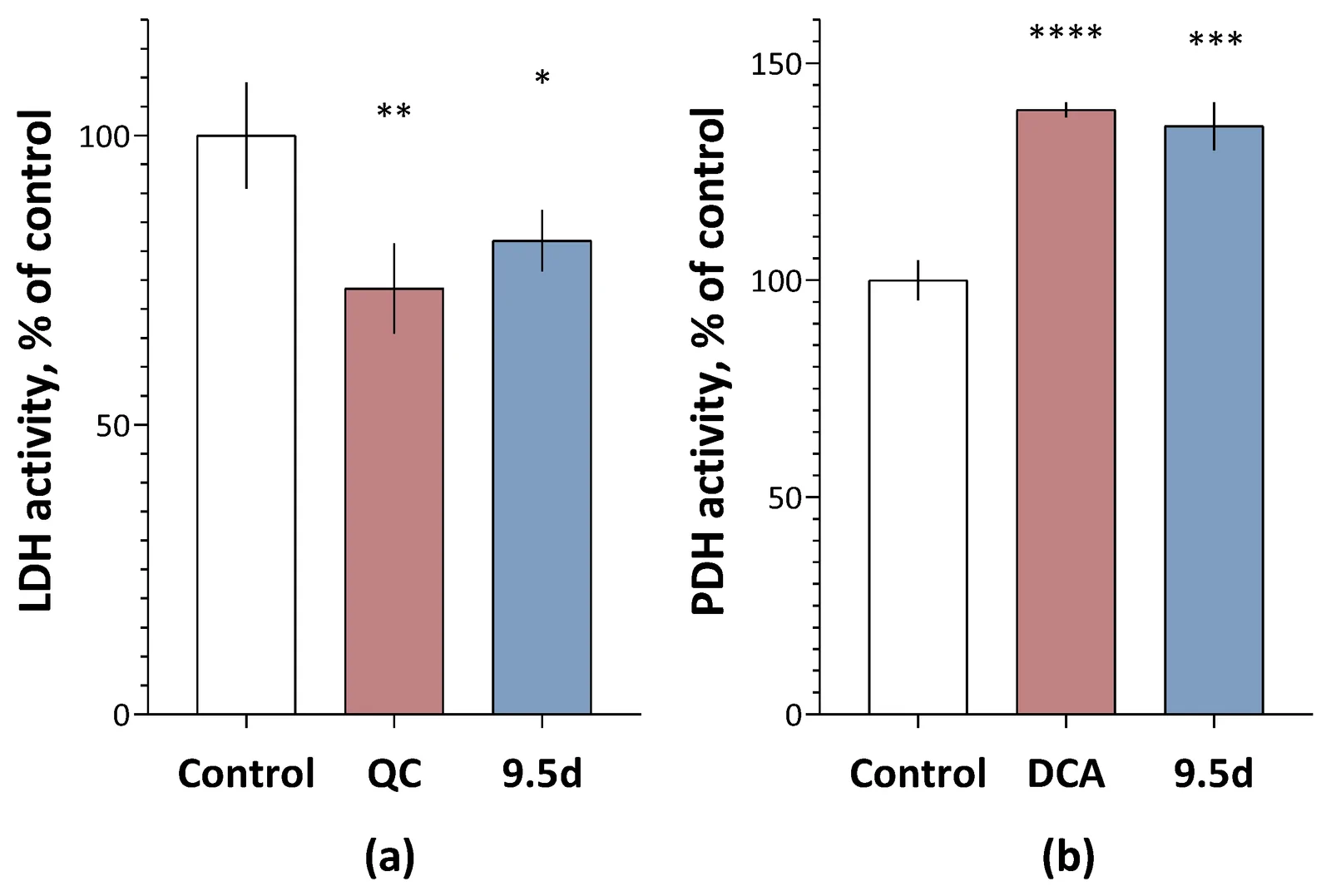
Effect of compound **9.5d** at a concentration of 1 µM on (**a**) lactate dehydrogenase (LDH) and (**b**) pyruvate dehydrogenase (PDH) activities in PANC-1 cells. Cells were incubated with the compounds for 48 h. Results are expressed as a percentage of the control (1% DMSO): Mean ± SEM (n = 3). Statistical significance was determined by one-way analysis of variance (ANOVA) followed by Dunnett’s post hoc test. * *p* < 0.05, ** *p* < 0.01, *** *p* < 0.001, **** *p* < 0.0001 compared to the control.

As shown in Figure 7a, incubation of PANC-1 pancreatic ductal adenocarcinoma cells with the reference flavonoid quercetin (QC) at a concentration of 20 µM for 48 h resulted in a 26% decrease in enzyme activity. This is consistent with previously published data, which provided the rationale for selecting this concentration [55]. In turn, the compound **9.5d** at a concentration of *ca* 0.9 µM, corresponding to 1/2 of the IC_50_ previously established by us in the MTT assay on this cell line, led to an 18% decrease in LDH activity. No less important results were obtained when evaluating the effect of **9.5d** on the pyruvate dehydrogenase complex. To validate the experimental system, a classical inhibitor of pyruvate dehydrogenase kinases—sodium dichloroacetate (DCA)—was used as a positive control. As shown in Figure 7b, incubation of PANC-1 cells with DCA at a concentration of 1 mM (which corresponds to the range of its therapeutic concentrations in vitro) resulted in a 39.3% increase in PDH enzymatic activity relative to the control. In turn, the **9.5d** at 0.9 µM demonstrated a comparable stimulatory effect, increasing PDH activity to virtually the same extent as a millimolar dose of DCA.

Taken together, the data obtained indicate that compound **9.5d** contributes to a decrease in lactate dehydrogenase activity and an increase in pyruvate dehydrogenase activity. This may reflect a redistribution of pyruvate towards oxidative metabolism. A similar metabolic profile is characteristic of compounds that can limit the production of glycolytic energy, contributing to oxidative stress and mitochondrial dysfunction, which can subsequently trigger apoptosis.

An important limitation of the present study is that the molecular targets responsible for the cellular activity of the compounds were not experimentally established. Accordingly, GAPDH and GSTO1 should be regarded as candidate targets rather than definitive cellular targets. The purpose of incorporating these proteins into the study was not to resolve target identity, but to test whether structurally plausible covalent target hypotheses could provide useful guidance for compound design. The prospective activity of the newly designed scaffolds supports the utility of this approach, while direct target identification remains an important subject for future investigation.

## 3. Materials and Methods

### 3.1. General

The ^1^H and ^13^C NMR spectra were recorded on a Bruker Avance 600 spectrometer (Bruker, Billerica, MA, USA) (operating frequency 600 MHz and 150 MHz, respectively), Bruker Avance 500 spectrometer (operating frequency 500 MHz and 126 MHz, respectively) (Bruker, Billerica, MA, USA) and Bruker Avance 400 spectrometer (operating frequency 400 MHz and 101 MHz, respectively) (Bruker, Billerica, MA, USA) with respect to the residual proton signals of deuterated solvents (DMSO-*d_6_*, CDCl_3_). The ^31^P NMR spectra were recorded on Bruker Avance 600 and Bruker Avance 400 spectrometers (operating frequencies of 243 MHz and 162 MHz, respectively) with 85% H_3_PO_4_ as the external standard. The IR spectra were recorded on a Vector 22 Fourier spectrometer by Bruker (Billerica, MA, USA) in the range of 400–4000 cm^−1^ from KBr pellets. The melting points were determined in glass capillaries on a Stuart SMP 10 instrument (Stuart Scientific Co. Ltd., Stone, UK). The halogen content was determined by the Schöniger method.

The high-resolution mass spectra (HRMS) with electrospray ionization (ESI) were obtained using an ESI-QTOF Impact II mass spectrometer with an Elute UHPLC system (Bruker Daltonik GmbH, Bremen, Germany). The column YMC-Triart C18 (50 × 2.0 mm; 3 μm) was used. The column thermostat temperature was set at 40 °C and the autosampler temperature at 12 °C. Elution solvents Milli-Q water (A) + 0.1% formic acid and HPLC-grade acetonitrile+ 0.1% formic acid (B) were used, and the elution gradient was the following: 0 min at 5% B, 3 min at 95% B, 4 min at 95% B, 4.1 min at 5% B, 6 min at 5% B with a flow rate of 0.3 mL/min. The injection volume was 2 μL. Measurements were made in positive mode in the range *m*/*z* 50–1900. The ESI source conditions were as follows: capillary voltage 4500 V, desolvation temperature 220 °C, drying gas nitrogen at a flow rate of 6 L/min. The samples were prepared in HPLC-grade acetonitrile to a concentration of 0.005 mg/mL. The sodium iodide solution in Milli-Q water (0.2 mg/mL) was used as a calibrant. The Hystar (Bruker Daltonik GmbH, Bremen, Germany, version 6.0) and the otofControl (Bruker Daltonik GmbH, Bremen, Germany, version 5.2) programs were used to control the chromatograph and mass spectrometer. Data processing was performed by DataAnalysis software (Bruker Daltonik GmbH, Bremen, Germany, version 5.3).

All commercially available reagents were used as received for the reactions without any purification. All solvents were purified and dried according to standard procedures. Dialkyl/diphenylphosphonates **12** were synthesized according to the previously reported procedure [54].

### 3.2. Synthesis of Starting Compounds

#### 3.2.1. Synthesis of Acetals **2b,c**

A solution of 3,3-diethoxypropan-1-amine (2.00 g, 1.41 mL, 17.70 mmol, 1 equiv.) and triethylamine (1.80 g, 2.45 mL, 17.70 mmol, 1 equiv.) in anhydrous dichloromethane (15 mL) was cooled to between −5 and −10 °C. A solution of the corresponding acid chloride (1.0 equiv.) in anhydrous dichloromethane (10 mL) was added dropwise with stirring. After complete addition, the reaction mixture was stirred between −5 and −10 °C for 2 h and then allowed to warm to room temperature. The precipitate was removed by filtration, and the filtrate was concentrated under reduced pressure. The residue was dissolved in chloroform (20 mL) and washed with distilled water (5 × 4 mL). The organic layer was dried over anhydrous Na_2_SO_4_, filtered, and concentrated under reduced pressure to afford the target compounds **2b** or **2c**.

*2-Chloro-N-(3,3-diethoxypropyl)acetamide* (**2b**). Yellow oil; 2.85 g (72%). **^1^H NMR** (500 MHz, CDCl_3_) δ 7.33 (s, 1H), 4.54 (t, *J* = 4.9 Hz, 1H), 3.97 (s, 2H), 3.65 (dq, *J* = 9.3, 7.0 Hz, 2H), 3.47 (dq, *J* = 9.4, 7.0 Hz, 2H), 3.41–3.30 (m, 2H), 1.84–1.75 (m, 2H), 1.18 (t, *J* = 7.1 Hz, 6H). **^13^C{^1^H} NMR** (126 MHz, CDCl_3_) δ 160.1, 97.0, 56.6, 37.0, 30.1, 26.9, 9.6. **IR** (liquid, cm^−1^) ν 1059, 1127, 1538, 1667, 1721, 2883, 2976, 3306. **MS (ESI)** *m*/*z*: calcd 223.1; found 224.4 [M + H]^+^. **Anal. Calcd** for C_9_H_18_ClNO_3_: C, 48.32; H, 8.11; Cl, 15.85; N, 6.26. Found: C, 48.29; H, 8.13; Cl, 15.87; N, 6.24.

*N-(3,3-diethoxypropyl)acetamide* (**2c**). Yellow oil; 2.25 g (67%). **^1^H NMR** (500 MHz, CDCl_3_) δ 6.35 (s, 1H), 4.50 (t, *J* = 5.2 Hz, 1H), 3.62 (dq, *J* = 9.4, 7.0 Hz, 2H), 3.45 (dq, *J* = 9.4, 7.0 Hz, 2H), 3.28 (q, *J* = 5.8 Hz, 2H), 1.89 (s, 3H), 1.81–1.70 (m, 2H), 1.16 (t, *J* = 7.0 Hz, 6H). **^13^C{^1^H} NMR** (126 MHz, CDCl_3_) δ 164.4, 96.7, 56.3, 29.9, 27.3, 17.5, 9.6. **IR** (liquid, cm^−1^) ν 1064, 1373, 1435, 3305. **MS (ESI)** *m*/*z*: calcd 189.1; found 189.9 [M + H]^+^. **Anal. Calcd** for C_9_H_19_NO_3_: C, 57.12; H, 10.12; N, 7.40. Found: C, 57.10; H, 10.14; N, 7.42.

#### 3.2.2. Synthesis of Acetals **2d–f**

To a stirred solution of 3,3-diethoxypropan-1-amine (1.00 g, 0.75 mL, 6.80 mmol, 1 equiv.) and triethylamine (3.44 g, 4.68 mL, 33.96 mmol, 5.0 equiv) in anhydrous benzene (20 mL), a solution of the corresponding acid chloride (1.0 equiv) in anhydrous benzene (10 mL) was added dropwise. The reaction mixture was stirred at room temperature for 2 h. The precipitate was removed by filtration, and the filtrate was concentrated under reduced pressure. The residue was dissolved in chloroform (20 mL), washed with distilled water (5 × 4 mL), dried over anhydrous Na_2_SO_4_, filtered, and concentrated under reduced pressure to afford acetals **2d-f**.

*N-(3,3-diethoxypropyl)benzamide* (**2d**). Yellow oil; 0.85 g (47%). **^1^H NMR** (400 MHz, CDCl_3_) δ 7.81–7.73 (m, 2H), 7.51–7.32 (m, 3H), 7.11 (s, 1H), 4.64 (t, *J* = 5.0 Hz, 1H), 3.71 (dq, *J* = 9.4, 7.1 Hz, 2H), 3.62–3.54 (m, 2H), 3.58–3.46 (m, 2H), 1.94 (ddd, *J* = 7.8, 6.2, 4.9 Hz, 2H), 1.22 (t, *J* = 7.1 Hz, 6H). **^13^C{^1^H} NMR** (126 MHz, CDCl_3_) δ 167.2, 134.7, 131.2, 128.4, 126.8, 102.7, 62.1, 35.9, 32.8, 15.3. **IR** (liquid, cm^−1^) ν 1491, 1544, 1638, 3082, 3295. **MS (ESI)** *m*/*z*: calcd 251.2; found 252.4 [M + H]^+^. **Anal. Calcd** for C_14_H_21_NO_3_: C, 66.91; H, 8.42; N, 5.57. Found: C, 66.90; H, 8.43; N, 5.59.

*2,2,2-trichloro-N-(3,3-diethoxypropyl)acetamide* (**2e**). Yellow oil; 0.65 g (32%). **^1^H NMR** (400 MHz, CDCl_3_) δ 7.75 (s, 1H), 4.63 (t, *J* = 4.7 Hz, 1H), 3.72 (dq, *J* = 9.4, 7.0 Hz, 2H), 3.58–3.47 (m, 4H), 1.97–1.86 (m, 2H), 1.23 (t, *J* = 7.0 Hz, 6H). **^13^C{^1^H} NMR** (126 MHz, CDCl_3_) δ 161.6, 102.7, 92.8, 62.6, 37.2, 32.1, 15.3. **IR** (liquid, cm^−1^) ν 1097, 3324. **MS (ESI)** *m*/*z*: calcd 291.0; found 292.2 [M + H]^+^. **Anal. Calcd** for C_9_H_16_Cl_3_NO_3_: C, 36.95; H, 5.51; Cl, 36.35; N, 4.79. Found: C, 36.98; H, 5.50; Cl, 36.32; N, 4.80.

*3-chloro-2,2-bis(chloromethyl)-N-(3,3-diethoxypropyl)*propenamide (**2f**). Yellow oil; 1.31 g (57%). **^1^H NMR** (600 MHz, CDCl_3_) δ 4.60 (t, *J* = 5.0 Hz, 1H), 3.86 (s, 6H), 3.70 (dq, *J* = 9.5, 7.1 Hz, 2H), 3.52 (dq, *J* = 9.5, 7.0 Hz, 2H), 3.45 (q, *J* = 5.4 Hz, 2H), 1.88–1.83 (m, 2H), 1.24 (t, *J* = 7.1 Hz, 6H). **^13^C{^1^H} NMR** (126 MHz, CDCl_3_) δ 162.9, 96.5, 56.6, 48.6, 38.7, 30.6, 27.4, 9.9. **MS (ESI)** *m*/*z*: calcd 337.1; found 338.0 [M + H]^+^. **Anal. Calcd** for C_12_H_22_Cl_3_NO_3_: C, 43.07; H, 6.63; Cl, 31.78; N, 4.19. Found: C, 43.10; H, 6.60; Cl, 31.77; N, 4.20.

#### 3.2.3. Synthesis of *N*-(3,3-Diethoxypropyl)-2,2,2-trifluoroacetamide (**2g**, Procedure Is Adopted from [57])

Ethyl trifluoroacetate (4.40 g, 3.68 mL, 31.0 mmol, 4.5 equiv.) was added dropwise to neat 3,3-diethoxypropan-1-amine (1.00 g, 0.75 mL, 6.80 mmol, 1 equiv.) cooled to –30 °C. The reaction mixture was stirred at −30 °C for 12 h. The solvent was then removed under reduced pressure to afford the corresponding trifluoroacetamide **2g** as a yellow oil. Yield 1.43 g (82%). **^1^H NMR** (400 MHz, CDCl_3_) δ 4.58 (t, *J* = 4.6 Hz, 1H), 3.72–3.63 (m, 2H), 3.53–3.42 (m, 4H), 1.90–1.81 (m, 2H), 1.21–1.16 (t, *J* = 7.1 Hz, 6H). **^13^C{^1^H} NMR** (126 MHz, CDCl_3_) δ 157.0 (q, *J* = 36.9 Hz), 116.0 (q, *J* = 287.3 Hz), 102.6, 62.8, 35.7, 31.9, 15.0. **MS (ESI)** *m*/*z*: calcd 243.1; found 244.3 [M + H]^+^. **Anal. Calcd** for C_9_H_16_F_3_NO_3_: C, 44.44; H, 6.63; N, 5.76. Found: C, 44.47; H, 6.61; N, 5.74.

#### 3.2.4. Synthesis of 1-(2-Chloroacetyl)indoline-2,3-dione (**2i**, Procedure Is Adopted from [58])

A mixture of indoline-2,3-dione (3.00 g, 20.39 mmol, 1.0 equiv) and chloroacetyl chloride (10.00 g, 88.54 mmol, 7.00 mL, 3.3 equiv) was refluxed for 23 h. The reaction mixture was then concentrated under reduced pressure. The residue was triturated with diethyl ether, and the resulting solid was collected by filtration through a sintered-glass funnel, washed with diethyl ether (25 mL), and dried under reduced pressure to afford the title compound as an orange solid. Yield 3.84 g (85%); m.p. 210–212 °C. **^1^H NMR** (600 MHz, DMSO-*d_6_*) δ 11.25 (s, 1H), 8.15 (dd, J = 8.7, 1.2 Hz, 1H), 7.73–7.65 (m, 2H), 7.33 (td, J = 7.6, 1.1 Hz, 1H), 4.37 (s, 2H). The characterization data is in accordance with previously reported [59].

### 3.3. Synthesis of Compounds ***3***, ***4*** and ***5***

#### 3.3.1. Synthesis of Compounds **1** and **3**

To a solution of the corresponding acetal (6.71 mmol, 1.0 equiv) in chloroform (10 mL) were added 4-hydroxy-6-methyl-2H-pyran-2-one (1.69 g, 13.41 mmol, 2.0 equiv) and trifluoroacetic acid (2 mL). The reaction mixture was stirred at room temperature for 48 h and then concentrated under reduced pressure. The residue was thoroughly washed with diethyl ether (4 × 20 mL). The resulting solid was collected by filtration through a sintered-glass funnel and dried under reduced pressure to afford target compounds **3a–g**.

*N-(3,3-bis(4-hydroxy-6-methyl-2-oxo-2H-pyran-3-yl)propyl)-2-chloroacetamide* (**1**). Yellow solid; 1.80 g (70%); m.p. 130–132 °C. **^1^H NMR** (400 MHz, DMSO-*d_6_*) δ 8.09 (t, J = 5.5 Hz, 1H), 6.15–5.81 (m, 2H), 4.41 (t, J = 8.1 Hz, 1H), 3.99 (s, 2H), 3.03–2.87 (m, 2H), 2.08 (t, J = 7.8 Hz, 2H). The characterization data is in accordance with previously reported [11].

*3,3-bis(4-hydroxy-6-methyl-2-oxo-2H-pyran-3-yl)propan-1-aminium trifluoroacetate* (**3a**). Yellow solid; 1.09 g (50%); m.p. 118–121 °C. **^1^H NMR** (400 MHz, DMSO-*d_6_*) δ 7.71 (d, *J* = 7.0 Hz, 3H), 6.04–5.88 (m, 2H), 4.30 (t, *J* = 8.1 Hz, 1H), 2.66 (dt, *J* = 10.8, 5.6 Hz, 2H), 2.25 (td, *J* = 12.1, 10.1, 6.3 Hz, 2H), 2.11 (s, 6H). **^13^C{^1^H} NMR** (101 MHz, DMSO-*d_6_*) δ 167.3, 165.0, 158.5 (q, J = 32.6), 116.8 (q, J = 297.0), 101.4, 101.1, 88.1, 38.4, 28.7, 28.6, 19.1. **HRMS (ESI)** *m*/*z*: [M-CF3COO^−^]^+^ calcd for C_15_H_18_NO_6_ 308.1129; found 308.1129. **Anal. Calcd** for C_17_H_18_F_3_NO_8_: C, 48.46; H, 4.31; N, 3.32. Found: C, 48.47; H, 4.33; N, 3.30.

*N-(3,3-bis(4-hydroxy-6-methyl-2-oxo-2H-pyran-3-yl)propyl)acetamide* (**3b**). Light yellow solid; 0.85 g (65%); m.p. 182–186 °C. **^1^H NMR** (400 MHz, DMSO-*d_6_*) δ 7.69 (t, *J* = 4.5 Hz, 1H), 5.98 (d, *J* = 1.1 Hz, 2H), 4.42 (t, *J* = 8.1 Hz, 1H), 2.88 (q, *J* = 7.6, 6.9 Hz, 2H), 2.19–2.09 (s, 6H), 2.10–1.97 (m, 2H), 1.74 (s, 3H). **^13^C{^1^H} NMR** (126 MHz, DMSO-*d_6_*) δ 168.8, 167.1, 165.7, 160.4, 102.2, 101.1, 37.8, 29.8, 28.1, 22.6, 19.0. **HRMS (ESI)** *m*/*z*: [M + H]^+^ calcd for C_17_H_20_NO_7_ 350.1234; found 350.1234; [M + Na]^+^ calcd for C_17_H_19_NO_7_Na 372.1054; found 372.1054. **Anal. Calcd** for C_17_H_19_NO_7_: C, 58.45; H, 5.48; N, 4.01. Found: C, 58.43; H, 5.50; N, 4.04.

*N-(3,3-bis(4-hydroxy-6-methyl-2-oxo-2H-pyran-3-yl)propyl)benzamide* (**3c**). Light yellow solid; 0.54 g (57%); m.p. 181–188 °C. **^1^H NMR** (600 MHz, DMSO-*d_6_*) δ 8.36 (t, *J* = 5.4 Hz, 1H), 7.82–7.76 (m, 2H), 7.50 (td, *J* = 7.3, 1.4 Hz, 1H), 7.44 (td, *J* = 7.5, 1.5 Hz, 2H), 5.99 (s, 2H), 4.57–4.46 (m, 1H), 3.14 (dt, *J* = 8.4, 6.2 Hz, 2H), 2.24–2.18 (m, 2H), 2.14 (s, 6H). **^13^C{^1^H} NMR** (126 MHz, DMSO-*d_6_*) δ 167.2, 165.8, 160.4, 134.8, 130.9, 128.1, 127.1, 102.3, 101.2, 64.9, 38.4, 29.6, 28.0, 19.0. **HRMS (ESI)** *m*/*z*: [M + H]^+^ calcd for C_22_H_22_NO_7_ 412.1391; found 412.1389; [M + Na]^+^ calcd for C_22_H_21_NO_7_Na 434.1210; found 434.1209. **Anal. Calcd** for C_22_H_21_NO_7_: C, 64.23; H, 5.15; N, 3.40. Found: C, 64.21; H, 5.17; N, 3.37.

*N-(3,3-bis(4-hydroxy-6-methyl-2-oxo-2H-pyran-3-yl)propyl)-2,2,2-trichloroacetamide* (**3d**). Beige solid; 0.77 g (72%); m.p. 126–129 °C. **^1^H NMR** (600 MHz, DMSO-*d_6_*) δ 8.90 (t, *J* = 5.6 Hz, 1H), 5.97 (s, 2H), 4.40 (t, *J* = 8.1 Hz, 1H), 3.11–3.02 (q, *J* = 6.8 Hz, 2H), 2.19–2.15 (m, 2H), 2.13 (s, 6H). **^13^C{^1^H} NMR** (126 MHz, DMSO-*d_6_*) δ 165.5, 161.2, 160.3, 128.3, 102.0, 101.1, 92.9, 30.7, 29.2, 28.1, 19.0. **HRMS (ESI)** *m*/*z*: [M + H]^+^ calcd for C_17_H_17_Cl_3_NO_7_ 452.0065; found 452.0064; [M + Na]^+^ calcd for C_17_H_16_Cl_3_NO_7_Na 473.9885; found 473.9882. **Anal. Calcd** for C_17_H_16_Cl_3_NO_7_: C, 45.11; H, 3.56; Cl, 23.49; N, 3.09. Found: C, 45.14; H, 3.53; Cl, 23.51; N, 3.07.

*N-(3,3-bis(4-hydroxy-6-methyl-2-oxo-2H-pyran-3-yl)propyl)-3-chloro-2,2-bis(chloromethyl)propanamide* (**3e**). Beige solid; 0.33 g (54%); m.p. 110–111 °C. **^1^H NMR** (500 MHz, DMSO-*d_6_*) δ 8.04 (t, *J* = 5.5 Hz, 1H), 6.00 (d, *J* = 1.1 Hz, 2H), 4.45 (t, *J* = 8.1 Hz, 1H), 3.85 (s, 6H), 2.98 (ddt, *J* = 7.9, 5.4, 3.3 Hz, 2H), 2.17–2.14 (m, 6H), 2.14–2.08 (m, 2H). **^13^C{^1^H} NMR** (126 MHz, DMSO-*d_6_*) δ 167.4, 167.1, 165.6, 160.4, 102.0, 101.0, 53.2, 44.0, 38.6, 29.5, 28.1, 19.0. **HRMS (ESI)** *m*/*z*: [M + H]^+^ calcd for C_20_H_23_Cl_3_NO_7_ 494.0535; found 494.0531; [M + Na]^+^ calcd for C_20_H_22_Cl_3_NO_7_Na 516.0354; found 516.0350. **Anal. Calcd** for C_20_H_22_Cl_3_NO_7_: C, 48.55; H, 4.48; Cl, 21.50; N, 2.83. Found: C, 48.53; H, 4.49; Cl, 21.48; N, 2.85.

*N-(3,3-bis(4-hydroxy-6-methyl-2-oxo-2H-pyran-3-yl)propyl)-2,2,2-trifluoroacetamide* (**3f**). Beige solid; 0.34 g (41%); m.p. 107–111 °C. **^1^H NMR** (400 MHz, DMSO-*d_6_*) δ 9.28 (t, *J* = 5.6 Hz, 1H), 5.97 (s, 2H), 4.41 (t, *J* = 8.1 Hz, 1H), 3.07 (q, *J* = 6.6 Hz, 2H), 2.22–2.15 (m, 2H), 2.14 (s, 6H). **^13^C{^1^H} NMR** (101 MHz, DMSO-*d_6_*) δ 167.05, 165.40, 160.31, 155.9 (q, J = 35.7 Hz), 115.9 (q, J = 288.3 Hz), 101.86, 101.00, 38.34, 28.88, 28.07, 19.01. **HRMS (ESI)** *m*/*z*: [M + H]^+^ calcd for C_17_H_17_F_3_NO_7_ 404.0952; found 404.0952; [M + Na]^+^ calcd for C_17_H_16_F_3_NO_7_Na 426.0771; found 426.0770. **Anal. Calcd** for C_17_H_16_F_3_NO_7_: C, 50.63; H, 4.00; F, 14.13; N, 3.47. Found: C, 50.65; H, 4.01; F, 14.11; N, 3.48.

#### 3.3.2. Synthesis of Compounds **4**

A mixture of acetal **2b** (0.40 g, 1.79 mmol, 1.0 equiv) and the corresponding heterocyclic compound (3.59 mmol, 2.0 equiv) in trifluoroacetic acid (3 mL) was refluxed for 8–30 h. The reaction mixture was then concentrated under reduced pressure. The residue was thoroughly washed with diethyl ether (4 × 20 mL). The resulting solid was collected by filtration through a sintered-glass funnel and dried under reduced pressure to afford the desired products **4a–c**.

*N-(3,3-bis(4-hydroxy-1-methyl-2-oxo-1,2-dihydroquinolin-3-yl)propyl)-2-chloroacetamide* (**4a**). Beige solid; 0.19 g (22%); m.p. 203–207 °C. **^1^H NMR** (400 MHz, DMSO-*d_6_*) δ 8.22–8.12 (m, 1H), 8.06 (dd, *J* = 8.1, 1.5 Hz, 2H), 7.73–7.61 (m, 4H), 7.38 (t, *J* = 7.4 Hz, 2H), 4.78 (t, *J* = 7.7 Hz, 1H), 3.93 (s, 2H), 3.72 (s, 6H), 3.02 (q, *J* = 6.7 Hz, 2H), 2.56 (q, *J* = 7.7 Hz, 2H). **^13^C{^1^H} NMR** (126 MHz, DMSO-*d_6_*) δ 165.7, 161.0, 159.9, 137.9, 131.3, 123.4, 122.7, 115.2, 110.9, 97.9, 42.5, 38.3, 30.8, 30.1, 27.9. **HRMS (ESI)** *m*/*z*: [M + H]^+^ calcd for C_25_H_25_ClN_3_O_5_ 482.1477; found 482.1474; [M + Na]^+^ calcd for C_25_H_24_ClN_3_O_5_Na 504.1297; found 504.1294. **Anal. Calcd** for C_25_H_24_ClN_3_O_5_: C, 62.31; H, 5.02; Cl, 7.36; N, 8.72. Found: C, 62.30; H, 5.05; Cl, 7.37; N, 8.74.

*N-(3,3-bis(5-methyl-3-oxo-2-(p-tolyl)-2,3-dihydro-1H-pyrazol-4-yl)propyl)-2-chloroacetamide* (**4b**). Yellow solid; 0.63 g (70%); m.p. 171–172 °C. **^1^H NMR** (500 MHz, DMSO-*d_6_*) δ 8.24 (t, *J* = 5.4 Hz, 1H), 7.56 (d, *J* = 8.5 Hz, 4H), 7.28–7.23 (m, 4H), 4.04 (s, 2H), 3.58 (t, *J* = 7.9 Hz, 1H), 3.05 (q, *J* = 6.5 Hz, 2H), 2.32 (s, 6H), 2.24 (s, 6H), 2.00–1.92 (m, 2H). **^13^C{^1^H} NMR** (126 MHz, DMSO-*d_6_*) δ 165.9, 157.3, 145.0, 135.5, 134.1, 129.4, 121.0, 105.1, 42.7, 37.7, 30.7, 25.7, 20.5, 11.1. **HRMS (ESI)** *m*/*z*: [M + H]^+^ calcd for C_27_H_31_ClN_5_O_3_ 508.2110; found 508.2108; [M + Na]^+^ calcd for C_27_H_30_ClN_5_O_3_Na 530.1929; found 530.1926. **Anal. Calcd** for C_27_H_30_ClN_5_O_3_: C, 63.84; H, 5.95; Cl, 6.98; N, 13.79. Found: C, 63.85; H, 5.93; Cl, 6.97; N, 13.80.

*N-(3,3-bis(2-(4-chlorophenyl)-5-methyl-3-oxo-2,3-dihydro-1H-pyrazol-4-yl)propyl)-2-chloroacetamide* (**4c**). Beige solid; 0.24 g (24%); m.p. 204–209 °C. **^1^H NMR** (400 MHz, DMSO-*d_6_*) δ 8.19 (t, *J* = 5.4 Hz, 1H), 7.85–7.65 (m, 4H), 7.51 (d, *J* = 8.9 Hz, 4H), 4.03 (s, 2H), 3.55 (t, *J* = 7.9 Hz, 1H), 3.04 (q, *J* = 6.5 Hz, 2H), 2.22 (s, 6H), 2.16 (q, *J* = 7.2 Hz, 2H). **^13^C{^1^H} NMR** (126 MHz, DMSO-*d_6_*) δ 165.9, 157.4, 135.9, 129.6, 128.9, 121.8, 105.3, 42.6, 37.7, 30.6, 25.8, 11.4 **HRMS (ESI)** *m*/*z*: [M + H]^+^ calcd for C_25_H_25_Cl_3_N_5_O_3_ 548.1017; found 548.1018; [M + Na]^+^ calcd for C_25_H_24_Cl_3_N5O_3_Na 570.0837; found 570.0838. **Anal. Calcd** for C_25_H_24_Cl_3_N_5_O_3_: C, 54.71; H, 4.41; Cl, 19.38; N, 12.76. Found: C, 54.72; H, 4.43; Cl, 19.36; N, 12.75.

#### 3.3.3. Synthesis of Compounds **5**

A mixture of indoline-2,3-dione (0.10 g, 0.68 mmol, 1.0 equiv) or 1-(2-chloroacetyl)indoline-2,3-dione (0.30 g, 1.35 mmol, 1.0 equiv) and benzo[*d*][1,3]dioxol-5-ol (0.19 g, 1.36 mmol, 2.0 equiv. or 0.37 g, 2.71 mmol, 2.0 equiv., respectively) in trifluoroacetic acid (3 mL) was refluxed for 24 h. The reaction mixture was then concentrated under reduced pressure. The residue was sequentially washed with water, benzene, and diethyl ether. The resulting solid was collected by filtration through a sintered-glass funnel and dried under reduced pressure to afford the desired products **5a** or **5b**.

*3,3-bis(6-hydroxybenzo[d]*[1,3]*dioxol-5-yl)indolin-2-one* (**5a**). Beige solid; 0.04 g (16%); m.p. 227–231 °C. **^1^H NMR** (500 MHz, DMSO-*d_6_*) δ 10.93 (s, 1H), 9.98 (s, 1H), 9.35 (s, 1H), 7.26 (td, *J* = 7.7, 1.3 Hz, 1H), 7.03 (td, *J* = 7.5, 1.1 Hz, 1H), 6.96 (d, *J* = 7.7 Hz, 1H), 6.89 (d, *J* = 7.3 Hz, 1H), 6.54 (s, 1H), 6.30 (s, 1H), 6.23 (d, *J* = 11.6 Hz, 2H), 5.96 (s, 1H), 5.90 (s, 3H). **^13^C{^1^H} NMR** (126 MHz, DMSO-*d_6_*) δ 183.5, 152.6, 149.4, 147.8, 146.8, 141.7, 140.4, 139.6, 132.1, 128.2, 125.8, 122.0, 117.2, 110.1, 107.9, 101.2, 100.9, 97.7, 59.3. **HRMS (ESI)** *m*/*z*: [M + H]^+^ calcd for C_22_H_16_NO_7_ 406.0921; found 406.0920; [M + Na]^+^ calcd for C_22_H_15_NO_7_Na 428.0741; found 428.0738. **Anal. Calcd** for C_22_H_15_NO_7_: C, 65.19; H, 3.73; N, 3.46. Found: C, 65.21; H, 3.72; N, 3.45.

*1-(2-chloroacetyl)-3,3-bis(6-hydroxybenzo[d]*[1,3]*dioxol-5-yl)indolin-2-one* (**5b**). Brown solid; 0.38 g (59%); m.p. 184–186 °C. **^1^H NMR** (500 MHz, DMSO-*d_6_*) δ 9.91 (s, 1H), 9.77 (s, 1H), 7.73 (d, *J* = 8.0 Hz, 1H), 7.47 (t, *J* = 7.7 Hz, 1H), 7.36 (s, 2H), 7.23 (t, *J* = 7.7 Hz, 1H), 7.10 (s, 1H), 6.98 (d, *J* = 8.0 Hz, 1H), 6.66 (s, 1H), 6.36 (s, 1H), 6.16 (s, 1H), 6.09 (s, 1H), 6.03 (s, 1H), 5.88 (s, 2H), 4.12 (d, *J* = 14.9 Hz, 1H), 3.72 (d, *J* = 15.0 Hz, 1H). **^13^C{^1^H} NMR** (126 MHz, DMSO-*d_6_*) δ 179.3, 163.8, 148.4, 147.7, 147.4, 146.5, 144.4, 140.1, 135.8, 129.9, 129.1, 128.3, 127.5, 125.9, 120.5, 116.2, 106.2, 106.0, 101.7, 101.0, 97.8, 94.0, 57.7, 42.5. **HRMS (ESI)** *m*/*z*: [M + H]^+^ calcd for C_24_H_17_ClNO_8_ 482.0637; found 482.0636; [M + Na]^+^ calcd for C_24_H_16_ClNO_8_Na 504.0457; found 504.0450. **Anal. Calcd** for C_24_H_16_ClNO_8_: C, 59.83; H, 3.35; N, 2.91; Cl, 7.36. Found: C, 59.85; H, 3.33; N, 2.90; Cl, 7.37.

#### 3.3.4. Synthesis of Compounds **9.5c–f** and **9.6c,e**

To a solution of the corresponding phosphonate **12** (1 mmol) in dichloromethane (5 mL), a solution of the corresponding 2-haloacyl halide (8 mmol) in dichloromethane (1 mL) was added dropwise. Potassium carbonate (8 mmol) was then added, and the reaction mixture was stirred at room temperature for 2–3 h. The resulting inorganic salts were removed by filtration, and the solvent was evaporated under reduced pressure. Distilled water was added to the resulting oil, and the mixture was allowed to stand for 3 days. The precipitated solid was collected by filtration, washed with water, and dried in a vacuum desiccator over P_2_O_5_ (4 h, 23 °C, 15 mm Hg) to give the target compounds.

*Diethyl[(2,4-bis(2-bromoacetamido)phenyl)(3,5-di-tert-butyl-4-hydroxyphenyl)methyl]phosphonate* (**9.5c**). White solid; 73%. M.p. 110–111 °C. IR (KBr, cm^−1^) ν 562, 771, 1025, 1049, 1210, 1607, 1680, 3409, 3627. **^1^H NMR** (600 MHz, DMSO-*d_6_*) δ 1.02 (t, *J* = 7.0 Hz, 3H), 1.03 (t, *J* = 7.0 Hz, 3H), 1.32 (s, 18H), 3.73 (m, 1H), 3.80 (m, 1H), 3.85 (m, 2H), 4.02 (s, 2H), 4.05 (q, *J* = 26.1 Hz, 2H), 4.76 (d, *J* = 26.3 Hz, 1H), 6.82 (s, 1H), 7.19 (s, 2H), 7.46 (dd, *J* = 9.0, 1.9 Hz, 1H), 7.68 (s, 1H), 7.73 (d, *J* = 8.0 Hz, 1H), 9.84 (s, 1H), 10.42 (s, 1H). **^13^C{^1^H} NMR** (101 MHz, DMSO-*d_6_*) δ 16.7 (d, *J* = 5.1 Hz), 16.8 (d, *J* = 6.1 Hz), 29.7, 29.9, 30.8, 34.9, 46.8 (d, *J* = 138.4 Hz), 63.9 (d, *J* = 6.1 Hz), 64.0 (d, *J* = 7.1 Hz), 117.7, 118.3, 125.4 (d, *J* = 4.0 Hz), 126.4 (d, *J* = 6.1 Hz), 126.7, 126.7, 131.9 (d, *J* = 8.1 Hz), 136.0 (d, *J* = 7.2 Hz), 136.7, 137.6, 153.6, 164.9, 165.9. **^31^P{^1^H} NMR** (243 MHz, DMSO-*d_6_*) δ 26.3. **Anal. Calcd** for C_29_H_41_Br_2_N_2_O_6_P: C, 55.77; H, 6.62; Br, 12.79; N, 4.49; P, 4.96. Found: C, 55.75; H, 6.64; Br, 12.78; N, 4.50; P, 4.97. **HRMS (ESI)** *m*/*z*: calcd for [M + H]^+^ C_29_H_41_Br_2_N_2_O_6_P 703.1142; found 703.1145.

*Dimethyl[(2,4-bis(2-bromoacetamido)phenyl)(3,5-di-tert-butyl-4-hydroxyphenyl)methyl]phosphonate* (**9.5d**). White solid; 70%. M.p. 146–167 °C. IR (KBr, cm^−1^) ν 565, 768, 1035, 1055, 1212, 1608, 1684, 3268, 3632. **^1^H NMR** (600 MHz, DMSO-*d_6_*) δ 1.41 (s, 18H), 3.59 (d, *J* = 10.7 Hz, 3H), 3.69 (d, *J* = 10.8 Hz, 3H), 3.86 (s, 2H), 3.96 (s, 2H), 4.61 (d, *J* = 26.6 Hz, 1H), 4.78 (s, 1H), 7.19 (s, 2H), 7.34 (d, *J* = 8.5 Hz, 1H), 7.67 (s, 1H), 7.72 (d, *J* = 8.5 Hz, 1H), 8.50 (s, 1H), 9.70 (s, 1H). **^13^C{^1^H} NMR** (101 MHz, DMSO-*d_6_*) δ 29.7, 29.8, 30.8, 34.9, 42.2 (d, *J* = 140.4 Hz), 54.2 (d, *J* = 9.1 Hz), 54.3 (d, *J* = 9.1 Hz), 117.8, 118.3, 125.3, 126.2, 126.6 (d, *J* = 6.8 Hz), 132.1 (d, *J* = 2.0 Hz), 136.2 (d, *J* = 8.1 Hz), 136.8, 137.7, 153.7, 164.7, 165.6. **^31^P{^1^H} NMR** (243 MHz, DMSO-*d_6_*) δ 29.1. **Anal. Calcd** for C_27_H_37_Br_2_N_2_O_6_P: C, 47.95; H, 5.51; Br, 23.63; N, 4.14; P, 4.58. Found: C, 47.95; H, 5.50; Br, 23.64; N, 4.17; P, 4.60. **HRMS (ESI)** *m*/*z*: [M + Na]^+^ calcd for C_27_H_37_Br_2_N_2_NaO_6_P 697.0654; found 697.0652; [M + H]^+^ calcd for C_27_H_38_Br_2_N_2_O_6_P 675.0829; found 675.0831.

*Dimethyl[(2,4-bis(2-chloroacetamido)phenyl)(3,5-di-tert-butyl-4-hydroxyphenyl)methyl]phosphonate* (**9.5f**). White solid; 75%. M.p. 166–168 °C. IR (KBr, cm^−1^) ν 629, 768, 1032, 1049, 1238, 1604, 1686, 3397, 3582. **^1^H NMR** (600 MHz, DMSO-*d_6_*) δ 1.34 (s, 18H), 3.49 (d, *J* = 10.3 Hz, 3H), 3.50 (d, *J* = 10.3 Hz, 3H), 4.23 (s, 4H), 4.81 (d, *J* = 25.9 Hz, 1H), 6.87 (s, 1H), 7.20 (s, 2H), 7.48 (d, *J* = 9.1 Hz, 1H), 7.70 (s, 1H), 7.73 (d, *J* = 8.0 Hz, 1H), 9.82 (s, 1H), 9.96 (s, 1H). **^13^C{^1^H} NMR** (151 MHz, DMSO-*d_6_*) δ 30.8, 35.0, 42.2 (d, *J* = 141.3 Hz), 43.4, 43.4, 53.3 (d, *J* = 6.5 Hz), 53.5 (d, *J* = 6.5 Hz), 117.5, 117.4, 126.1 (d, *J* = 6.8 Hz), 127.2, 127.9, 130.4, 136.0 (d, *J* = 10.5 Hz), 137.9, 139.4, 153.1, 165.2, 165.6. **^31^P{^1^H} NMR** (243 MHz, DMSO-*d_6_*) δ 29.5. **Anal. Calcd** for C_27_H_37_Cl_2_N_2_O_6_P: C, 55.20; H, 6.35; Cl, 12.07; N, 4.77; P, 5.27. Found: C, 55.35; H, 6.36; Cl, 12.10; N, 4.78; P, 5.28. **HRMS (MALDI-TOF/TOF)** *m*/*z*: calcd for [M + H]^+^ C_27_H_38_Cl_2_N_2_O_6_P 587.1839; found 587.1842.

*Diphenyl[(2,4-bis(2-bromoacetamido)phenyl)(3,5-di-tert-butyl-4-hydroxyphenyl)methyl]phosphonate* (**9.6c**). White solid; 68% yield; m.p. 93–94 °C. IR (KBr, cm^−1^) ν 584, 764, 938, 1239, 1598, 1679, 3269, 3628. **^1^H NMR** (600 MHz, DMSO-*d_6_*) δ 1.29 (s, 18H), 4.01 (s, 2H), 4.10 (q, *J* = 26.1 Hz, 2H), 5.25 (d, *J* = 27.3 Hz, 1H), 6.59 (d, *J* = 8.1 Hz, 2H), 6.96 (d, *J* = 6.9 Hz, 2H), 7.08 (t, *J* = 7.4 Hz, 1H), 7.15 (t, *J* = 7.4 Hz, 1H), 7.18 (t, *J* = 7.5 Hz, 1H), 7.30 (m, 4H), 7.50 (d, *J* = 10.0 Hz, 1H), 7.72 (s, 1H), 7.90 (d, *J* = 8.9 Hz, 1H), 9.97 (s, 1H), 10.47 (s, 1H). **^13^C{^1^H} NMR** (101 MHz, DMSO-*d_6_*) δ 29.7, 29.8, 30.7, 34.9, 46.4 (d, *J* = 138.4 Hz), 117.9, 118.6, 120.8 (d, *J* = 4.0 Hz), 121.1 (d, *J* = 4.0 Hz), 124.4 (d, *J* = 5.1 Hz), 125.7 (d, *J* = 5.1 Hz), 125.8, 126.0, 127.2, 127.3, 130.2, 131.7 (d, *J* = 5.1 Hz), 135.9 (d, *J* = 8.1 Hz), 137.0, 138.0, 150.4 (d, *J* = 9.1 Hz), 150.7 (d, *J* = 9.1 Hz), 154.1, 164.8, 165.6. **^31^P{^1^H} NMR** (243 MHz, DMSO-*d_6_*) δ 19.8. **HRMS (ESI)** *m*/*z*: calcd for [M + H]^+^ C_37_H_42_Br_2_N_2_O_6_P 799.1142; found 799.1144. **Anal. Calcd** for C_37_H_41_Br_2_N_2_O_6_P: C, 55.51; H, 5.16; Br, 19.96; N, 3.50; P, 3.87. Found: C, 55.52; H, 5.14; Br, 19.98; N, 3.51; P, 3.89.

*Diphenyl[(2,4-bis(2-chloroacetamido)phenyl)(3,5-di-tert-butyl-4-hydroxyphenyl)methyl]phosphonate* (**9.6e**). White solid; 70%. M.p. 88–90 °C. IR (KBr, cm^−1^) ν 629, 766, 939, 1239, 1594, 1686, 3390, 3630. **^1^H NMR** (500 MHz, DMSO-*d_6_*) δ 1.31 (s, 18H), 4.25, 4.27, 4.30 (s, 4H), 5.28 (d, *J* = 27.0 Hz, 1H), 6.61 (d, *J* = 8.0 Hz, 2H), 6.96 (d, *J* = 7.7 Hz, 2H), 7.10 (t, *J* = 7.4 Hz, 1H), 7.20 (m, 3H), 7.30 (m, 4H), 7.53 (d, *J* = 10.0 Hz, 1H), 7.76 (s, 1H), 7.94 (d, *J* = 8.7 Hz, 1H), 9.92 (s, 1H), 10.43 (s, 1H). **^13^C{^1^H} NMR** (126 MHz, DMSO-*d_6_*) δ 30.6, 34.9, 43.2 (d, *J* = 140.3 Hz), 43.4, 43.9, 117.6, 118.1, 120.4 (d, *J* = 3.7 Hz), 120.9 (d, *J* = 3.8 Hz), 125.3, 125.6, 125.7 (d, *J* = 5.3 Hz), 127.1, 129.9, 130.0, 130.0, 136.2 (d, *J* = 12.6 Hz), 138.2, 139.6, 150.3 (d, *J* = 9.6 Hz), 150.7 (d, *J* = 9.4 Hz), 153.7, 165.2, 165.7. **^31^P{^1^H} NMR** (162 MHz, DMSO-*d_6_*) δ 19.8. **Anal. Calcd** for C_37_H_41_Cl_2_N_2_O_6_P: C, 62.45; H, 5.81; Cl, 9.96; N, 3.94; P, 4.35. Found: C, 62.23; H, 5.80; Cl, 9.93; N, 3.93; P, 4.34. **HRMS (ESI)** *m*/*z*: calcd for [M + H]^+^ C_37_H_42_Cl_2_N_2_O_6_P 711.2152; found 711.2155.

### 3.4. Docking Studies

For the docking, a tetramer of PKM2 (PDB code 3GR4), GAPDH (PDB code 6M61) and a dimer of GSTO1 (PDB code 4YQU) [28] were obtained from the Protein Data Bank. Receptors were prepared for covalent docking by first removing all solvent and ligand molecules (except for the NAD^+^ molecule for GAPDH; see below), adding hydrogen atoms and charges using ChimeraX 1.11.1 software [46], and processing them with the Meeko 0.6.1 python package [45]. For the covalent docking to the Cys317, Cys326, and Cys474 residues of PKM2, only subunit A of the tetramer was used based on the spatial positions of these residues. For the Cys31 and Cys424 residues, subunits A/B and A/C were considered, respectively, due to their proximity to these residues. For the GAPDH (Cys152 residue), only the O subunit of the tetramer was used for the docking. The docking was performed both with the NAD^+^ removed from the binding site and with NAD^+^ retained in its native position. For the docking to the Cys32 residue of GSTO1, only subunit A was used.

The following box dimensions (points_x points_y points_z, grid spacing (in Å)) and coordinates of center (x, y, z (in Å)) were used in docking: PKM2, residue Cys317 (box: 74 74 70, 0.375; center: −7.304 4.252 16.082); PKM2, residue Cys326 (box: 72 74 72, 0.375; center: −5.281 9.462 5.545); PKM2, residue Cys474 (box: 72 74 70, 0.375; center: −22.239 −16.233 1.186); PKM2, residue Cys31 (box: 72 70 76, 0.375; center: 18.994 −0.338 4.908); PKM2, residue Cys424 (box: 74 70 76, 0.375; center: 4.394 −16.740 5.446); GAPDH, residue Cys152 (box: 94 94 96, 0.375; center: 48.185 27.445 16.743); GSTO1, residue Cys32 (box: 88 90 82, 0.375; center: 19.102 −9.945 0.776).

Ligands for covalent docking were prepared by first converting their SMILES representations to 3D coordinates (XYZ format) using the Open Babel 3.1.1 program [60], followed by geometry optimization with the semiempirical GFN2-xTB method [42] as implemented in ORCA 6.0.0 [43,44]. The optimized structures were further prepared for docking using the Meeko 0.6.1 python package [45]. For compounds **9.1–9.6**, which possess a stereocenter, both (*R*)- and (*S*)-enantiomers were modeled to assess whether the stereochemical configuration substantially affected the predicted binding affinity.

Covalent docking was performed using AutoDock-GPU 1.6 [47] with the default settings and the number of runs set to 100 (--nruns 100). The affinity grid maps required by AutoDock-GPU were generated using AutoGrid 4.2.7 utility [61]. The generated poses were clustered using the built-in AutoDock-GPU 1.6 clustering tool based on the RMSD (2.0 Å cutoff by default) to identify the most populated clusters and their lowest-energy conformations. All docking runs were carried out in triplicate to confirm the reproducibility of the predicted binding poses and energies; the mean docking score from the three runs was used in subsequent analysis. PLIP v2.4.0 [48] was used for protein-ligand interaction analysis.

### 3.5. Biological Studies

#### 3.5.1. Evaluation of the Cytotoxicity of Compounds

The cytotoxic effect on cells was determined using the colorimetric method of cell proliferation-the MTT test. NADP-H-dependent cellular oxidoreductase enzymes can, under certain conditions, reflect the number of viable cells. These enzymes are able to reduce the tetrazolium dye (MTT)–3-(4,5-dimethylthiazol-2-yl)-2,5-diphenyl-tetrazolium bromide to insoluble blue-violet formazan, which crystallizes inside the cell. The amount of formazan formed is proportional to the number of cells with active metabolism. Cells were seeded on a 96-well Nunc plate at a concentration of 5 × 10^3^ cells per well in a volume of 100 μL of medium and cultured in a CO_2_ incubator at 37 °C until a monolayer was formed. Then the nutrient medium was removed, and 100 µL of solutions of the test drug in the given dilutions were added to the wells, which were prepared directly in the nutrient medium with the addition of 5% DMSO to improve solubility. In the first well of a 96-well plate, the DMSO concentration was 1.25%. The plate also contained control wells with the same solvent content (1.25%), in which cell viability was ≥98%. After 48 h of incubation of the cells with the tested compounds, the nutrient medium was removed from the plates and 100 µL of the nutrient medium without serum with MTT at a concentration of 0.5 mg/mL was added and incubated for 4 h at 37 °C. Formazan crystals were added 100 µL of DMSO to each well. Optical density was recorded at 540 nm on an Invitrologic microplate reader (Medico-Biological Union, Novosibirsk, Russia). The experiments for all compounds were repeated three times. The compounds were tested in the concentration range of 1–100 µM. The IC_50_ values were calculated using the online calculator Quest Graph™ IC_50_ Calculator (AAT Bioquest, Inc., Pleasanton, CA, USA; Version 2026, https://www.aatbio.com/tools/ic50-calculator, accessed on 21 September 2026).

#### 3.5.2. Determination of Lactate Dehydrogenase Activity

Lactate dehydrogenase (LDH) activity in PANC-1 cells was determined using the commercial Lactate Dehydrogenase (LDH) Activity Assay Kit (E-BC-K046-S, Elabscience, Wuhan, China) according to the manufacturer’s protocol. The method is based on the colorimetric detection of pyruvate formed in the LDH-catalyzed reaction, yielding a colored product whose intensity is proportional to the enzyme activity.

The optical density of the samples was recorded at 450 nm using an AIFR-01 Uniplan plate spectrophotometer, Pikon (Moscow, Russia). Each experiment was performed in triplicate.

#### 3.5.3. Determination of Pyruvate Dehydrogenase Activity

Pyruvate dehydrogenase (PDH) activity was determined in PANC-1 cell lysates using the PDH Activity Assay Kit (E-BC-K650-M, ElabScience, Wuhan, China). The principle of the method is based on recording the rate of reduction of 2,6-dichlorophenolindophenol (DCPIP), which is coupled to the PDH-catalyzed oxidative decarboxylation of pyruvate. During the reaction, pyruvate is converted to acetyl-coenzyme A, and the reducing equivalents generated through oxidation reduce DCPIP via a system of intermediate carriers.

The optical density of the samples was recorded at 605 nm using an AIFR-01 Uniplan plate spectrophotometer, Pikon (Moscow, Russia). Each experiment was performed in triplicate.

## 4. Conclusions

In conclusion, we successfully combined ligand-based SAR with structure-based covalent docking to develop a target-hypothesis-guided strategy for exploring haloacetamide-derived di(hetero)arylmethanes. GAPDH and GSTO1 were treated as candidate molecular targets based on prior biological and chemical evidence, rather than as experimentally established targets. Covalent docking identified binding-site features and compound-protein interaction patterns that were associated with cytotoxic potency and were subsequently used as structural constraints for scaffold design. Importantly, these calculations were not intended to establish target identity; their purpose was to determine whether candidate target hypotheses could provide actionable information for prospective molecular design. The subsequent synthesis and biological evaluation of the designed phosphorylated diarylmethanes with haloacetamide warheads provided a prospective test of this strategy and resulted in highly potent bromoacetamide derivatives (IC_50_ = 0.9 µM against M-HeLa and 4.5 µM against HuTu-80), rivaling the reference drug doxorubicin. The present data do not establish GAPDH or GSTO1 as the exclusive or primary cellular targets of the compounds, and alternative protein targets cannot be excluded. Nevertheless, these findings demonstrate that covalent target hypotheses can serve as practical design constraints for the discovery of novel scaffolds even when definitive molecular target identification is not yet available, providing a complementary strategy for covalent drug-discovery programs operating under incomplete target information.

## Figures and Tables

**Table 1:** Influence of amide and di(hetero)aryl moieties on the cytotoxicity (IC_50_, µM) of di(hetero)arylmethane derivatives ^a^.

Cmpd	IC_50_ (µM)					
	Cancer Cell Lines				Normal Cell Lines	
	M-HeLa	SI	HuTu-80	SI	WI38	Chang Liver
**1b**	56.2 ± 4.7	2.47	1.9 ± 0.2	73.16	–	139.0 ± 11
**3a**	79.8 ± 9.7	1.39	76.6 ± 17	1.45	117.4 ± 7.6	111.3 ± 4.6
**3b**	77.6 ± 7.4	1.59	106.7 ± 20	1.16	126.4 ± 3.1	123.4 ± 8.5
**3c**	74.1 ± 2.0	1.58	71.8 ± 17	1.63	116.7 ± 16	117.3 ± 7.6
**3d**	91.6 ± 8.8	1.27	82.2 ± 15	1.41	134.7 ± 23	115.9 ± 12
**3e**	88.7 ± 7.5	>1.13 ^c^	>100	–	>100	–
**3f**	79.4 ± 10	1.52	84.6 ± 11	1.43	121.3 ± 5.1	120.9 ± 3.7
**4a**	20.4 ± 1.7	2.07 ^c^	9.8 ± 3.0	4.31 ^c^	42.2 ± 0.3	–
**4b**	11.0 ± 3.6	1.78 ^c^	0.7 ± 0.1	28.00 ^c^	19.6 ± 5.2	–
**4c**	9.6 ± 2.3	2.03 ^c^	0.9 ± 0.2	21.67^ c^	19.5 ± 5.9	–
**4d** ^b^	65.3 ± 5.4	1.04	52.1 ± 4.2	1.31	–	68.1 ± 5.4
**4e** ^b^	84.6 ± 7.5	>1.18	26.2 ± 1.7	>3.82	–	>100
**4f** ^b^	49.6 ± 3.9	>2.02	29.3 ± 2.1	>3.41	–	>100
**4g** ^b^	38.4 ± 2.7	1.43	18.1 ± 1.4	3.03	–	54.8 ± 4.4
**5a**	58.3 ± 5.9	1.14	60.9 ± 6.3	1.09	49.9 ± 2.9	66.2 ± 6.8
**5b**	67.3 ± 4.6	1.43	30.6 ± 4.9	3.14	-	96.0 ± 2.6
**DOX**	2.1 ± 0.1	1	3.0 ± 0.01	0.7	3.5 ± 0.1	2.1 ± 0.1

^a^ three independent experiments were carried out; “–“ means not tested, SI = IC_50_(Chang liver)/IC_50_(cancer cells), the hit compound is highlighted in green, the most active compounds are highlighted in yellow; ^b^ the data was taken from paper [11]; ^c^ SI calculated using WI38 cell line cytotoxicity.

**Table 2:** Cytotoxicity (IC_50_, µM) of synthesized compounds **9.5c–f** and **9.6c,e** against cancer and normal cell lines ^a^.

Cmpd	X	R	IC_50_ (µM)					
			Cancer Cell Lines				Normal Cell Lines	
			M-HeLa	SI	HuTu-80	SI	WI38	Chang Liver
**9.5c**	Br	Et	1.9 ± 0.1	2.37	5.8 ± 0.5	0.78	2.3 ± 0.1	4.5 ± 0.4
**9.5d**	Br	Me	0.9 ± 0.08	5.78	4.5 ± 0.4	1.16	1.7 ± 0.1	5.2 ± 0.4
**9.5f**	Cl	Me	16.8 ± 1.3	2.37	-	–	-	39.8 ± 2.8
**9.6c**	Br	Ph	2.3 ± 0.2	1.22	4.3 ± 0.4	0.65	2.7 ± 0.2	2.8 ± 0.3
**9.6e**	Cl	Ph	31.8 ± 2.8	>3.14	-	–	-	>100
**1** ^b^	–	–	56.2 ± 4.7	2.47	1.9 ± 0.2	73.16	–	139.0 ± 11
**4b** ^b^			11.0 ± 3.6	1.78 ^c^	0.7 ± 0.1	28.00 ^c^	19.6 ± 5.2	–
**4c** ^b^			9.6 ± 2.3	2.03 ^c^	0.9 ± 0.2	21.67 ^c^	19.5 ± 5.9	–
**Doxorubicin**	–	–	2.1 ± 0.1	1.00	3.0 ± 0.01	0.70	3.5 ± 0.1	2.1 ± 0.1

^a^ three independent experiments were carried out; “–“ means not tested, SI = IC_50_(Chang liver)/IC_50_(cancer cells), the most active compounds is highlighted in green; ^b^ hit compounds, ^c^ SI calculated using WI38 cell line cytotoxicity.

## Data Availability

The original contributions presented in this study are included in the article/Supplementary Material. Further inquiries can be directed to the corresponding author.

## Supplementary Materials

The following supporting information can be downloaded at: https://www.mdpi.com/article/10.3390/ijms27219559/s1 [62,63,64,65].
