# Supplementary material for: Combining Ligand- and Structure-Based Approaches to Develop Haloacetamide-Derived Di(hetero)arylmethanes as Potential Anticancer Agents
Source: Int J Mol Sci. 2026 Oct 26;27(21):9559. doi: 10.3390/ijms27219559 (PMC13762480; doi:10.3390/ijms27219559)
Supplement: Supplementary file 1 [file ijms-27-09559-s001.zip › SI_HPLC_traces.pdf]

Compound Spectrum SmartFormula Report

Analysis Info

Analysis Name	D:\Data\IOPC\Babaev\2025\Smolobochkin\Tod-7_2_1_3184 — Blank_MeOH_1_1_3185.d	Acquisition Date	12/12/2025 12:43:50 PM
Method	LCMS_pos_6min_5-95B(2)_50_1300mz_new.m	Operator	Demo User
Sample Name	Tod-7	Instrument	impact II 1825265.00000
Comment	+1mkl in MeOH<br>Original analysis: 'Tod-7_2_1_3184.d',<br>Subtracted analysis: 'Blank_MeOH_1_1_3185.d' (Xpose, 0.5s, 5),<br>Processed with: 'DataAnalysis 5.3.556',<br>Date: 2025-12-12T13:19:00		

Acquisition Parameter

Source Type	ESI	Ion Polarity	Positive	Set Nebulizer	2.0 Bar
Focus	Not active	Set Capillary	4500 V	Set Dry Heater	220 °C
Scan Begin	50 m/z	Set End Plate Offset	-500 V	Set Dry Gas	6.0 l/min
Scan End	1900 m/z	Set Collision Cell RF	1500.0 Vpp	Set Divert Valve	Source

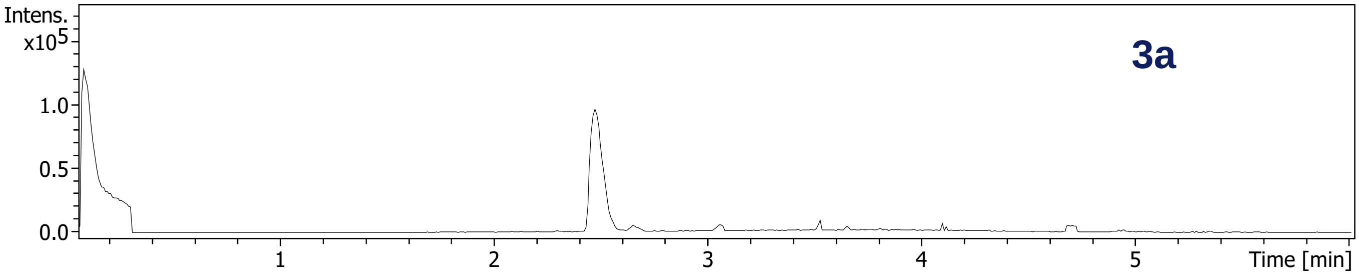

+MS, 2.5min, #288

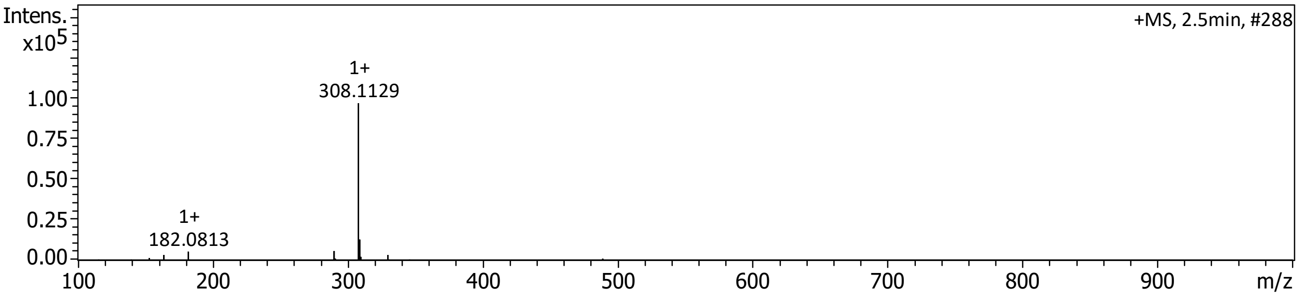

Display Report

Analysis Info

Analysis Name	D:\Data\IOPC\Babaev\2025\Smolobochkin\Tod-11_3_1_3187 — Blank_MeOH_1_1_3183.d	Acquisition Date	12/12/2025 1:42:40 PM
Method	LCMS_pos_6min_5-95B(2)_50_1300mz_new.m	Operator	Demo User
Sample Name	Tod-11	Instrument	impact II 1825265.00000
Comment	+5mkl in MeOH<br>Original analysis: 'Tod-11_3_1_3187.d',<br>Subtracted analysis: 'Blank_MeOH_1_1_3183.d' (Xpose, 0.5s, 5),<br>Processed with: 'DataAnalysis 5.3.556',<br>Date: 2025-12-12T14:20:31		

Acquisition Parameter

Source Type	ESI	Ion Polarity	Positive	Set Nebulizer	2.0 Bar
Focus	Not active	Set Capillary	4500 V	Set Dry Heater	220 °C
Scan Begin	50 m/z	Set End Plate Offset	-500 V	Set Dry Gas	6.0 l/min
Scan End	1900 m/z	Set Collision Cell RF	1500.0 Vpp	Set Divert Valve	Source

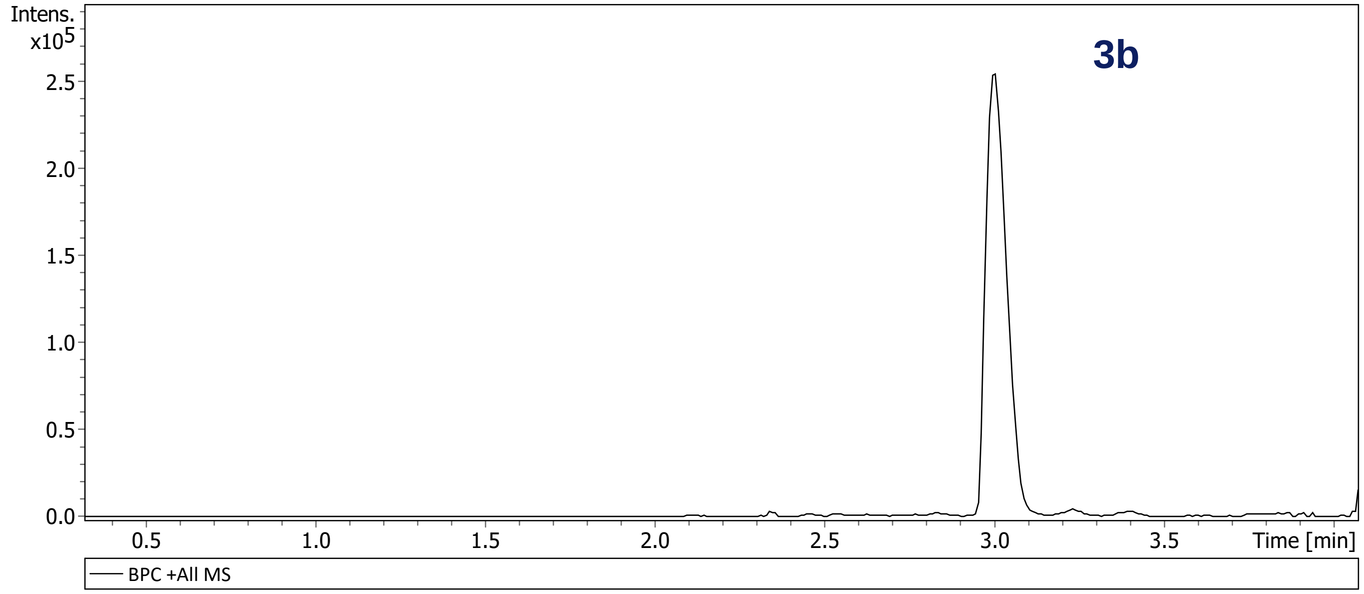

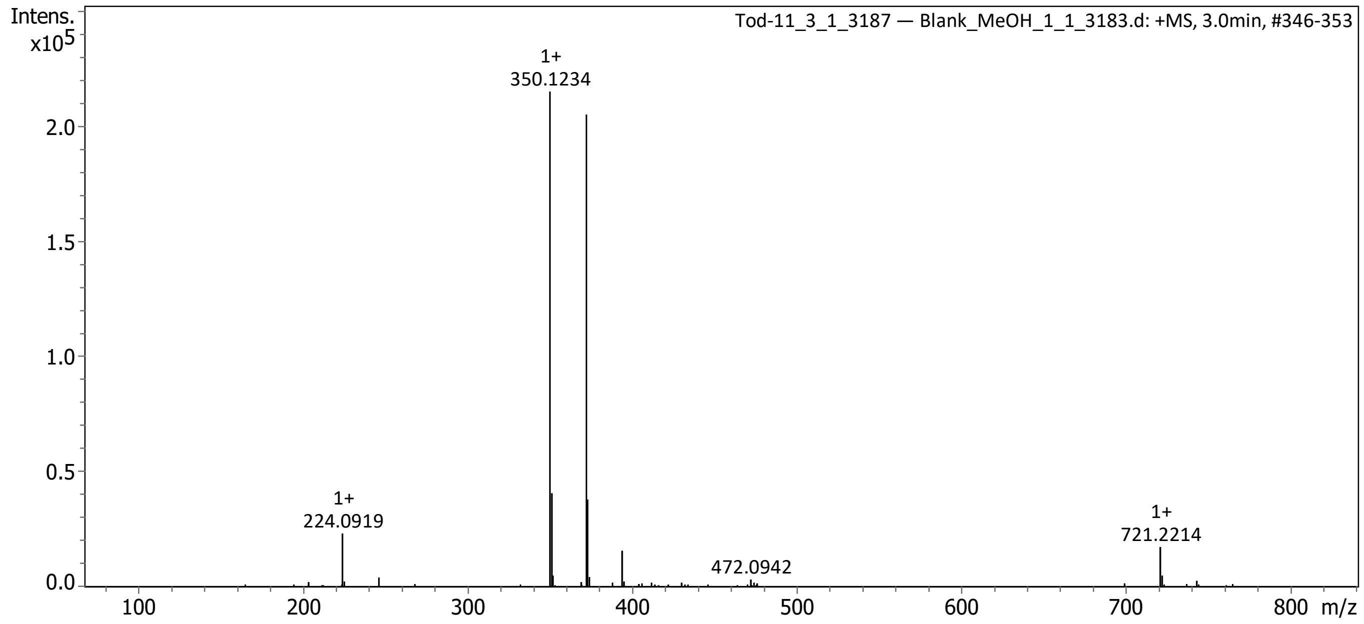

Display Report

Analysis Info

		Acquisition Date	12/12/2025 2:07:47 PM
Analysis Name	D:\Data\IOPC\Babaev\2025\Smolobochkin\Tod-17_5_1_3191 — Blank_MeOH_1_1_3190.d		
Method	LCMS_pos_6min_5-95B(2)_50_1300mz_new.m	Operator	Demo User
Sample Name	Tod-17	Instrument	impact II 1825265.00000
Comment	+5mkl in MeOH<br>Original analysis: 'Tod-17_5_1_3191.d',<br>Subtracted analysis: 'Blank_MeOH_1_1_3190.d' (Xpose, 0.5s, 5),<br>Processed with: 'DataAnalysis 5.3.556',<br>Date: 2025-12-12T14:42:46		

Acquisition Parameter

Source Type	ESI	Ion Polarity	Positive	Set Nebulizer	2.0 Bar
Focus	Not active	Set Capillary	4500 V	Set Dry Heater	220 °C
Scan Begin	50 m/z	Set End Plate Offset	-500 V	Set Dry Gas	6.0 l/min
Scan End	1900 m/z	Set Collision Cell RF	1500.0 Vpp	Set Divert Valve	Source

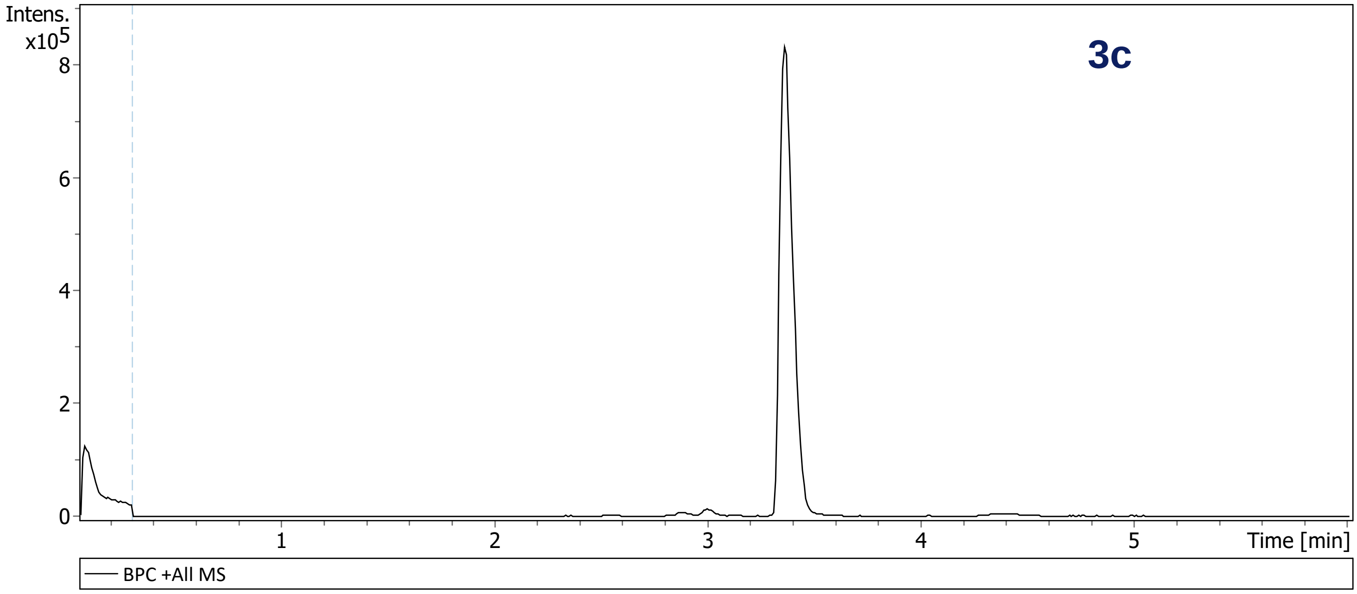

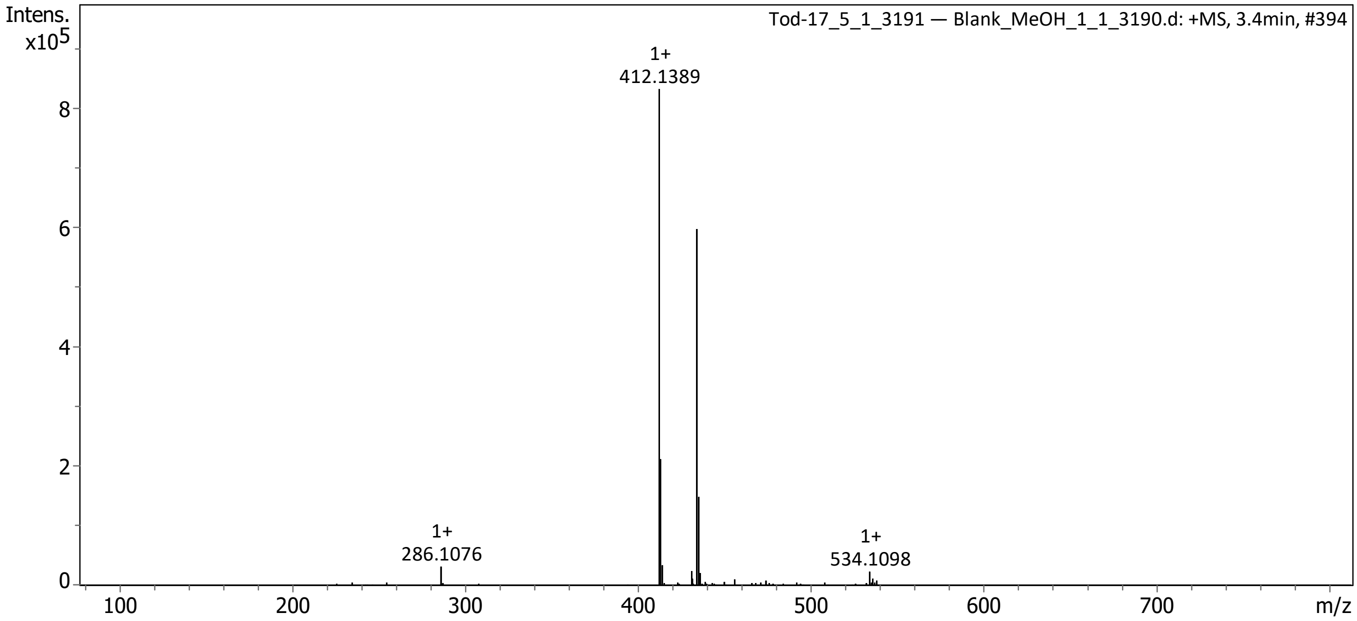

Compound Spectrum SmartFormula Report

Analysis Info

Analysis Name	D:\Data\IOPC\Babaev\2026\Burilov\Tod-16_12_1_3882 — blank_H2O-ACN_1_1_3880.d	Acquisition Date	10/14/2026 3:26:49 PM	
Method	LCMS_pos_8min_5-95B1_50_1300mz.m	Operator	Demo User	
Sample Name	Tod-16	Instrument	impact II	1825265.00000
Comment	Original analysis: 'Tod-16_12_1_3882.d',<br>Subtracted analysis: 'blank_H2O-ACN_1_1_3880.d' (Xpose, 0.5s, 5),<br>Processed with: 'DataAnalysis 5.3.556',<br>Date: 2026-10-14T16:05:32			

Acquisition Parameter

Source Type	ESI	Ion Polarity	Positive	Set Nebulizer	2.0 Bar
Focus	Not active	Set Capillary	4500 V	Set Dry Heater	220 °C
Scan Begin	50 m/z	Set End Plate Offset	-500 V	Set Dry Gas	6.0 l/min
Scan End	1900 m/z	Set Collision Cell RF	1500.0 Vpp	Set Divert Valve	Source

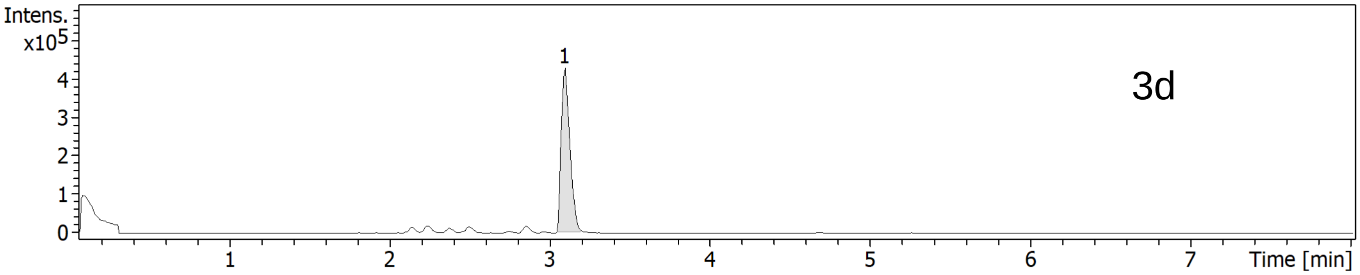

#	RT [min]	Int. Type	Trace	Area	Range [min]	S/N	I
1	3.1	Manual	BPC +All MS	1579115	3.0 - 3.2	2804.6	427785

Cmpd 1, 3.1 min

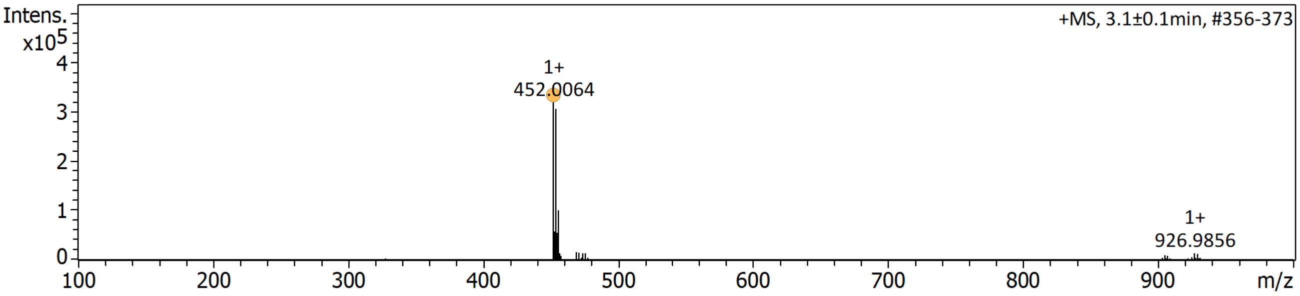

Meas. m/z	#	Ion Formula	m/z	err [ppm]	Mean err [ppm]	rdb	N-Rule	e⁻ Conf	mSigma	Std I	Std Mean m/z	Std I VarNorm	Std m/z Diff	Std Comb Dev
452.006363	1	C17H17Cl3NO7	452.006511	0.3	0.6	18.0	ok	even	17.5	16.0	n.a.	n.a.	n.a.	n.a.

Display Report

Analysis Info

Analysis Name	D:\Data\IOPC\Babaev\2025\Smolobochkin\Tod-132_13_1_3207 — Blank_MeOH_1_1_3206.d	Acquisition Date	12/12/2025 3:48:21 PM
Method	LCMS_pos_6min_5-95B(2)_50_1300mz_new.m	Operator	Demo User
Sample Name	Tod-132	Instrument	impact II 1825265.00000
Comment	+5mkl in MeOH<br>Original analysis: 'Tod-132_13_1_3207.d',<br>Subtracted analysis: 'Blank_MeOH_1_1_3206.d' (Xpose, 0.5s, 5),<br>Processed with: 'DataAnalysis 5.3.556',<br>Date: 2025-12-12T16:13:31		

Acquisition Parameter

Source Type	ESI	Ion Polarity	Positive	Set Nebulizer	2.0 Bar
Focus	Not active	Set Capillary	4500 V	Set Dry Heater	220 °C
Scan Begin	50 m/z	Set End Plate Offset	-500 V	Set Dry Gas	6.0 l/min
Scan End	1900 m/z	Set Collision Cell RF	1500.0 Vpp	Set Divert Valve	Source

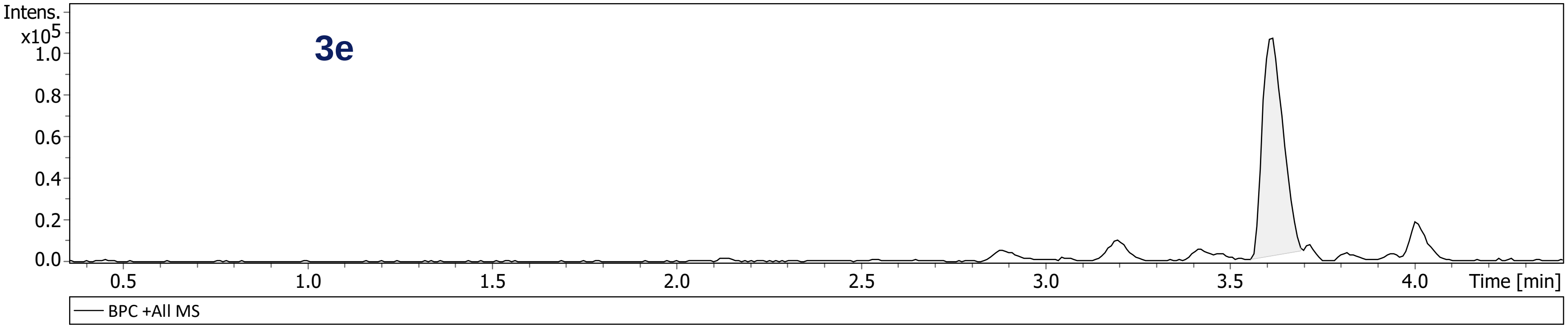

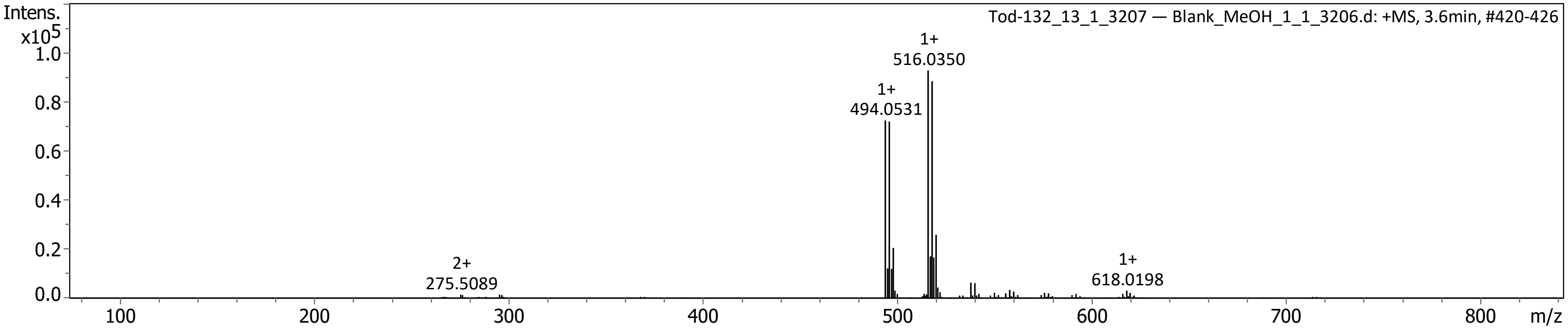

Display Report

Analysis Info

Analysis Name	D:\Data\IOPC\Babaev\2025\Smolobochkin\Tod-29_6_1_3193 — Blank_MeOH_1_1_3192.d	Acquisition Date	12/12/2025 2:20:20 PM
Method	LCMS_pos_6min_5-95B(2)_50_1300mz_new.m	Operator	Demo User
Sample Name	Tod-29	Instrument	impact II 1825265.00000
Comment	+5mkl in MeOH<br>Original analysis: 'Tod-29_6_1_3193.d',<br>Subtracted analysis: 'Blank_MeOH_1_1_3192.d' (Xpose, 0.5s, 5),<br>Processed with: 'DataAnalysis 5.3.556',<br>Date: 2025-12-12T15:14:46		

Acquisition Parameter

Source Type	ESI	Ion Polarity	Positive	Set Nebulizer	2.0 Bar
Focus	Not active	Set Capillary	4500 V	Set Dry Heater	220 °C
Scan Begin	50 m/z	Set End Plate Offset	-500 V	Set Dry Gas	6.0 l/min
Scan End	1900 m/z	Set Collision Cell RF	1500.0 Vpp	Set Divert Valve	Source

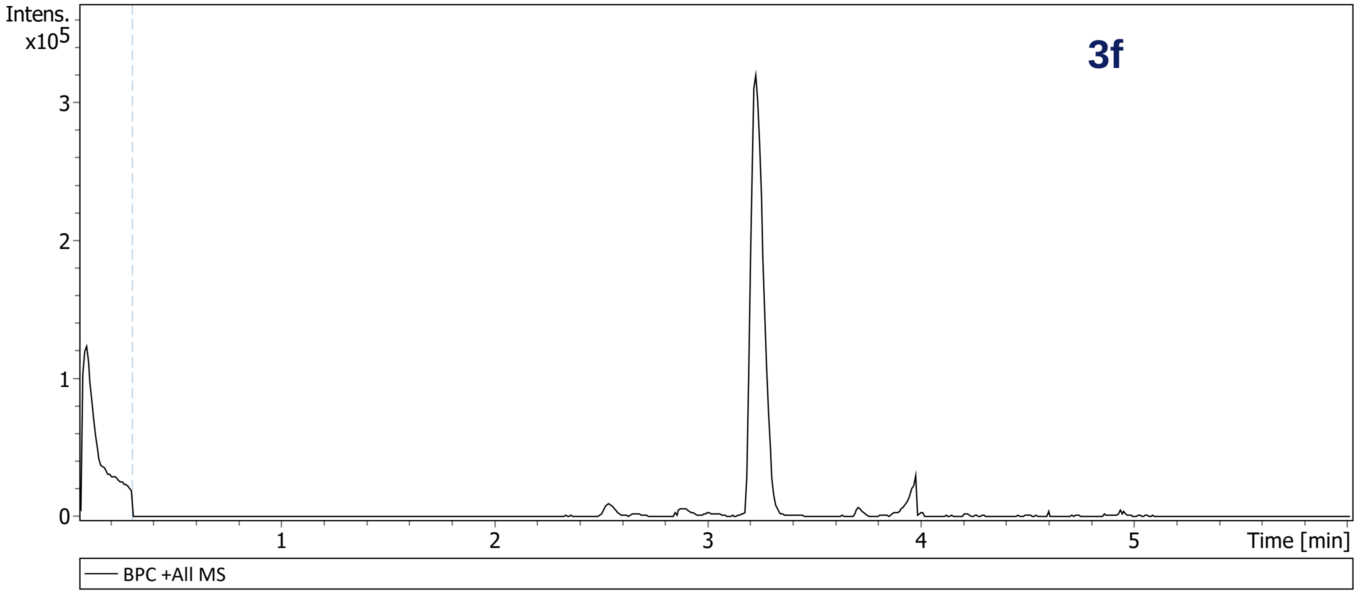

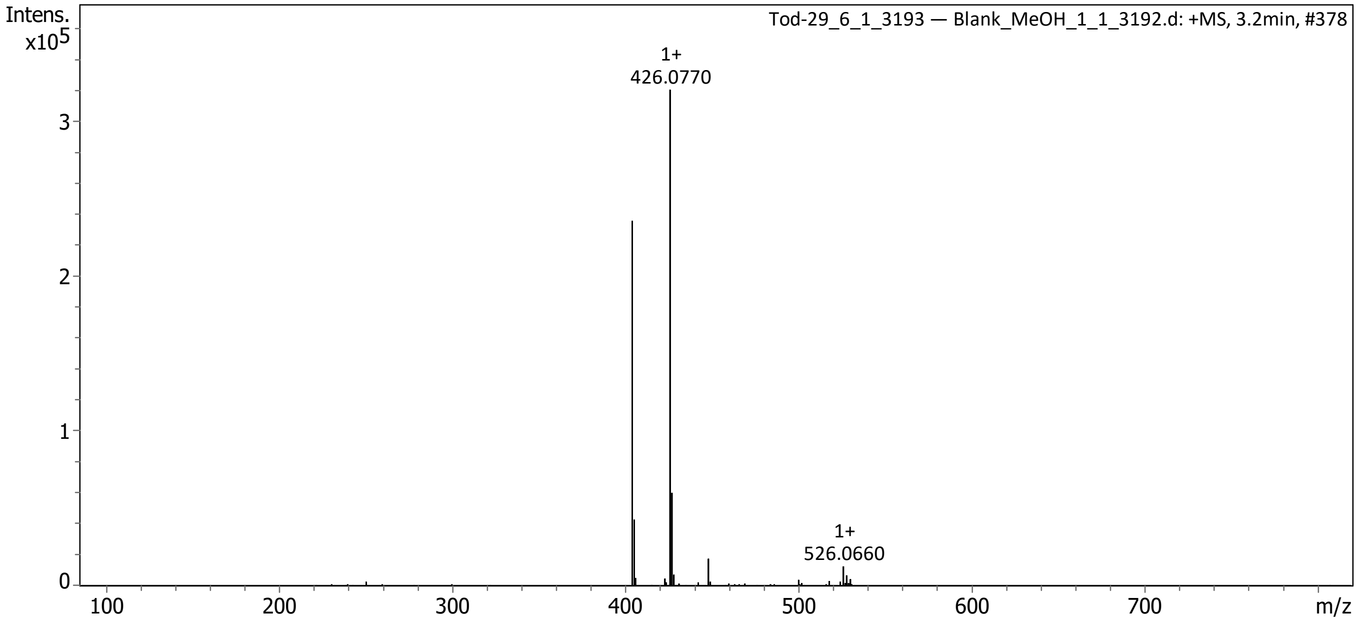

Compound Spectrum SmartFormula Report

Analysis Info

Analysis Name	D:\Data\IOPC\Babaev\2026\Burilov\Tod-97_13_1_3883 — blank_H2O-ACN_1_1_3880.d	Acquisition Date	10/14/2026 3:35:05 PM
Method	LCMS_pos_8min_5-95B1_50_1300mz.m	Operator	Demo User
Sample Name	Tod-97	Instrument	impact II 1825265.00000
Comment	Original analysis: 'Tod-97_13_1_3883.d',<br>Subtracted analysis: 'blank_H2O-ACN_1_1_3880.d' (Xpose, 0.5s, 5),<br>Processed with: 'DataAnalysis 5.3.556',<br>Date: 2026-10-14T16:06:39		

Acquisition Parameter

Source Type	ESI	Ion Polarity	Positive	Set Nebulizer	2.0 Bar
Focus	Not active	Set Capillary	4500 V	Set Dry Heater	220 °C
Scan Begin	50 m/z	Set End Plate Offset	-500 V	Set Dry Gas	6.0 l/min
Scan End	1900 m/z	Set Collision Cell RF	1500.0 Vpp	Set Divert Valve	Source

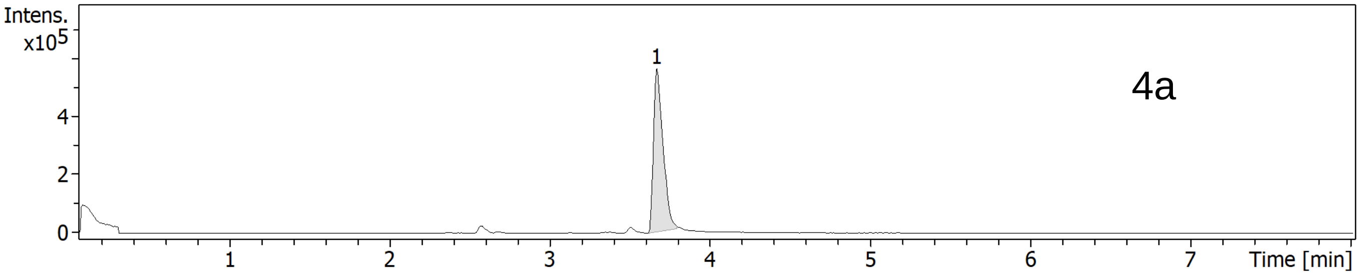

#	RT [min]	Int. Type	Trace	Area	Range [min]	S/N	I
1	3.7	Manual	BPC +All MS	2321723	3.6 - 3.8	2889.5	562877

Cmpd 1, 3.7 min

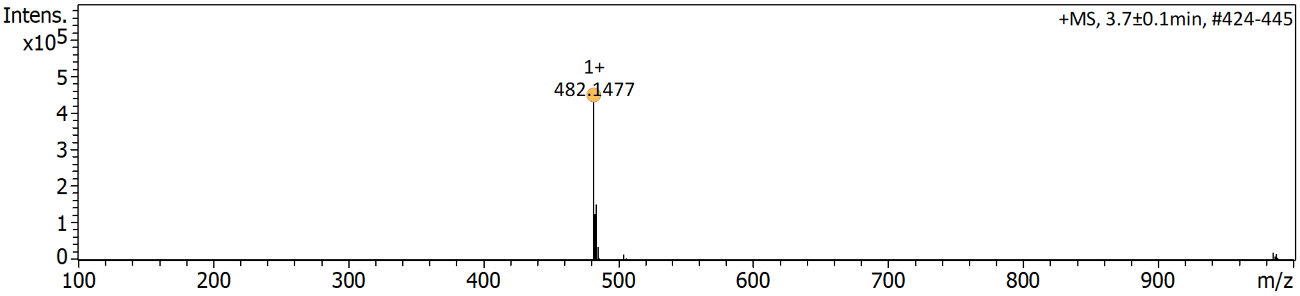

Meas. m/z	#	Ion Formula	m/z	err [ppm]	Mean err [ppm]	rdb	N-Rule	e⁻ Conf	mSigma	Std I	Std Mean m/z	Std I VarNorm	Std m/z Diff	Std Comb Dev
482.147691	1	C25H25ClN3O5	482.147725	0.1	0.3	18.0	ok	even	10.3	15.0	n.a.	n.a.	n.a.	n.a.

Display Report

Analysis Info

		Acquisition Date	12/12/2025 2:58:02 PM
Analysis Name	D:\Data\IOPC\Babaev\2025\Smolobochkin\Tod-91_9_1_3199 — Blank_MeOH_1_1_3198.d		
Method	LCMS_pos_6min_5-95B(2)_50_1300mz_new.m	Operator	Demo User
Sample Name	Tod-91	Instrument	impact II 1825265.00000
Comment	+5mkl in MeOH<br>Original analysis: 'Tod-91_9_1_3199.d',<br>Subtracted analysis: 'Blank_MeOH_1_1_3198.d' (Xpose, 0.5s, 5),<br>Processed with: 'DataAnalysis 5.3.556',<br>Date: 2025-12-12T15:41:41		

Acquisition Parameter

Source Type	ESI	Ion Polarity	Positive	Set Nebulizer	2.0 Bar
Focus	Not active	Set Capillary	4500 V	Set Dry Heater	220 °C
Scan Begin	50 m/z	Set End Plate Offset	-500 V	Set Dry Gas	6.0 l/min
Scan End	1900 m/z	Set Collision Cell RF	1500.0 Vpp	Set Divert Valve	Source

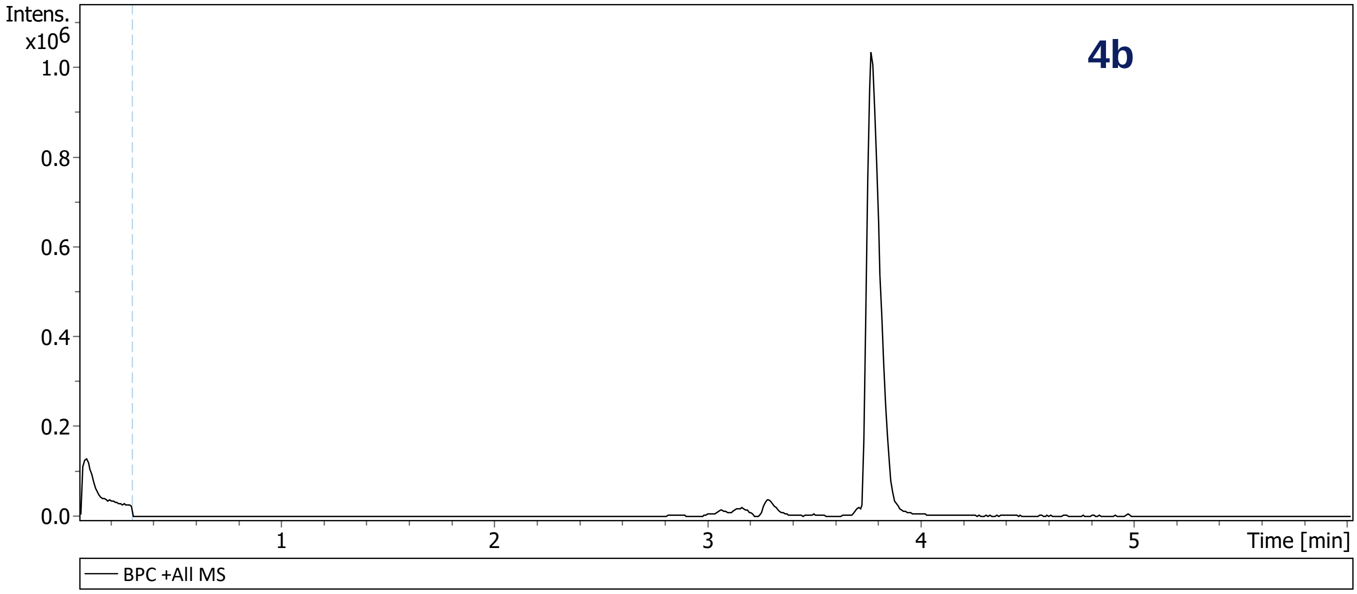

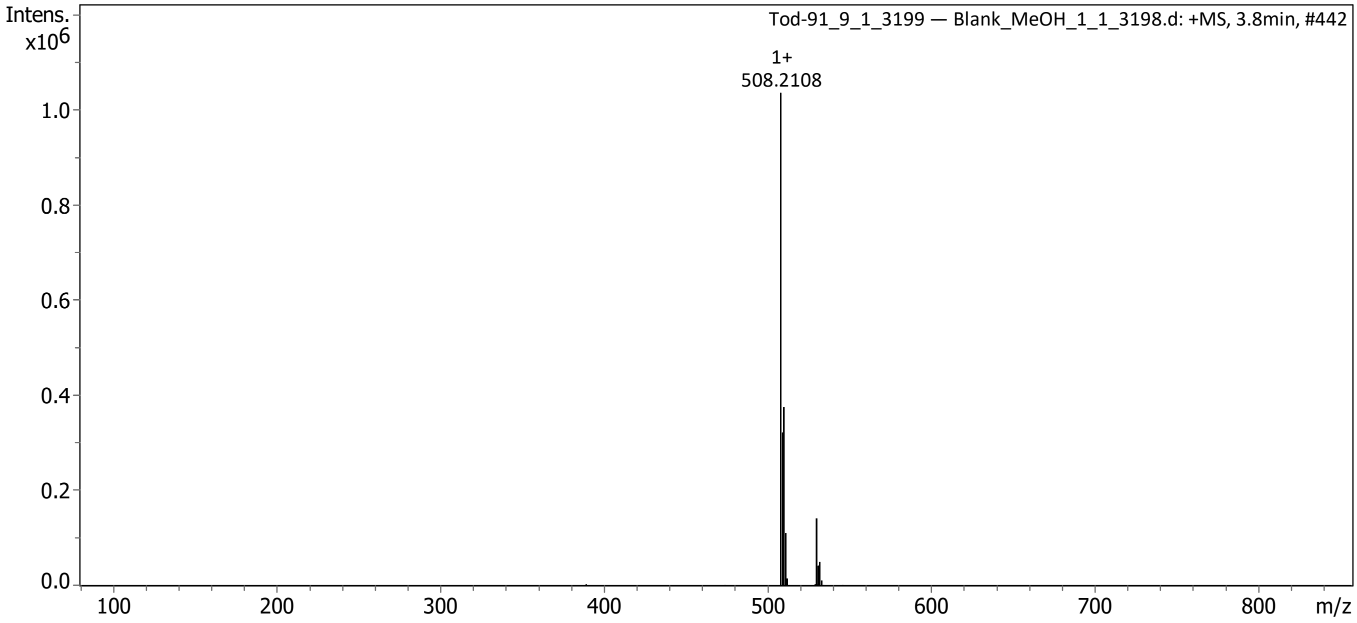

Display Report

Analysis Info

Analysis Name	D:\Data\IOPC\Babaev\2025\Smolobochkin\Tod-70_8_1_3197 — Blank_MeOH_1_1_3196.d	Acquisition Date	12/12/2025 2:45:28 PM
Method	LCMS_pos_6min_5-95B(2)_50_1300mz_new.m	Operator	Demo User
Sample Name	Tod-70	Instrument	impact II 1825265.00000
Comment	+5mkl in MeOH<br>Original analysis: 'Tod-70_8_1_3197.d',<br>Subtracted analysis: 'Blank_MeOH_1_1_3196.d' (Xpose, 0.5s, 5),<br>Processed with: 'DataAnalysis 5.3.556',<br>Date: 2025-12-12T15:35:21		

Acquisition Parameter

Source Type	ESI	Ion Polarity	Positive	Set Nebulizer	2.0 Bar
Focus	Not active	Set Capillary	4500 V	Set Dry Heater	220 °C
Scan Begin	50 m/z	Set End Plate Offset	-500 V	Set Dry Gas	6.0 l/min
Scan End	1900 m/z	Set Collision Cell RF	1500.0 Vpp	Set Divert Valve	Source

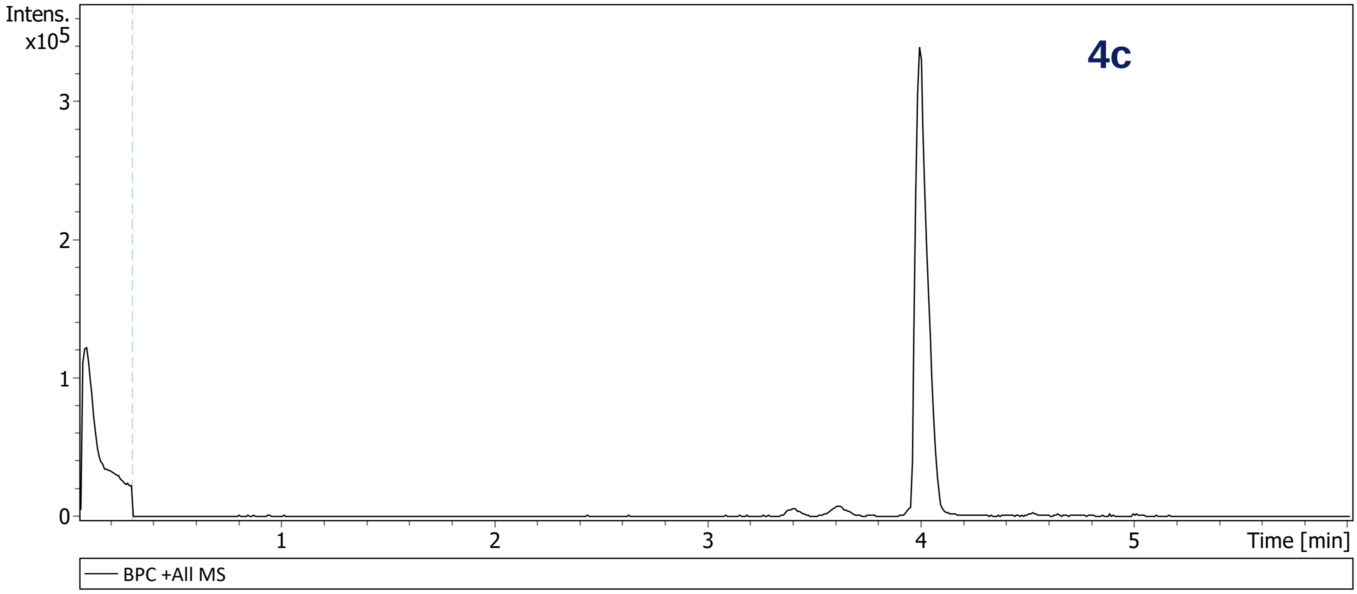

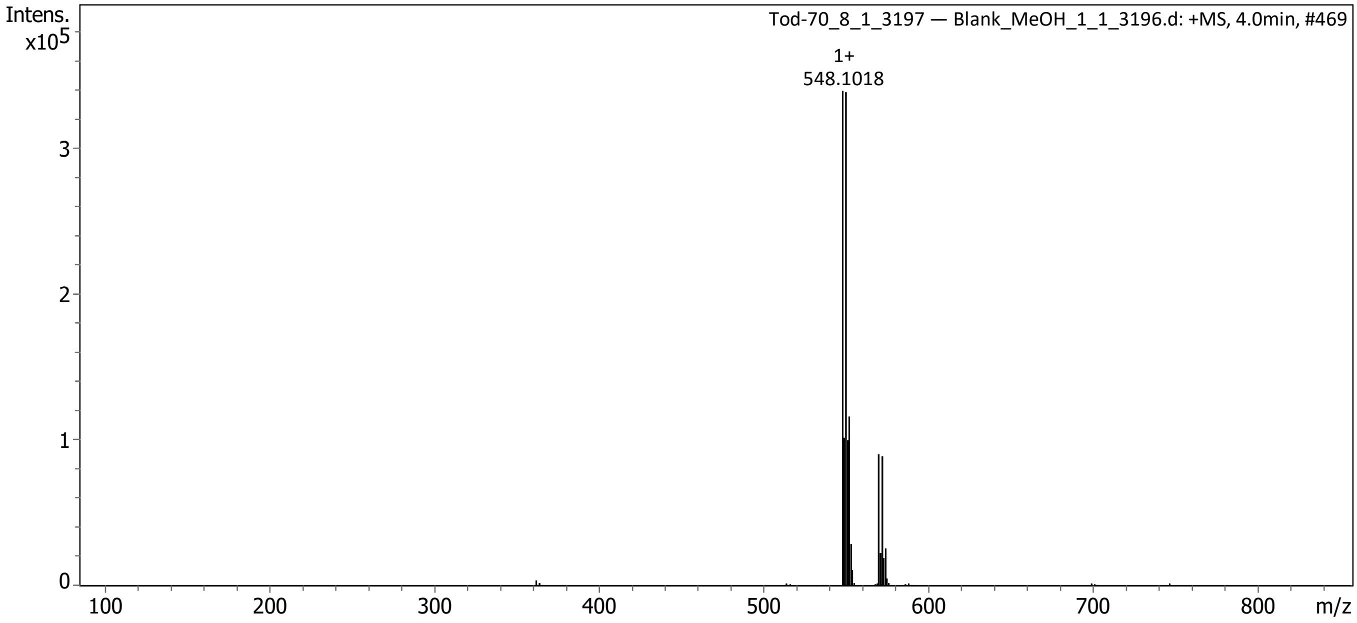

Display Report

Analysis Info

Analysis Name	D:\Data\IOPC\Babaev\2025\Smolobochkin\Tod-48_7_1_3195 — Blank_MeOH_1_1_3194.d	Acquisition Date	12/12/2025 2:32:54 PM
Method	LCMS_pos_6min_5-95B(2)_50_1300mz_new.m	Operator	Demo User
Sample Name	Tod-48	Instrument	impact II 1825265.00000
Comment	+5mkl in MeOH<br>Original analysis: 'Tod-48_7_1_3195.d',<br>Subtracted analysis: 'Blank_MeOH_1_1_3194.d' (Xpose, 0.5s, 5),<br>Processed with: 'DataAnalysis 5.3.556',<br>Date: 2025-12-12T15:30:13		

Acquisition Parameter

Source Type	ESI	Ion Polarity	Positive	Set Nebulizer	2.0 Bar
Focus	Not active	Set Capillary	4500 V	Set Dry Heater	220 °C
Scan Begin	50 m/z	Set End Plate Offset	-500 V	Set Dry Gas	6.0 l/min
Scan End	1900 m/z	Set Collision Cell RF	1500.0 Vpp	Set Divert Valve	Source

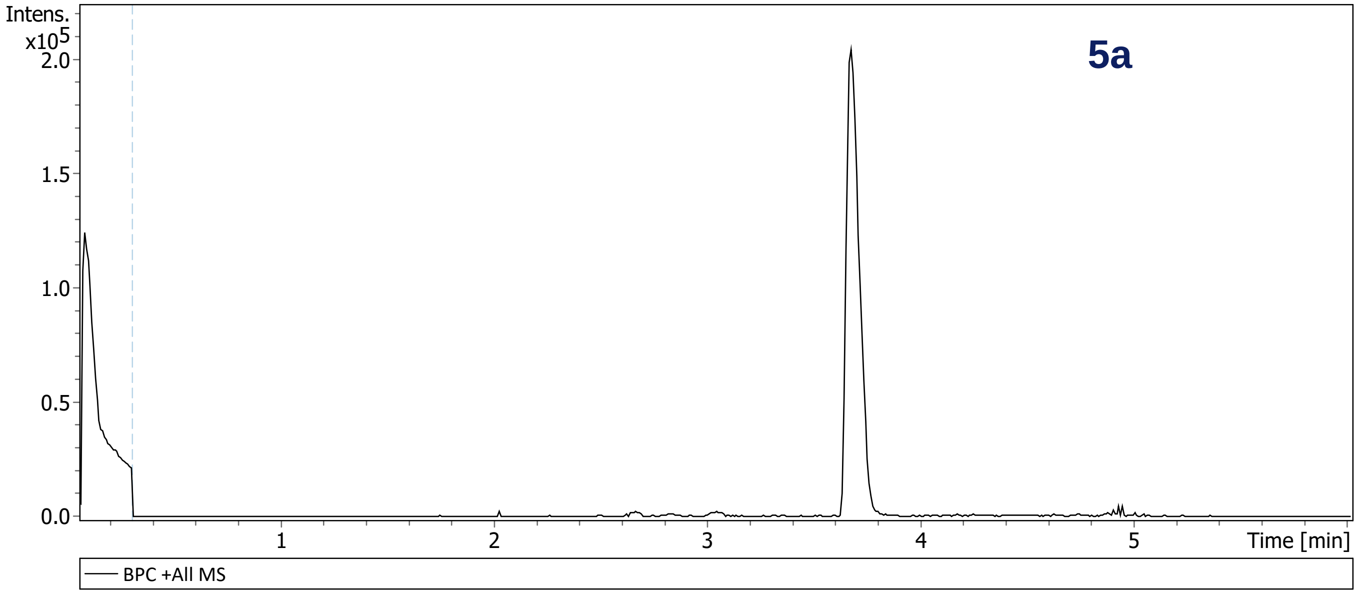

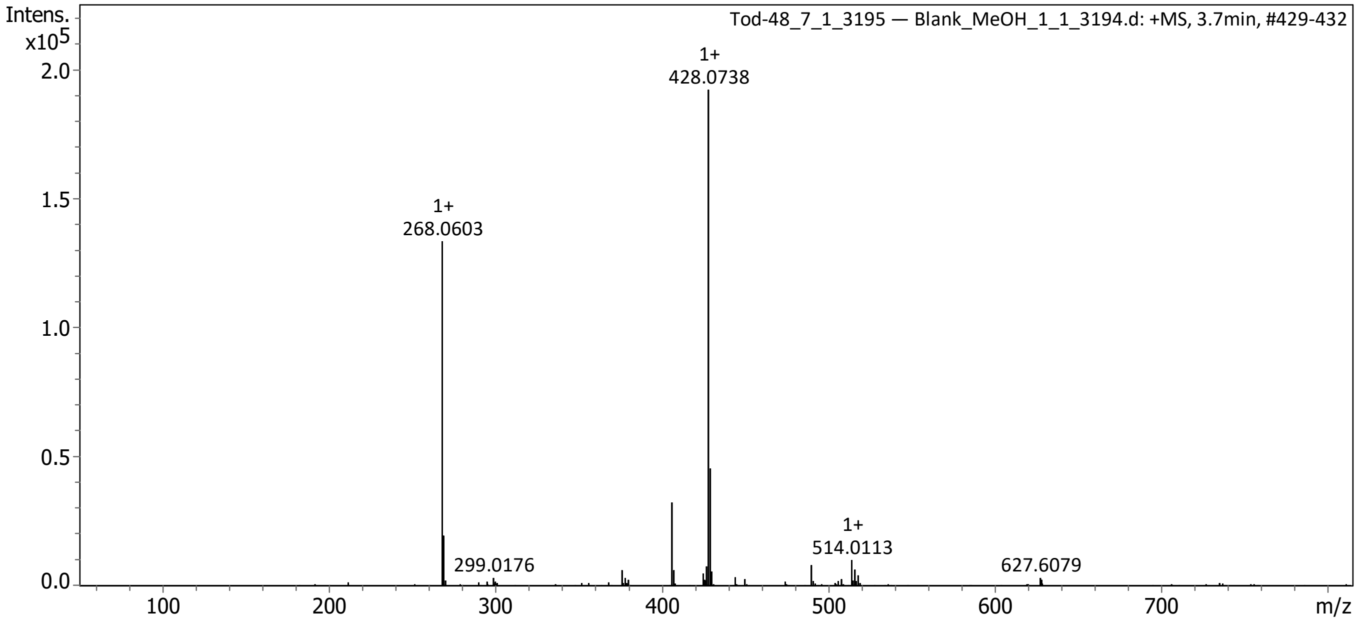

Display Report

Analysis Info

Analysis Name	D:\Data\IOPC\Babaev\2025\Smolobochkin\Tod-106_11_1_3203 — Blank_MeOH_1_1_3202.d	Acquisition Date	12/12/2025 3:23:11 PM
Method	LCMS_pos_6min_5-95B(2)_50_1300mz_new.m	Operator	Demo User
Sample Name	Tod-106	Instrument	impact II 1825265.00000
Comment	+5mkl in MeOH<br>Original analysis: 'Tod-106_11_1_3203.d',<br>Subtracted analysis: 'Blank_MeOH_1_1_3202.d' (Xpose, 0.5s, 5),<br>Processed with: 'DataAnalysis 5.3.556',<br>Date: 2025-12-12T15:48:09		

Acquisition Parameter

Source Type	ESI	Ion Polarity	Positive	Set Nebulizer	2.0 Bar
Focus	Not active	Set Capillary	4500 V	Set Dry Heater	220 °C
Scan Begin	50 m/z	Set End Plate Offset	-500 V	Set Dry Gas	6.0 l/min
Scan End	1900 m/z	Set Collision Cell RF	1500.0 Vpp	Set Divert Valve	Source

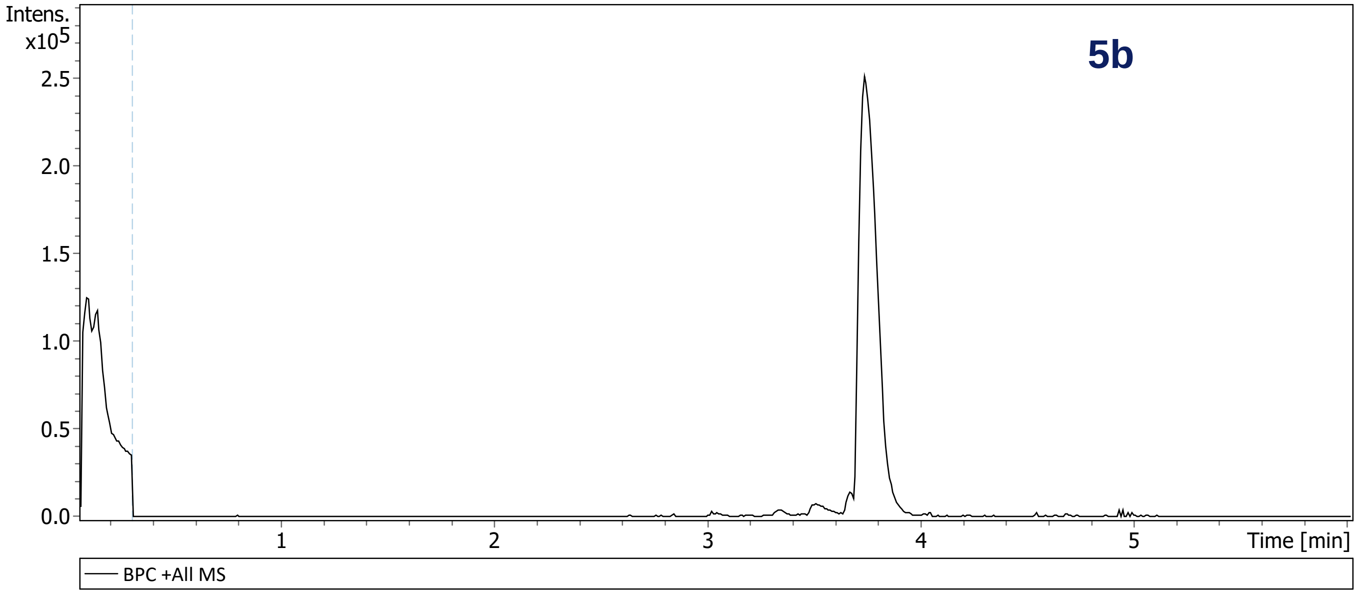

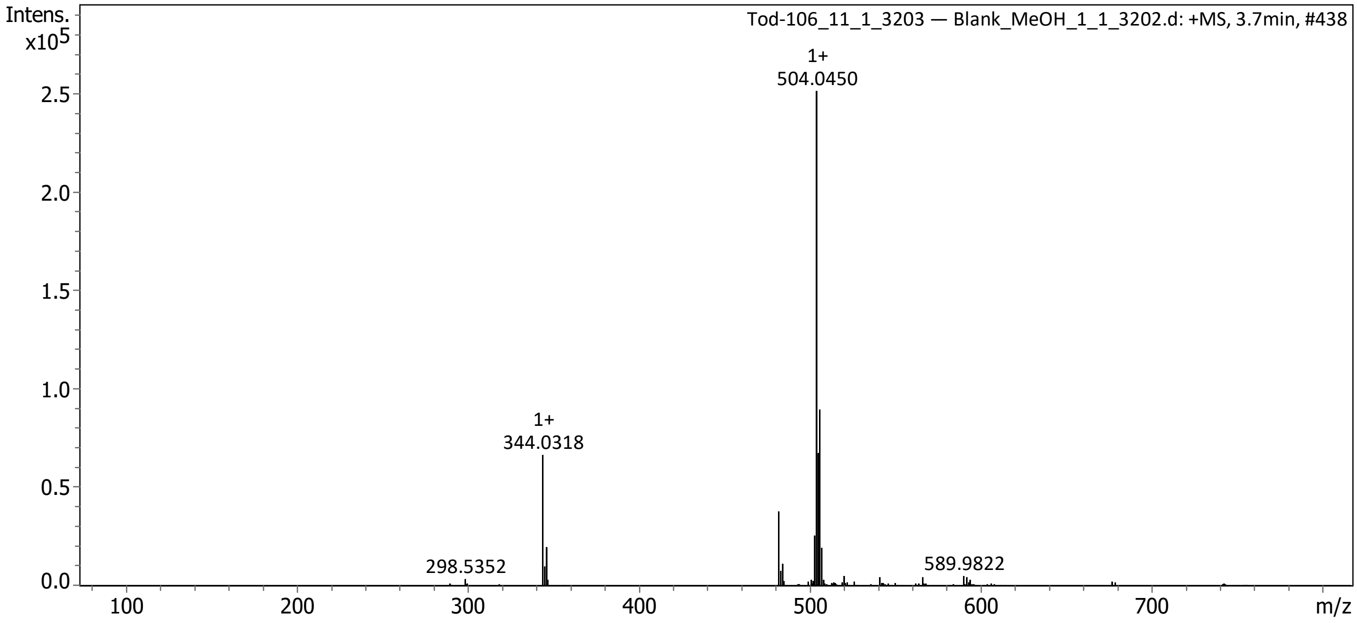

Compound Spectrum SmartFormula Report

Analysis Info

Analysis Name	D:\Data\IOPC\Babaev\2026\Burilov\T67_3_1_3857 — blank_H2O-ACN_2_1_3856.d	Acquisition Date	9/28/2026 12:00:59 PM
Method	LCMS_pos_8min_5-95B1_50_1300mz.m	Operator	Demo User
Sample Name	T67	Instrument	impact II 1825265.00000
Comment	Original analysis: 'T67_3_1_3857.d',<br>Subtracted analysis: 'blank_H2O-ACN_2_1_3856.d' (Xpose, 0.5s, 5),<br>Processed with: 'DataAnalysis 5.3.556',<br>Date: 2026-09-28T13:05:42		

Acquisition Parameter

Source Type	ESI	Ion Polarity	Positive	Set Nebulizer	2.0 Bar
Focus	Not active	Set Capillary	4500 V	Set Dry Heater	220 °C
Scan Begin	50 m/z	Set End Plate Offset	-500 V	Set Dry Gas	6.0 l/min
Scan End	1900 m/z	Set Collision Cell RF	1500.0 Vpp	Set Divert Valve	Source

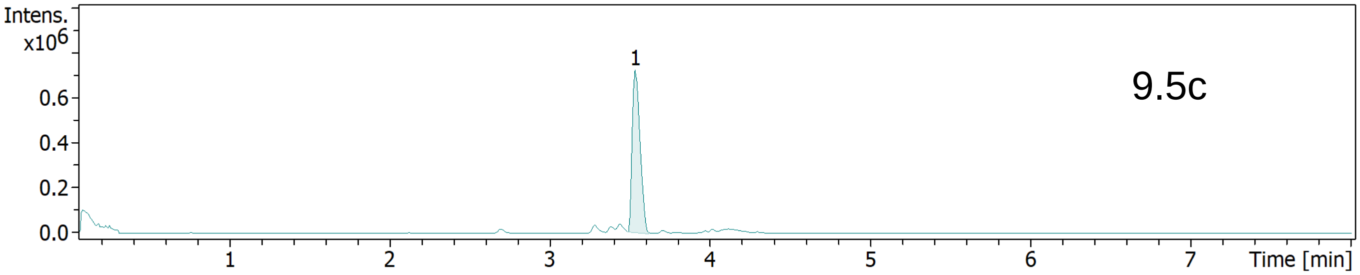

#	RT [min]	Int. Type	Trace	Area	Range [min]	S/N	I
1	3.5	Manual	BPC +All MS	2407890	3.5 - 3.6	4541.9	721801

Cmpd 1, 3.5 min

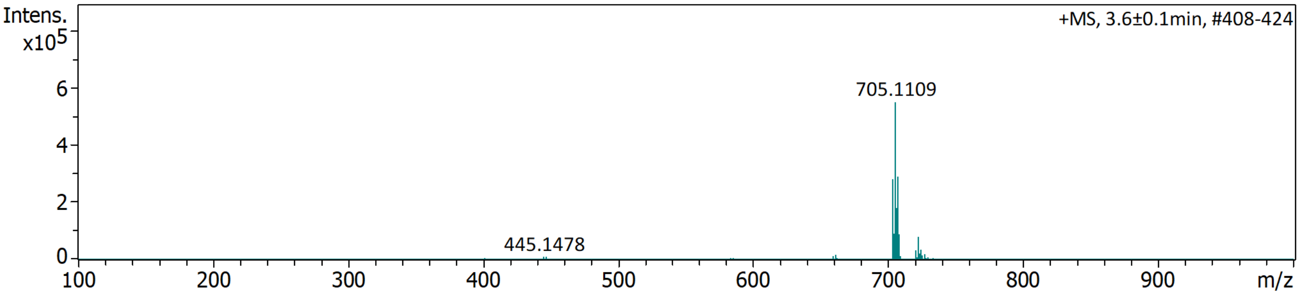

+MS, 0.2±0.1min, #6-19

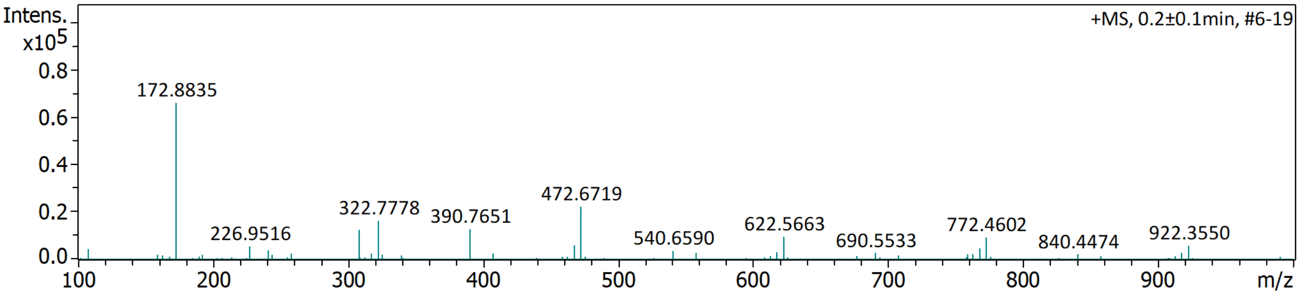

Compound Spectrum SmartFormula Report

Analysis Info

Analysis Name	D:\Data\IOPC\Babaev\2026\Burilov\T56a_9_1_3878 — blank_H2O-ACN_1_1_3876.d	Acquisition Date	10/14/2026 2:53:39 PM	
Method	LCMS_pos_8min_5-95B1_50_1300mz.m	Operator	Demo User	
Sample Name	T56a	Instrument	impact II	1825265.00000
Comment	Original analysis: 'T56a_9_1_3878.d',<br>Subtracted analysis: 'blank_H2O-ACN_1_1_3876.d' (Xpose, 0.5s, 5),<br>Processed with: 'DataAnalysis 5.3.556',<br>Date: 2026-10-14T15:06:48			

Acquisition Parameter

Source Type	ESI	Ion Polarity	Positive	Set Nebulizer	2.0 Bar
Focus	Not active	Set Capillary	4500 V	Set Dry Heater	220 °C
Scan Begin	50 m/z	Set End Plate Offset	-500 V	Set Dry Gas	6.0 l/min
Scan End	1900 m/z	Set Collision Cell RF	1500.0 Vpp	Set Divert Valve	Source

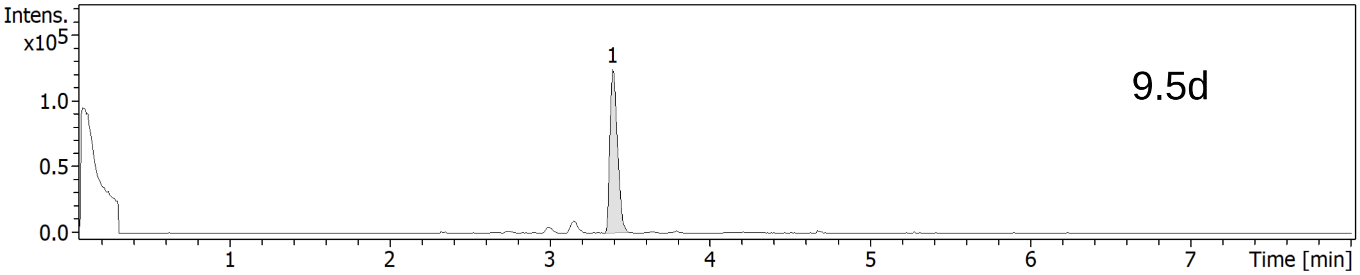

#	RT [min]	Int. Type	Trace	Area	Range [min]	S/N	I
1	3.4	Manual	BPC +All MS	403579	3.3 - 3.5	1027.5	125074

Cmpd 1, 3.4 min

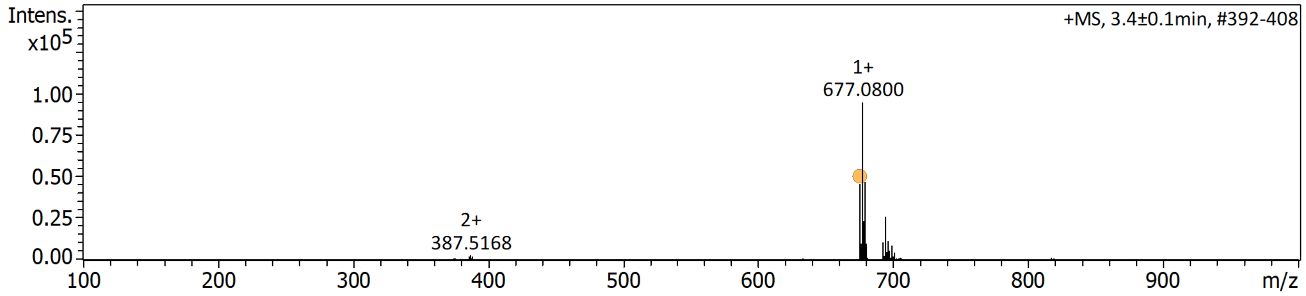

Meas. m/z	#	Ion Formula	m/z	err [ppm]	Mean err [ppm]	rdb	N-Rule	e⁻ Conf	mSigma	Std I	Std Mean m/z	Std I VarNorm	Std m/z Diff	Std Comb Dev
675.082087	1	C27H38Br2N2O6P	675.082876	1.2	1.4	15.0	ok	even	33.6	39.0	n.a.	n.a.	n.a.	n.a.

Compound Spectrum SmartFormula Report

Analysis Info

		Acquisition Date	9/28/2026 12:34:08 PM	
Analysis Name	D:\Data\IOPC\Babaev\2026\Burilov\T10_7_1_3861 — blank_H2O-ACN_2_1_3856.d			
Method	LCMS_pos_8min_5-95B1_50_1300mz.m	Operator	Demo User	
Sample Name	T10	Instrument	impact II	1825265.00000
Comment	Original analysis: 'T10_7_1_3861.d',<br>Subtracted analysis: 'blank_H2O-ACN_2_1_3856.d' (Xpose, 0.5s, 5),<br>Processed with: 'DataAnalysis 5.3.556',<br>Date: 2026-09-28T13:07:33			

Acquisition Parameter

Source Type	ESI	Ion Polarity	Positive	Set Nebulizer	2.0 Bar
Focus	Not active	Set Capillary	4500 V	Set Dry Heater	220 °C
Scan Begin	50 m/z	Set End Plate Offset	-500 V	Set Dry Gas	6.0 l/min
Scan End	1900 m/z	Set Collision Cell RF	1500.0 Vpp	Set Divert Valve	Source

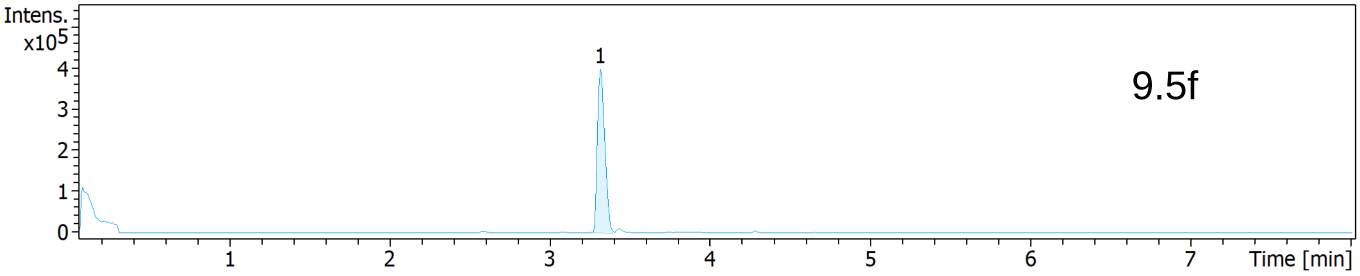

#	RT [min]	Int. Type	Trace	Area	Range [min]	S/N	I
1	3.3	Manual	BPC +All MS	1246330	3.3 - 3.4	3665.7	399029

Cmpd 1, 3.3 min

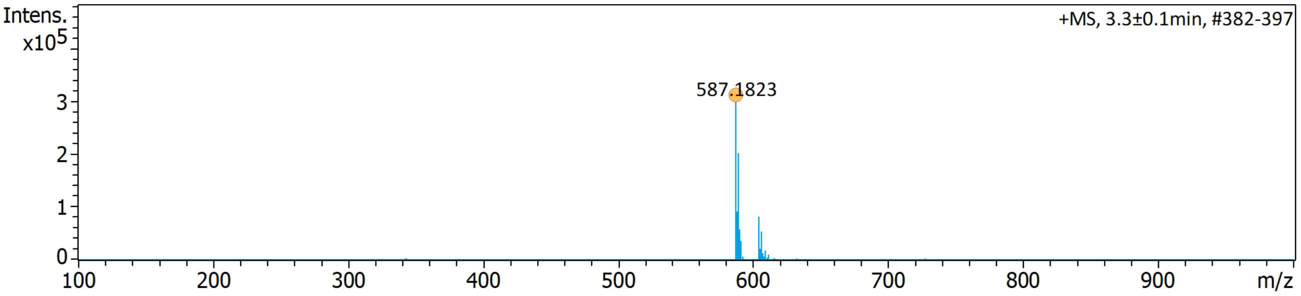

Meas. m/z	#	Ion Formula	m/z	err [ppm]	Mean err [ppm]	rdb	N-Rule	e⁻ Conf	mSigma	Std I	Std Mean m/z	Std I VarNorm	Std m/z Diff	Std Comb Dev
587.182316	1	C27H38Cl2N2O6P	587.183906	2.7	3.0	17.0	ok	even	11.2	12.2	n.a.	n.a.	n.a.	n.a.
	1	C27H38Cl2N2O6P	587.183906	2.7	3.0	17.0	ok	even	11.2	12.2	n.a.	n.a.	n.a.	n.a.

+MS, 0.1min, #6-17

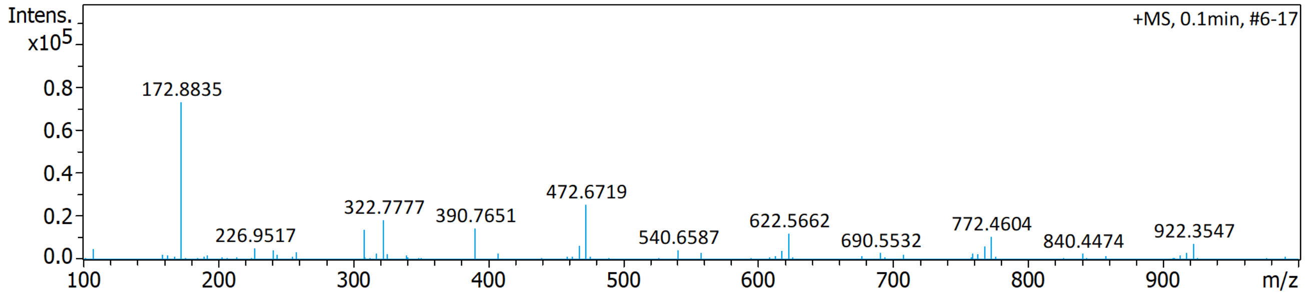
Intens.
x10^5
+MS, 0.1min, #6-17
0.8
0.6
0.4
0.2
0.0
172.8835
226.9517
322.7777
390.7651
472.6719
540.6587
622.5662
690.5532
772.4604
840.4474
922.3547
100
200
300
400
500
600
700
800
900
m/z

9.5f

Compound Spectrum SmartFormula Report

Analysis Info

Analysis Name	D:\Data\IOPC\Babaev\2026\Burilov\T68_11_1_3881 — blank_H2O-ACN_1_1_3876.d	Acquisition Date	10/14/2026 3:18:31 PM
Method	LCMS_pos_8min_5-95B1_50_1300mz.m	Operator	Demo User
Sample Name	T68	Instrument	impact II 1825265.00000
Comment	Original analysis: 'T68_11_1_3881.d',<br>Subtracted analysis: 'blank_H2O-ACN_1_1_3876.d' (Xpose, 0.5s, 5),<br>Processed with: 'DataAnalysis 5.3.556',<br>Date: 2026-10-14T15:34:29		

Acquisition Parameter

Source Type	ESI	Ion Polarity	Positive	Set Nebulizer	2.0 Bar
Focus	Not active	Set Capillary	4500 V	Set Dry Heater	220 °C
Scan Begin	50 m/z	Set End Plate Offset	-500 V	Set Dry Gas	6.0 l/min
Scan End	1900 m/z	Set Collision Cell RF	1500.0 Vpp	Set Divert Valve	Source

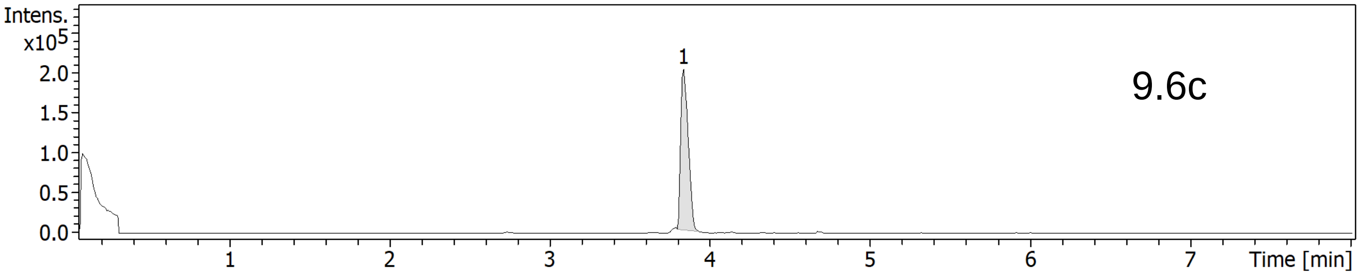

#	RT [min]	Int. Type	Trace	Area	Range [min]	S/N	I
1	3.8	Manual	BPC +All MS	642426	3.8 - 3.9	2187.2	204516

Cmpd 1, 3.8 min

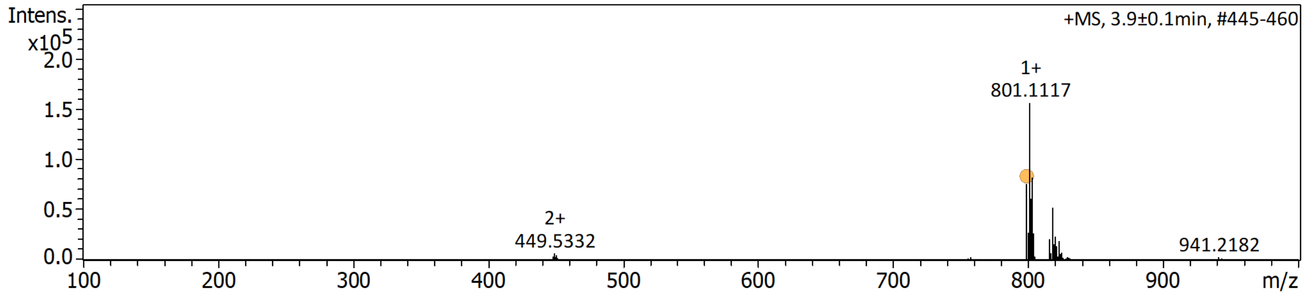

Meas. m/z	#	Ion Formula	m/z	err [ppm]	Mean err [ppm]	rdb	N-Rule	e⁻ Conf	mSigma	Std I	Std Mean m/z	Std I VarNorm	Std m/z Diff	Std Comb Dev
799.113729	1	C37H42Br2N2O6P	799.114176	0.6	0.6	23.0	ok	even	22.4	22.4	n.a.	n.a.	n.a.	n.a.

Compound Spectrum SmartFormula Report

Analysis Info

Analysis Name	D:\Data\IOPC\Babaev\2026\Burilov\T32_6_1_3860 — blank_H2O-ACN_2_1_3856.d	Acquisition Date	9/28/2026 12:25:50 PM
Method	LCMS_pos_8min_5-95B1_50_1300mz.m	Operator	Demo User
Sample Name	T32	Instrument	impact II 1825265.00000
Comment	Original analysis: 'T32_6_1_3860.d',<br>Subtracted analysis: 'blank_H2O-ACN_2_1_3856.d' (Xpose, 0.5s, 5),<br>Processed with: 'DataAnalysis 5.3.556',<br>Date: 2026-09-28T13:07:12		

Acquisition Parameter

Source Type	ESI	Ion Polarity	Positive	Set Nebulizer	2.0 Bar
Focus	Not active	Set Capillary	4500 V	Set Dry Heater	220 °C
Scan Begin	50 m/z	Set End Plate Offset	-500 V	Set Dry Gas	6.0 l/min
Scan End	1900 m/z	Set Collision Cell RF	1500.0 Vpp	Set Divert Valve	Source

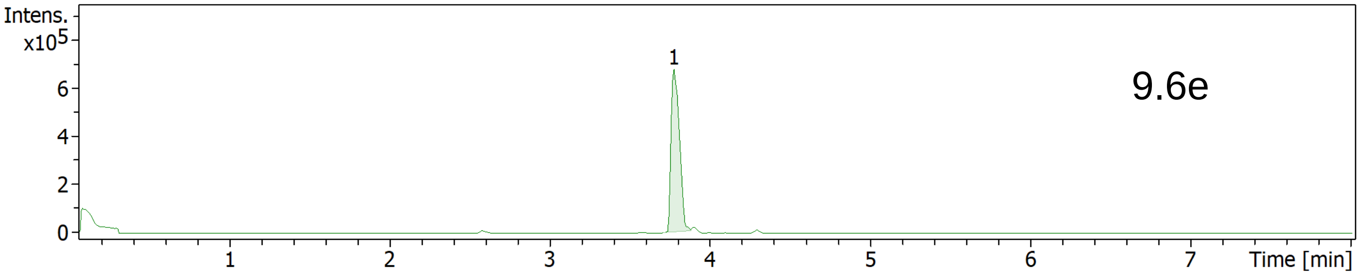

#	RT [min]	Int. Type	Trace	Area	Range [min]	S/N	I
1	3.8	Manual	BPC +All MS	2413650	3.7 - 3.9	5728.4	677627

Cmpd 1, 3.8 min

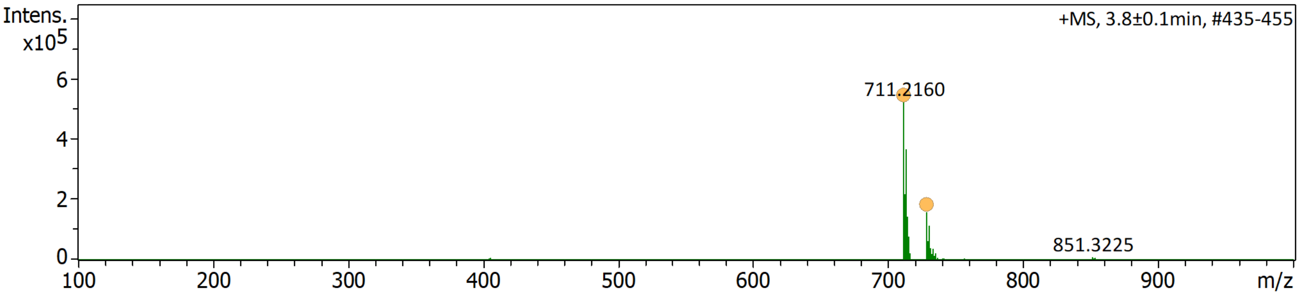

Meas. m/z	#	Ion Formula	m/z	err [ppm]	Mean err [ppm]	rdb	N-Rule	e⁻ Conf	mSigma	Std I	Std Mean m/z	Std I VarNorm	Std m/z Diff	Std Comb Dev
711.216042	1	C37H42Cl2N2O6P	711.215206	-1.2	-1.0	25.0	ok	even	15.4	13.3	n.a.	n.a.	n.a.	n.a.
	1	C37H42Cl2N2O6P	711.215206	-1.2	-1.0	25.0	ok	even	15.4	13.3	n.a.	n.a.	n.a.	n.a.
	1	C37H42Cl2N2O6P	711.215206	-1.2	-1.0	25.0	ok	even	15.4	13.3	n.a.	n.a.	n.a.	n.a.
	1	C37H42Cl2N2O6P	711.215206	-1.2	-1.0	25.0	ok	even	15.4	13.3	n.a.	n.a.	n.a.	n.a.
728.242533	1	C37H45Cl2N3O6P	728.241755	-1.1	9.6	24.0	ok	even	81.2	83.4	n.a.	n.a.	n.a.	n.a.
	1	C37H45Cl2N3O6P	728.241755	-1.1	9.6	24.0	ok	even	81.2	83.4	n.a.	n.a.	n.a.	n.a.

+MS, 0.2min, #8-19

Compound Spectrum SmartFormula Report

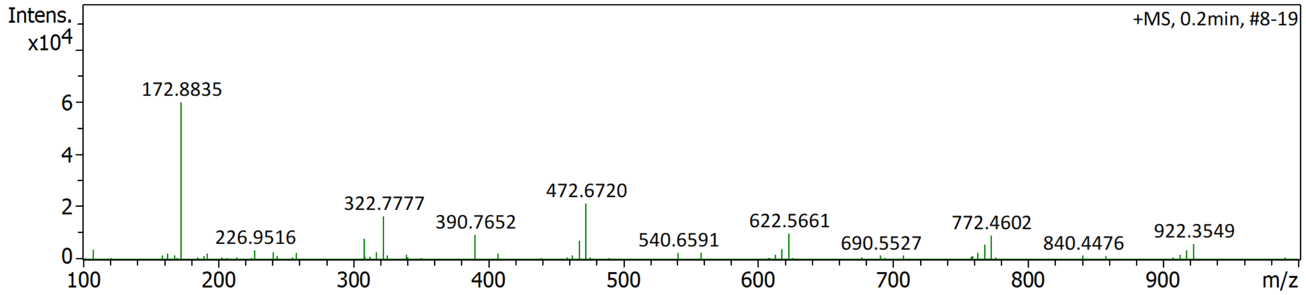

9.6e
