# Supplementary material for: Combining Ligand- and Structure-Based Approaches to Develop Haloacetamide-Derived Di(hetero)arylmethanes as Potential Anticancer Agents
Source: Int J Mol Sci. 2026 Oct 26;27(21):9559. doi: 10.3390/ijms27219559 (PMC13762480; doi:10.3390/ijms27219559)
Supplement: Supplementary file 1 [file ijms-27-09559-s001.zip › SI_spectra.pdf]

# Combining ligand- and structure-based approaches to develop haloacetamide-derived di(hetero)arylmethanes as potential anticancer agents

Karina O. Shibaeva,[a] Andrey V. Smolobochkin,[a] Hoang Bao Tran Nguyen,[a] Adel M. Shakirov,[a] Elmira M. Gibadullina,[a] Anna P. Lyubina,[a] Anastasiia S. Sapunova,[a] Alexandra D. Voloshina,[a], Yulia R. Aleksandrova,[b] Margarita E. Neganova,[b] Almir S. Gazizov,[*a] Alexander R. Burilov,[a] Michail A. Pudovik[a]

[a] *Arbuzov Institute of Organic and Physical Chemistry, FRC Kazan Scientific Center, Russian Academy of Sciences, Arbuzova str. 8, Kazan, Russian Federation*, e-mail:agazizov@iopc.ru

[b] *A.N. Nesmeyanov Institute of Organoelement Compounds of Russian Academy of Sciences, Vavilova str., 28, Moscow, Russian Federation*

## Table of Contents

NMR spectra of starting compounds . . . . . . . . . . . . . . . . . . . . . . . . . . . . . . . . . . . . . . . . S1
NMR spectra of compounds 3-5, 9.5c,d,f and 9.6c,e . . . . . . . . . . . . . . . . . . . . . . . . . . . . . . S20

# NMR spectra of starting compounds

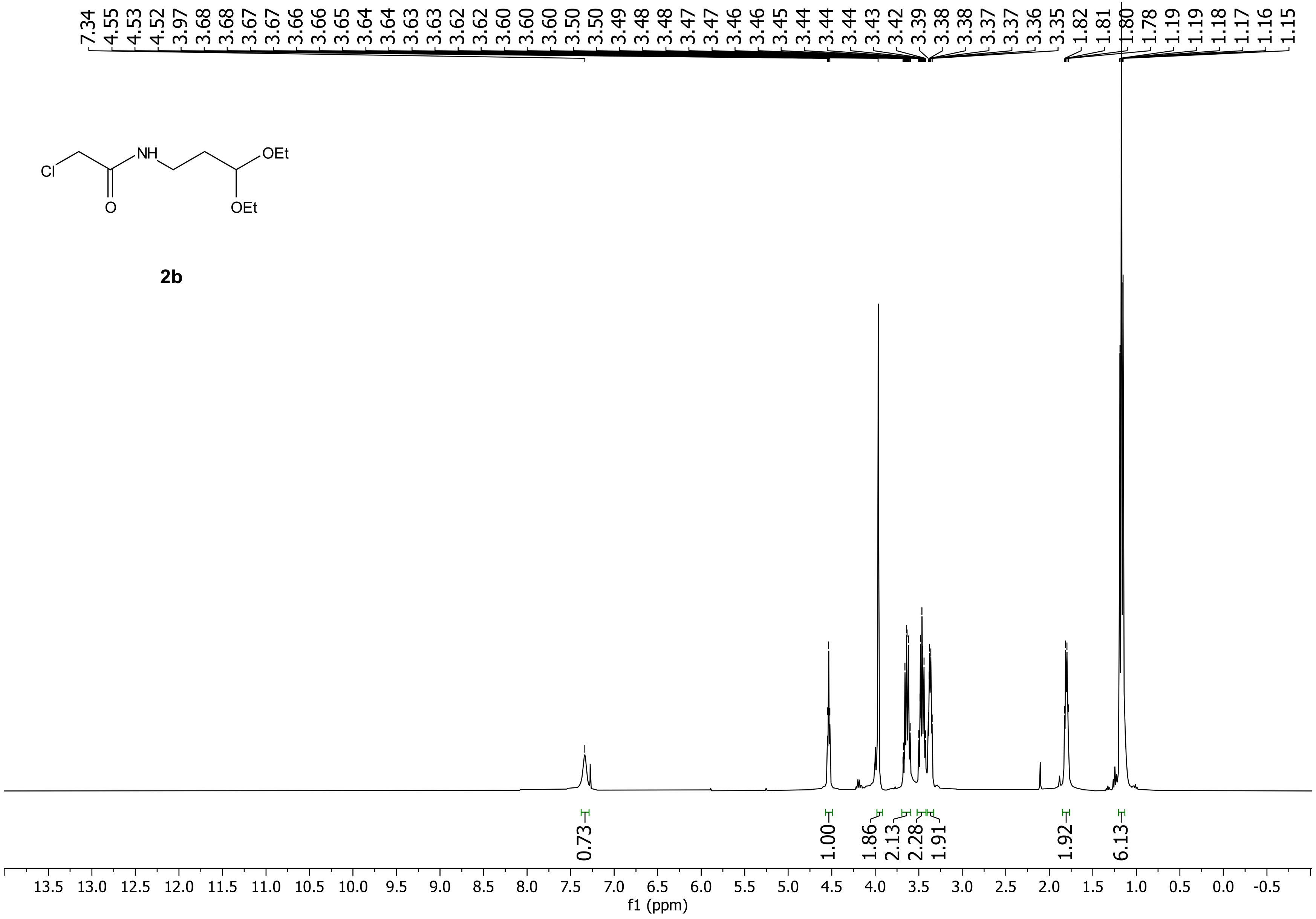

**Figure S23.** $^{1}H$ NMR spectrum ($CDCl_3$, 500MHz) of the compound **2b**

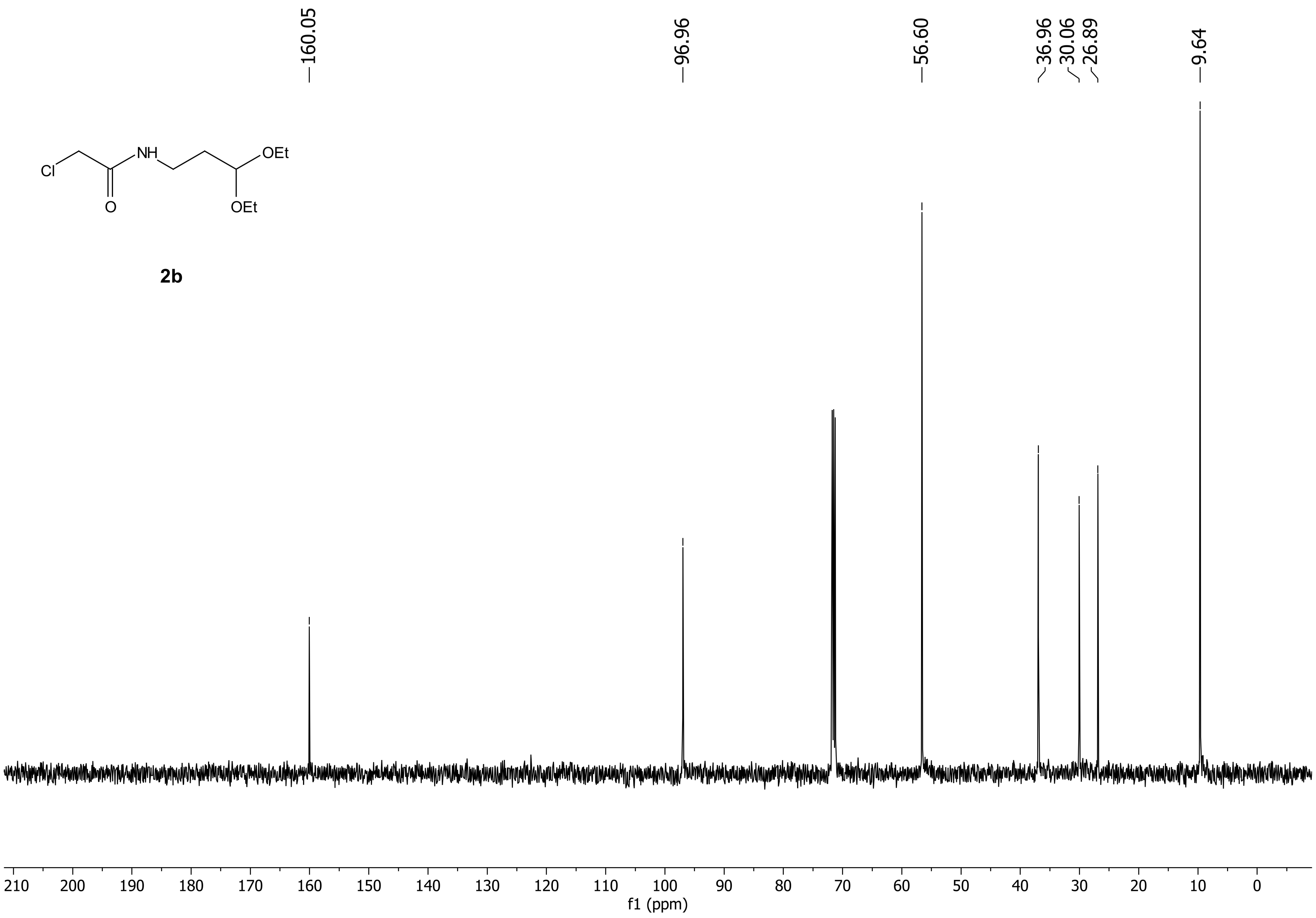


**Figure S24.** $^{13}C\{^1H\}$ NMR spectrum ($CDCl_3$, 126MHz) of the compound **2b**

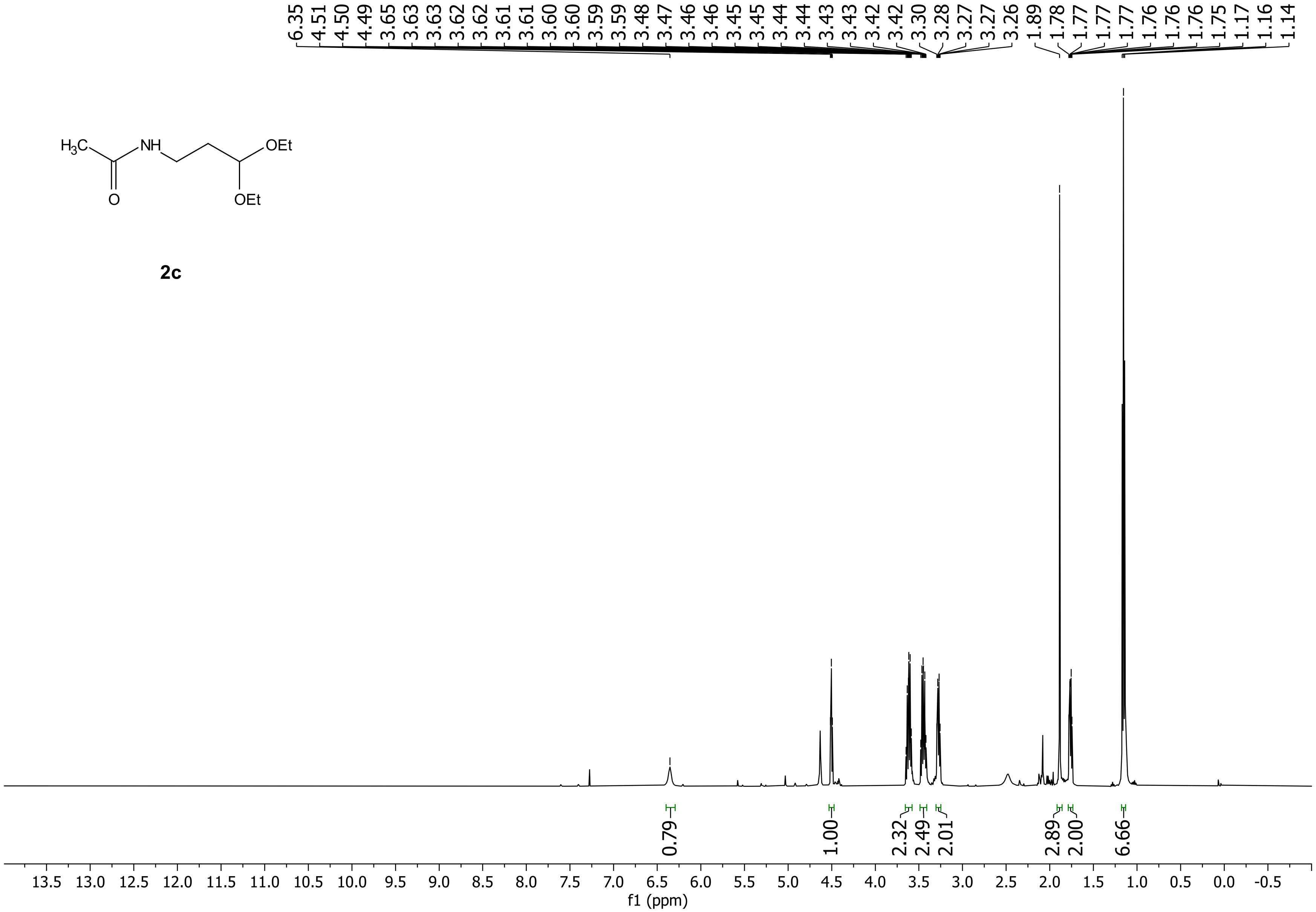

Figure S25. $^{1}H$ NMR spectrum ($CDCl_3$, 500MHz) of the compound **2c**

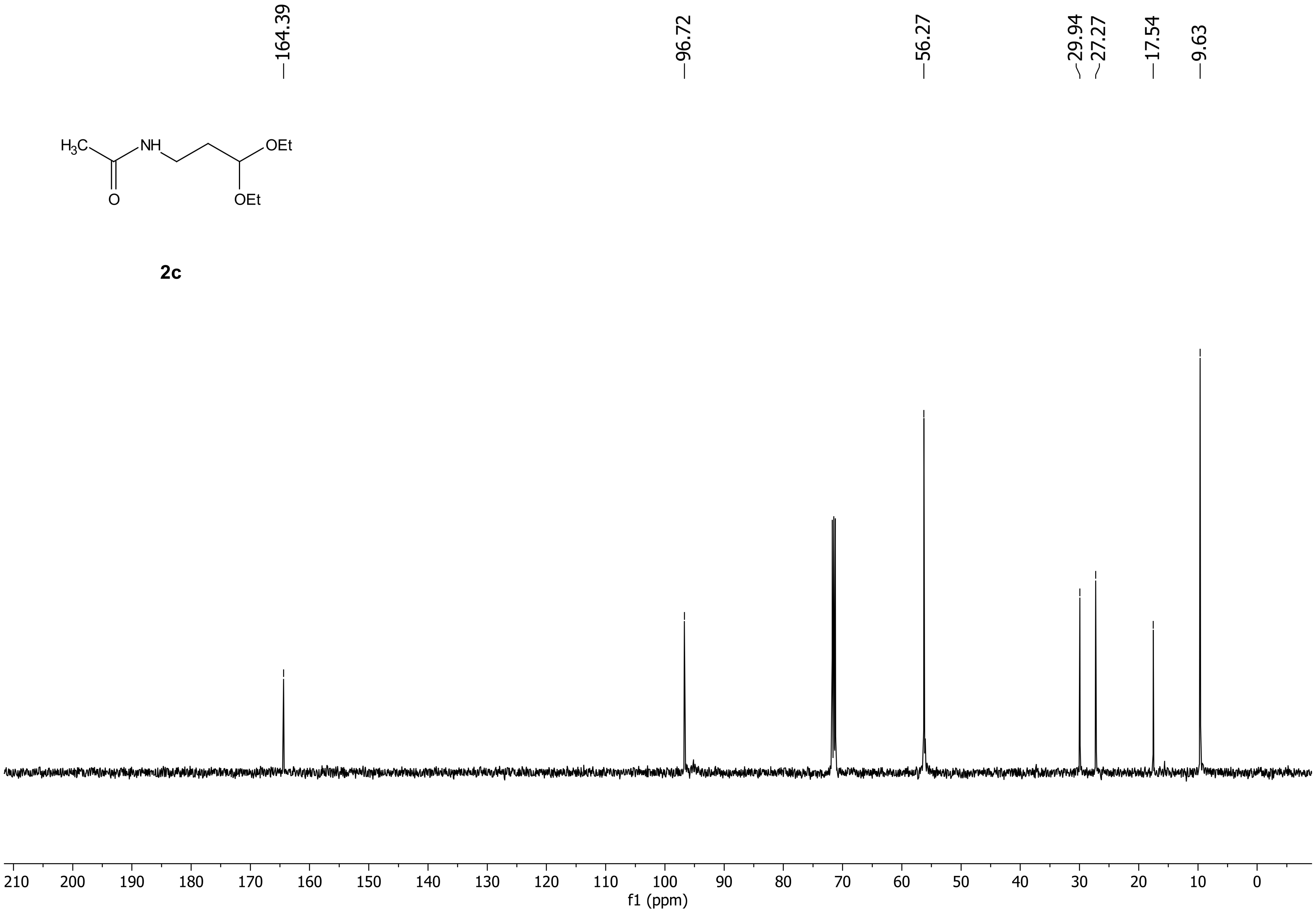


**2c**

**Figure S26.** $^{13}C\{^{1}H\}$ NMR spectrum ($CDCl_3$, 126MHz) of the compound **2c**

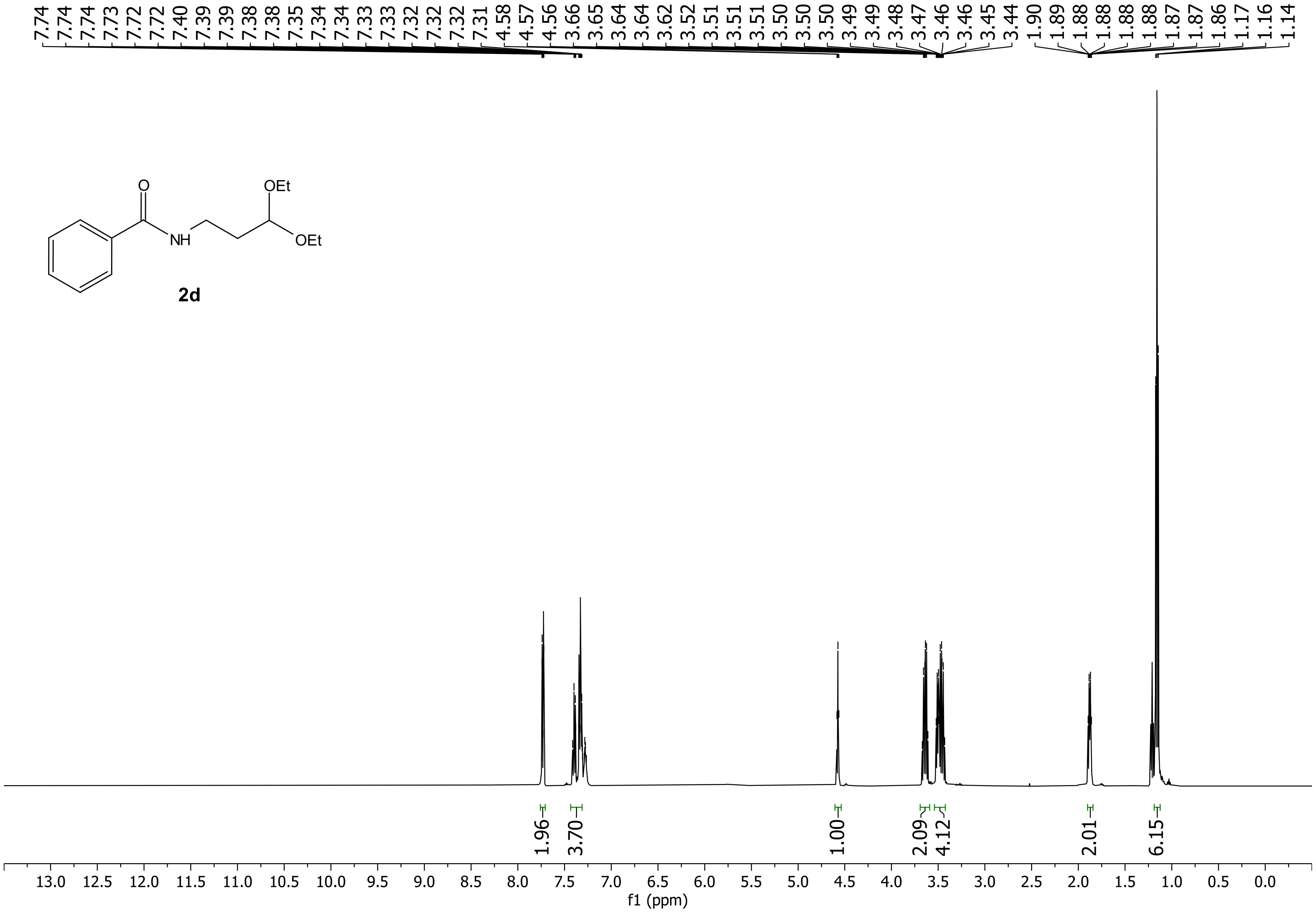

**Figure S27.** $^{1}H$ NMR spectrum ($CDCl_3$, 400MHz) of the compound **2d**

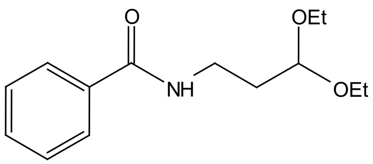


**2d**

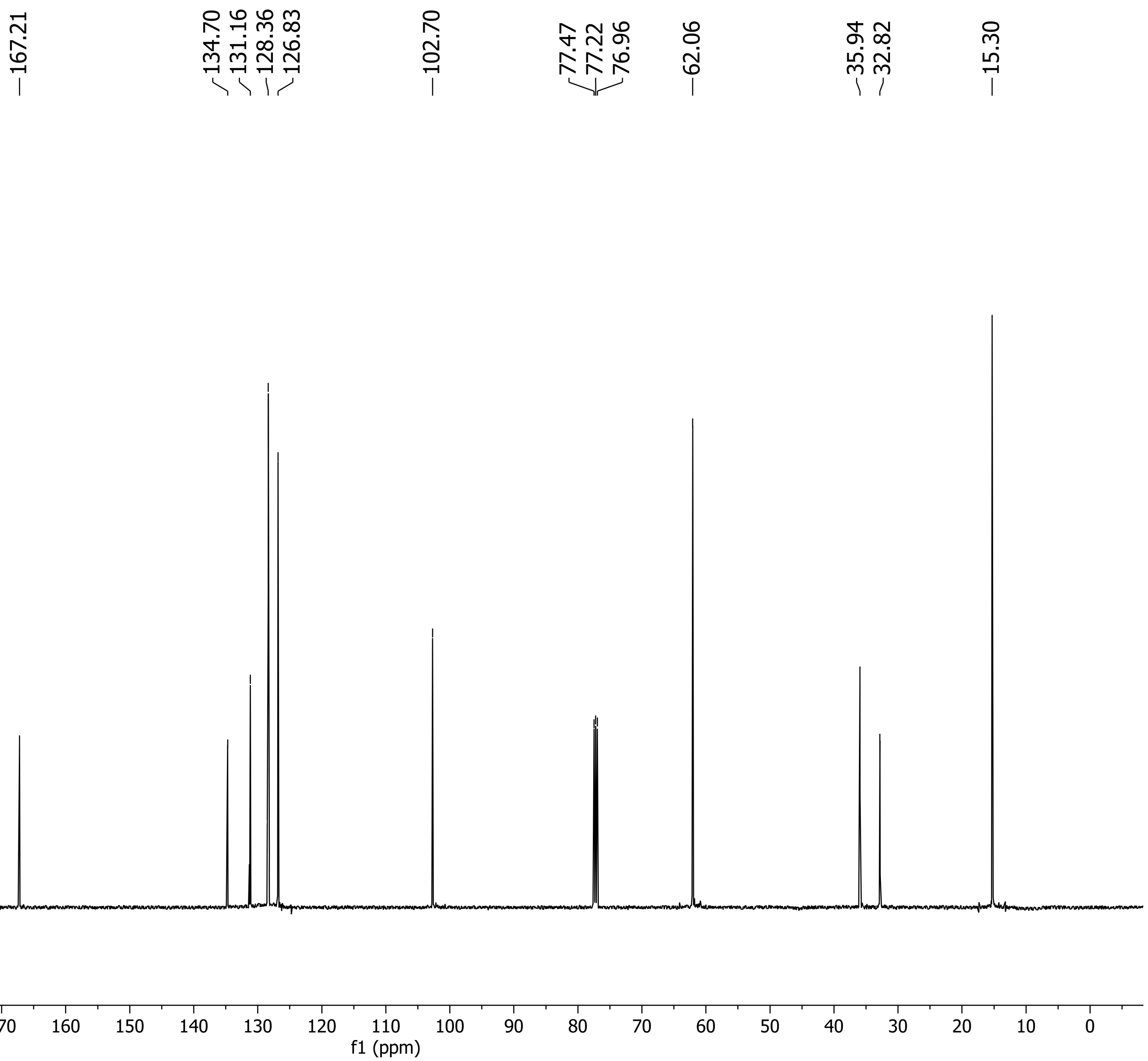


**Figure S28.** $^{13}C\{^1H\}$ NMR spectrum ($CDCl_3$, 126MHz) of the compound **2d**

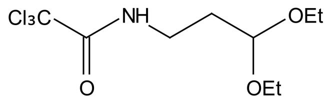


**2e**

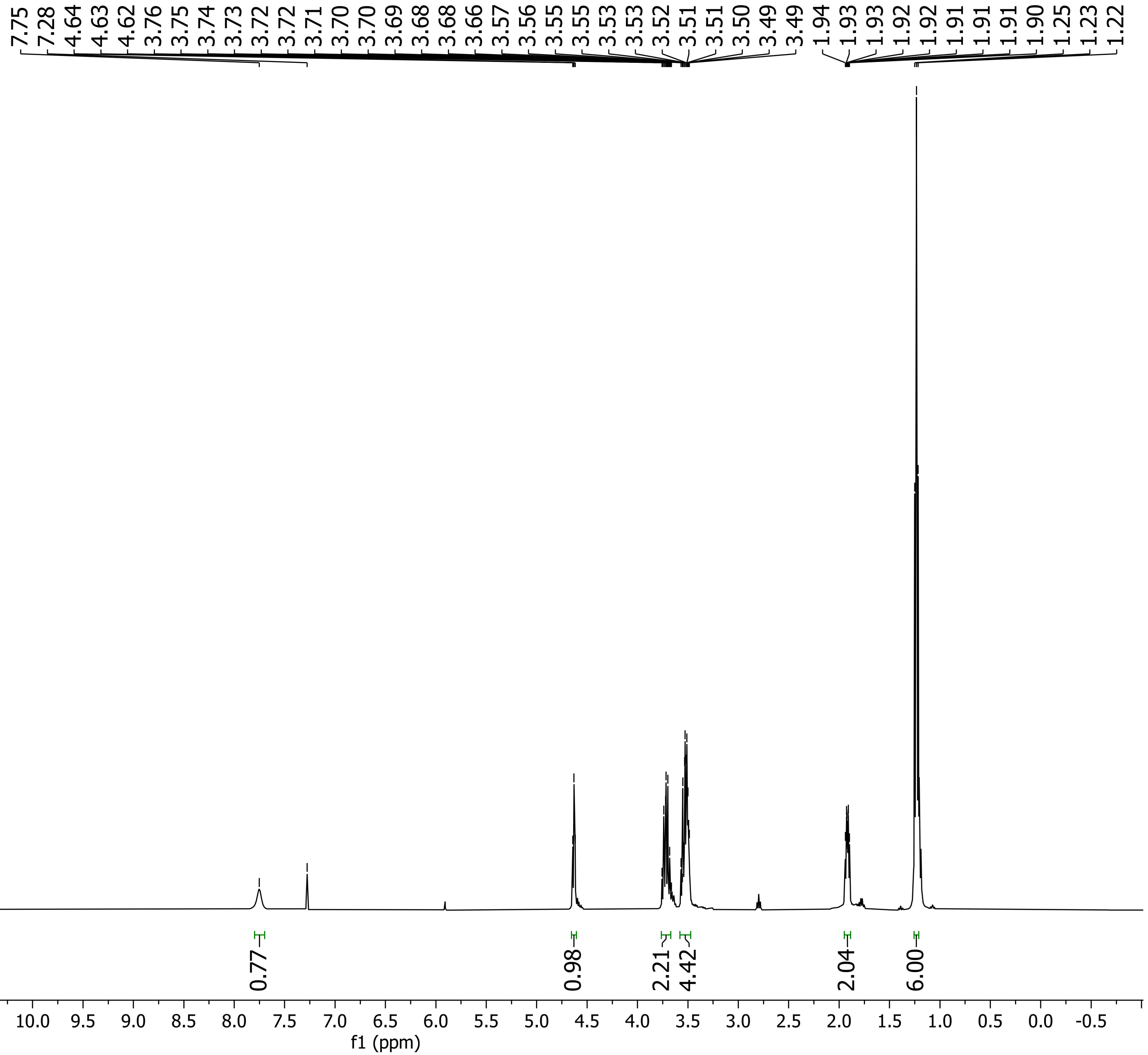


**Figure S29.** $^{1}H$ NMR spectrum ($CDCl_3$, 400MHz) of the compound **2e**

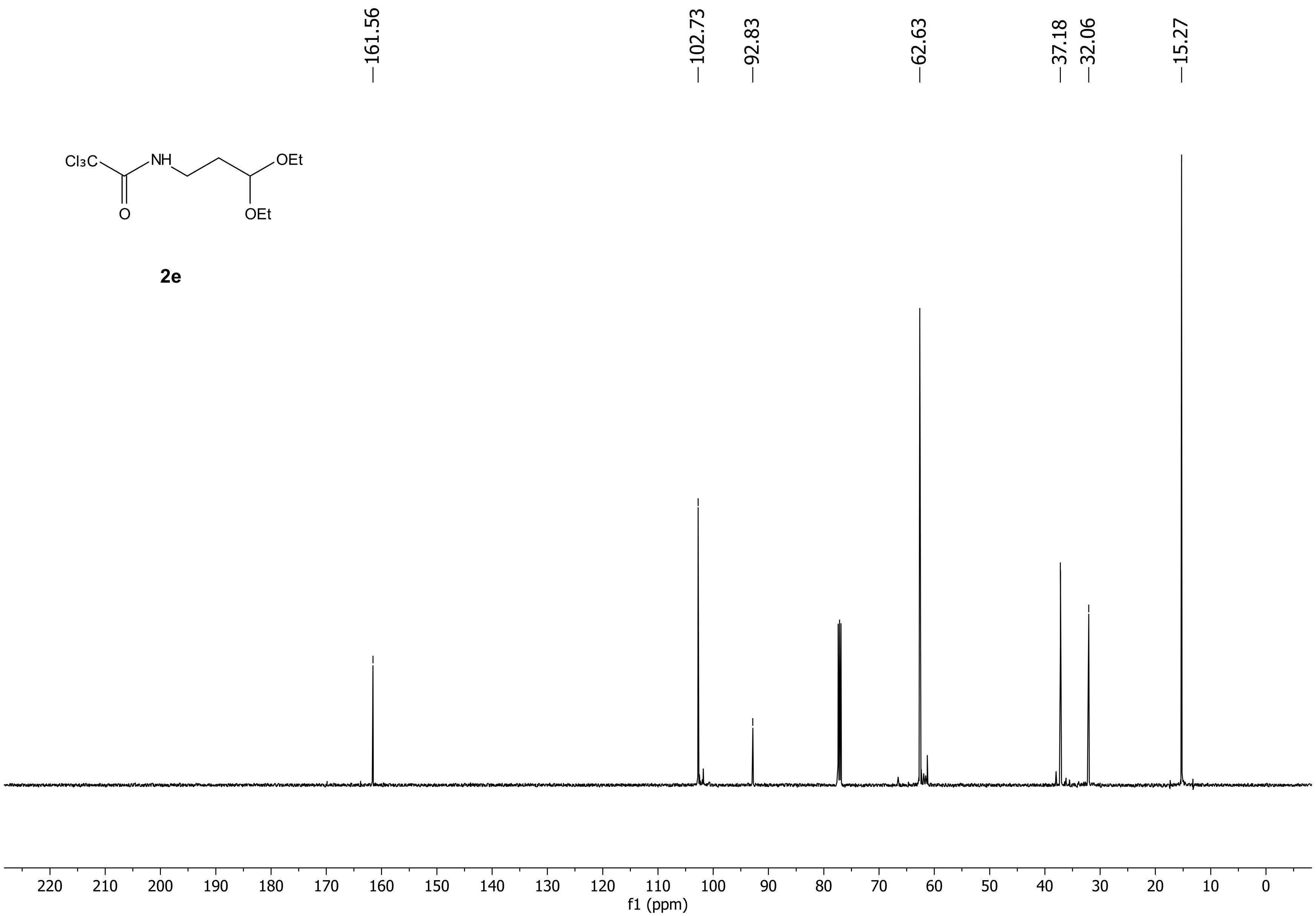


**Figure S30.** $^{13}C\{^{1}H\}$ NMR spectrum ($CDCl_3$, 126MHz) of the compound **2e**

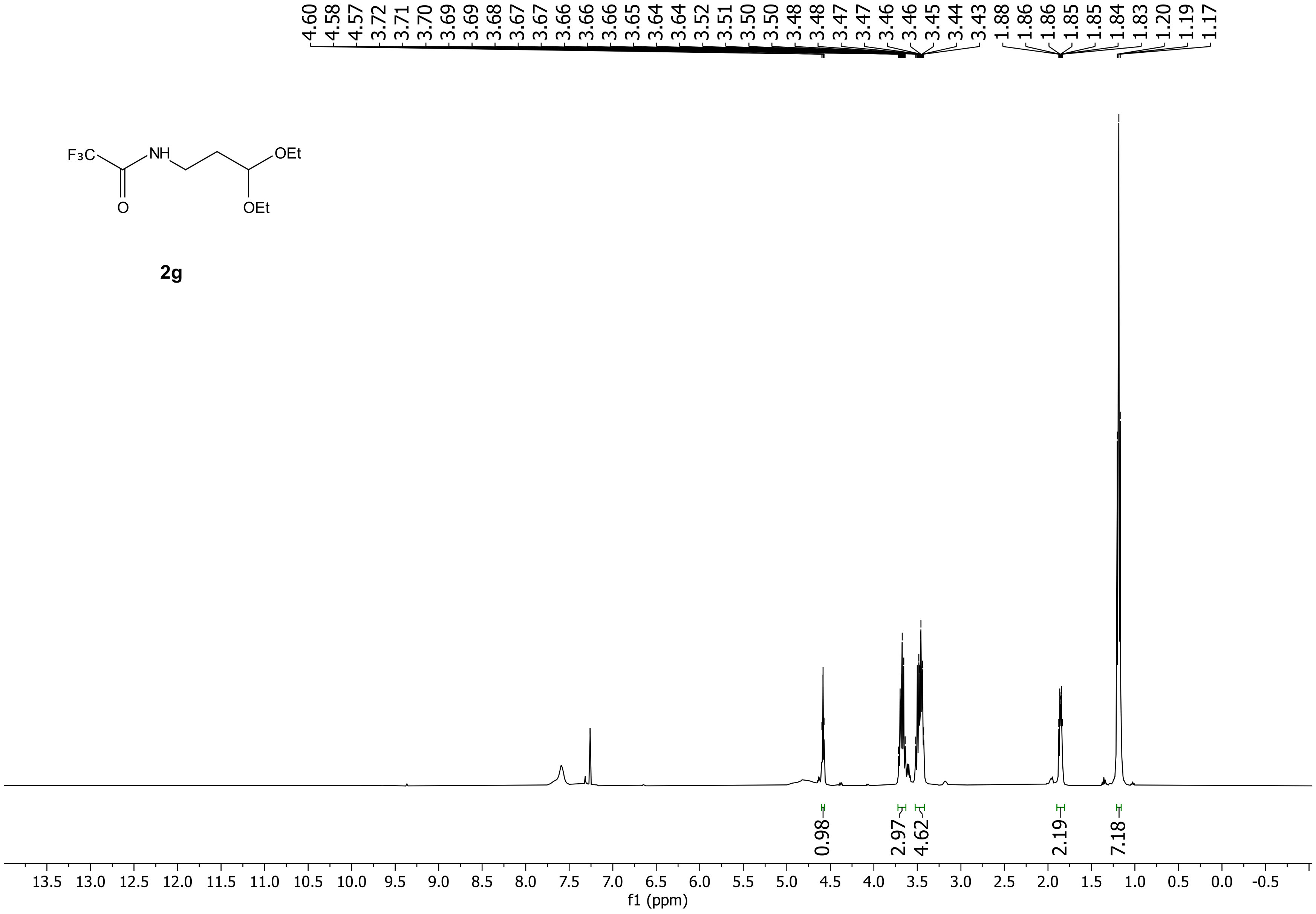

**Figure S31.** $^{1}H$ NMR spectrum ($CDCl_3$, 400MHz) of the compound **2g**

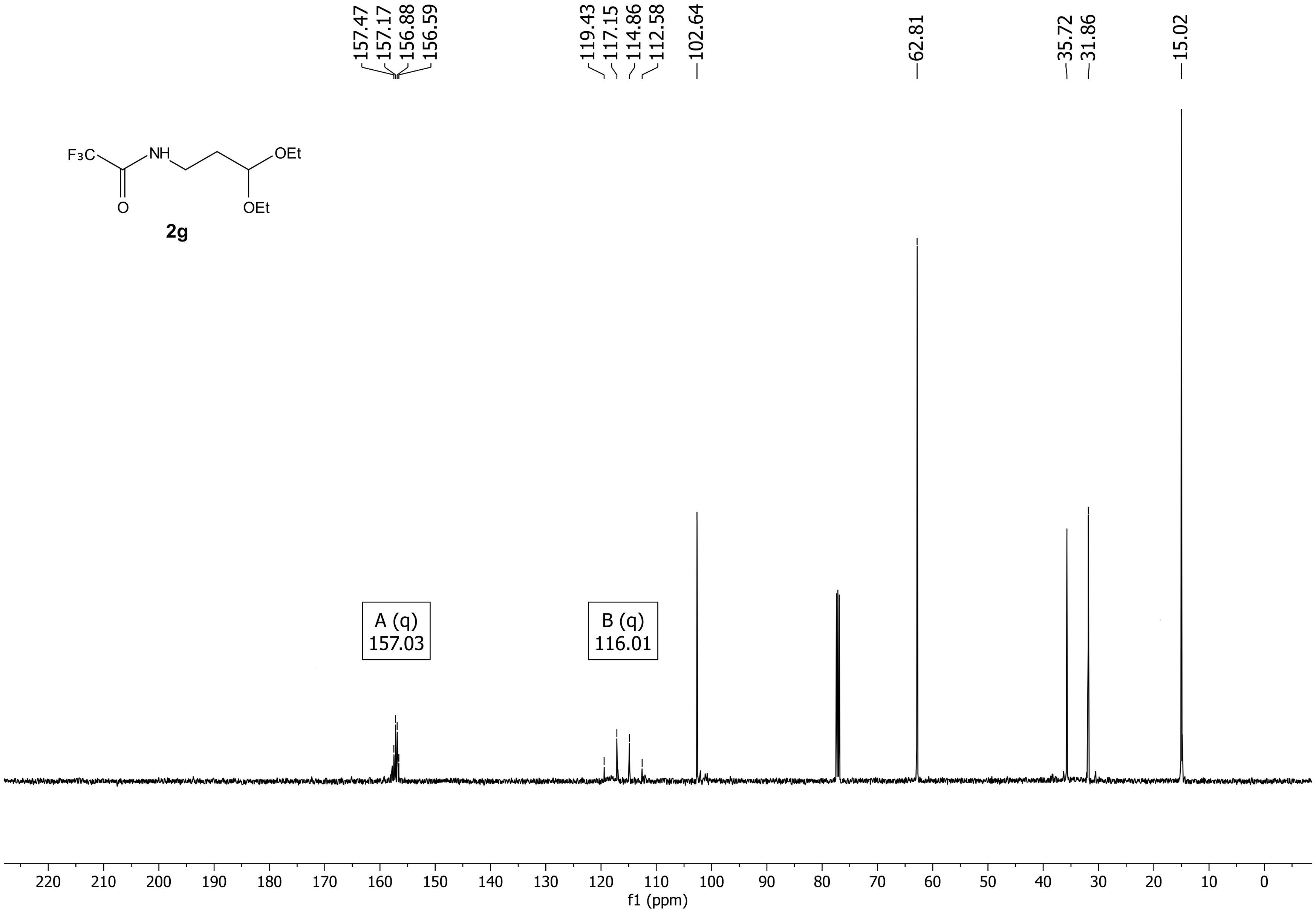


**Figure S32.** $^{13}C\{^{1}H\}$ NMR spectrum ($CDCl_3$, 126MHz) of the compound **2g**

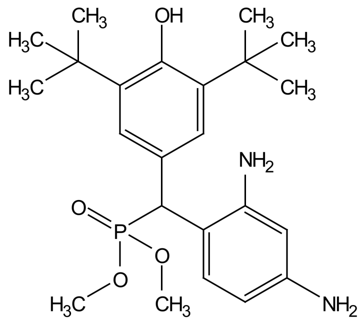

**12a**

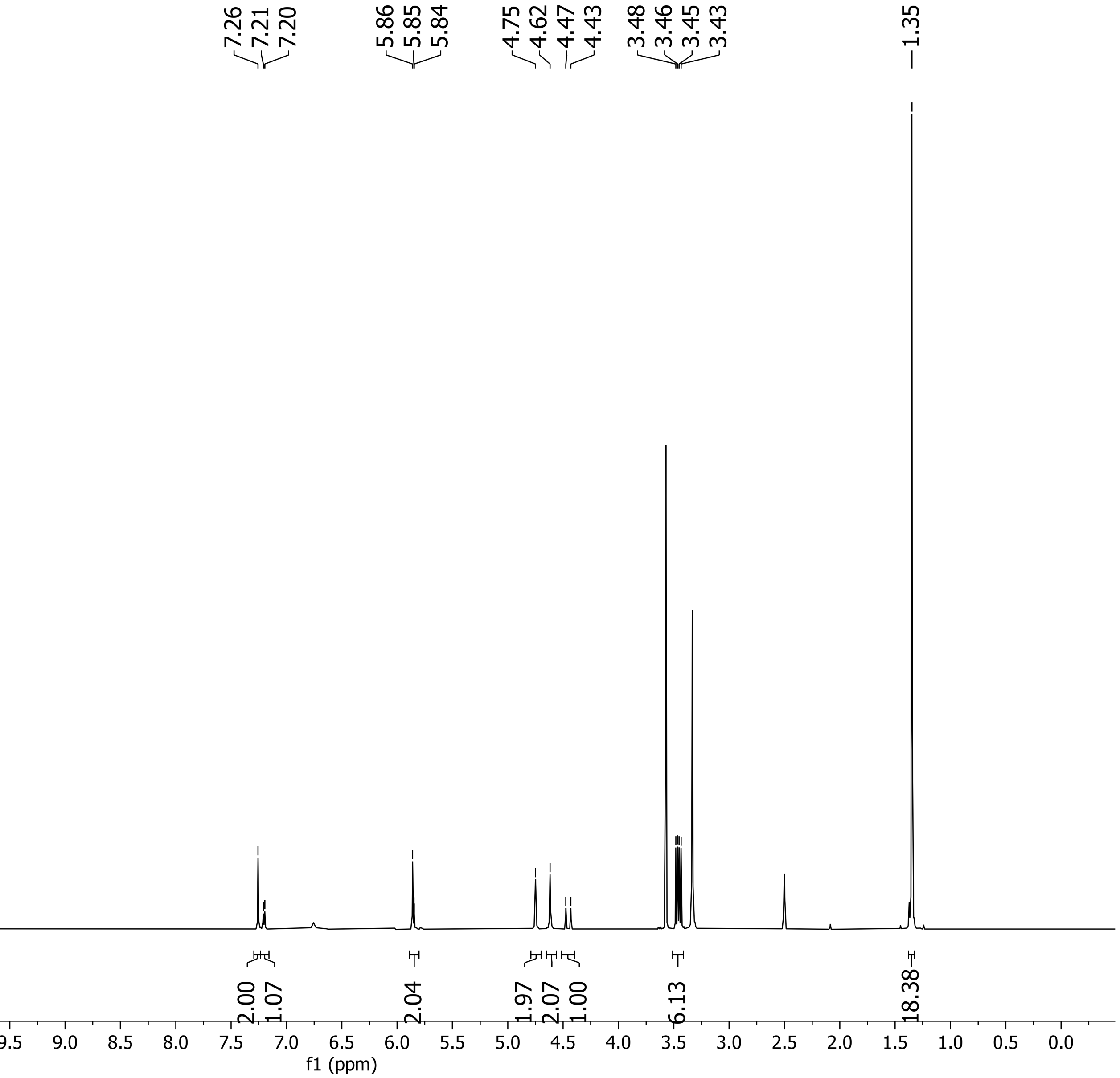

**Figure S33.** $^{1}H$ NMR spectrum ($(CD_3)_2SO$, 600MHz) of the compound **12a**

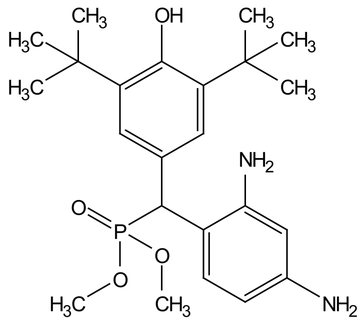


**12a**

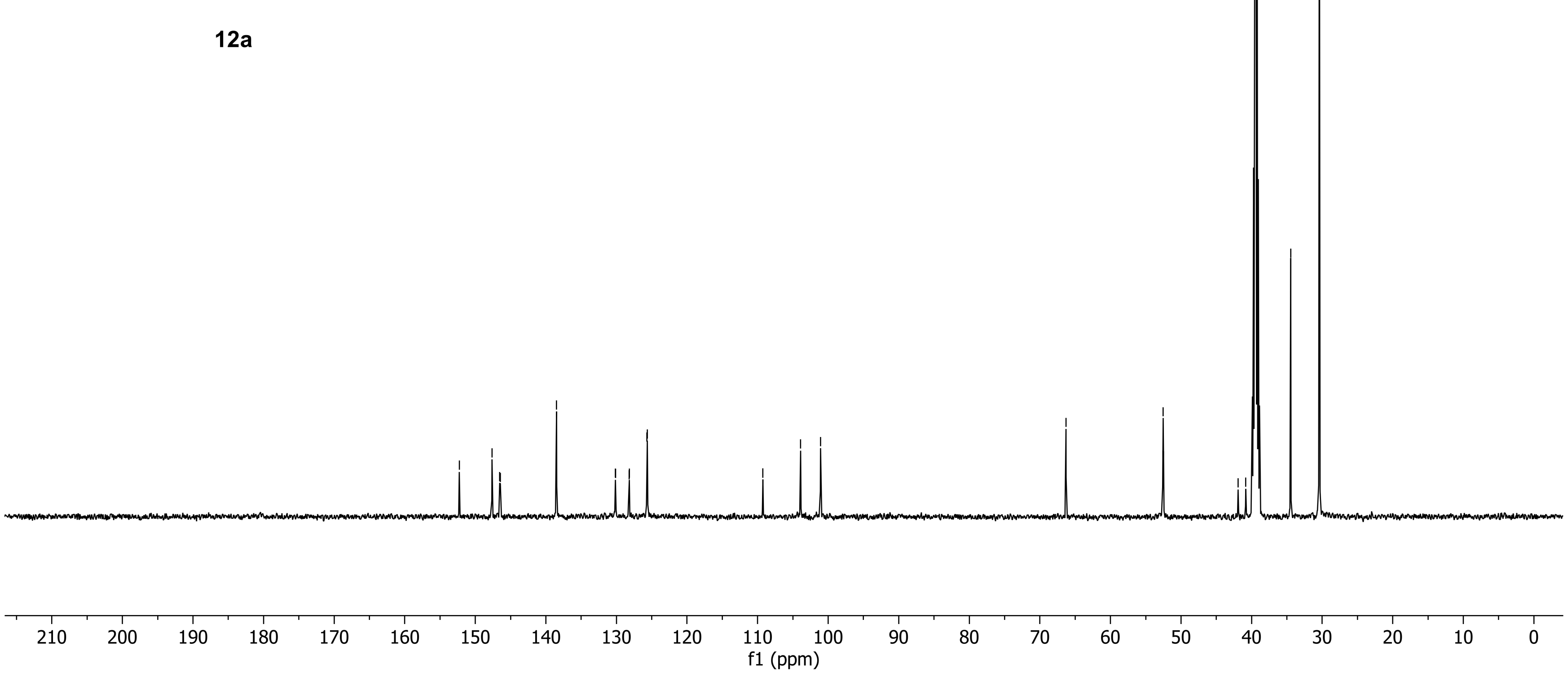


**Figure S34.** $^{13}C\{^{1}H\}$ NMR spectrum ($(CD_3)_2SO$, 101MHz) of the compound **12a**

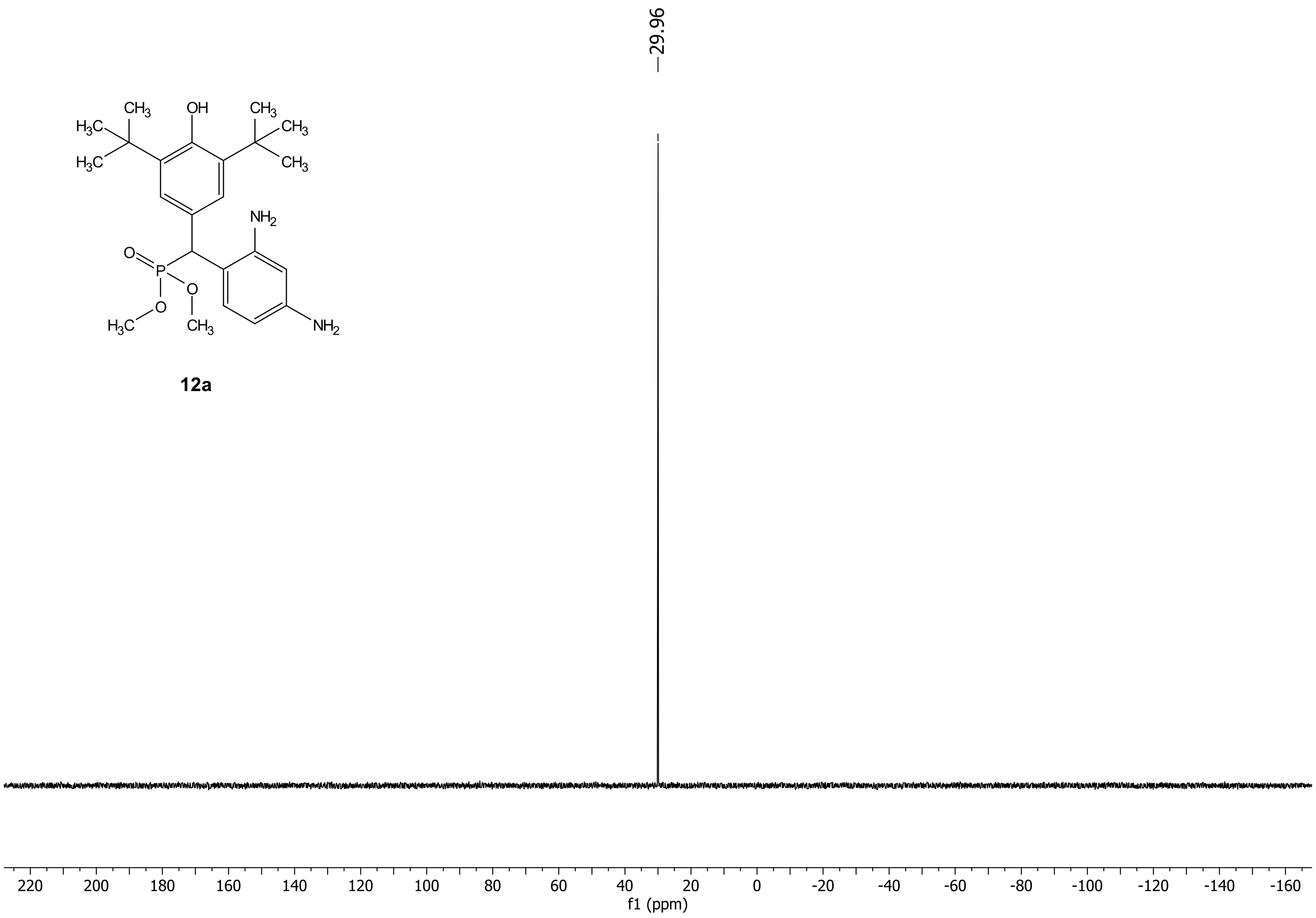


**Figure S35.** $^{31}P\{^{1}H\}$ NMR spectrum ($(CD_3)_2SO$, 243MHz) of the compound **12a**

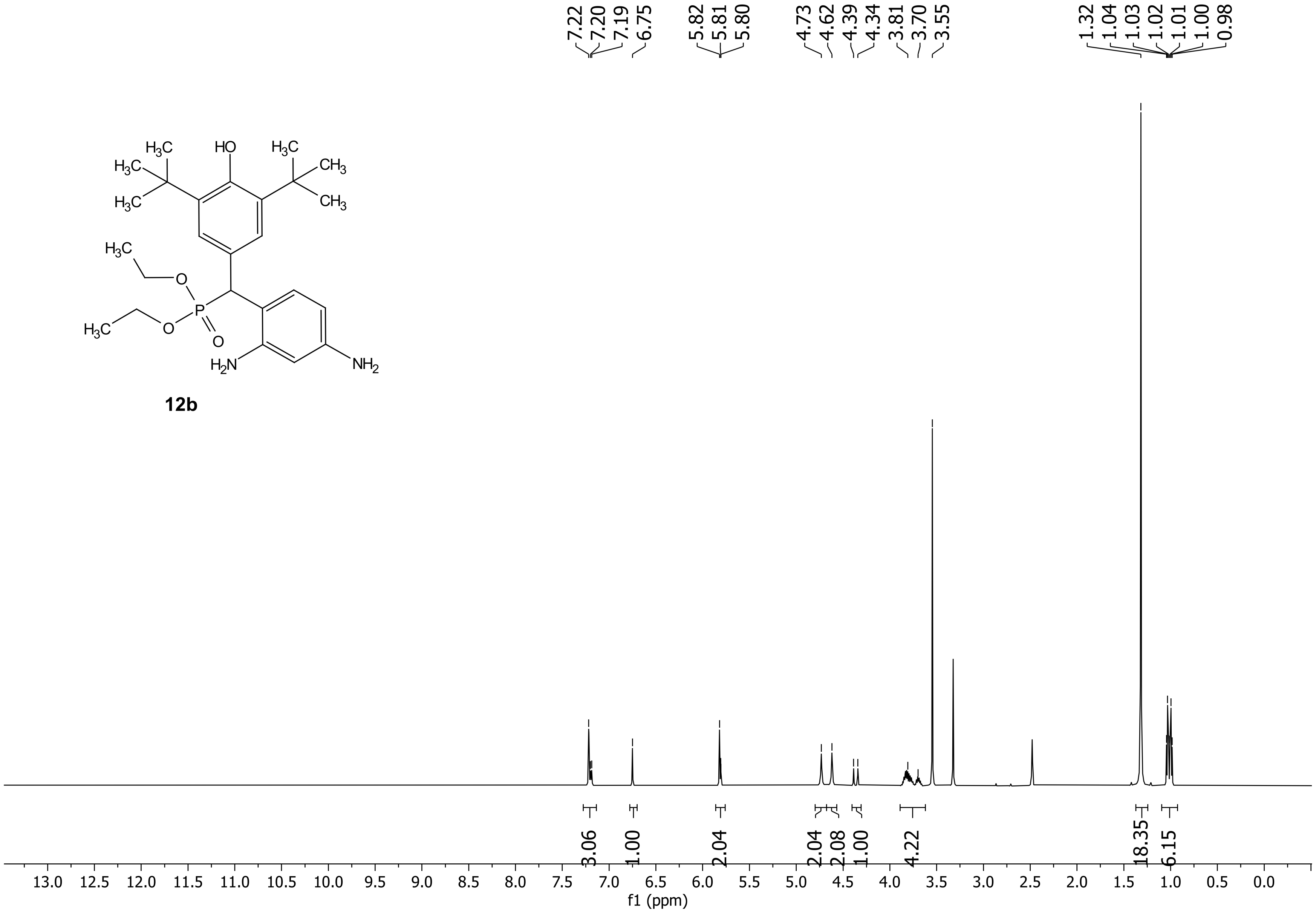


**Figure S36.** $^{1}$H NMR spectrum ($(CD_3)_2SO$, 600MHz) of the compound **12b**

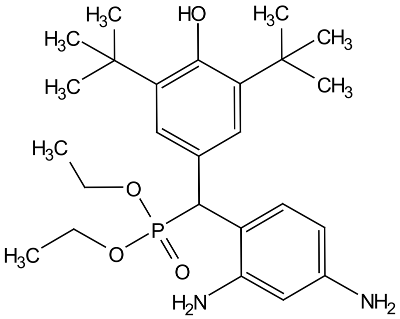

**12b**

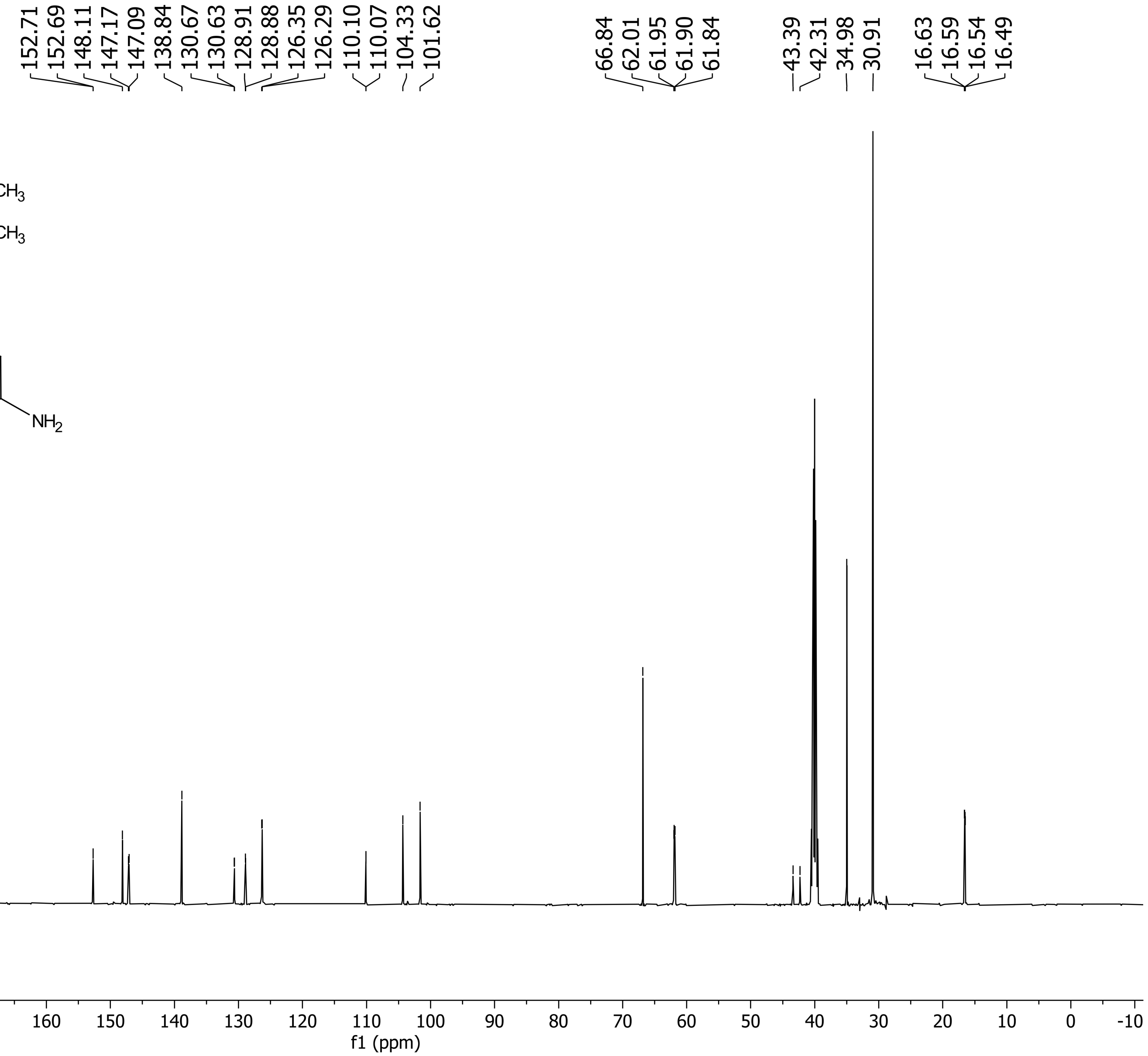


**Figure S37.** $^{13}C\{^{1}H\}$ NMR spectrum ($(CD_3)_2SO$, 101MHz) of the compound **12b**

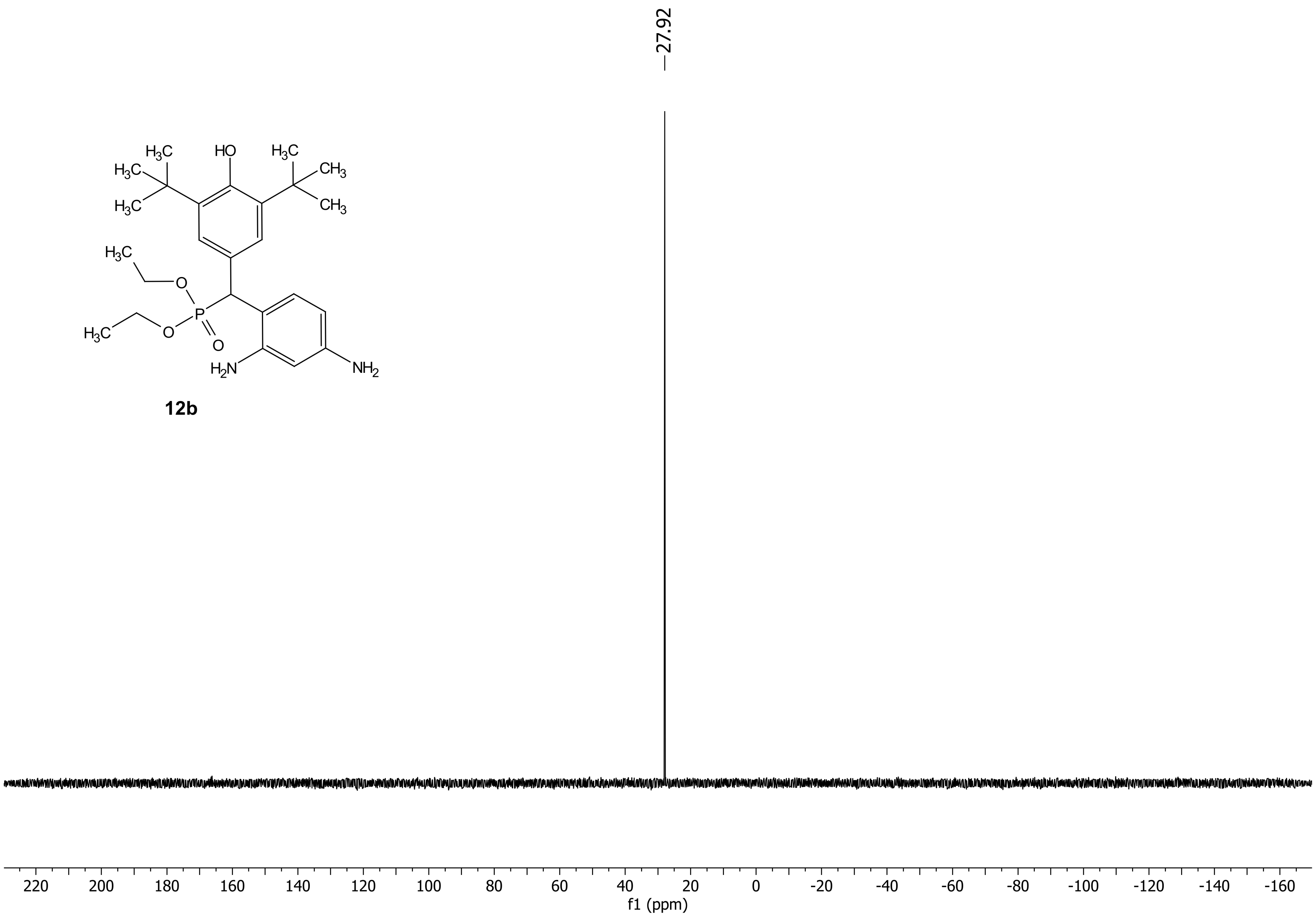


**Figure S38.** $^{31}P\{^{1}H\}$ NMR spectrum ($(CD_3)_2SO$, 243MHz) of the compound **12b**

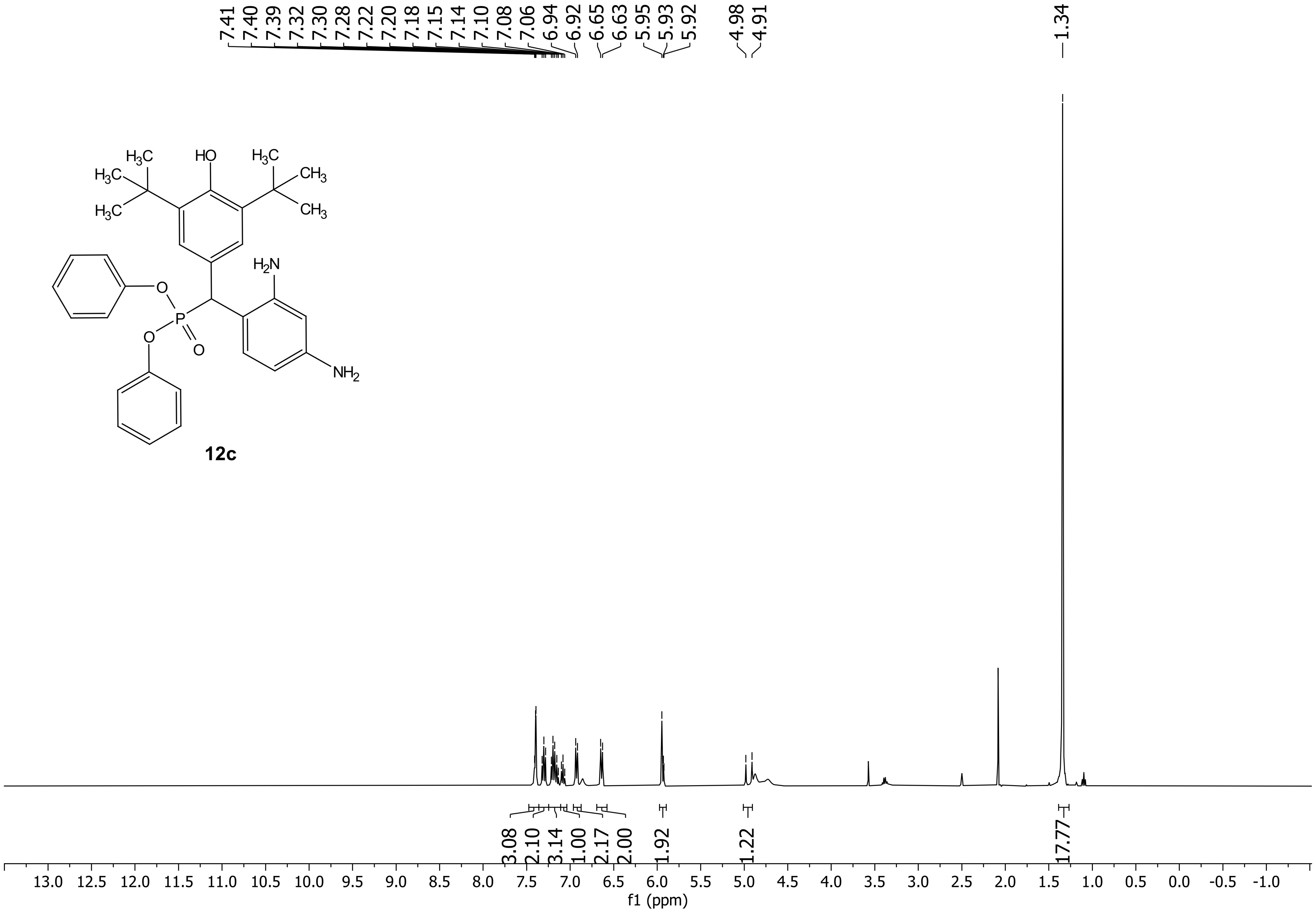


**Figure S39.** $^1H$ NMR spectrum ($(CD_3)_2SO$, 600MHz) of the compound **12c**

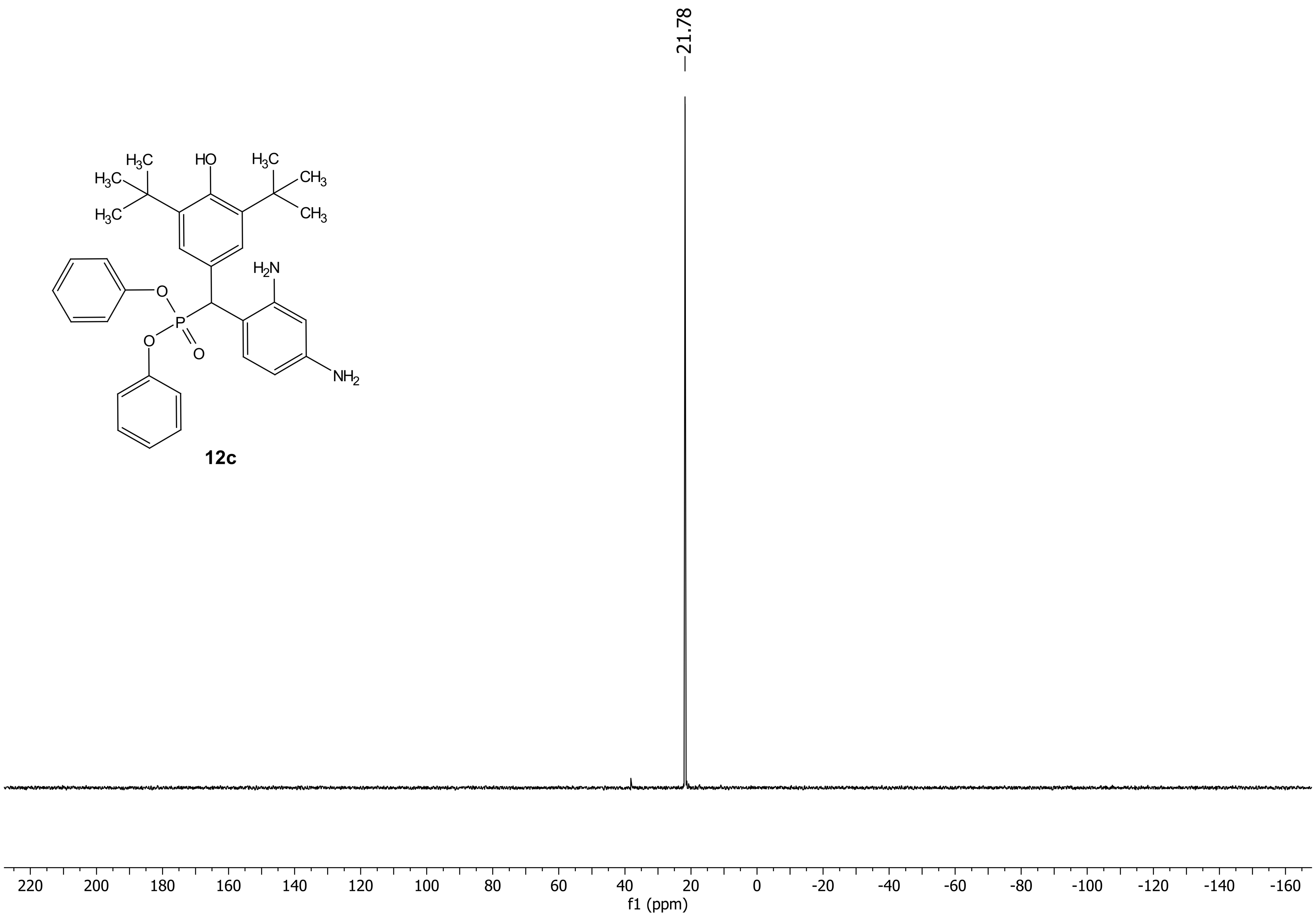


**Figure S40.** $^{31}P\{^1H\}$ NMR spectrum ($(CD_3)_2SO$, 243MHz) of the compound **12c**

# NMR spectra of compounds 3-5, 9.5c,d,f and 9.6c,e

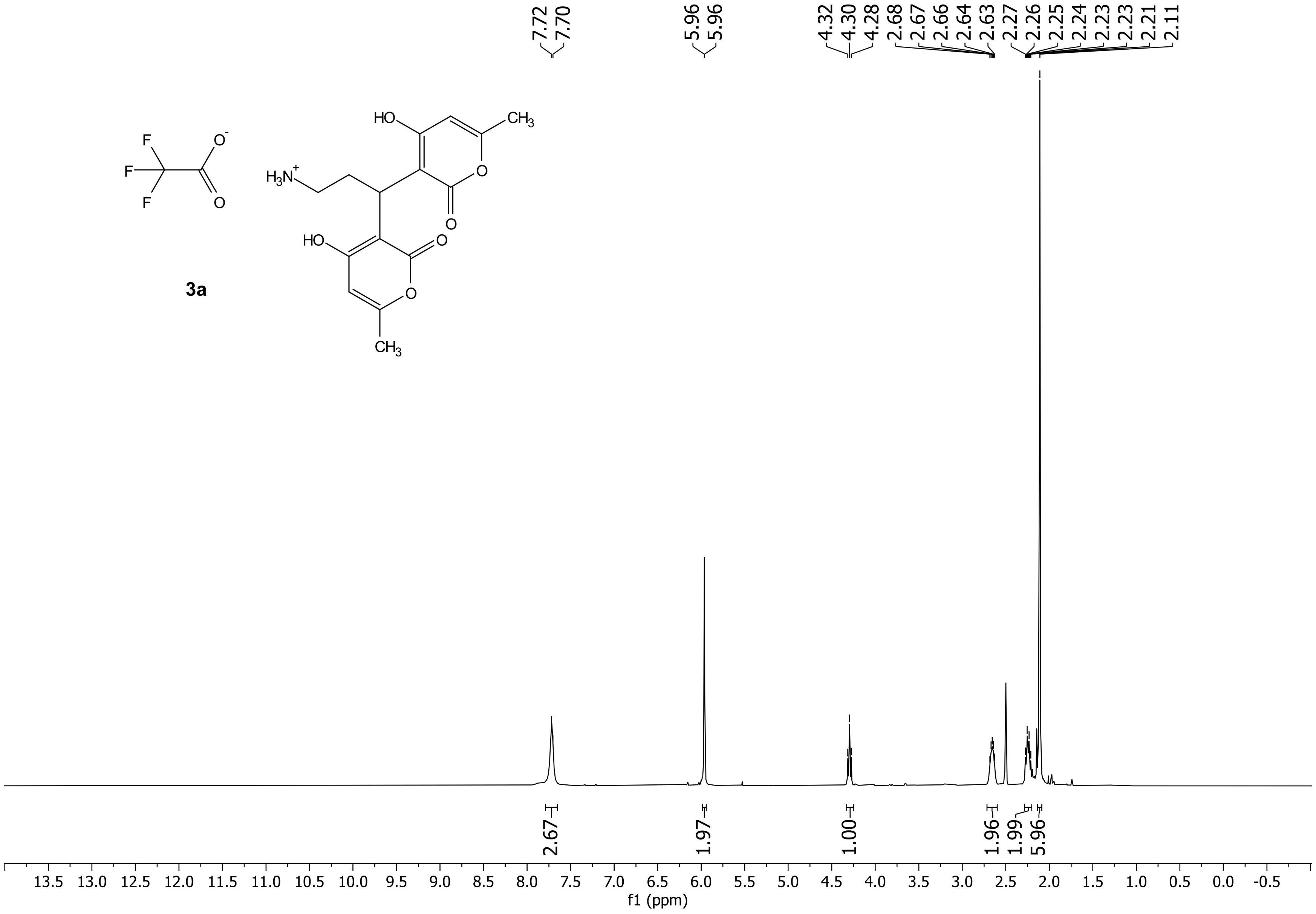


**3a**

**Figure S41.** $^1$H NMR spectrum ($(CD_3)_2SO$, 400MHz) of the compound **3a**

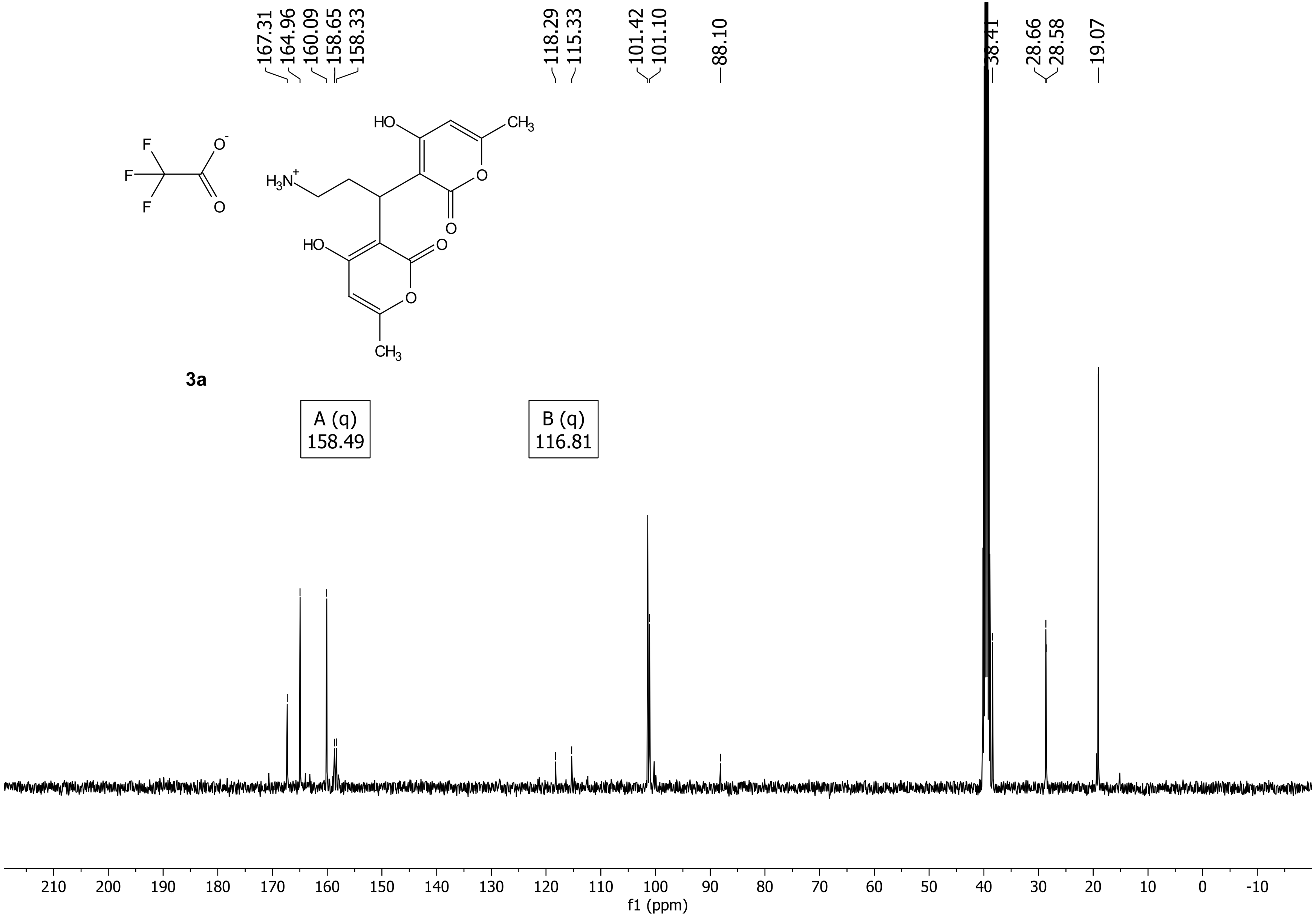


**Figure S42.** $^{13}C\{^1H\}$ NMR spectrum ($(CD_3)_2SO$, 101MHz) of the compound **3a**

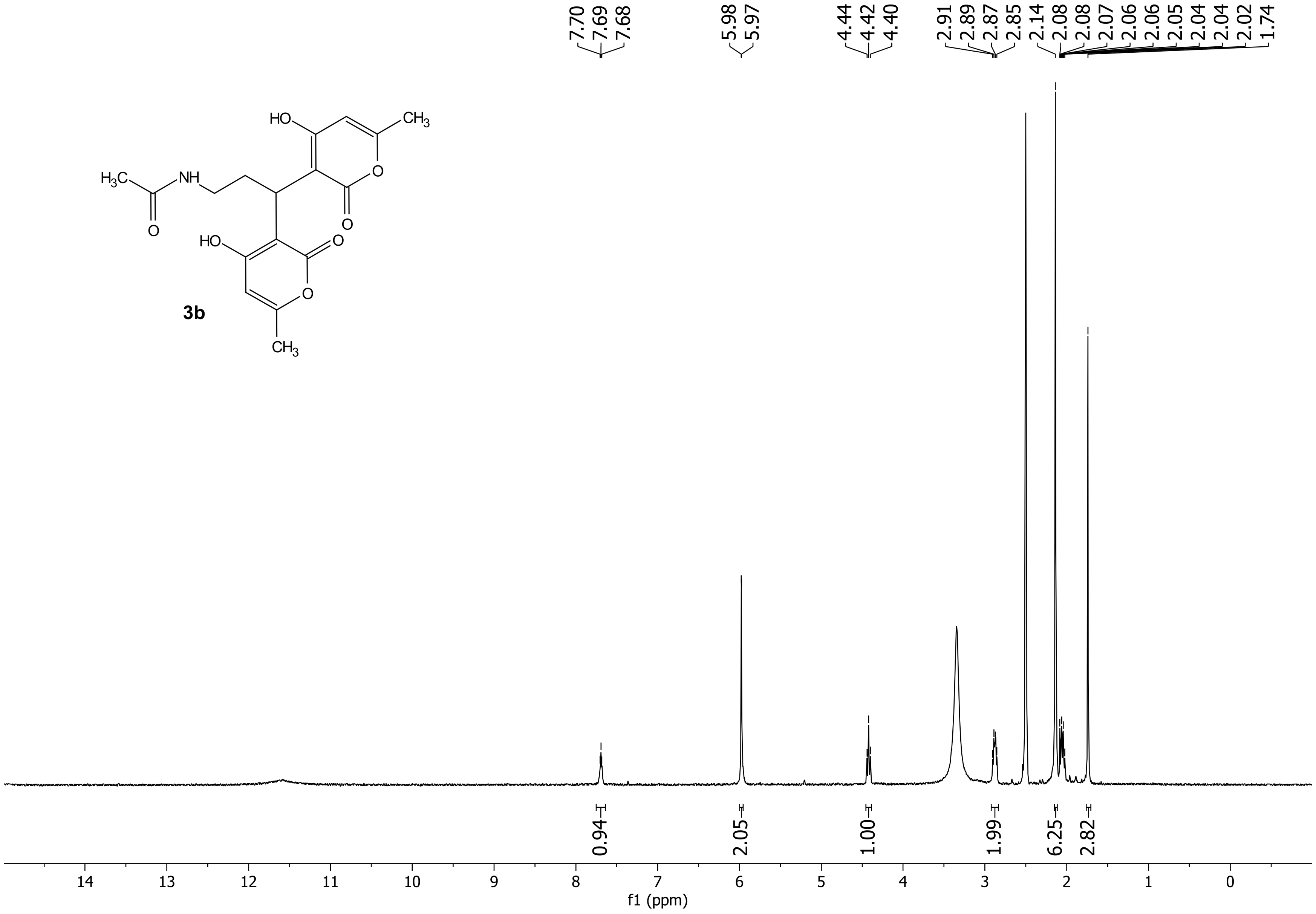


**Figure S43.** $^{1}H$ NMR spectrum ($(CD_3)_2SO$, 400MHz) of the compound **3b**

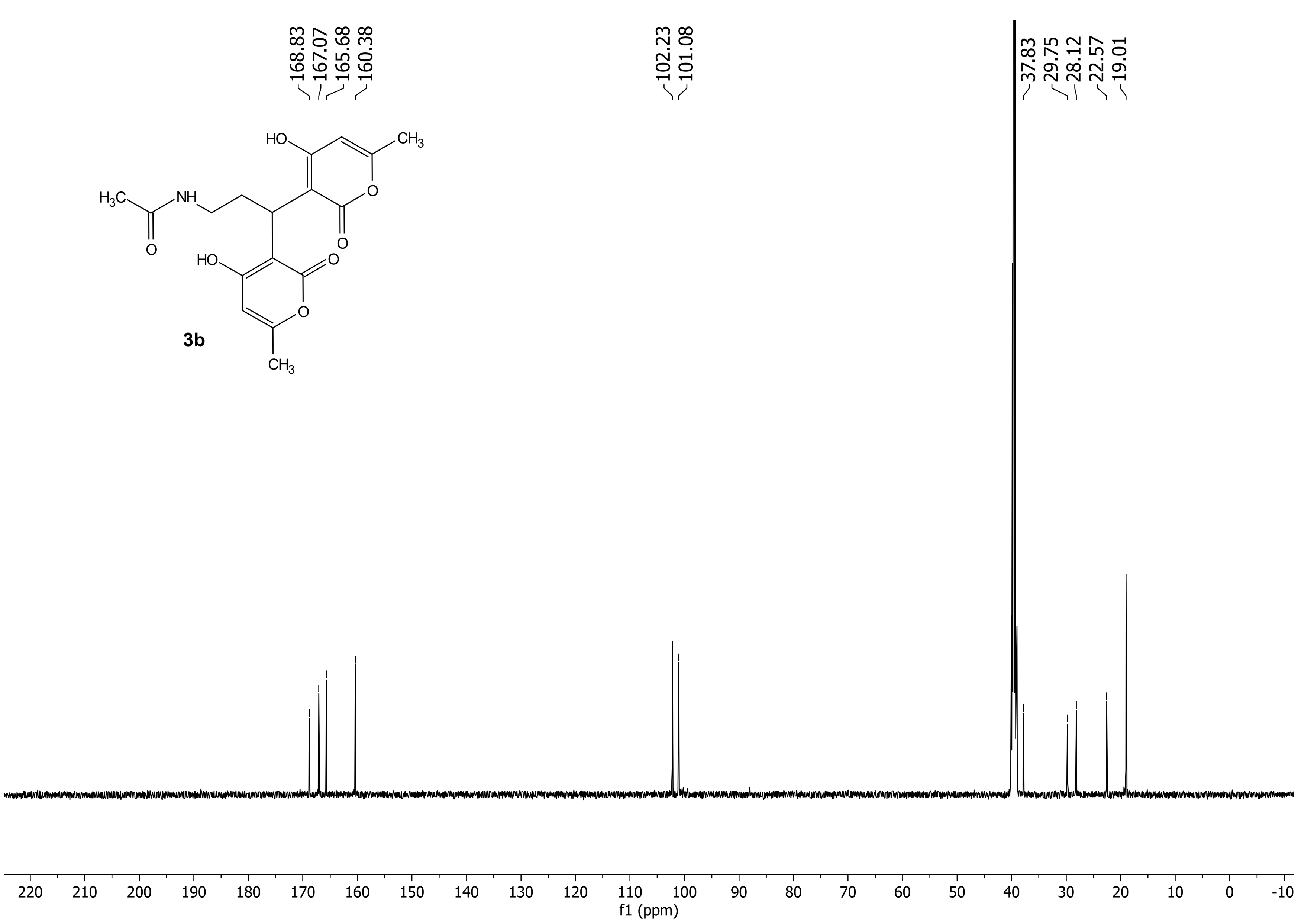


**Figure S44.** $^{13}C\{^{1}H\}$ NMR spectrum ($(CD_3)_2SO$, 126MHz) of the compound **3b**

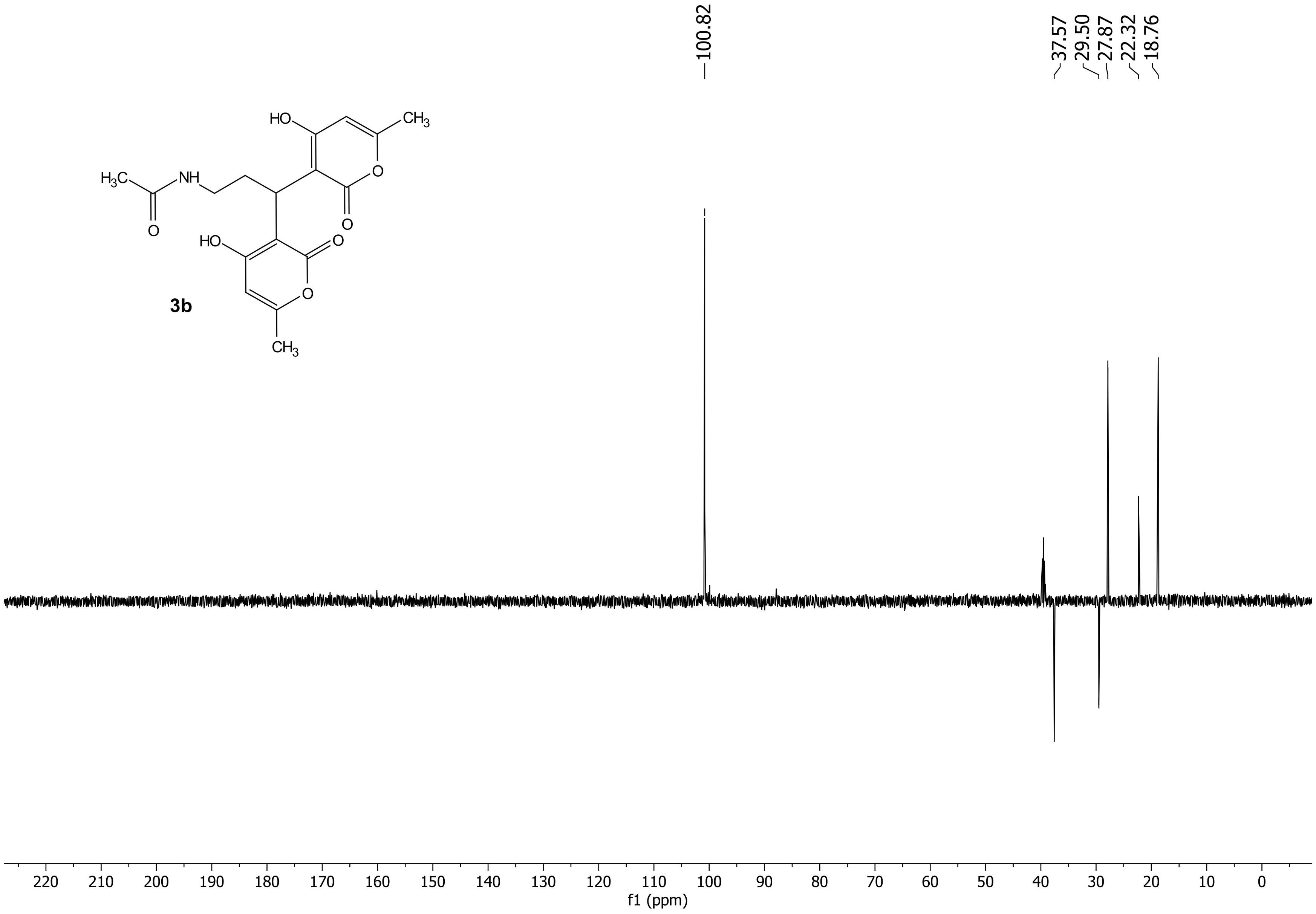


**Figure S45.** DEPT-135 NMR spectrum (126 MHz, $(CD_3)_2SO$) of compound **3b**

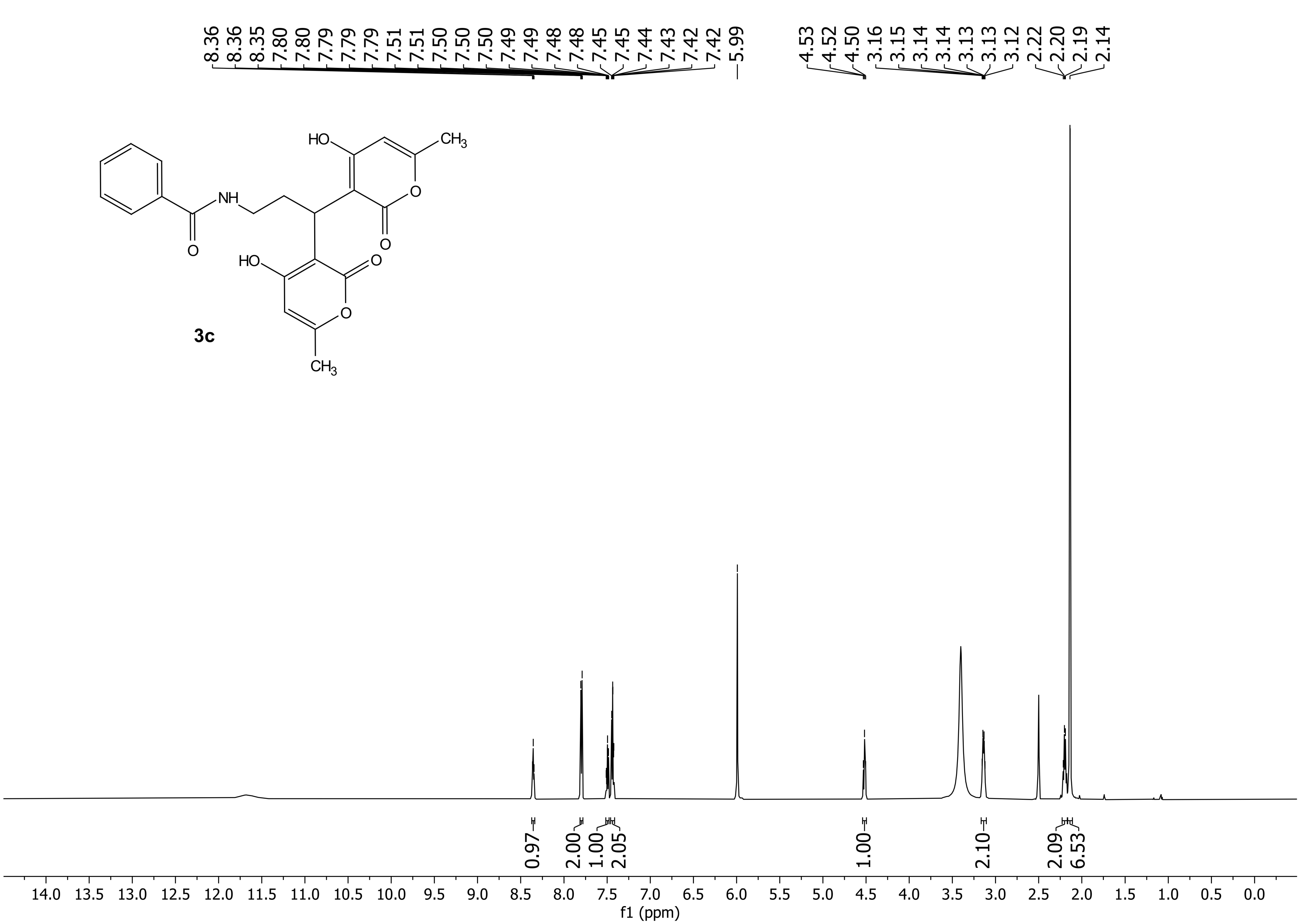


**Figure S46.** $^{1}$H NMR spectrum ($(CD_3)_2SO$, 400MHz) of the compound **3c**

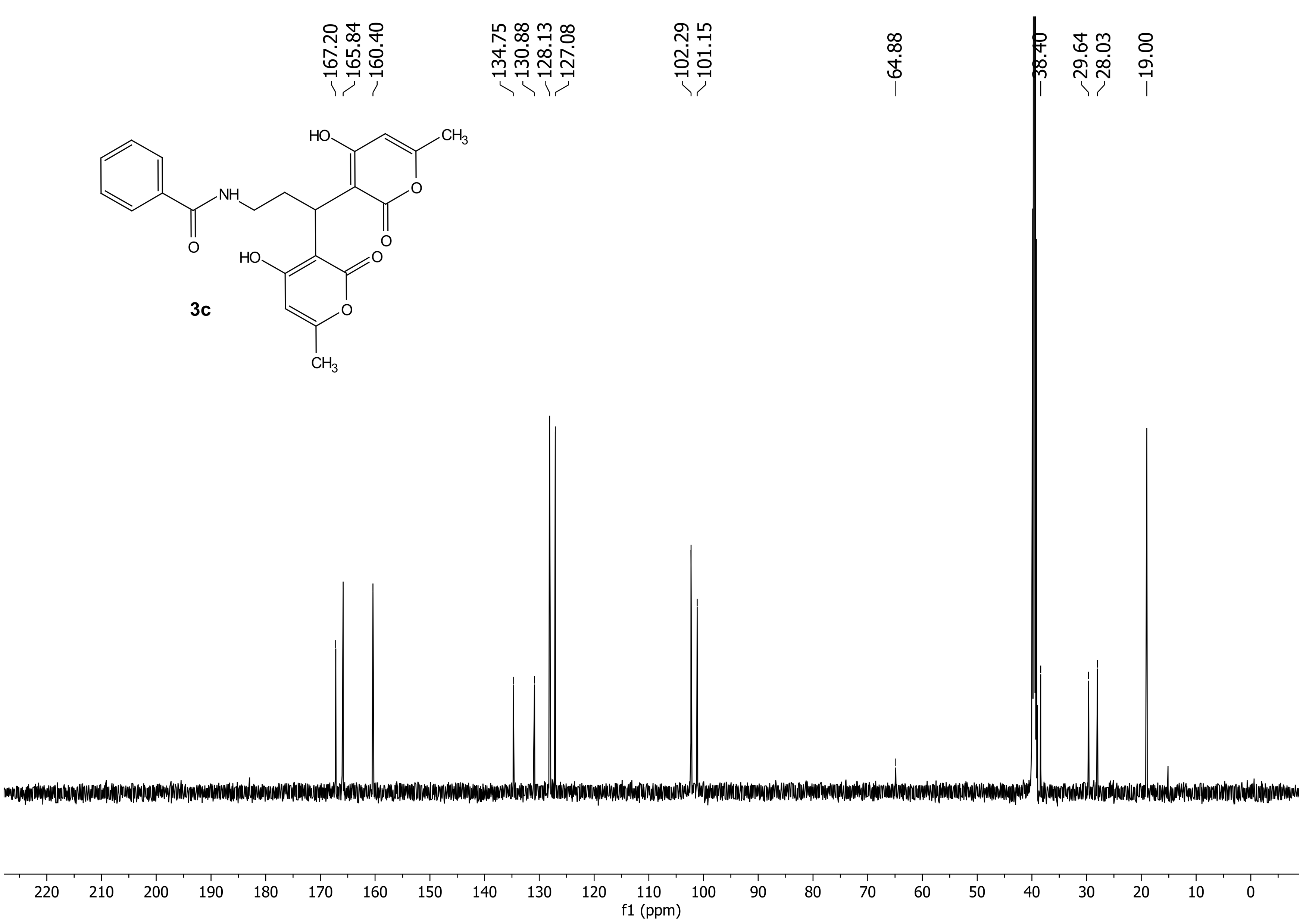


**Figure S47.** $^{13}C\{^{1}H\}$ NMR spectrum ($(CD_3)_2SO$, 126MHz) of the compound **3c**

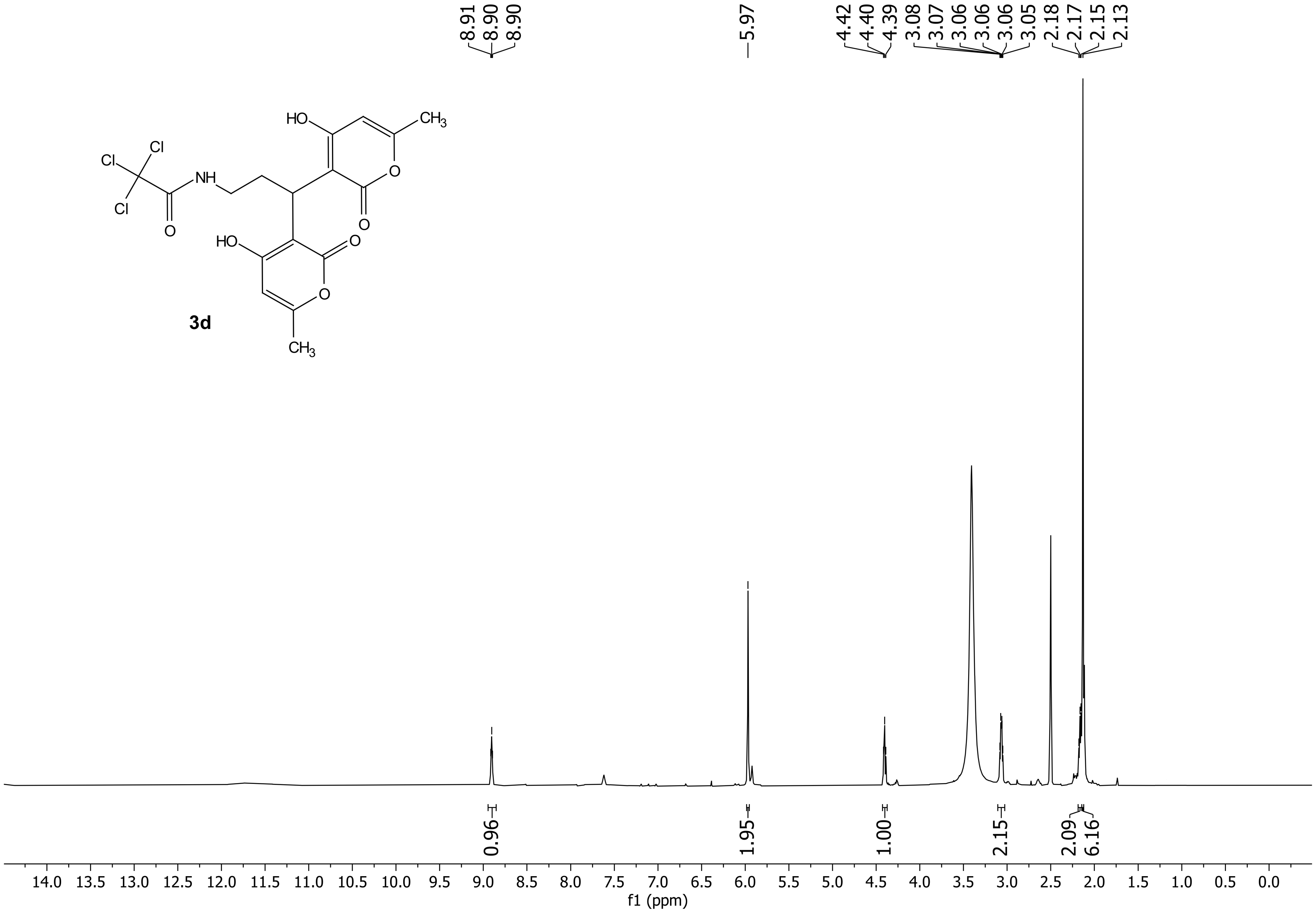


**Figure S48.** $^{1}$H NMR spectrum ($(CD_3)_2SO$, 600MHz) of the compound **3d**

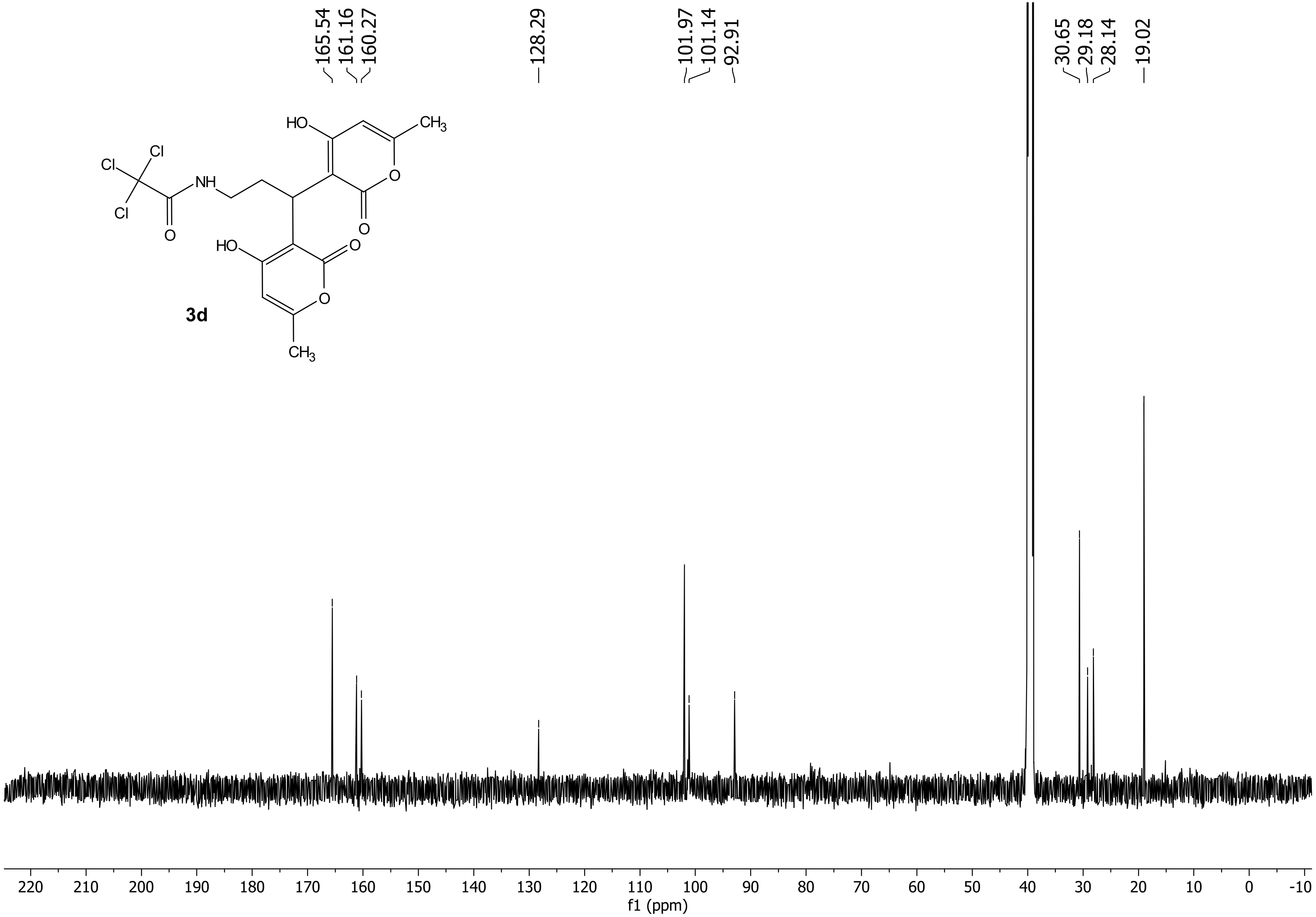


**Figure S49.** $^{13}C\{^{1}H\}$ NMR spectrum ($(CD_3)_2SO$, 126MHz) of the compound **3d**

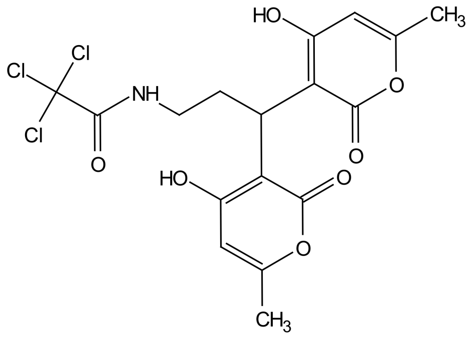

3d

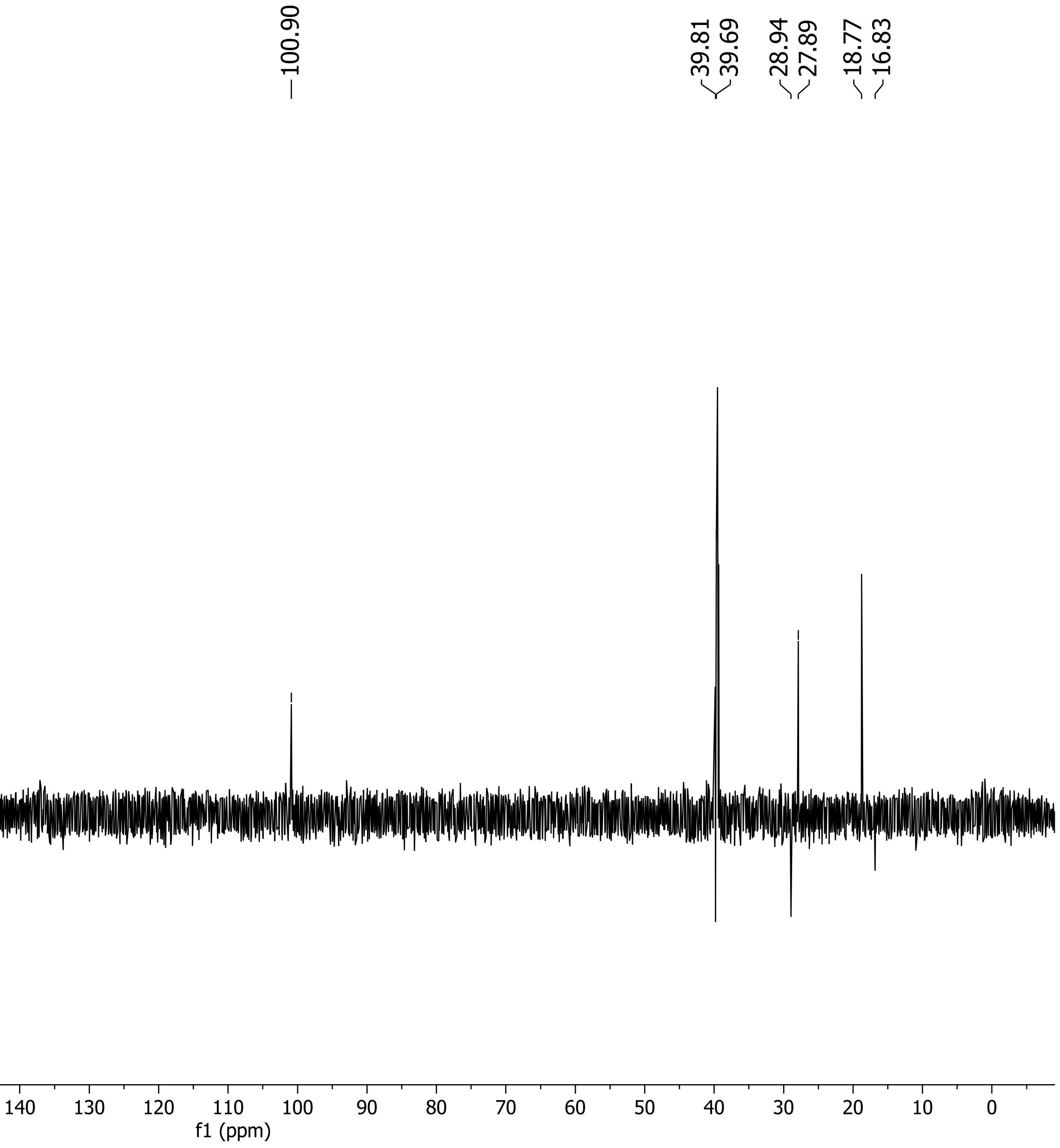


**Figure S50.** DEPT-135 NMR spectrum (126 MHz, $(CD_3)_2SO$) of compound **3d**

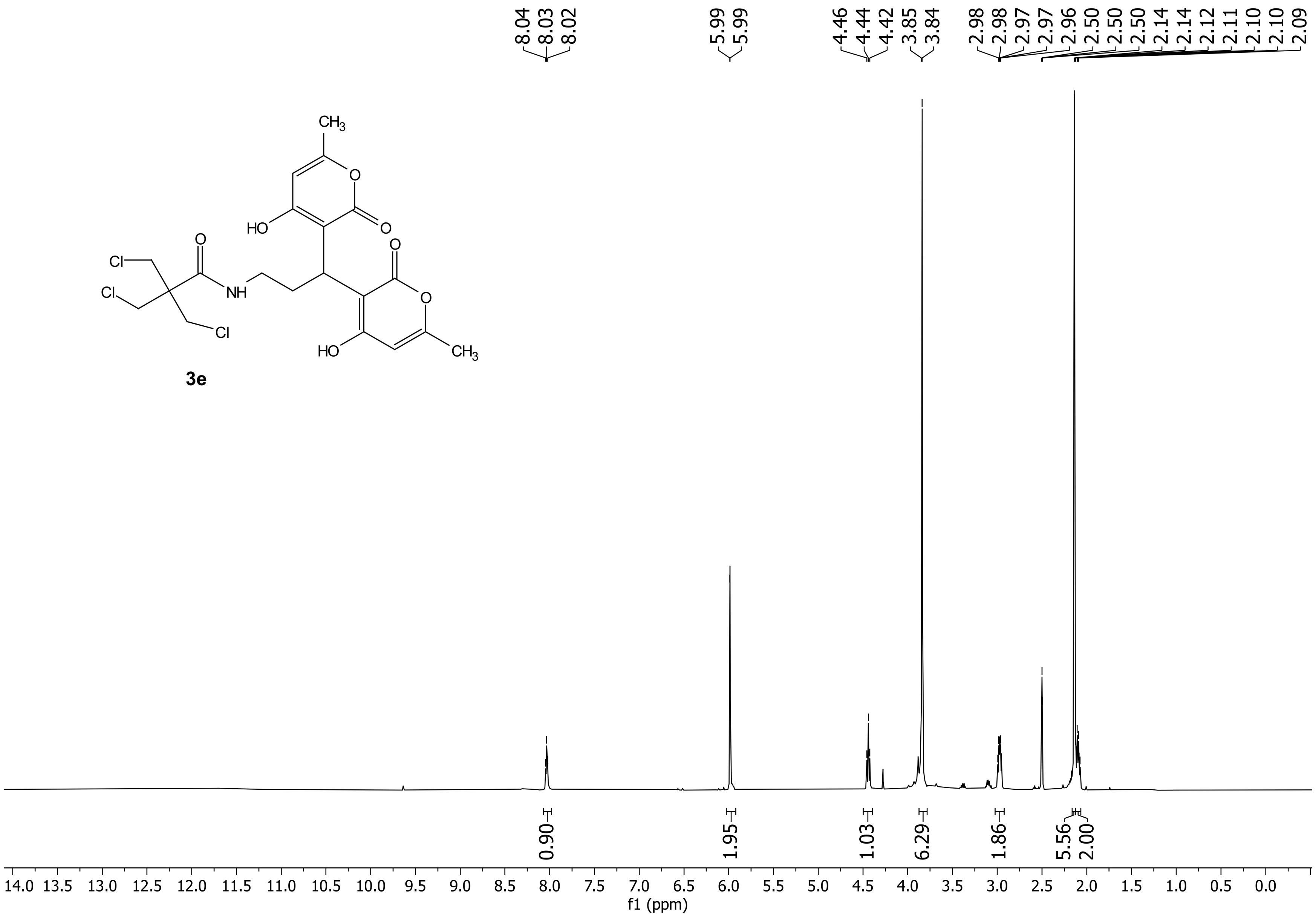


**Figure S51.** $^{1}$H NMR spectrum ($(CD_3)_2SO$, 500MHz) of the compound **3e**

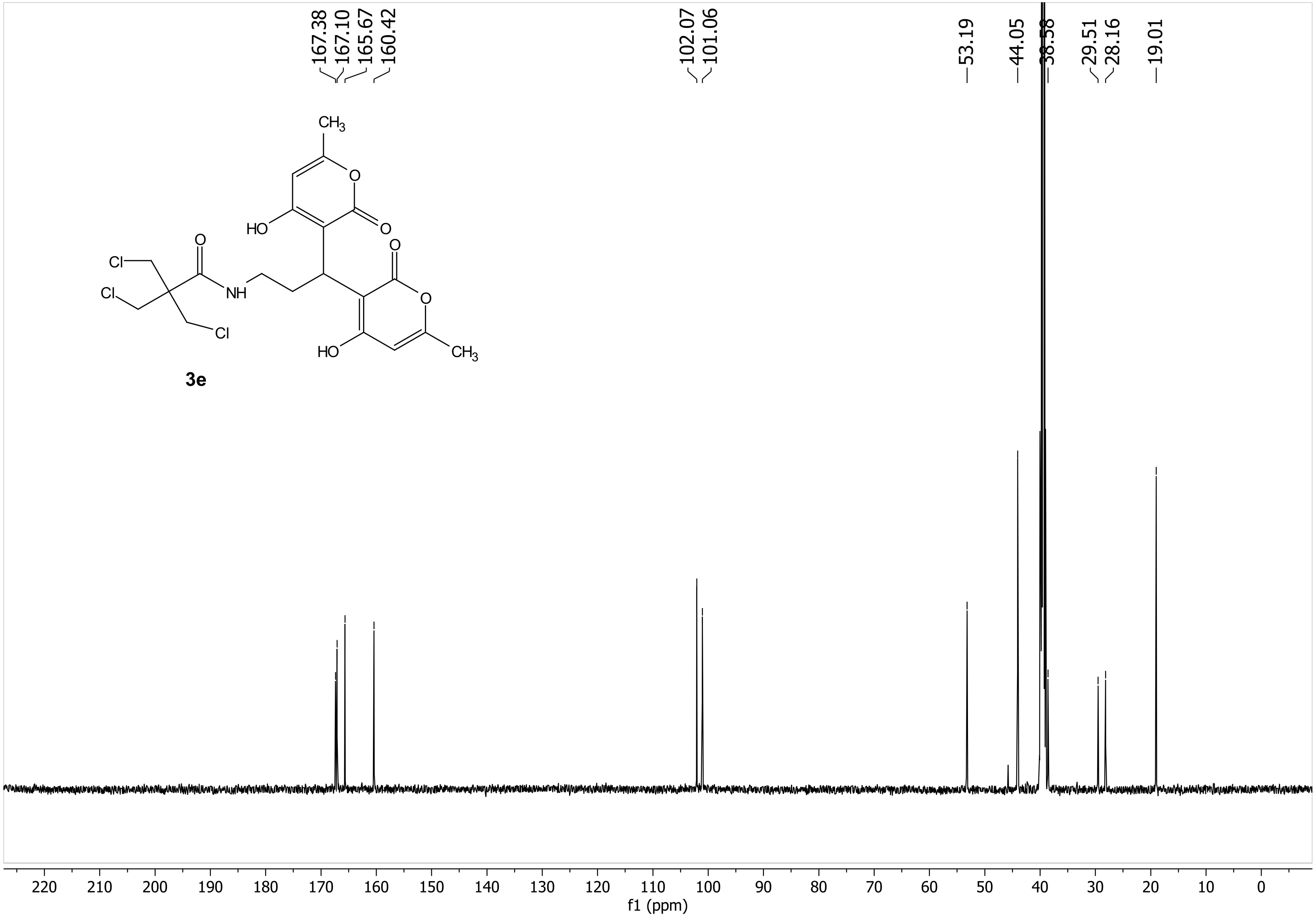


**Figure S52.** $^{13}C\{^{1}H\}$ NMR spectrum ($(CD_3)_2SO$, 126MHz) of the compound **3e**

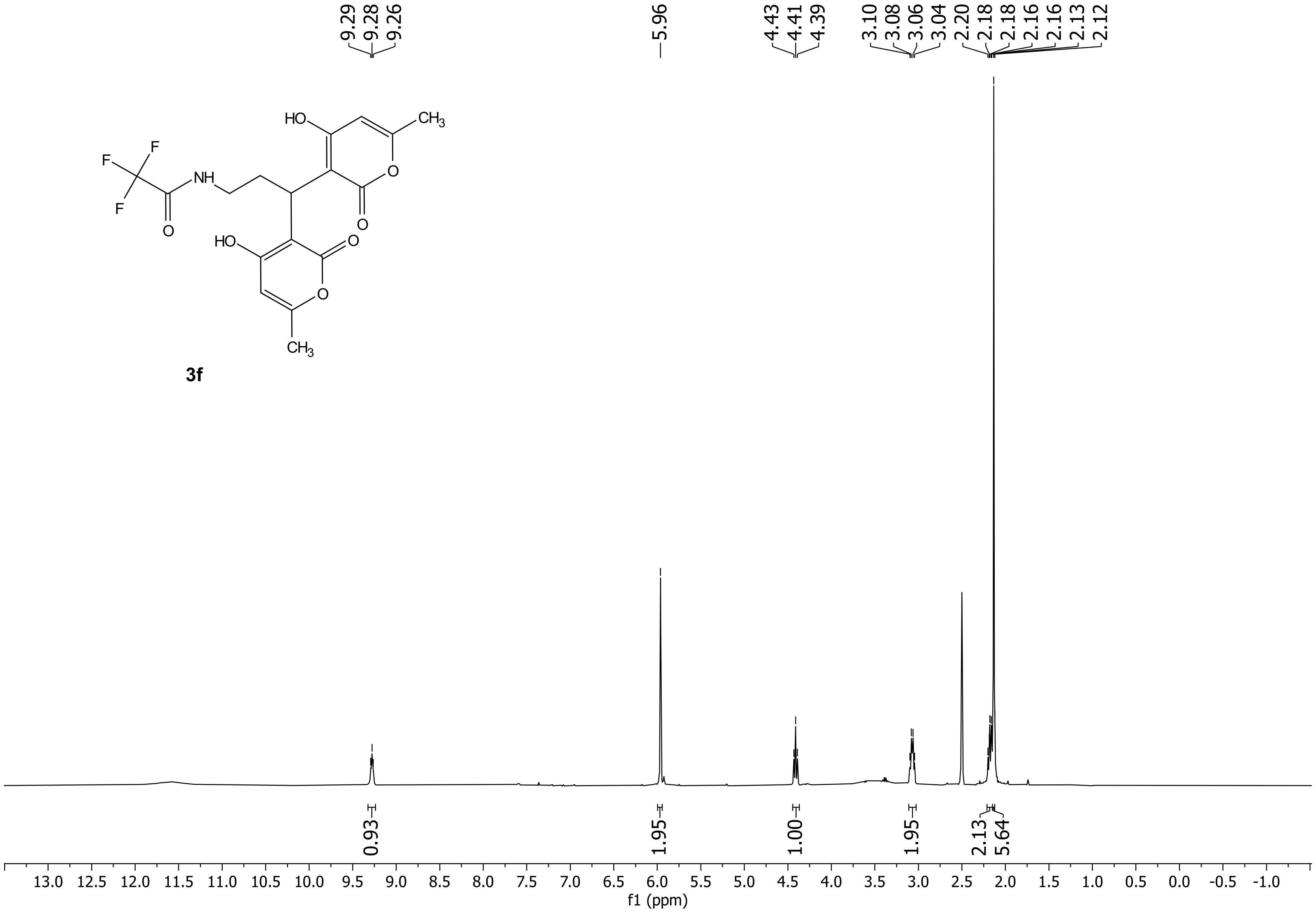


**Figure S53.** $^{1}H$ NMR spectrum ($(CD_3)_2SO$, 400MHz) of the compound **3f**

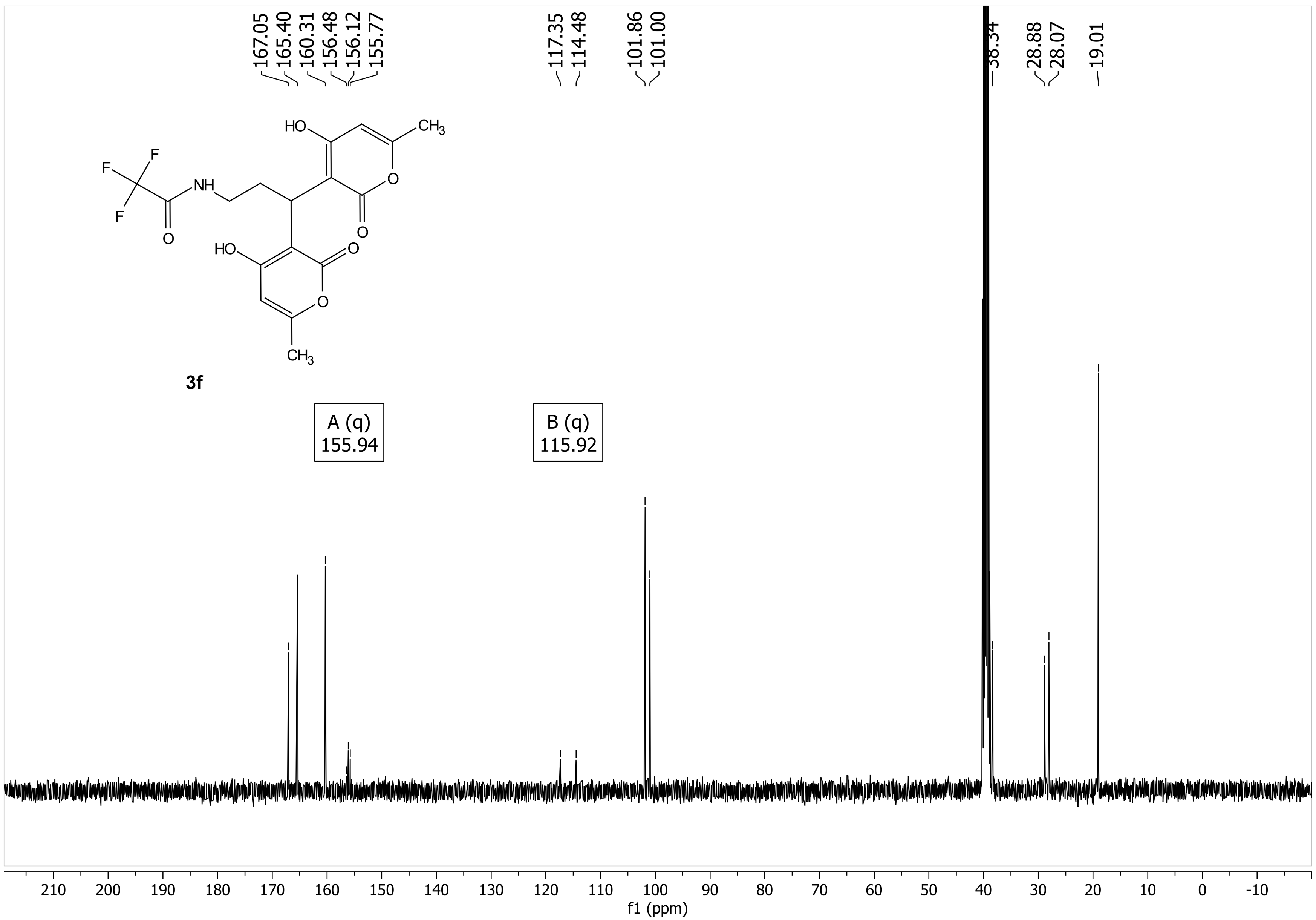


**Figure S54.** $^{13}C\{^1H\}$ NMR spectrum ($(CD_3)_2SO$, 101MHz) of the compound **3f**

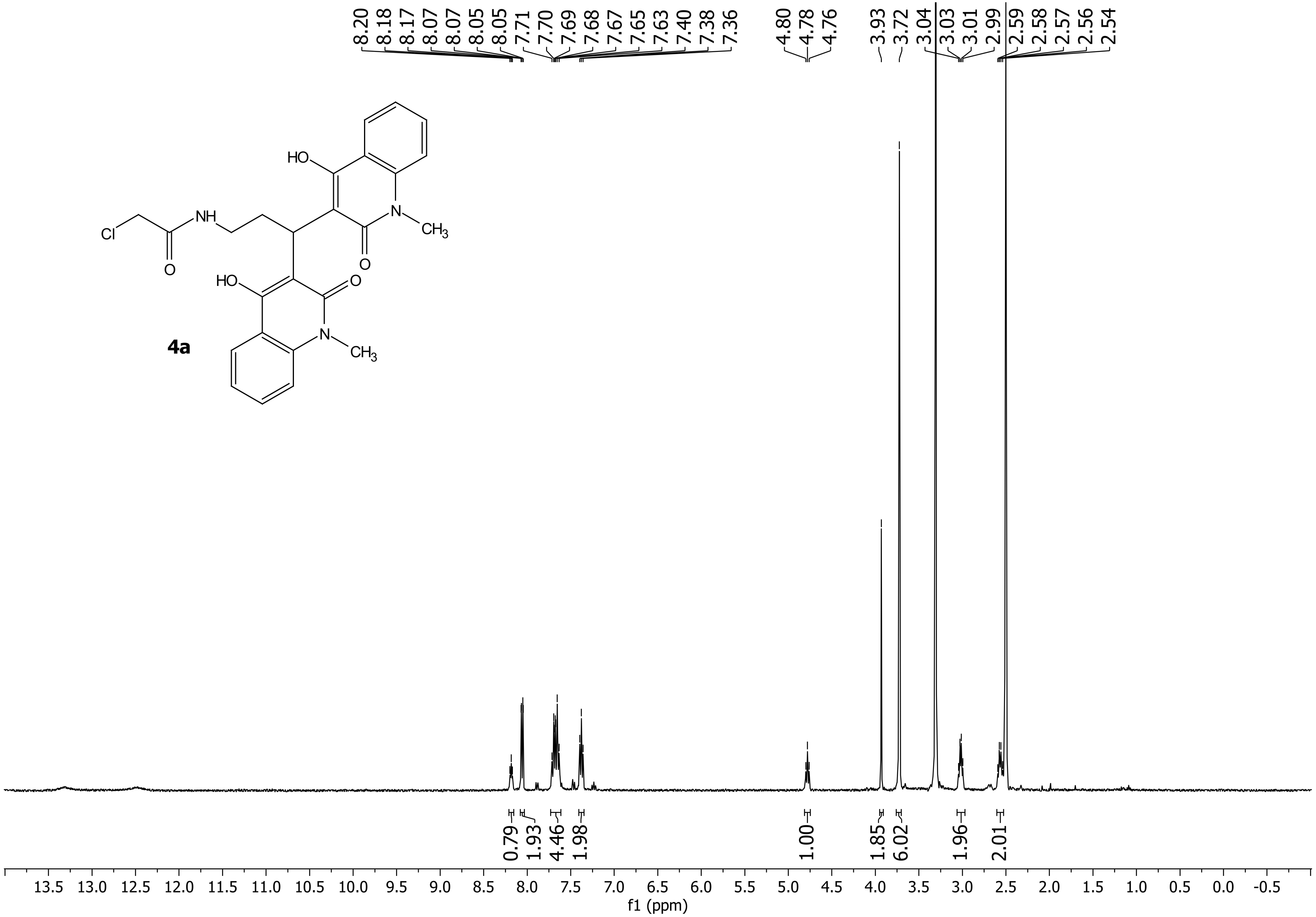


**Figure S55.** $^{1}$H NMR spectrum ($(CD_3)_2SO$, 400MHz) of the compound **4a**

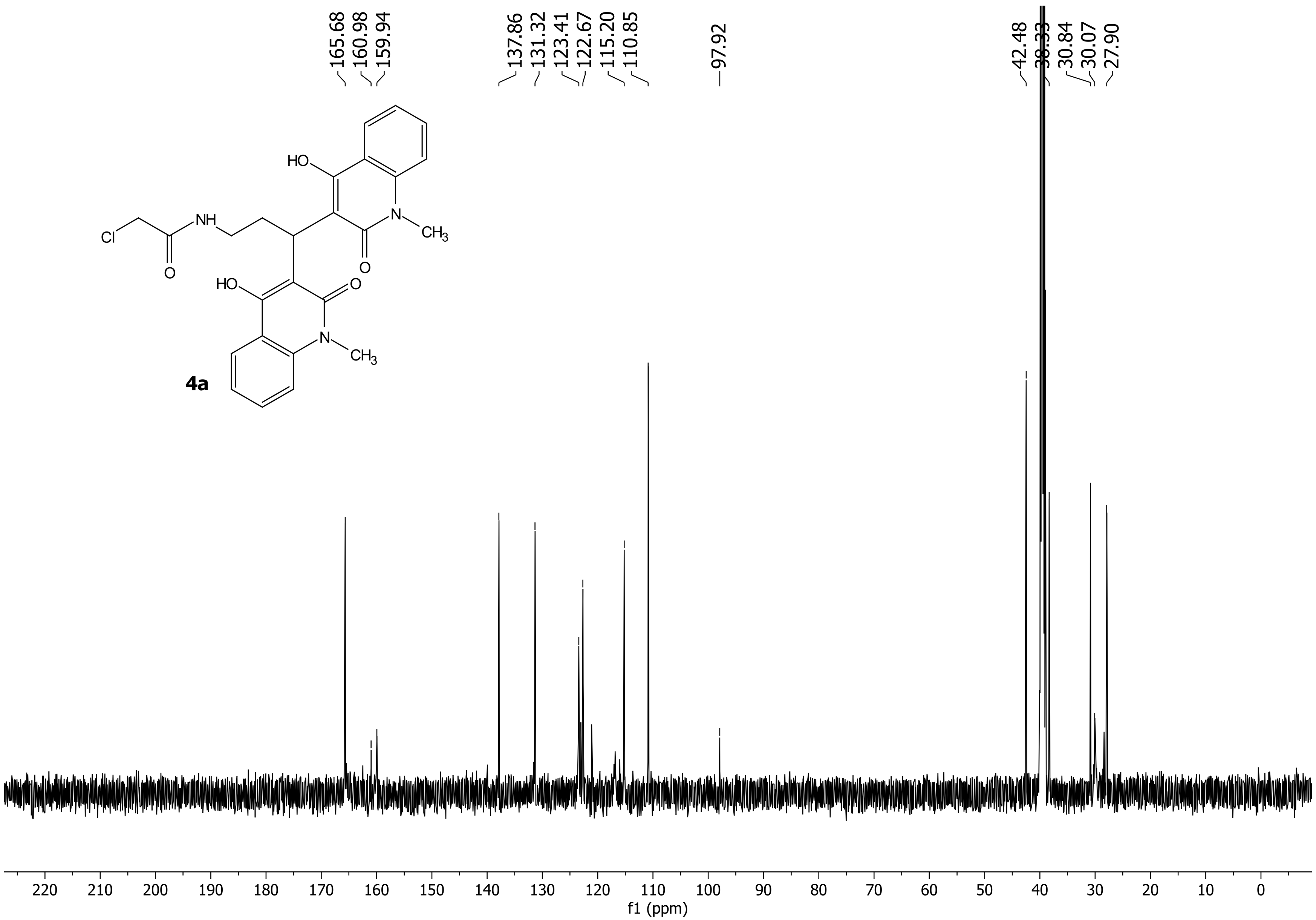


**Figure S56.** $^{13}C\{^{1}H\}$ NMR spectrum ($(CD_3)_2SO$, 126MHz) of the compound **4a**

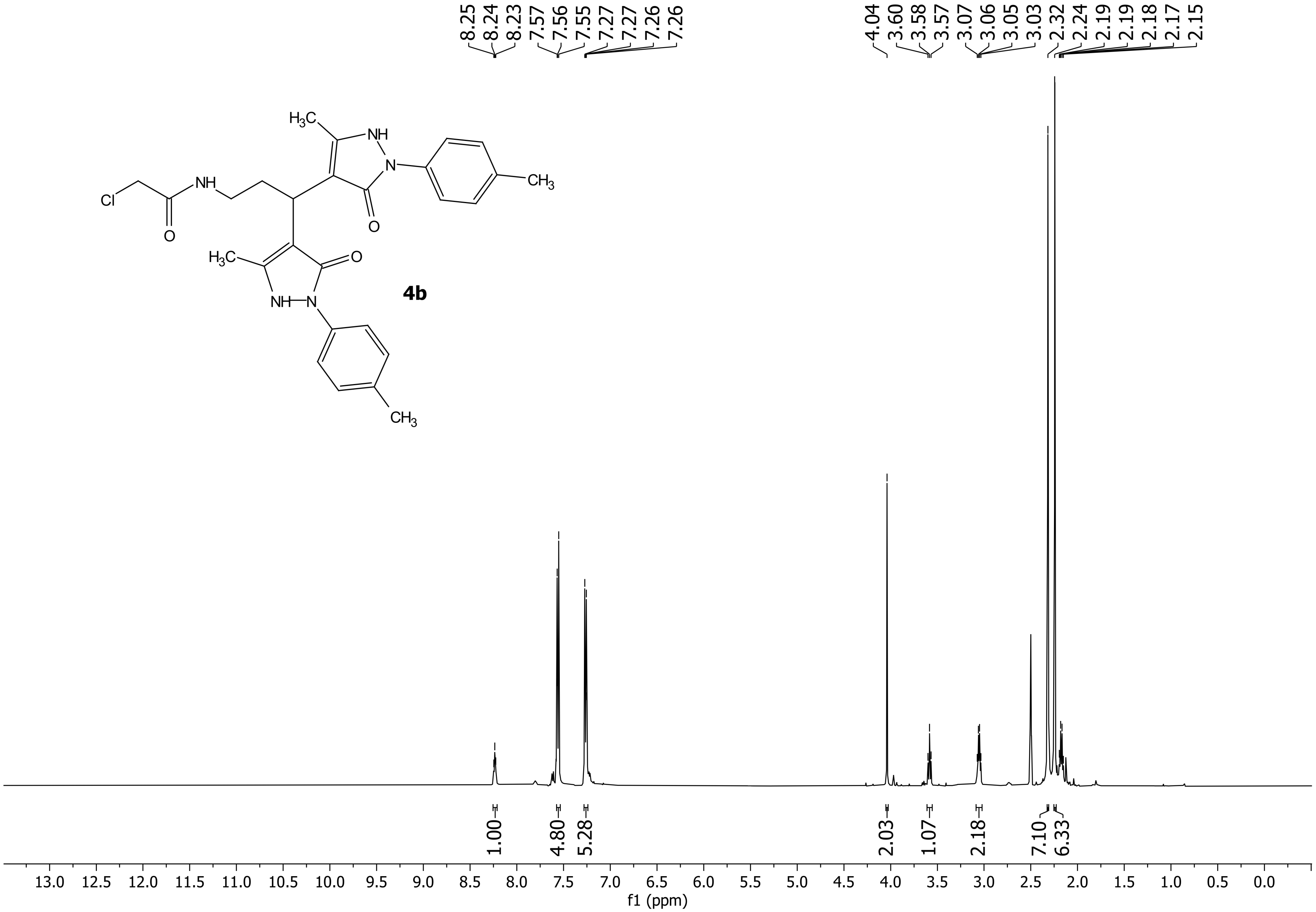


**4b**

**Figure S57.** $^{1}H$ NMR spectrum ($(CD_3)_2SO$, 500MHz) of the compound **4b**

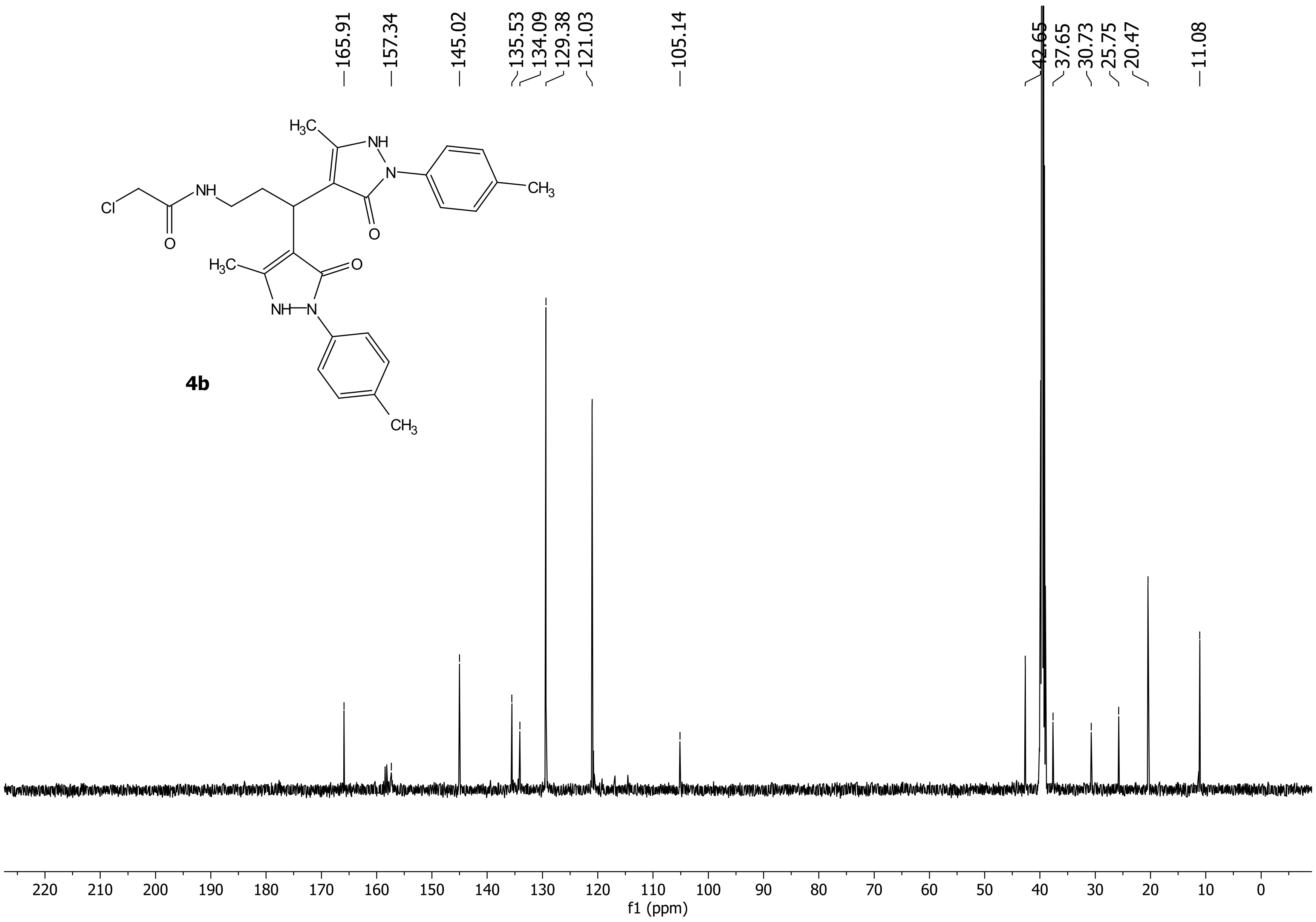


**Figure S58.** $^{13}C\{^{1}H\}$ NMR spectrum ($(CD_3)_2SO$, 126MHz) of the compound **4b**

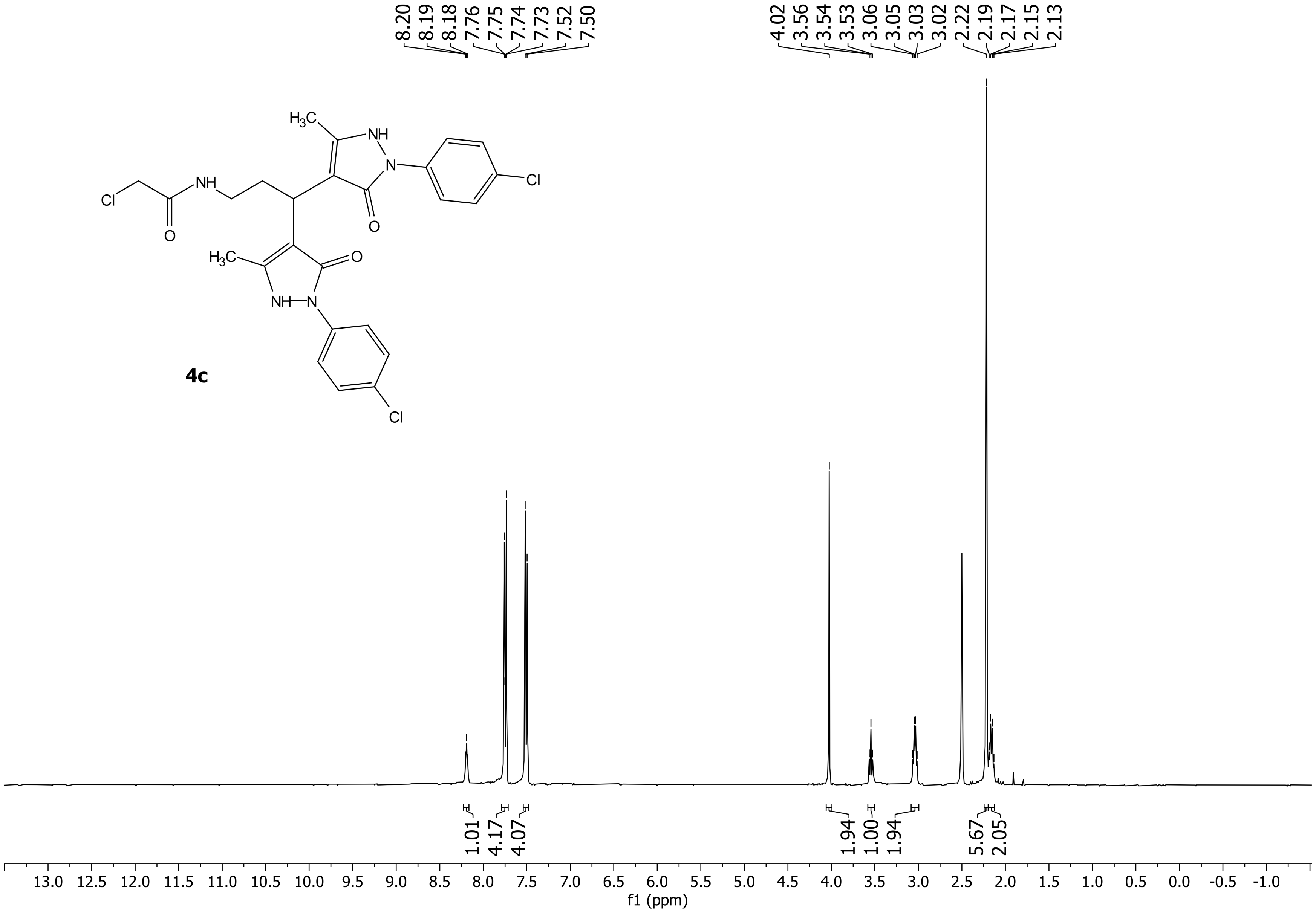


**Figure S59.** $^1H$ NMR spectrum ($(CD_3)_2SO$, 400MHz) of the compound **4c**

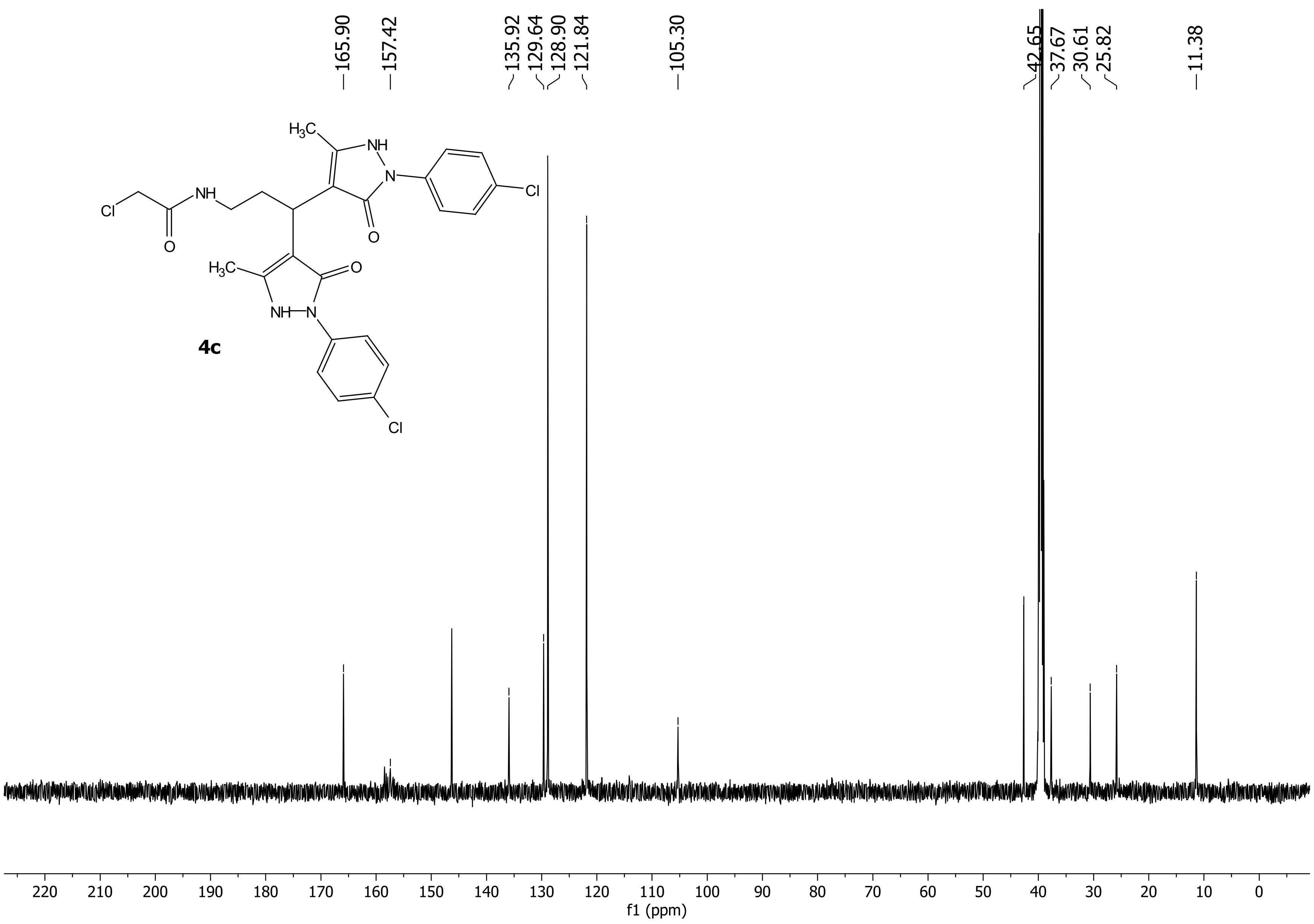


**Figure S60.** $^{13}C\{^{1}H\}$ NMR spectrum ($(CD_3)_2SO$, 126MHz) of the compound **4c**

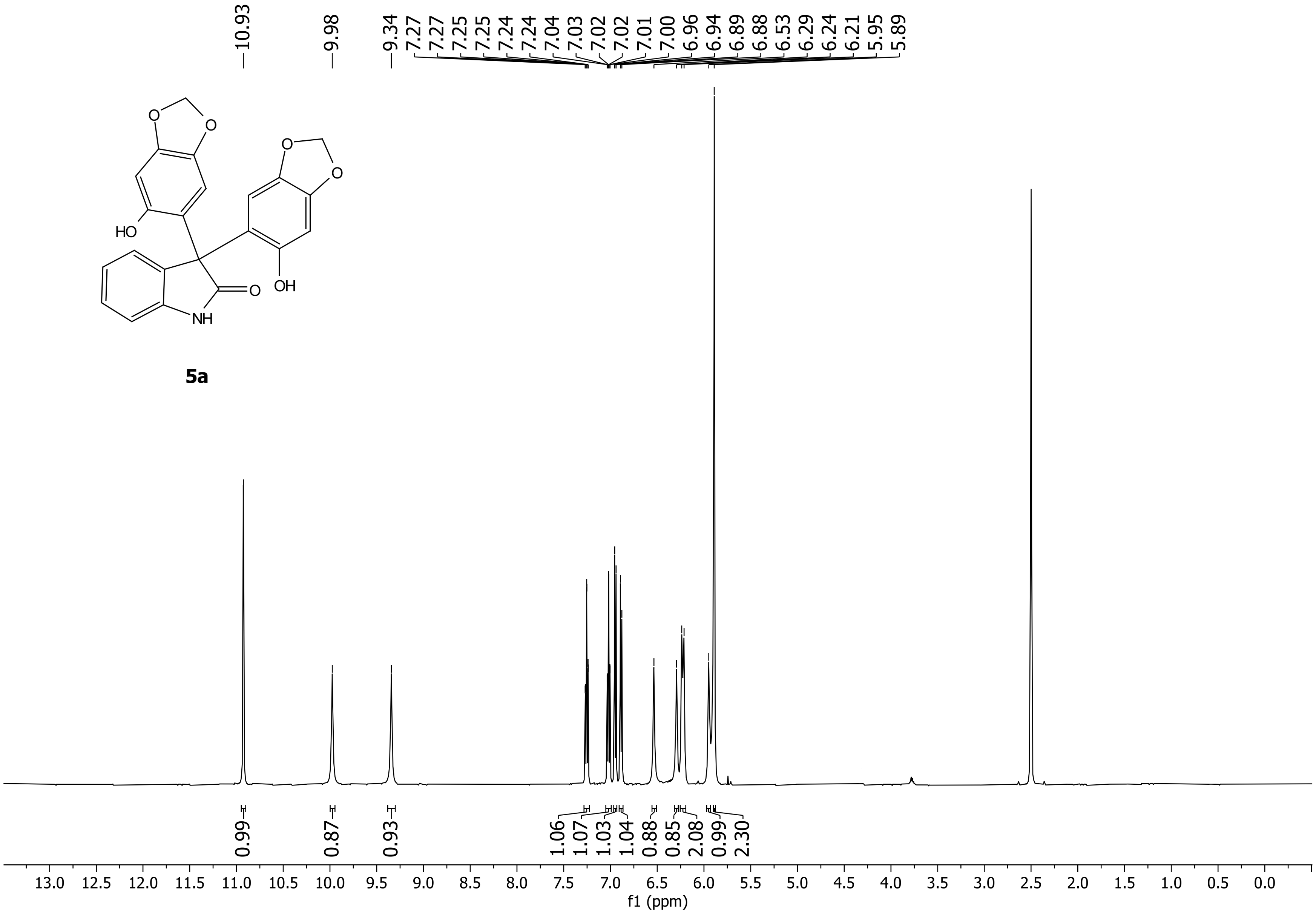


**Figure S61.** $^{1}$H NMR spectrum ($(CD_3)_2SO$, 500MHz) of the compound **5a**

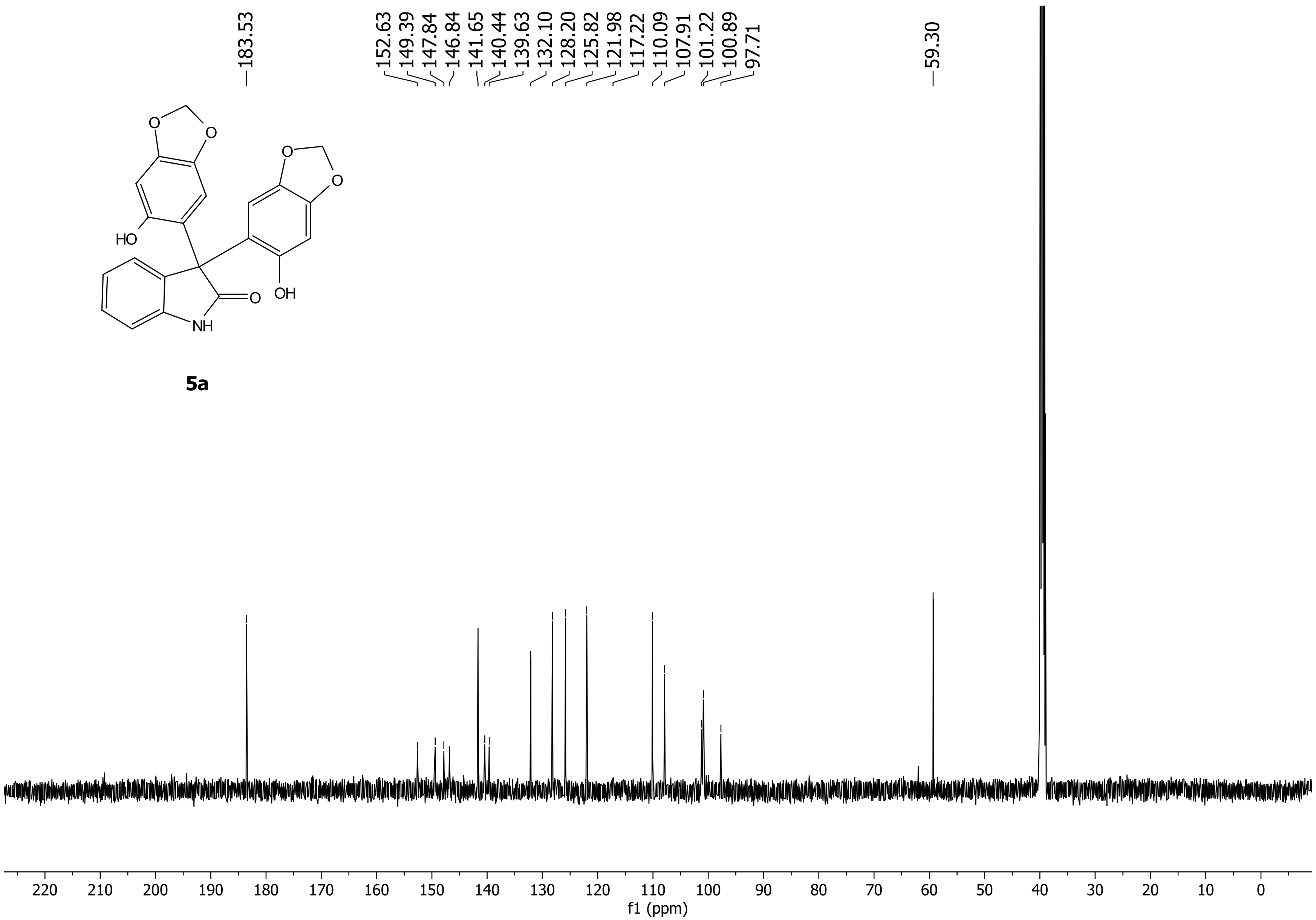


**Figure S62.** $^{13}C\{^1H\}$ NMR spectrum ($(CD_3)_2SO$, 126MHz) of the compound **5a**

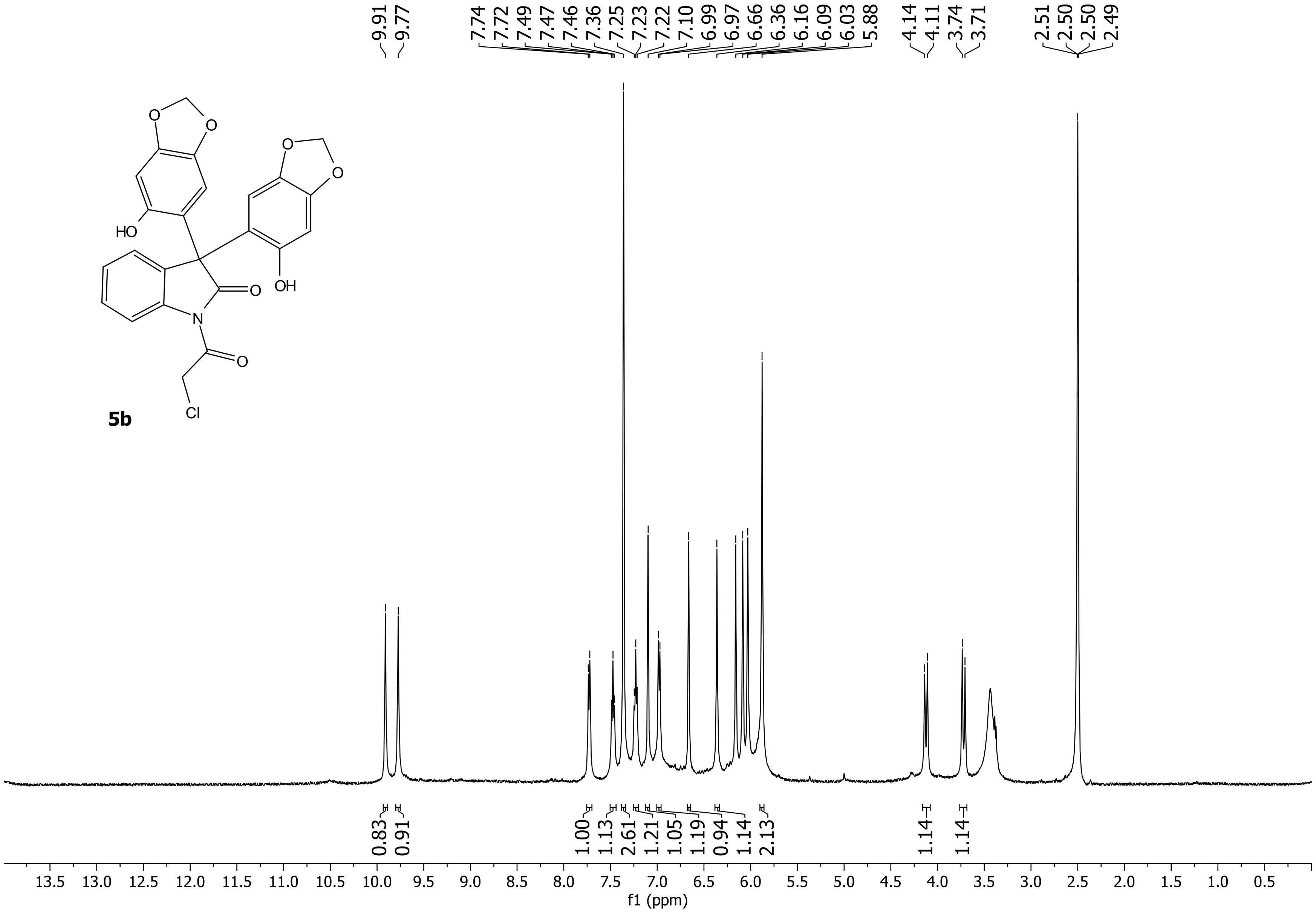


**Figure S63.** $^{1}$H NMR spectrum ($(CD_3)_2SO$, 500MHz) of the compound **5b**

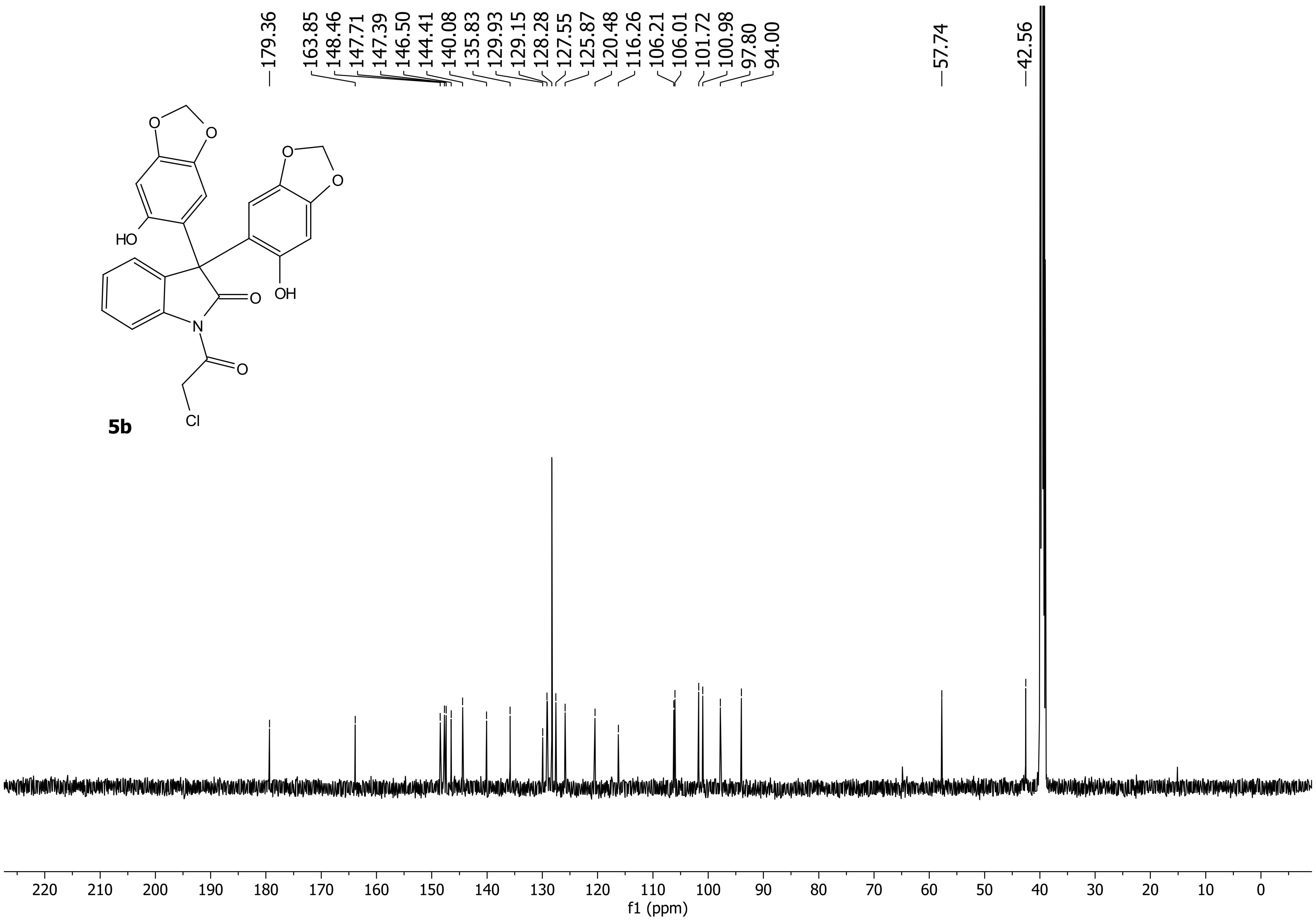


**Figure S64.** $^{13}C\{^{1}H\}$ NMR spectrum ($(CD_3)_2SO$, 126MHz) of the compound **5b**

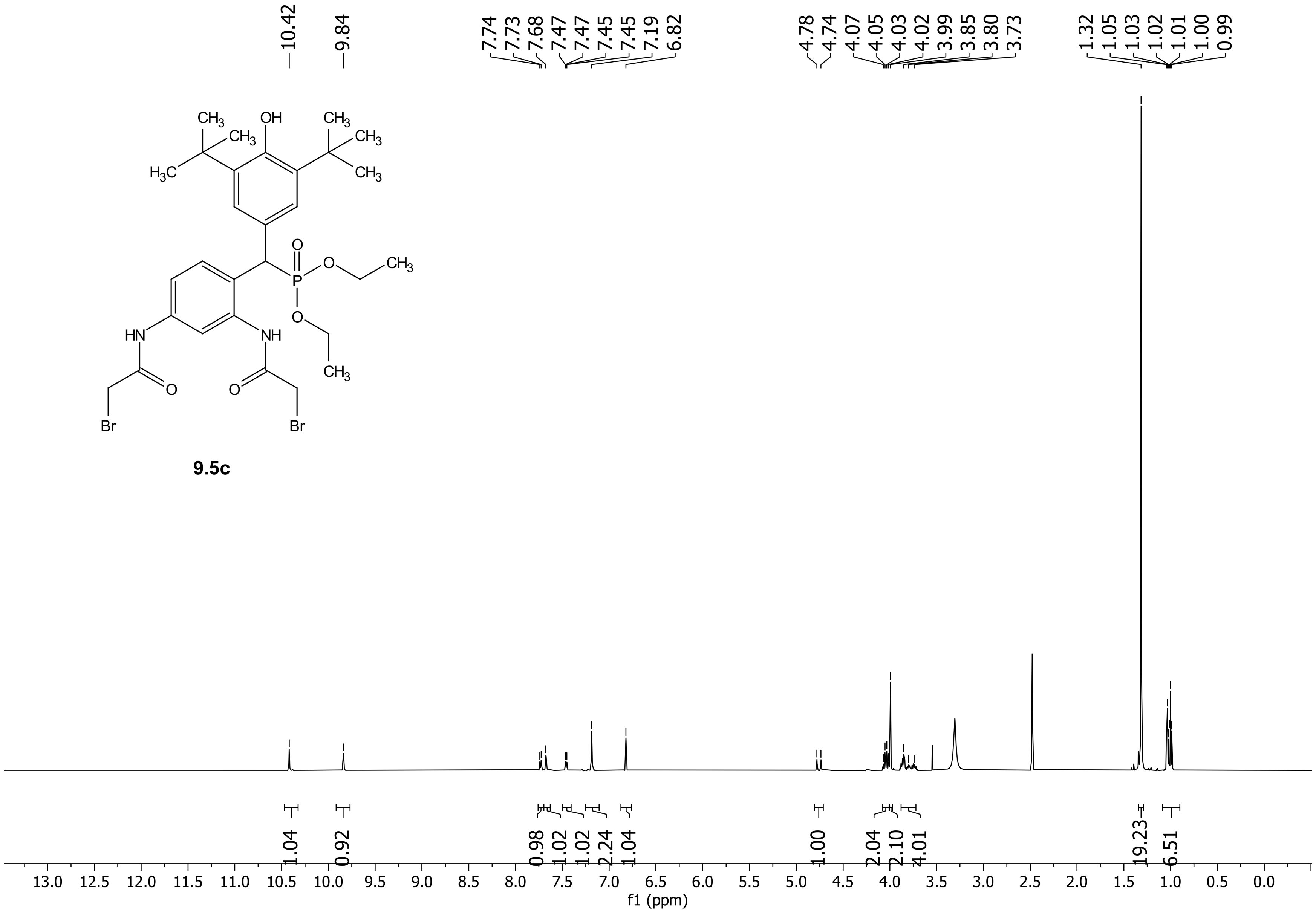


**Figure S65.** $^{1}H$ NMR spectrum ($(CD_3)_2SO$, 600MHz) of the compound **9.5c**

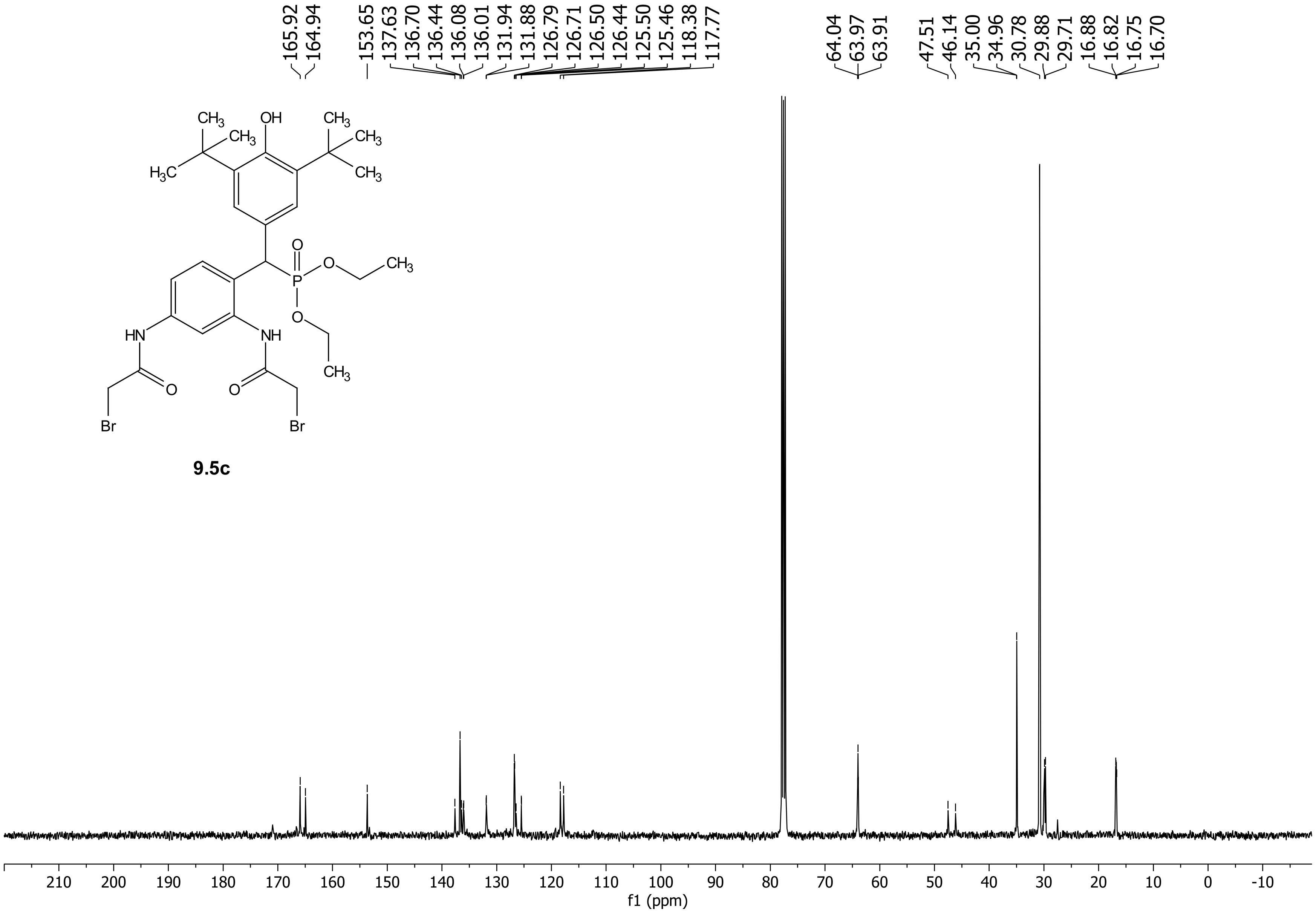


**Figure S66.** $^{13}C\{^{1}H\}$ NMR spectrum ($(CD_3)_2SO$, 101MHz) of the compound **9.5c**

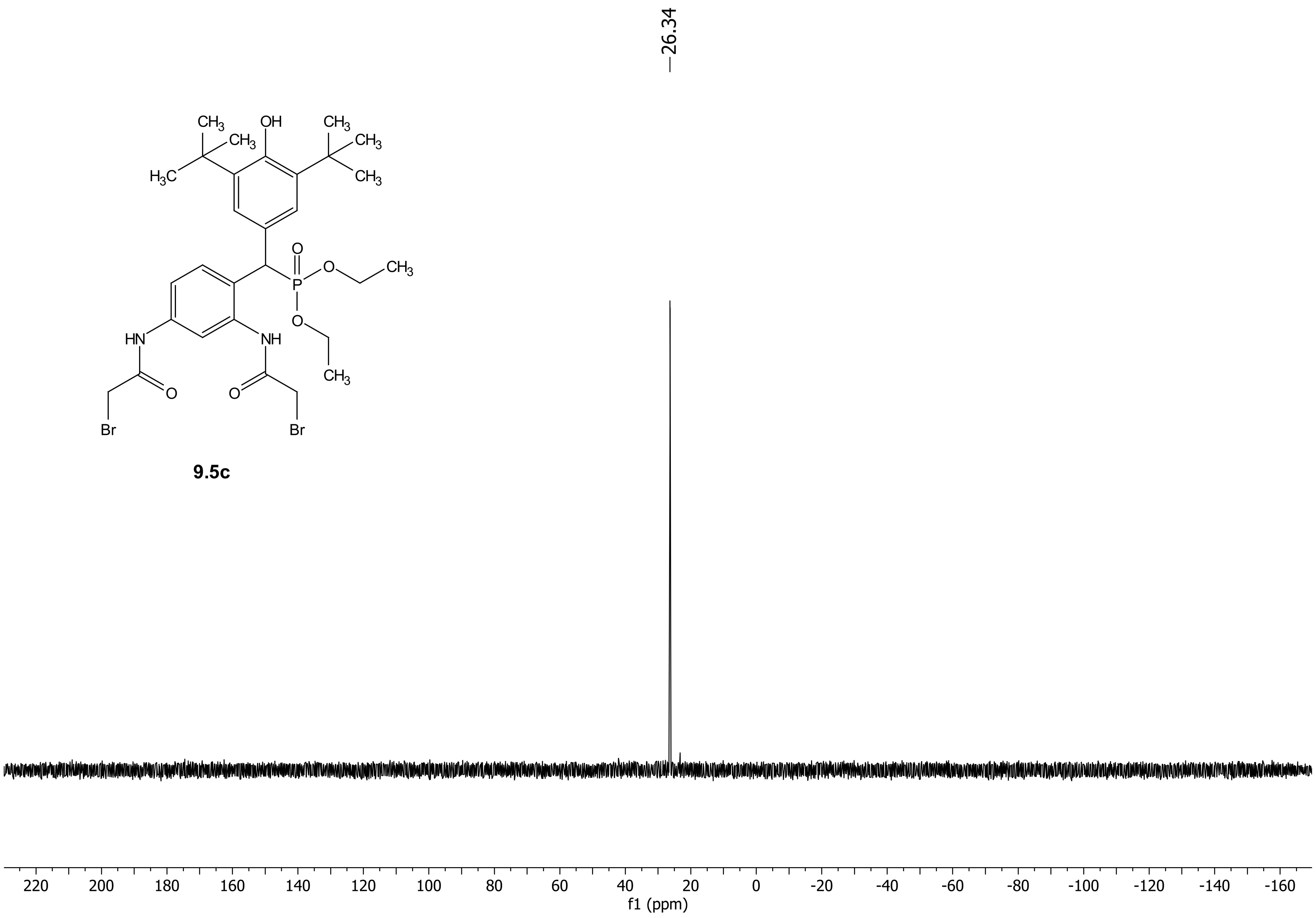


**Figure S67.** $^{31}P\{^1H\}$ NMR spectrum ($(CD_3)_2SO$, 243MHz) of the compound **9.5c**

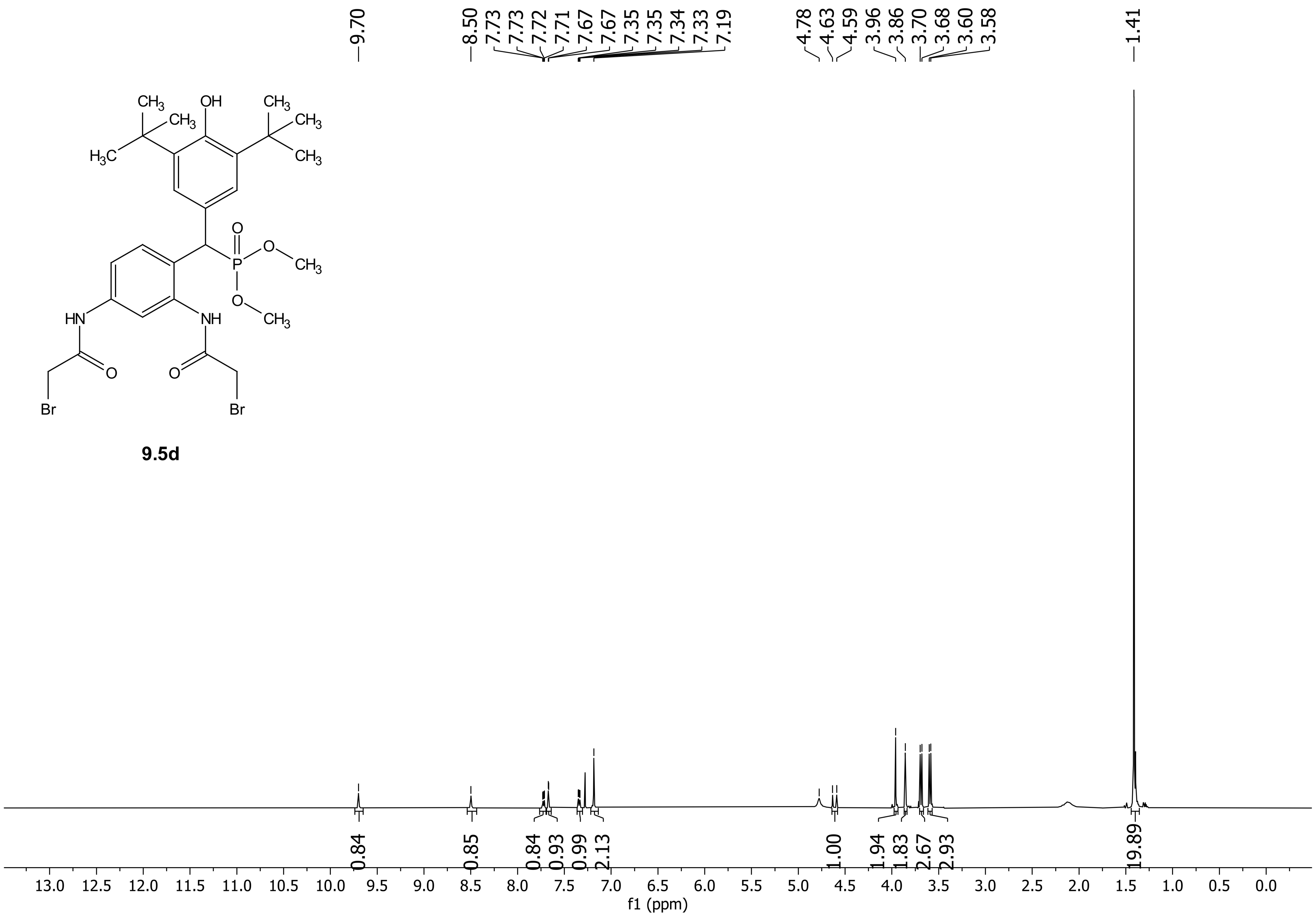


**9.5d**

**Figure S68.** $^{1}H$ NMR spectrum ($(CD_3)_2SO$, 600MHz) of the compound **9.5d**

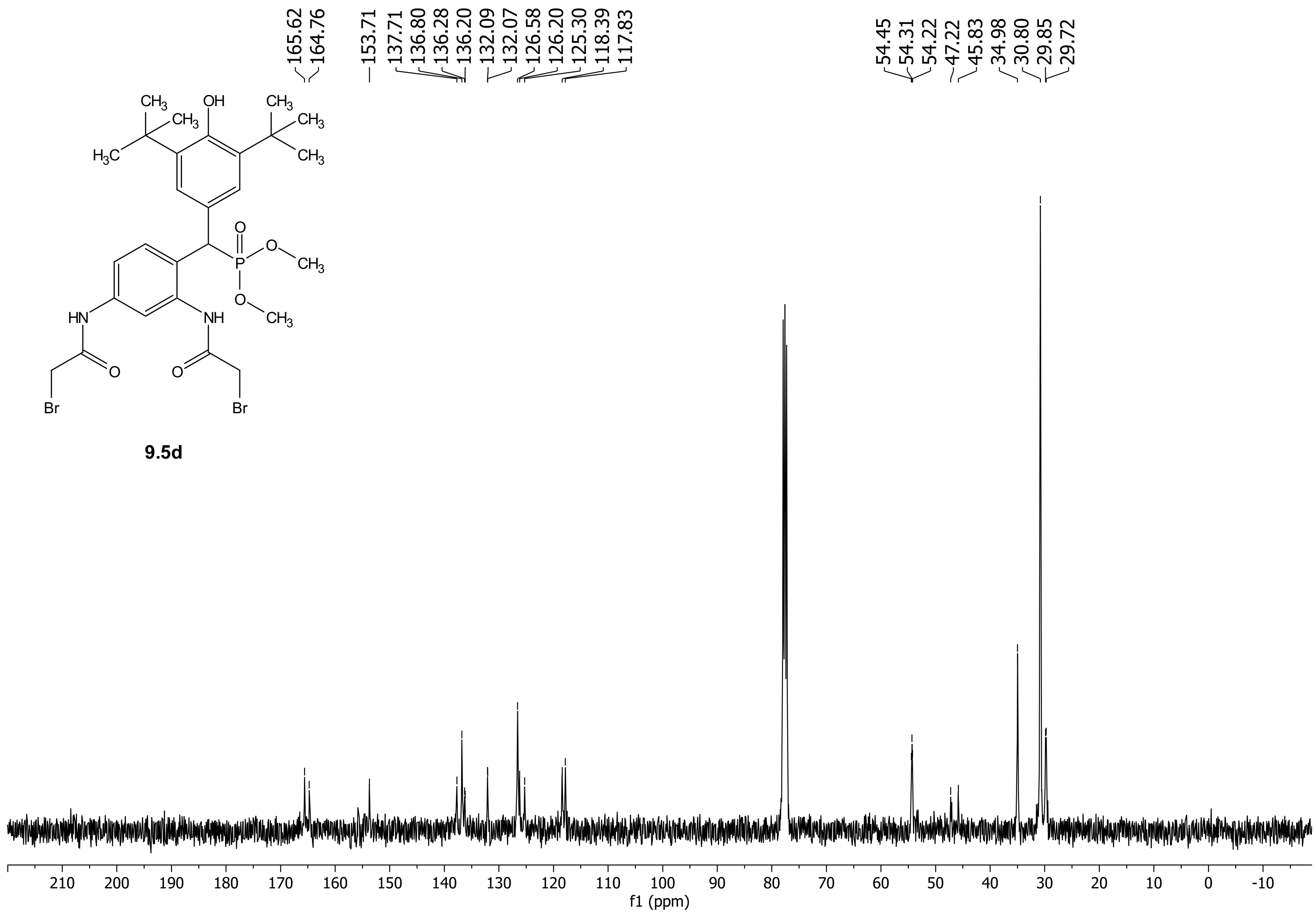


**Figure S69.** $^{13}C\{^{1}H\}$ NMR spectrum ($(CD_3)_2SO$, 101MHz) of the compound **9.5d**

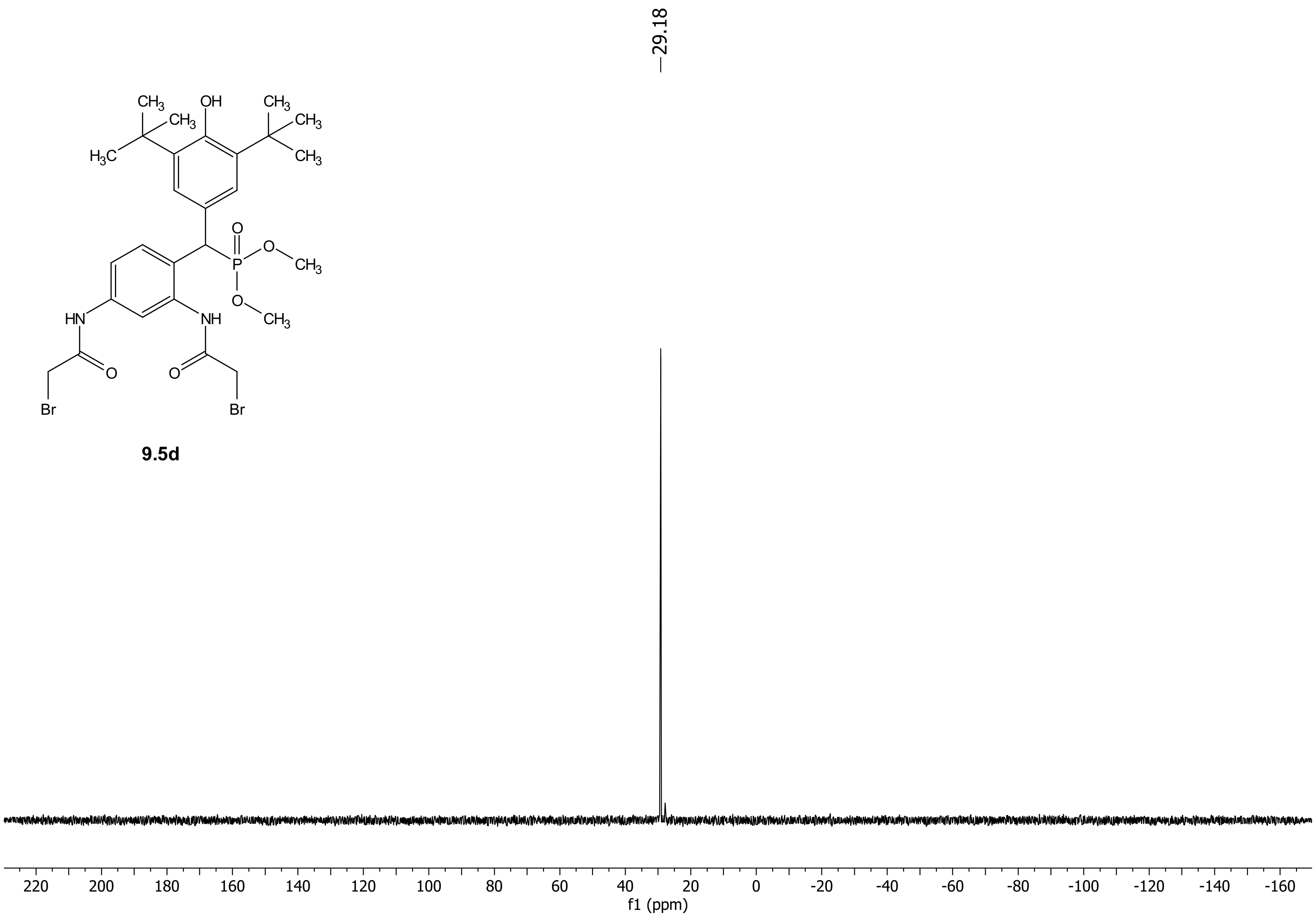


**Figure S70.** $^{31}P\{^1H\}$ NMR spectrum ($(CD_3)_2SO$, 243MHz) of the compound **9.5d**

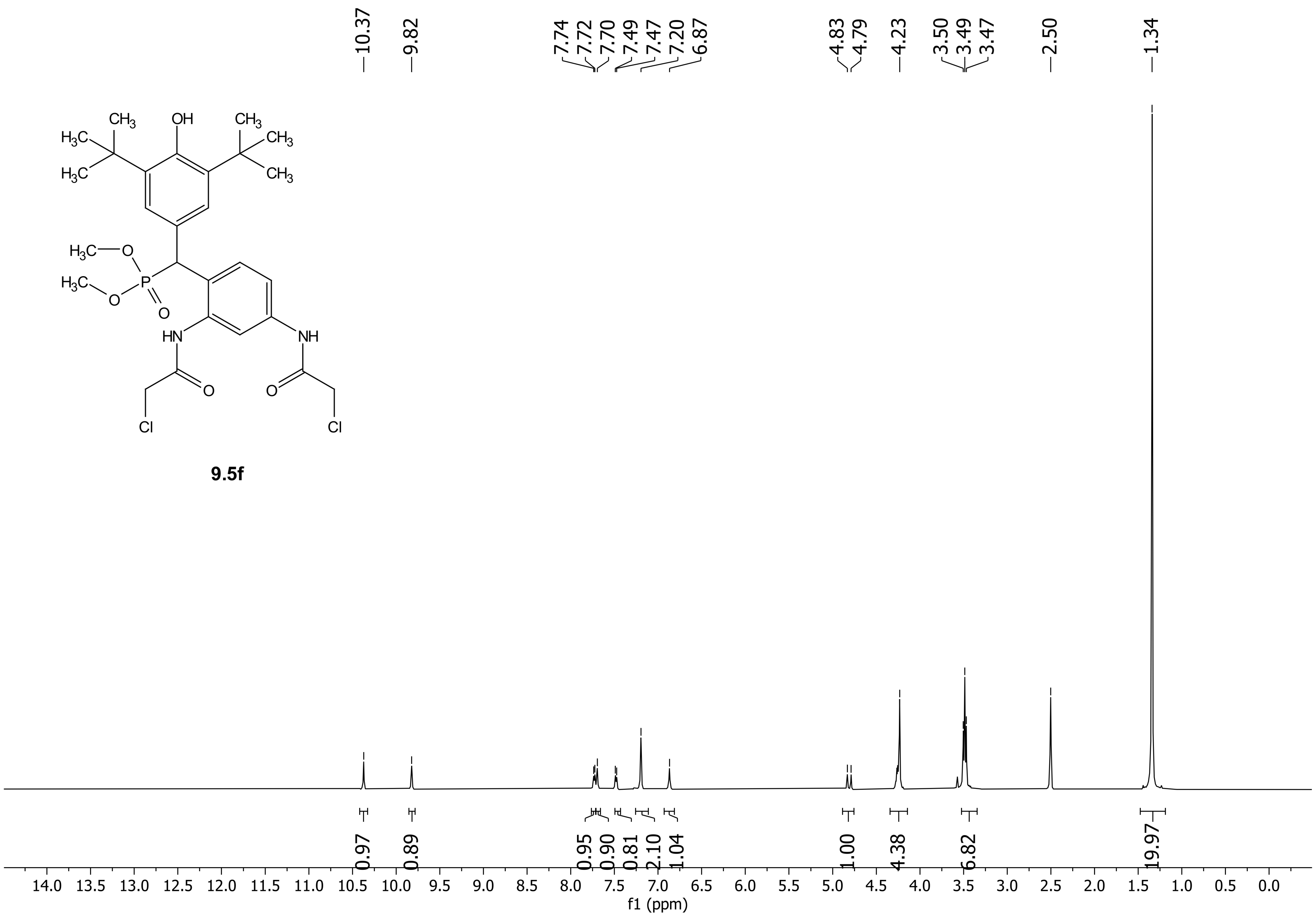


**Figure S71.** $^{1}H$ NMR spectrum ($(CD_3)_2SO$, 600MHz) of the compound **9.5f**

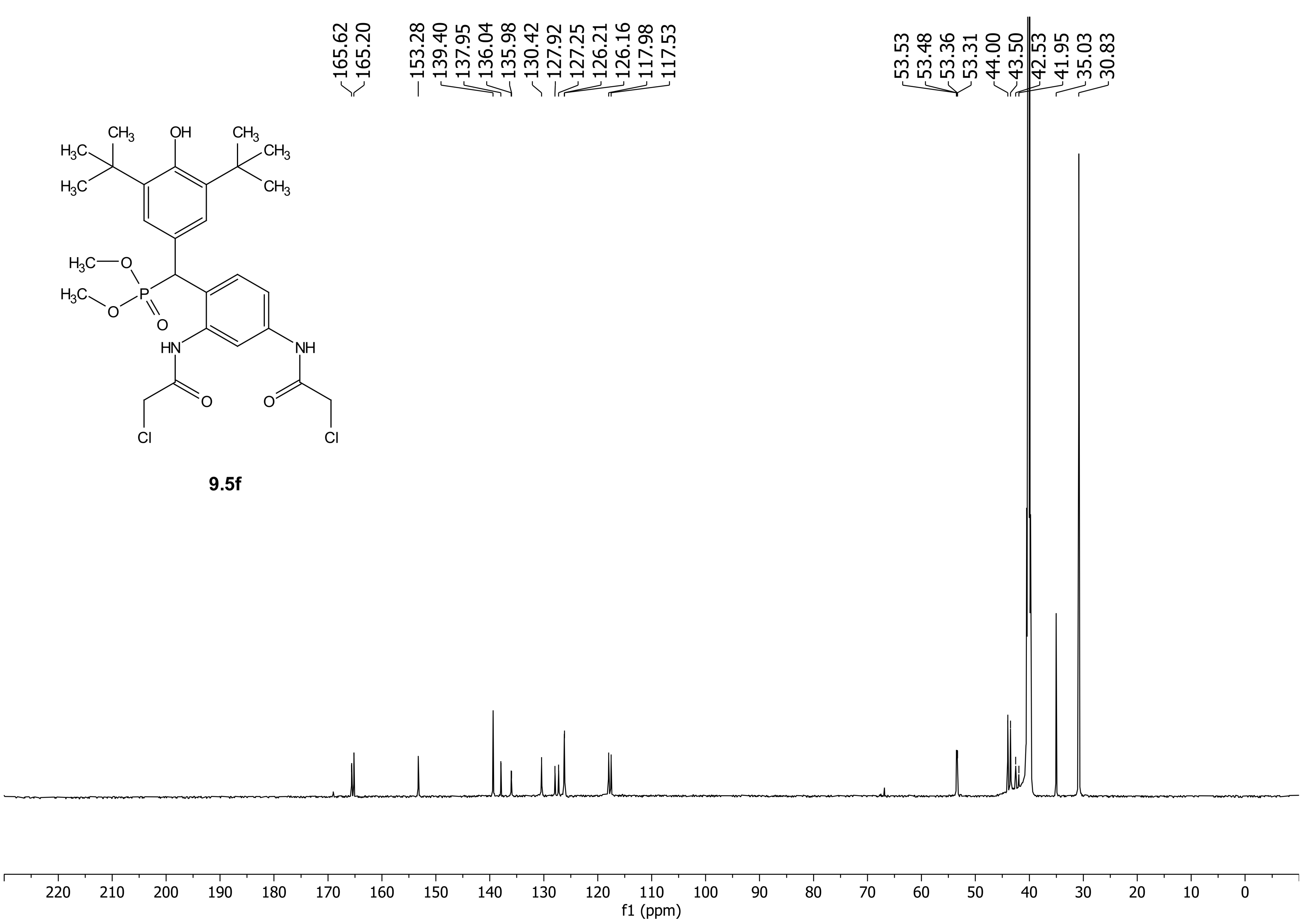


**Figure S72.** $^{13}C\{^{1}H\}$ NMR spectrum ($(CD_3)_2SO$, 151MHz) of the compound **9.5f**

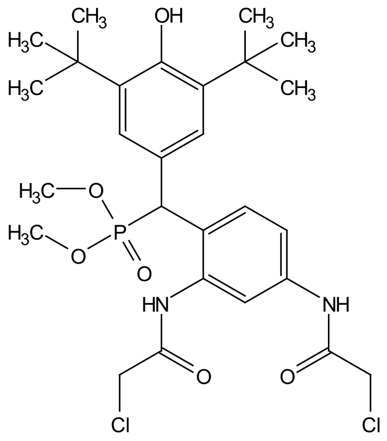


**9.5f**

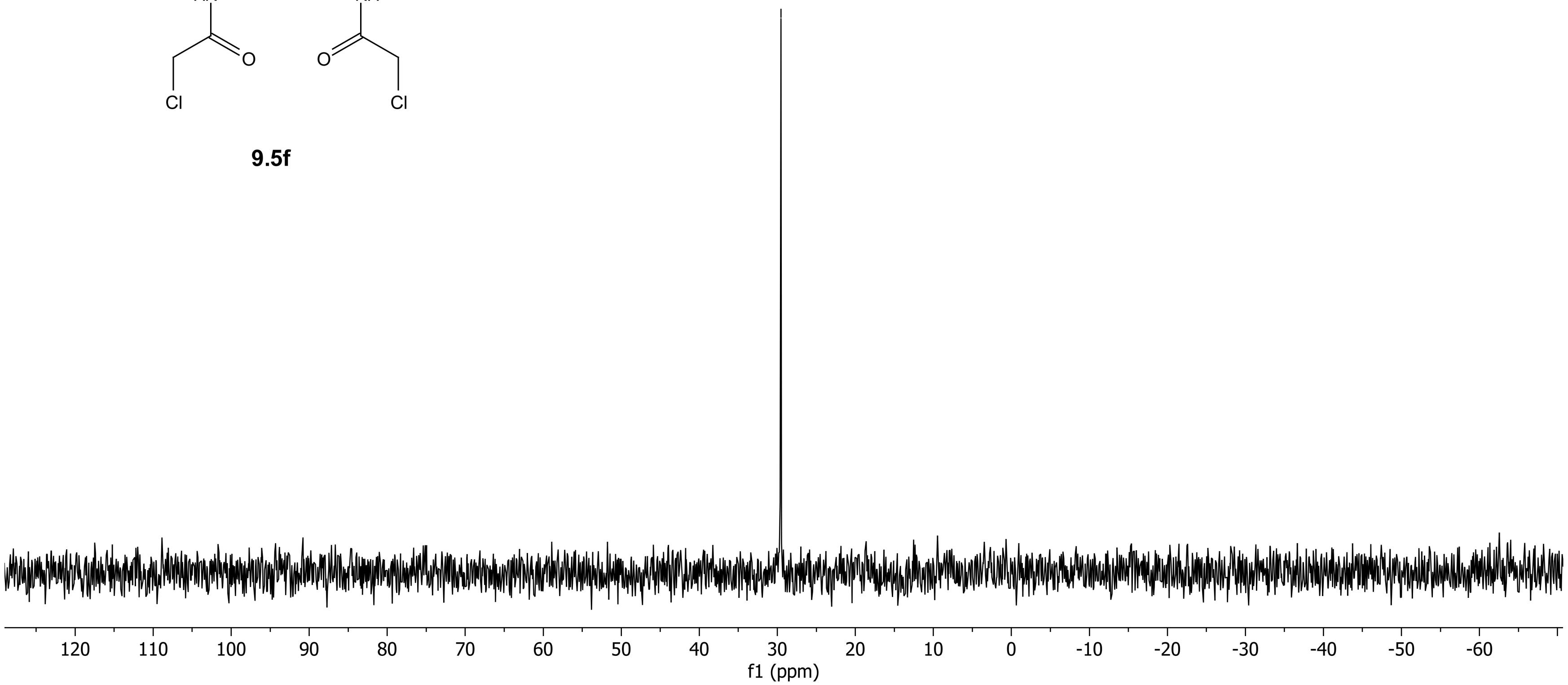


**Figure S73.** $^{31}P\{^{1}H\}$ NMR spectrum ($(CD_3)_2SO$, 243MHz) of the compound **9.5f**

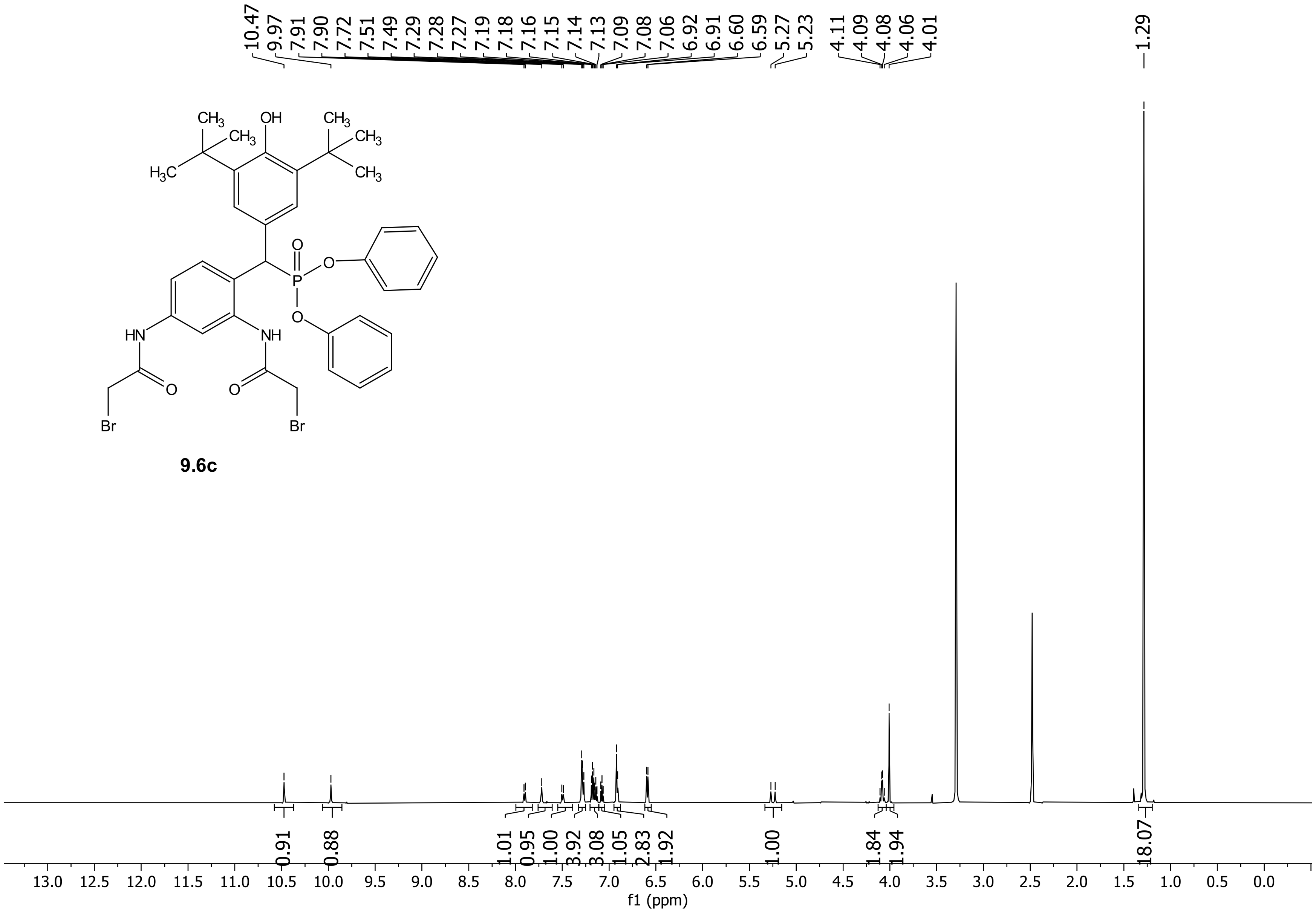

9.6c

**Figure S74.** $^1H$ NMR spectrum ($(CD_3)_2SO$, 600MHz) of the compound **9.6c**

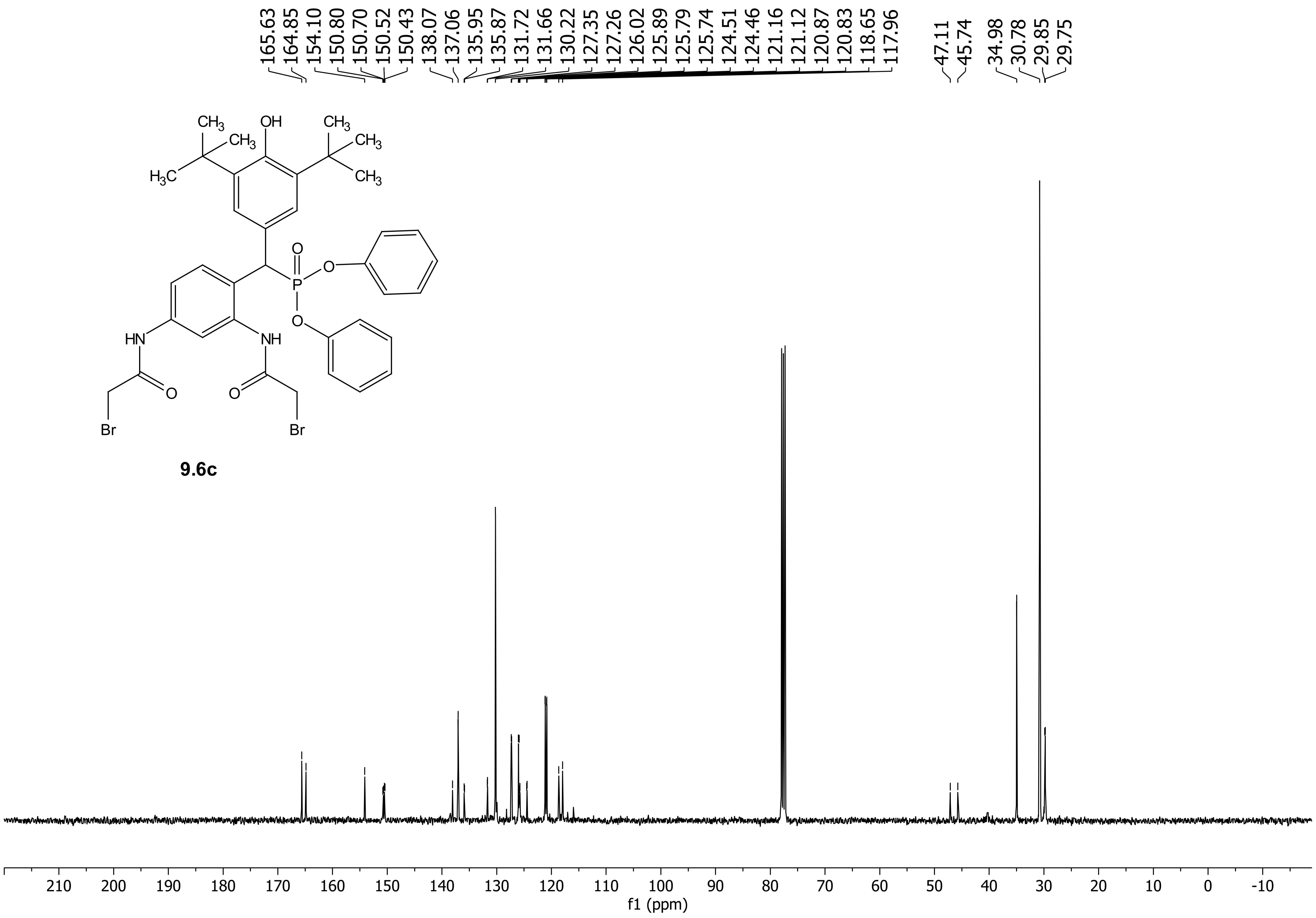


**Figure S75.** $^{13}C\{^1H\}$ NMR spectrum ($(CD_3)_2SO$, 101MHz) of the compound **9.6c**

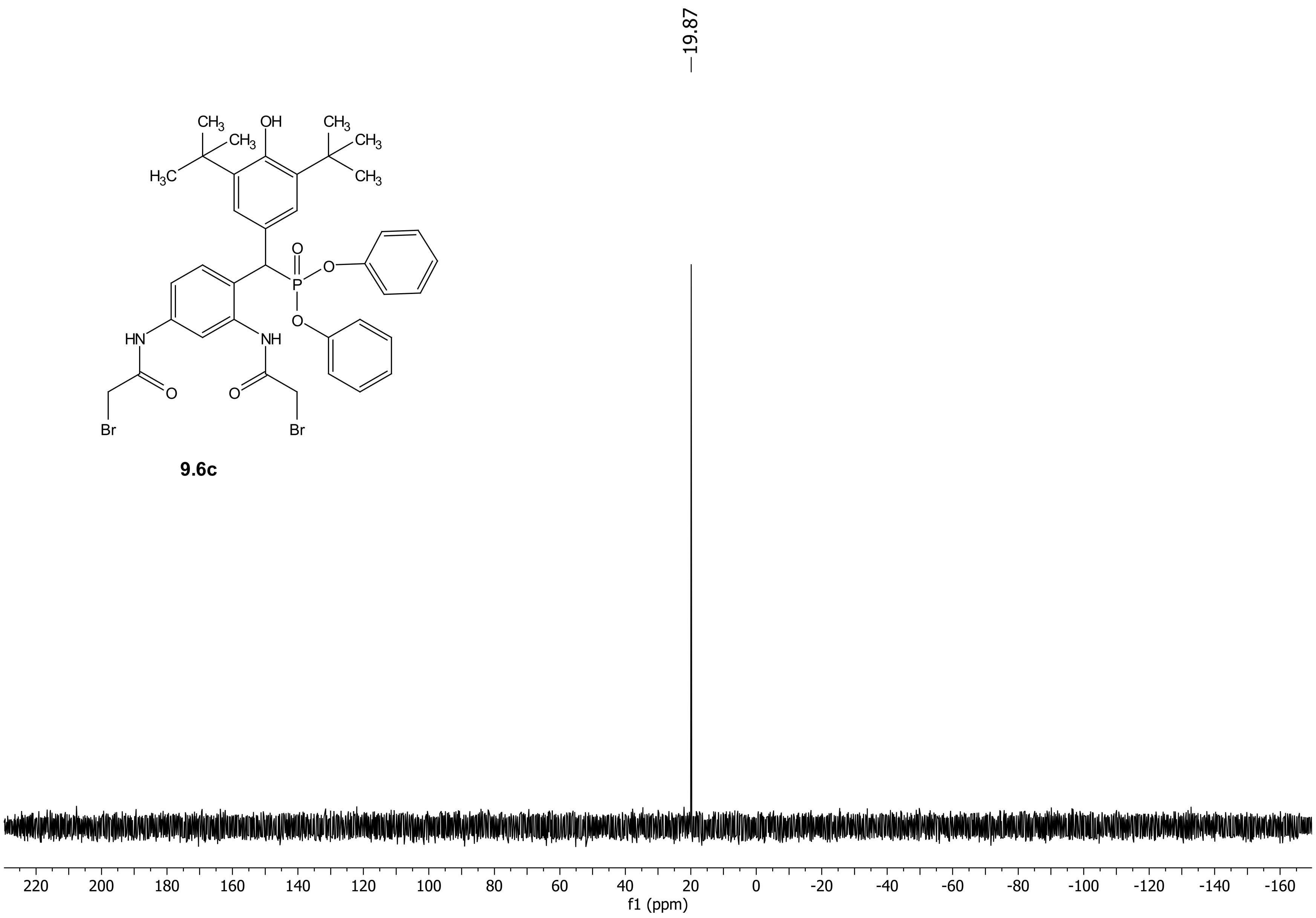


**Figure S76.** $^{31}P\{^{1}H\}$ NMR spectrum ($(CD_3)_2SO$, 243MHz) of the compound **9.6c**

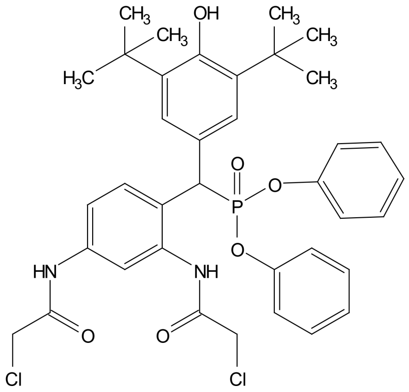


**9.6e**

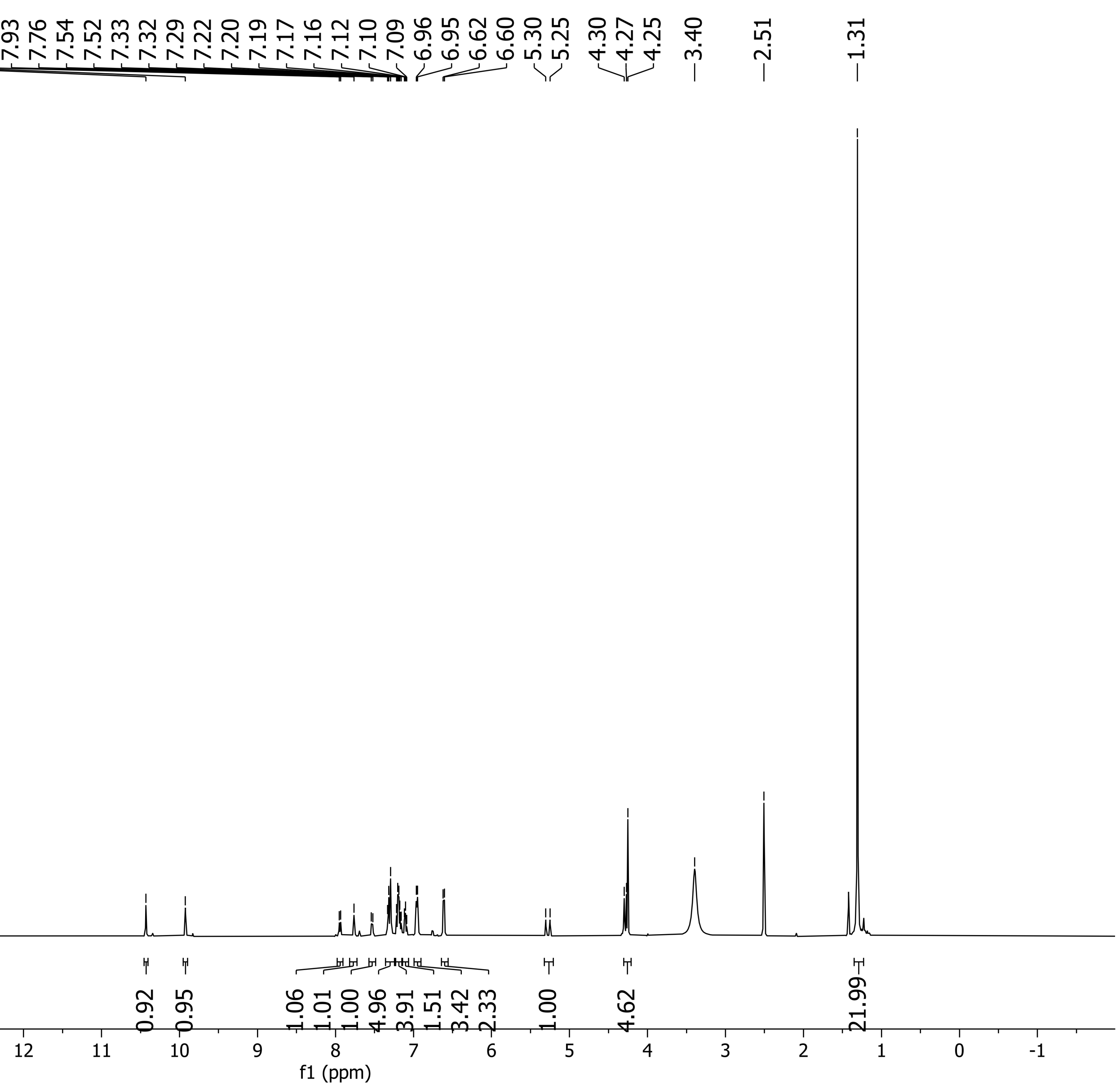


**Figure S77.** $^{1}$H NMR spectrum ($(CD_3)_2SO$, 500MHz) of the compound **9.6e**

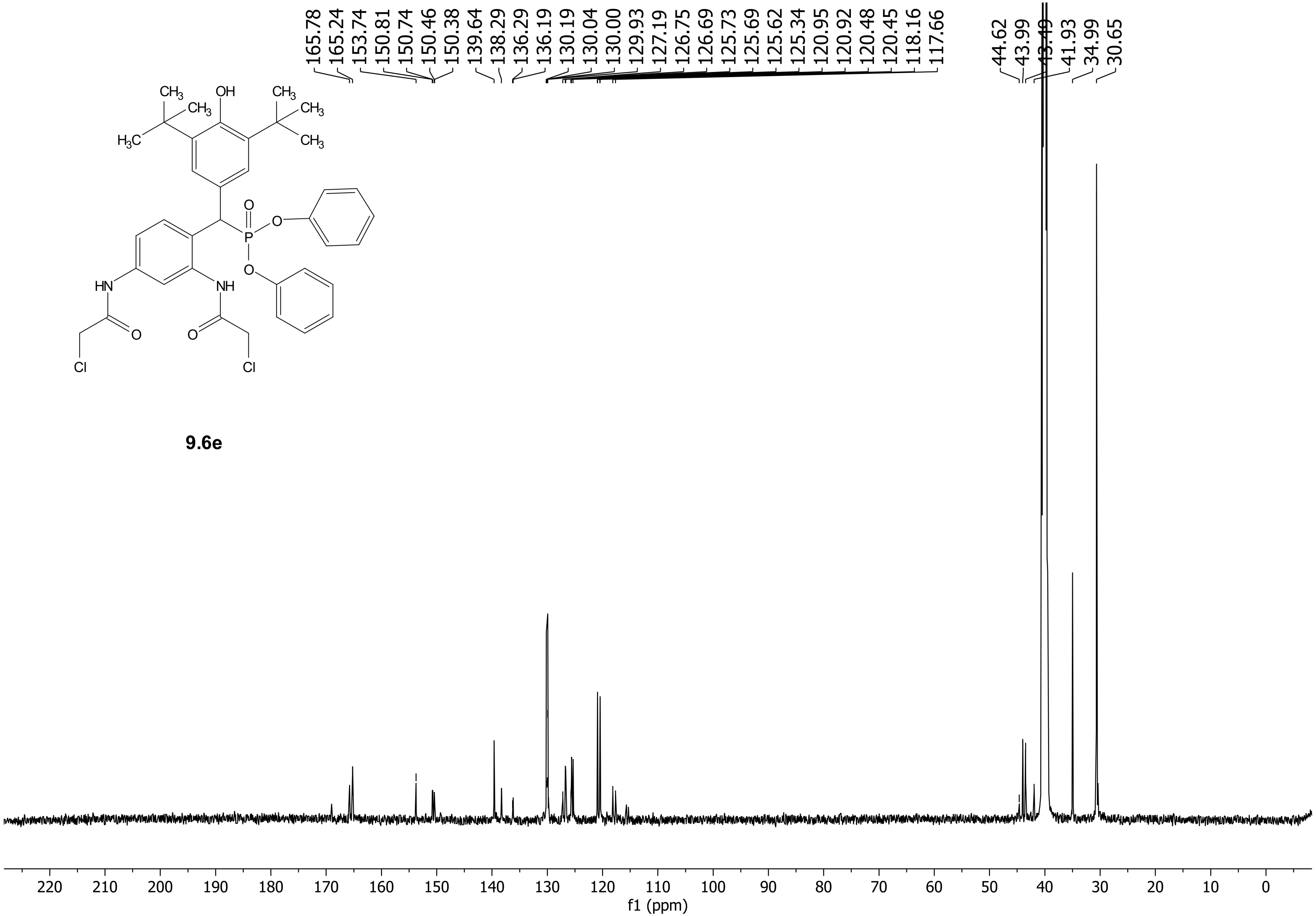


**9.6e**

**Figure S78.** $^{13}C\{^{1}H\}$ NMR spectrum ($(CD_3)_2SO$, 126MHz) of the compound **9.6e**

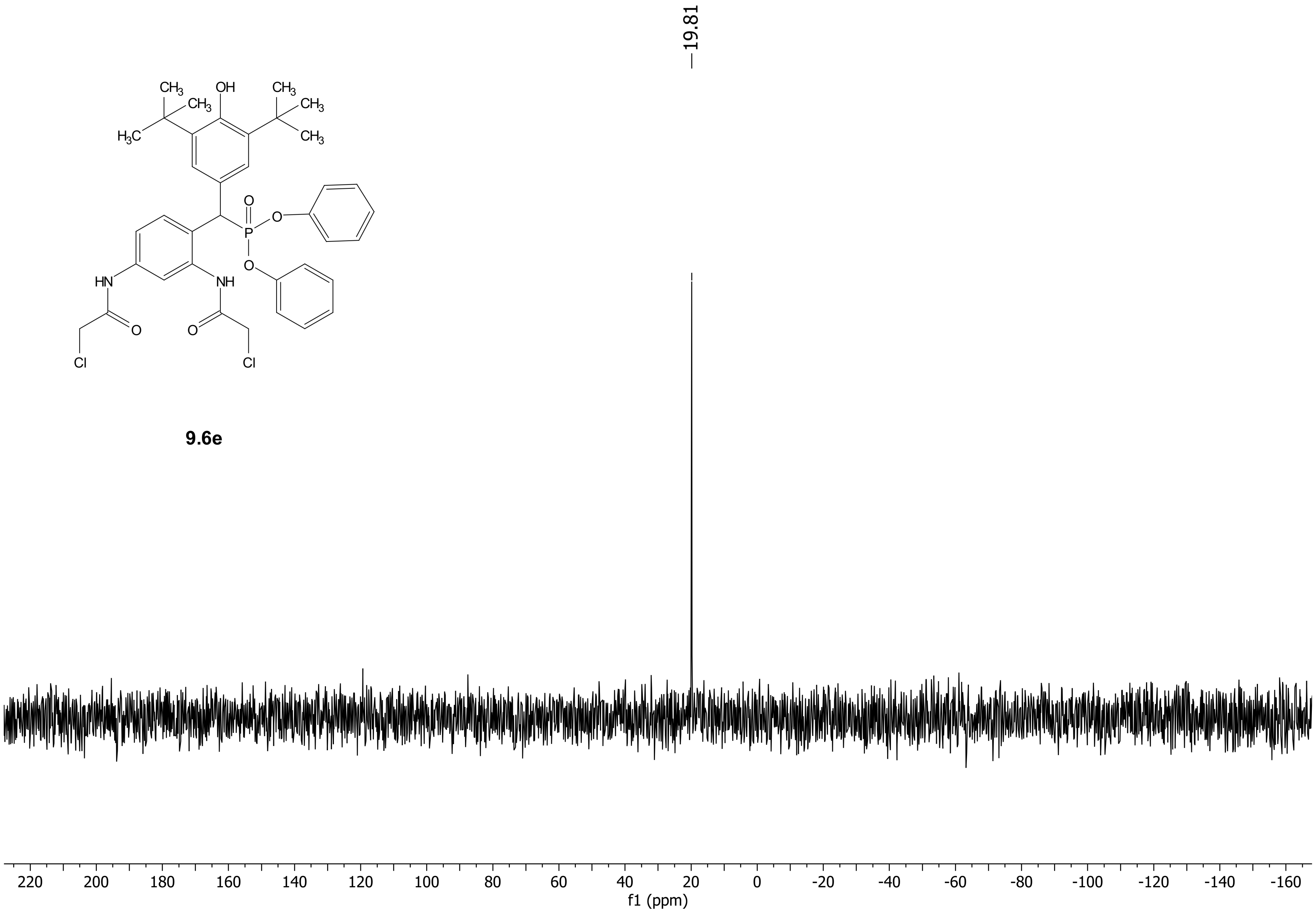


**9.6e**

**Figure S79.** $^{31}P\{^{1}H\}$ NMR spectrum ($(CD_3)_2SO$, 152MHz) of the compound **9.6e**
