# Supplementary material for: Combining Ligand- and Structure-Based Approaches to Develop Haloacetamide-Derived Di(hetero)arylmethanes as Potential Anticancer Agents
Source: Int J Mol Sci. 2026 Oct 26;27(21):9559. doi: 10.3390/ijms27219559 (PMC13762480; doi:10.3390/ijms27219559)
Supplement: Supplementary file 1 [file ijms-27-09559-s001.zip › SI_v7_IJMS_r1_v4.pdf]

Combining ligand- and structure-based approaches to develop haloacetamide-derived di(hetero)arylmethanes as potential anticancer agents

Karina O. Shibaeva,[a] Andrey V. Smolobochkin,[a] Elena A. Chugunova,[a] Hoang Bao Tran Nguyen,[b] Adel M. Shakirov,[a] Elmira M. Gibadullina,[a] Anna P. Lyubina,[a] Anastasiia S. Sapunova,[a] Alexandra D. Voloshina,[a] Yulia R. Aleksandrova,[c] Margarita E. Neganova,[c] Almir S. Gazizov*,[a] Alexander R. Burilov,[a] Michail A. Pudovik,[a] Oleg G. Sinyashin[a]

[a] *Arbuzov Institute of Organic and Physical Chemistry, FRC Kazan Scientific Center, Russian Academy of Sciences, Arbuzova str. 8, Kazan, Russian Federation; e-mail: agazizov@iopc.ru*

[b] *Kazan National Research Technological University, 420015K. Marksa str. 68, Kazan, Russian Federation*

[c] *A.N. Nesmeyanov Institute of Organoelement Compounds of Russian Academy of Sciences, Vavilova str., 28, Moscow, Russian Federation*

Contents

Docking studies 2

General 2

Reference ligands docking 5

Ligands docking 6

Potential hits prediction 14

Full cytotoxicity data for compounds 9.5c-f and 9.6c,e 19

References 20

Docking studies

General

For the docking, a tetramer of PKM2 (PDB code 3GR4), GAPDH (PDB code 6M61) and a dimer of GSTO1 (PDB code 4YQU) were obtained from the Protein Data Bank. Receptors were prepared for covalent docking by first removing all solvent and ligand molecules (except for NAD^+ molecule for GAPDH, see below), adding hydrogen atoms and charges using ChimeraX software,[1] and processing them with Meeko 0.6.1 python package.[2] For the covalent docking to the Cys317, Cys326, and Cys474 residues of the PKM2, only subunit A of the tetramer was used based on the spatial positions of these residues (Figure S1). For the Cys31 and Cys424 residues, subunits A/B and A/C were considered, respectively, due to their proximity to these residues (Figure S2). For the GAPDH (Cys152 residue), only O subunit of the tetramer was used for the docking (Figure S3). The docking was performed both with the NAD^+ removed from the binding site and with NAD^+ retained in its native position (hereafter denoted as GAPDH and GAPDH (NAD^+)). For the docking to the Cys32 residue of GSTO1, only subunit A was used (Figure S4).

The box dimensions and coordinates of its center used in docking are provided in Table S1.

Table S1 Docking grid parameters used for covalent docking

Protein	Residue	Grid dimensions x, y, z (points)[a]	Grid center x, y, z (Å)
PKM2	Cys317	74, 74, 70	−7.304, 4.252, 16.082
PKM2	Cys326	72, 74, 72	−5.281, 9.462, 5.545
PKM2	Cys474	72, 74, 70	−22.239, −16.233, 1.186
PKM2	Cys31	72, 70, 76	18.994, −0.338, 4.908
PKM2	Cys424	74, 70, 76	4.394, −16.740, 5.446
GAPDH	Cys152	94, 94, 96	48.185, 27.445, 16.743
GSTO1	Cys32	88, 90, 82	19.102, −9.945, 0.776

[a] *grid spacing was 0.375 Å for all docking calculations, the respective box size is equal to n_points X grid_size*

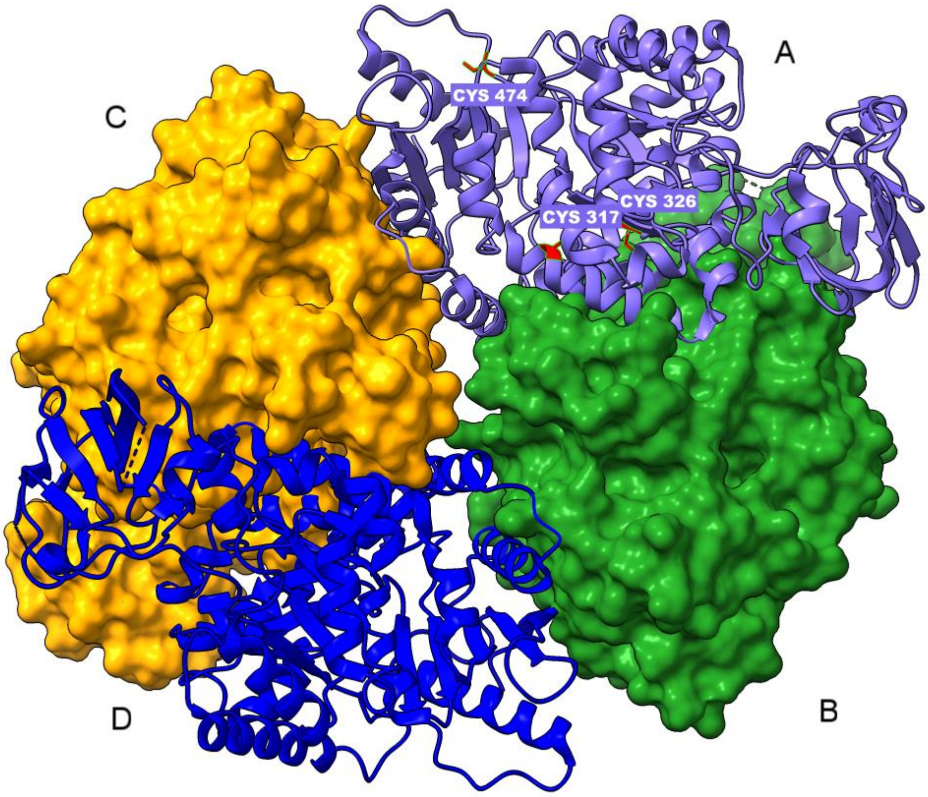

Figure S1 Tetramer of PKM2 (PDB code 3GR4) with Cys317, Cys326, and Cys474 residues highlighted in red

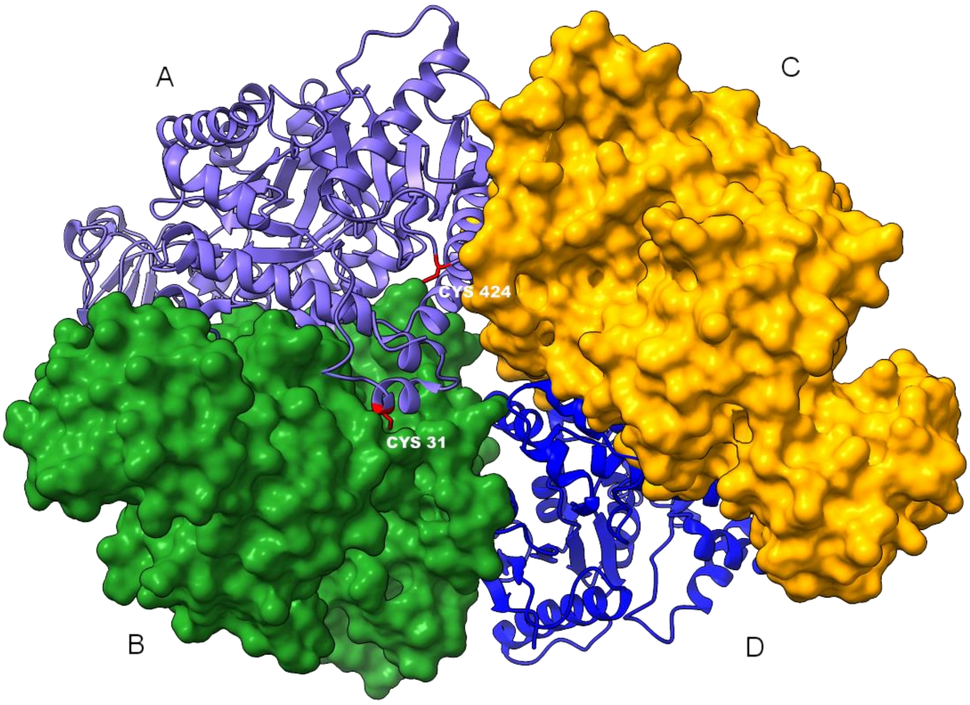

Figure S2 Tetramer of PKM2 (PDB code 3GR4) with Cys31 and Cys424 residues highlighted in red

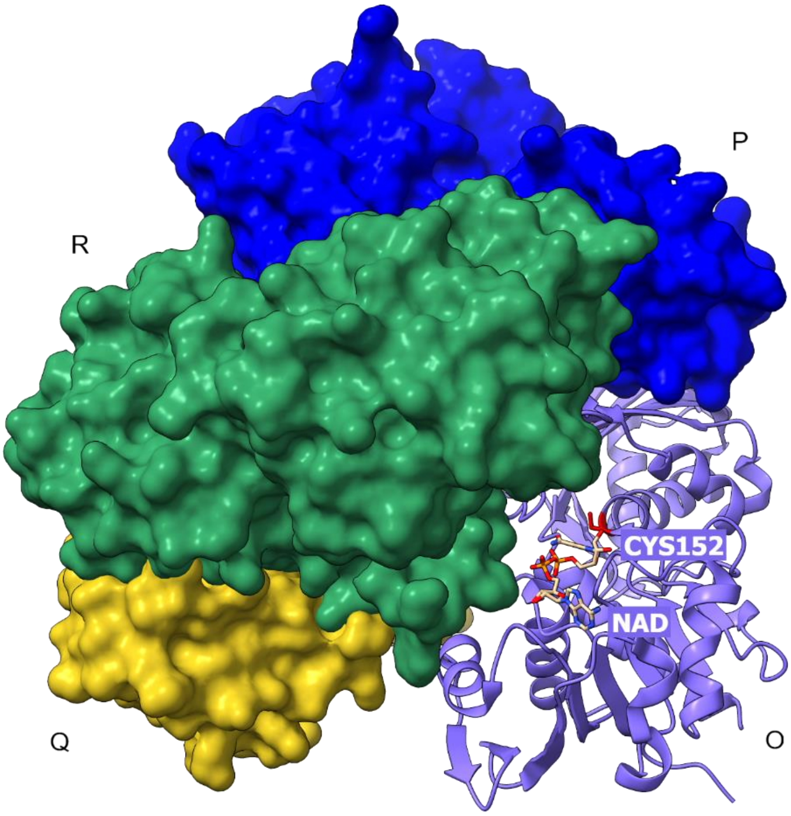

Figure S3 Tetramer of GAPDH (PDB code 6M61) with highlighted Cys152 residue (red) and NAD$^+$ molecule

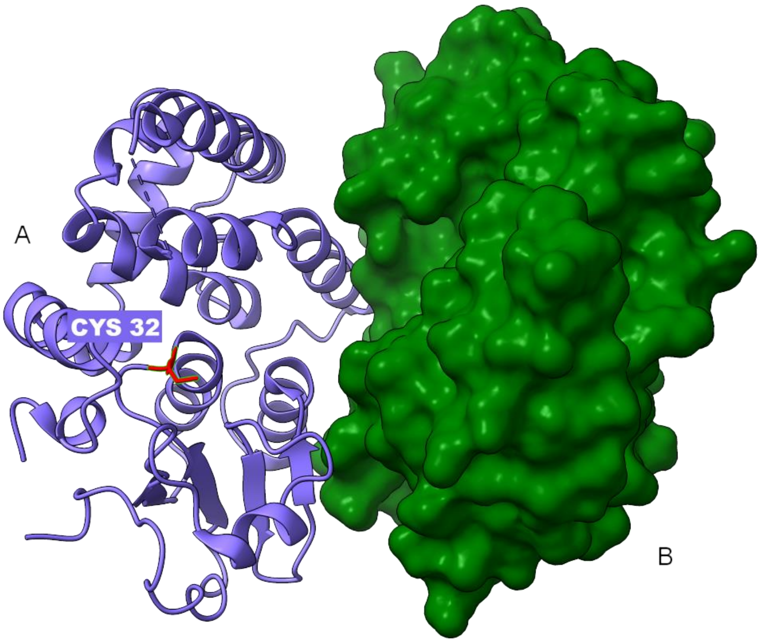

Figure S4 Dimer of GSTO1 (PDB code 4YQU) with Cys32 residue highlighted in red

Ligands for covalent docking were prepared by first converting their SMILES representations to 3D coordinates (XYZ format) using the Open Babel 3.1.1 program,[3] followed by geometry optimization with the semiempirical GFN2-xTB method[4] as implemented in ORCA 6.0.0.[5,6] The optimized structures were further prepared for docking using Meeko 0.6.1 python package.[2] For compounds **9.1**-**9.6**, which possess a stereocenter, both (*R*)- and (*S*)-enantiomers were modelled to assess whether the stereochemical configuration substantially affected the predicted binding affinity.

Covalent docking was performed using AutoDock-GPU 1.6[7] with the default settings and the number of runs set to 100 (--nruns 100). The affinity grid maps required by AutoDock-GPU were generated using AutoGrid 4.2.7 utility.[8] The generated poses were clustered using the built-in AutoDock-GPU 1.6 clustering tool based on the RMSD (2.0 Å cutoff by default) to identify the most populated clusters and their lowest-energy conformations. All docking runs were carried out in triplicate to confirm the reproducibility of the predicted binding poses and energies; the mean docking score from the three runs was used in subsequent analysis.

PLIP v2.4.0[9] was used for protein-ligand interaction analysis.

Reference ligands docking

To validate the selected docking protocol, we first performed covalent docking of the reference ligands. Heptelidic acid from PDB entry 6M61 was used as the reference ligand for GAPDH, whereas *N*-(5-(azepan-1-ylsulfonyl)-2-methoxyphenyl)-2-chloroacetamide (**C1-31**) from PDB entry 4YQU served as the reference ligand for GSTO1. No PDB entry containing a covalently bound ligand was available for PKM2; therefore, reference docking was not performed for this target.

In both cases, the lowest-energy pose in the most populated conformational cluster closely reproduced the reference binding pose (Figure S5). For GAPDH and heptelidic acid, the RMSD was 1.063 ± 0.022 Å (mean ± SD), and the docking score was -7.42 kcal/mol (mean value). For GSTO1 and **C1-31**, the docking score was -9.08 kcal/mol (mean value), and the RMSD was 2.084 ± 0.012 Å. The latter value is at the upper limit of the acceptable range. Nevertheless, given the high convergence between replicate runs and the conformational flexibility of the seven-membered azepane ring, which is challenging to reproduce, this result was considered suitable for the purposes of this study.

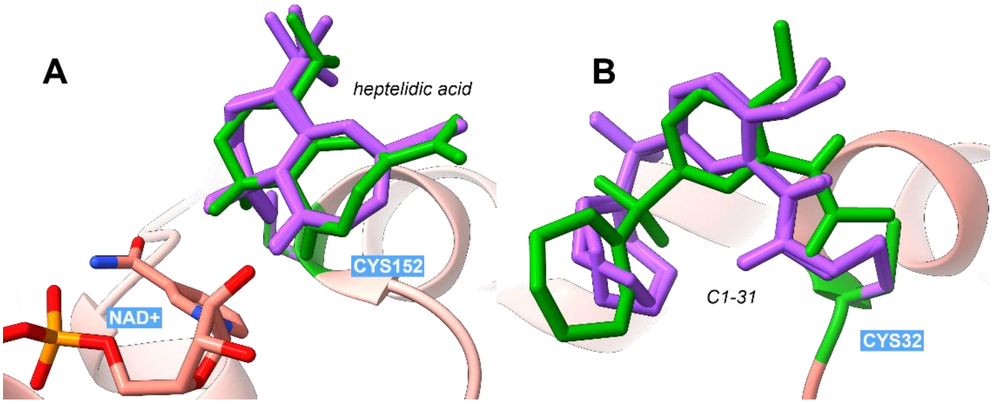

Figure S5 Comparison of highest-scored covalent docking poses with reference ligands for GAPDH/heptelidic acid (A) and GSTO1/**C1-31** (B) (original ligands, green; docked poses, magenta)

Comparison of the original and re-docked GSTO1 ligand poses showed that several hydrophobic interactions were retained. In particular, interactions with Leu56, Ile131, and Phe34 were reproduced in the re-docked pose, with distances of 3.54, 3.37, and 4.75 Å, respectively, compared with 3.94, 3.74, and 4.94 Å in the original structure.[10] An additional hydrophobic contact with Trp222 was detected in the re-docked pose. Interactions with Val72, Phe225, Leu226, and Tyr229 observed in the original structure were not reproduced. Overall, the re-docked pose retained several of the principal hydrophobic interactions observed in the experimentally determined binding mode (Figure S6).

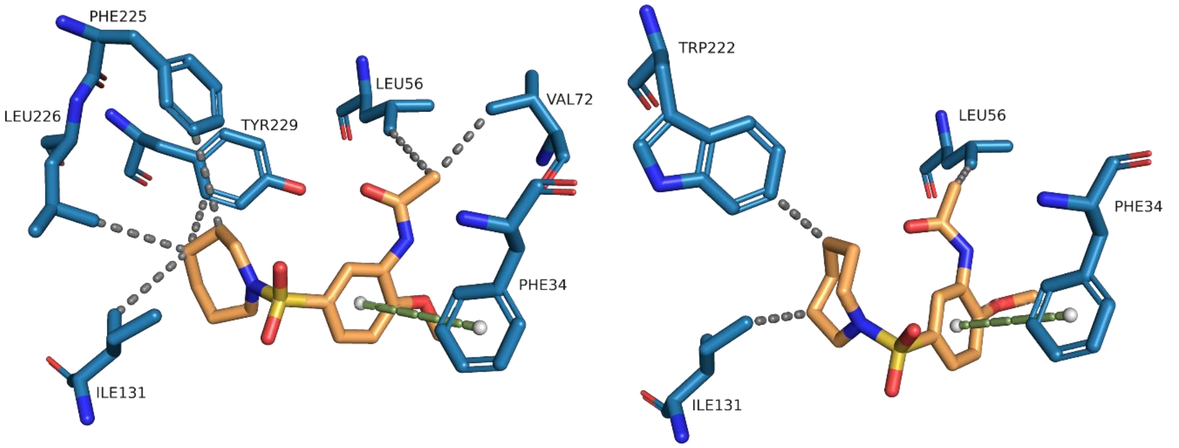

Figure S6 Interactions of the original (left) and redocked (right) poses of the reference ligand for GSTO1

For GAPDH, comparison of the original and re-docked heptelidic acid poses showed preservation of several interactions relevant to ligand positioning in the active site. The hydrogen bonds with Gly212 and Ala213 were retained in the re-docked pose, with donor–acceptor distances of 3.31 and 3.93 Å, respectively, compared with 3.25 and 3.75 Å in the original structure.[11] The hydrophobic contact with Thr177 was also retained, with a distance of 3.97 Å in the re-docked pose compared with 3.80 Å in the original structure. Thus, despite local differences in the interaction pattern, the re-docked pose retained several principal interactions observed in the original heptelidic acid-GAPDH complex (Figure S7).

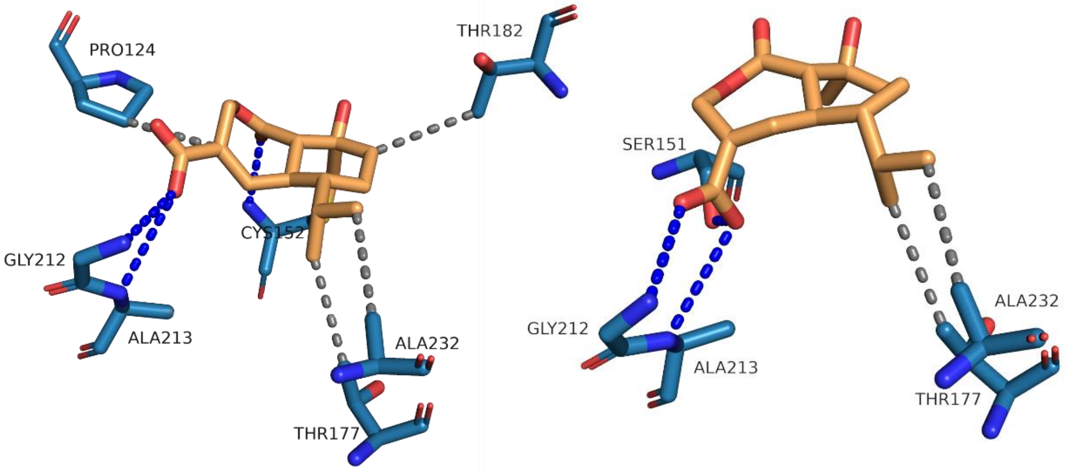

Figure S7 Interactions of the original (left) and redocked (right) poses of the reference ligand for GAPDH

Ligands docking

For compounds **4b** and **4c**, which contain two pyrazol-3-one moieties, three tautomeric forms are possible (Figure S8). According to studies by Feeney[12] and Mazzola,[13] the chemical shift of the carbon atom directly bonded to the oxygen atom is expected to be *ca* 155 ppm for the pyrazolones in the enol forms **4b″** and **4c″** (OH/OH) and *ca* 165 ppm for those in the keto forms **4b** and **4c** (NH/NH). Because the corresponding carbon atoms in compounds **4b** and **4c** resonate at *ca* 157 ppm, we conclude that these compounds exist predominantly in the enol form. The predominance of the enol form was also observed in 10% d_6-DMSO in D_2O,[14] a solvent system resembling an aqueous biological environment. We included nevertheless all three tautomeric forms of both compounds in the docking studies. In most cases, the OH/OH tautomers **4b″** and **4c″** showed essentially the same affinity as the NH/NH (keto) tautomers. Therefore, these affinity values were used to correlate target affinity with cytotoxicity toward the M-HeLa and HuTu-80 cancer cell lines. The affinity scores for the docked ligands are given in Table S2.

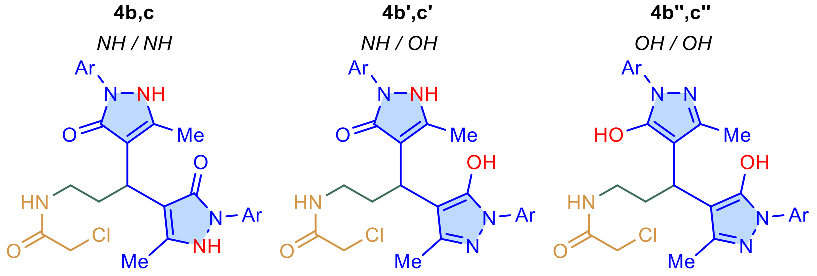

*Ar = 4-Me-C_6H_4 (**4b**), 4-Cl-C_6H_4 (**4c**)*

Figure S8 Possible tautomers for the compounds **4b** and **4c**

To assess whether docking scores were associated with cytotoxicity, Pearson correlation analysis was performed between the mean docking scores and the corresponding cytotoxicity values for each protein/residue model and cell line. For each correlation, the Pearson correlation coefficient (*r*) and two-sided *p* value were calculated. Because multiple correlations were evaluated, *p* values were adjusted using the Bonferroni correction. Statistical significance was defined as an *adjusted p* < 0.05. Each correlation was based on n = 10 compounds. The complete correlation analysis is presented in Figures S9-S16 and Table S3.

Leave-one-out (LOO) sensitivity analysis was performed for the three docking score–cytotoxicity correlations that remained significant after Bonferroni correction. For each correlation, observations were sequentially omitted and Cook's Distance was calculated to assess the influence of individual compounds on the fitted relationship. A threshold of 4/N was used to identify potentially influential observations, where N = 10. None of the observations exceeded this threshold in any of the three analyses, indicating that no individual compound was identified as influential according to the applied criterion (Figures S17-S19).

Table S2 Covalent docking scores for the M2 splice isoform of pyruvate kinase (PKM2, PDB code 3GR4), glyceraldehyde 3-phosphate dehydrogenase (GAPDH, PDB code 6M61) and glutathione S-transferase omega 1 (GSTO1, PDB code 4YQU)[a]

Cmpd	GAPDH					
	CYS152			***CYS152 (NAD$^+$)***		
	docking score (mean)	*RMSD (mean)*	*RMSD (max)*	*docking score (mean)*	*RMSD (mean)*	*RMSD (max)*
**1**[b]	-10.75	0.089	0.121	-11.39	0.058	0.08
**2b**[c]	-6.82	0.021	0.025	-6.99	0.024	0.033
**4a**	-12.06	0.038	0.048	-12.83	0.044	0.059
**4b**	-12.86	0.726	1.051	-13.07	0.064	0.081
**4b'**	-13.43	0.731	1.047	-13.05	0.529	0.746
**4b''**[b]	-12.92	0.503	0.744	-13.07	0.695	0.851
**4c**	-12.92	0.03	0.034	-13.05	0.512	0.748
**4c'**	-13.35	0.539	0.754	-13.21	0.526	0.756
**4c''**[b]	-12.94	0.512	0.747	-12.96	0.029	0.036
**4d**	-11.42	0.803	1.193	-11.73	0.022	0.03
**4e**	-9.78	0.097	0.129	-9.4	0.018	0.02
**4f**	-9.79	0.042	0.051	-9.72	0.077	0.106
**4g**	-10.05	0.068	0.086	-9.51	0.014	0.018
**5b**	-9.40	0.122	0.152	-10.22	0.012	0.016

Cmpd	GSTO1			PKM2		
	CYS32			***CYS31***		
	docking score (mean)	*RMSD (mean)*	*RMSD (max)*	*docking score (mean)*	*RMSD (mean)*	*RMSD (max)*
**1**[b]	-10.46	0.054	0.069	-8.93	0.066	0.091
**2b**[c]	-6.79	0.39	0.469	-5.00	0.04	0.05
**4a**	-11.64	0.143	0.201	-10.53	0.208	0.287
**4b**	-12.81	0.793	1.076	-11.32	0.255	0.323
**4b'**	-12.73	0.608	0.787	-10.67	0.528	0.745

**4b’’[b]**	-12.48	0.611	0.834	-10.91	0.073	0.103
**4c**	-12.84	0.891	1.094	-10.97	0.734	1.059
**4c’**	-12.49	0.822	1.126	-10.11	0.173	0.256
**4c’’[b]**	-12.54	0.772	0.933	-11.03	0.519	0.748
**4d**	-10.75	0.193	0.268	-10.2	0.101	0.124
**4e**	-9.34	0.618	0.917	-8.95	0.107	0.152
**4f**	-9.43	0.353	0.496	-7.18	0.026	0.036
**4g**	-9.19	0.213	0.288	-8.11	0.042	0.046
**5b**	-8.37	0.027	0.031	-6.20	0.021	0.029

**Cmpd**	**PKM2**					
	CYS317			***CYS326***		
	docking score (mean)	*RMSD (mean)*	*RMSD (max)*	*docking score (mean)*	*RMSD (mean)*	*RMSD (max)*
**1[b]**	13.87	1.39	1.943	-4.02	0.035	0.048
**2b[c]**	0.34	0.552	0.823	-1.18	0.063	0.089
**4a**	8.41	0.065	0.075	-5.07	0.056	0.08
**4b**	14.46	0.642	0.781	-4.25	0.055	0.06
**4b’**	14	0.531	0.73	-4.75	0.821	1.121
**4b’’[b]**	10.02	0.611	0.796	-5.59	0.747	0.873
**4c**	14.86	0.88	1.09	-3.44	0.563	0.806
**4c’**	13.96	0.265	0.373	-4.71	1.019	1.241
**4c’’[b]**	10.32	0.55	0.785	-4.61	0.595	0.774
**4d**	15.08	1.432	2.064	-4.45	0.023	0.026
**4e**	15.2	0.024	0.03	-4.05	0.932	1.391
**4f**	17.9	0.084	0.119	3.81	0.066	0.076
**4g**	17.41	0.14	0.198	5.65	0.117	0.152
**5b**	177.98	0.055	0.071	201.85	0.453	0.557

**Cmpd**	**PKM2**					
	CYS317			***CYS326***		
	docking score (mean)	*RMSD (mean)*	*RMSD (max)*	*docking score (mean)*	*RMSD (mean)*	*RMSD (max)*
**1[b]**	-11.42	0.039	0.049	-8.88	0.018	0.025
**2b[c]**	-7.37	0.06	0.076	-5.33	0.02	0.028
**4a**	-12.73	0.62	0.92	-10.40	0.052	0.064
**4b**	-14.72	0.033	0.042	-10.60	0.156	0.215
**4b’**	-15.41	0.525	0.745	-11.46	0.523	0.747
**4b’’[b]**	-15.36	0.031	0.039	-11.71	0.076	0.11
**4c**	-14.65	0.549	0.755	-10.54	0.082	0.107
**4c’**	-13.09	0.123	0.172	-10.09	0.55	0.753
**4c’’[b]**	-15.18	0.737	1.061	-11.74	0.065	0.091
**4d**	-12.84	0.137	0.162	-9.68	0.089	0.13
**4e**	-11.56	0.097	0.133	-7.85	0.014	0.018
**4f**	-10.7	0.062	0.085	-6.35	0.03	0.043
**4g**	-10.4	0.208	0.275	-6.38	0.026	0.038
**5b**	-10.16	0.023	0.027	7.96	0.168	0.234

[a] all docking runs were performed in triplicate; [b] hit compounds; [c] inactive compound

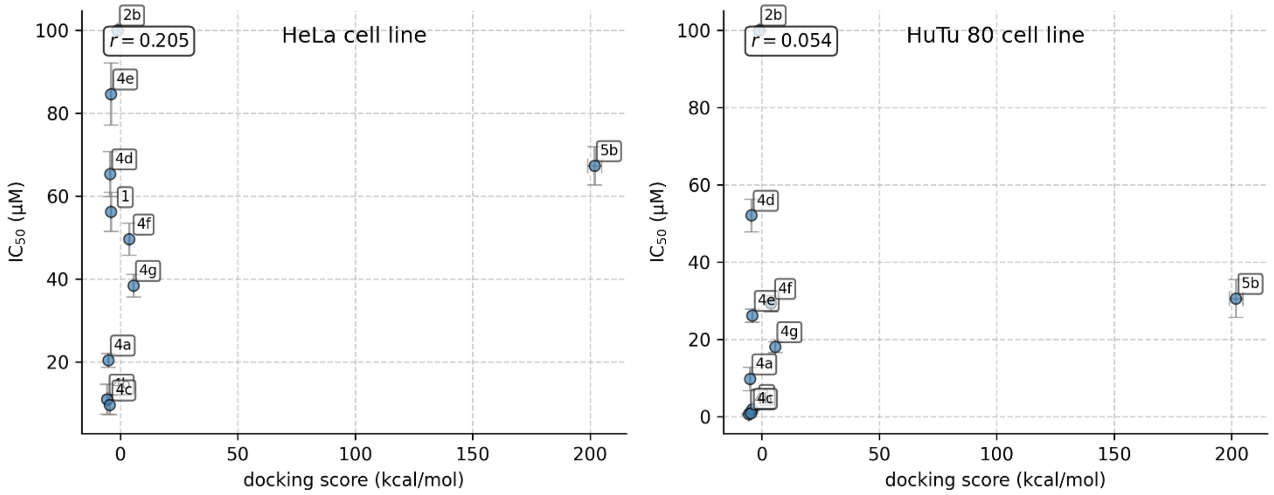

Figure S 9 Covalent docking score *vs* IC_{50} plot for the Cys326 residue of PKM2 in HeLa and HuTu-80 cell lines

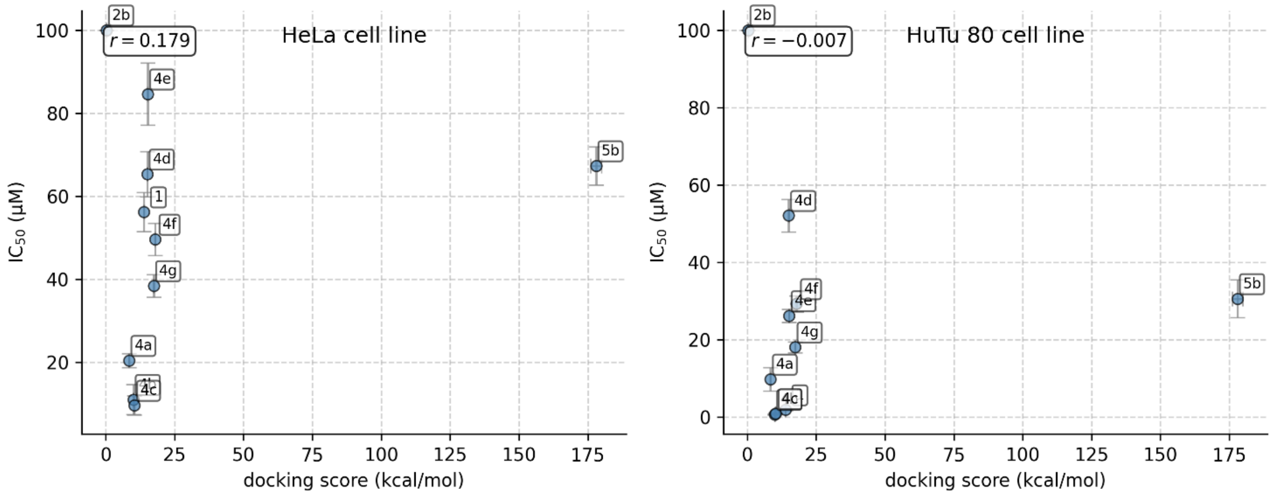

Figure S10 Covalent docking score *vs* IC_{50} plot for the Cys317 residue of PKM2 (PDB code 3GR4) in HeLa and HuTu-80 cell lines

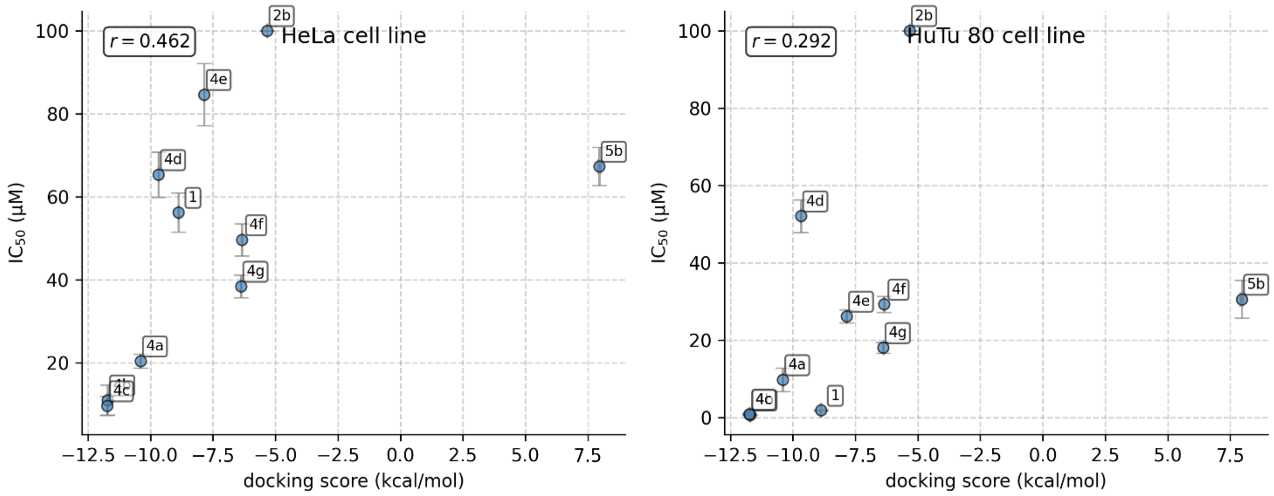

Figure S11 Covalent docking score *vs* IC_{50} plot for the Cys474 residue of PKM2 (PDB code 3GR4) in HeLa and HuTu-80 cell lines

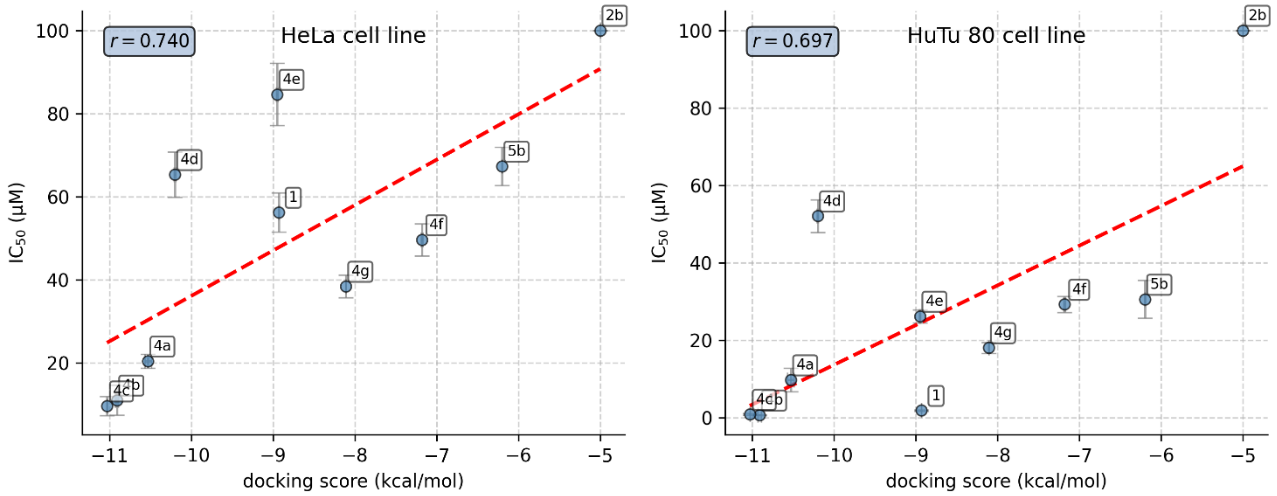

Figure S12 Covalent docking score *vs* IC_{50} plot for the Cys31 residue of PKM2 (PDB code 3GR4) in HeLa and HuTu-80 cell lines

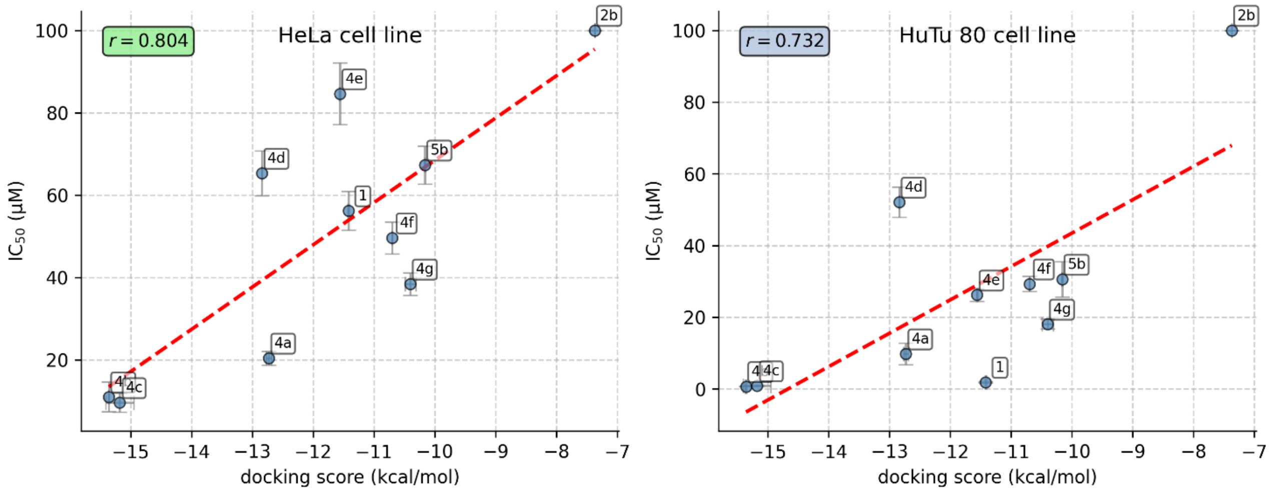

Figure S13 Covalent docking score *vs* IC_{50} plot for the Cys424 residue of PKM2 (PDB code 3GR4) in HeLa and HuTu-80 cell lines

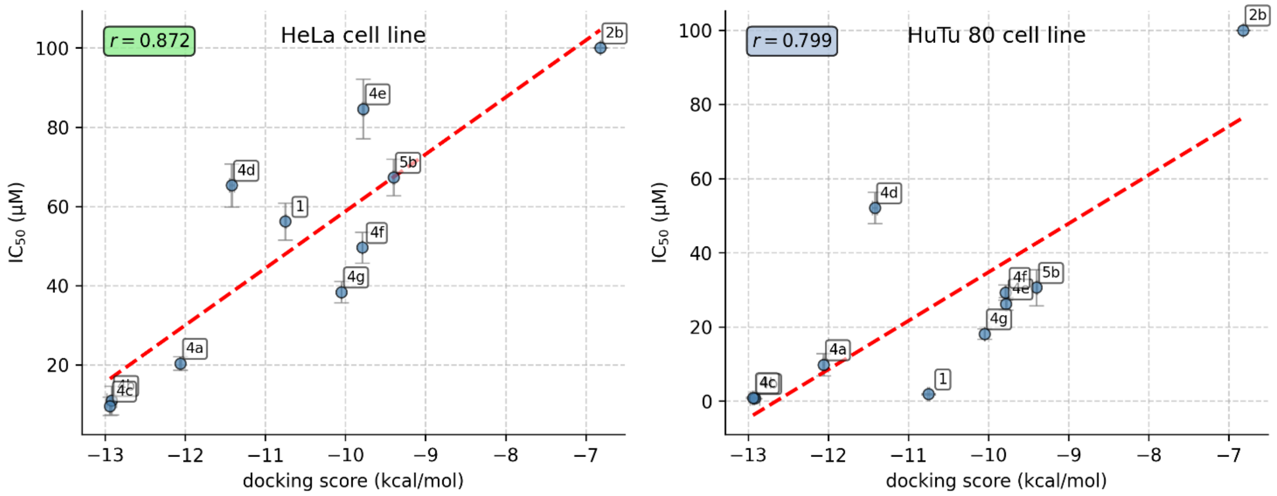

Figure S14 Covalent docking score *vs* IC_{50} plot for the Cys152 residue of GAPDH (PDB code 6M61) with NAD^+ molecule removed in HeLa and HuTu-80 cell lines

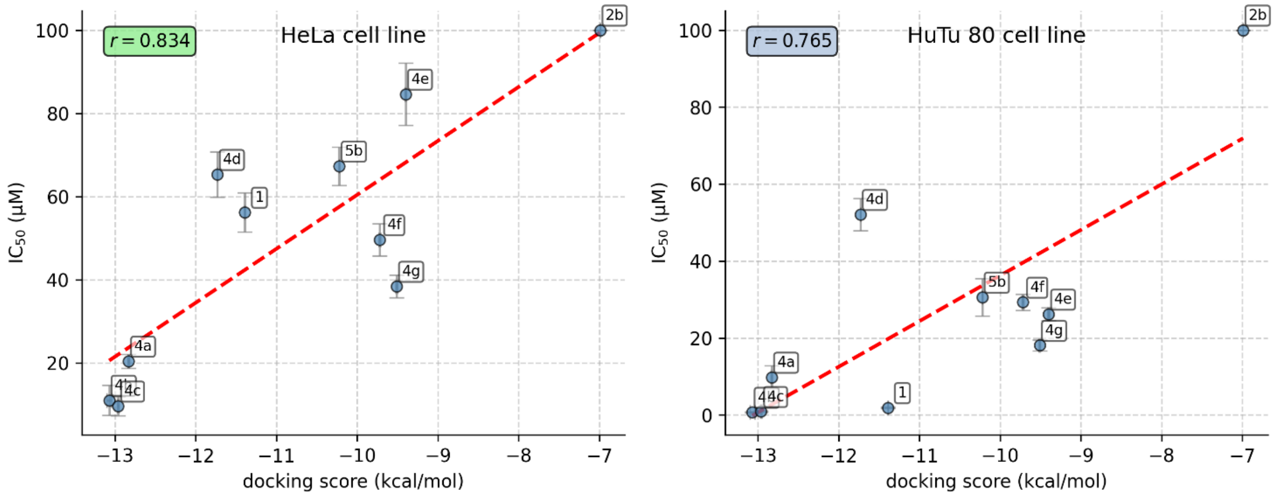

Figure S15 Covalent docking score versus IC_{50} plot for the Cys152 residue of GAPDH (PDB code 6M61) with NAD^+ molecule retained in HeLa and HuTu-80 cell lines

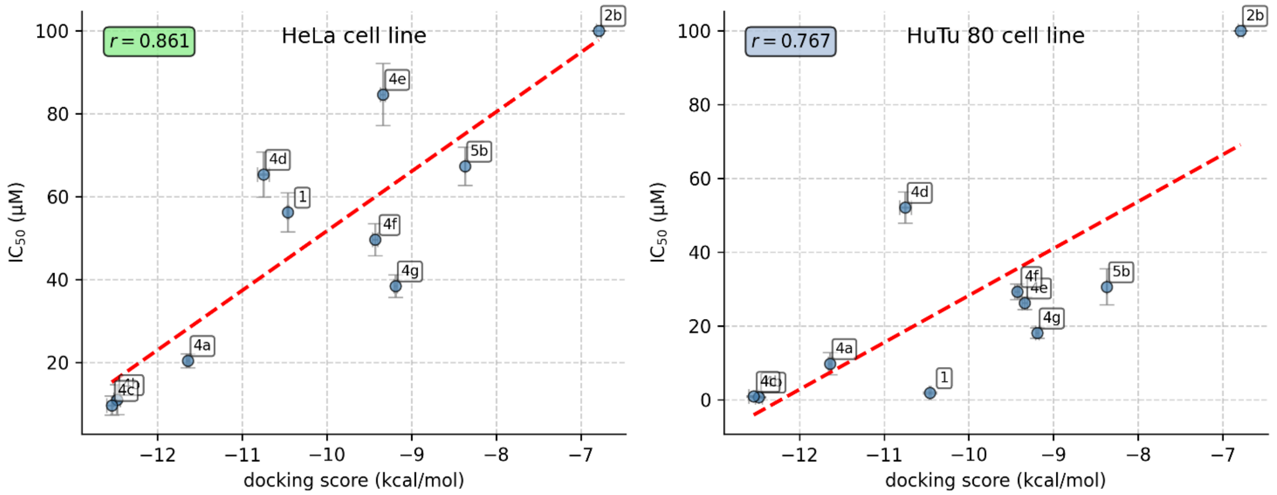

Figure S 16 Covalent docking score *vs* IC_{50} plot for the Cys32 residue of GSTO1 (PDB code 4YQU) in HeLa and HuTu-80 cell lines

Table S3 Correlation between docking scores and cytotoxicity for the tested compounds[a]

target	residue	cell line	Pearson *r*	*p* value	Bonferroni-adjusted *p*	Significant
GAPDH (NAD^+)	Cys152	HUTU	0.765	0.0099	0.1586	No
GAPDH (NAD^+)	Cys152	M-HELA	0.834	0.0027	0.0437	Yes
GAPDH	Cys152	HUTU	0.799	0.0056	0.0889	No
GAPDH	Cys152	M-HELA	0.872	0.0010	0.0160	Yes
GSTO1	Cys32	HUTU	0.766	0.0098	0.1566	No
GSTO1	Cys32	M-HELA	0.862	0.0013	0.0213	Yes
PKM2	Cys317	HUTU	−0.007	0.9855	1.0000	No
PKM2	Cys317	M-HELA	0.179	0.6202	1.0000	No
PKM2	Cys31	HUTU	0.697	0.0250	0.4005	No
PKM2	Cys31	M-HELA	0.740	0.0144	0.2308	No

PKM2	Cys326	HUTU	0.054	0.8827	1.0000	No
PKM2	Cys326	M-HELA	0.205	0.5707	1.0000	No
PKM2	Cys424	HUTU	0.732	0.0160	0.2566	No
PKM2	Cys424	M-HELA	0.804	0.0051	0.0814	No
PKM2	Cys474	HUTU	0.292	0.4131	1.0000	No
PKM2	Cys474	M-HELA	0.462	0.1789	1.0000	No

n = 10 for all correlations; statistical significance was assessed at α = 0.05; p values were adjusted for multiple comparisons using the Bonferroni correction

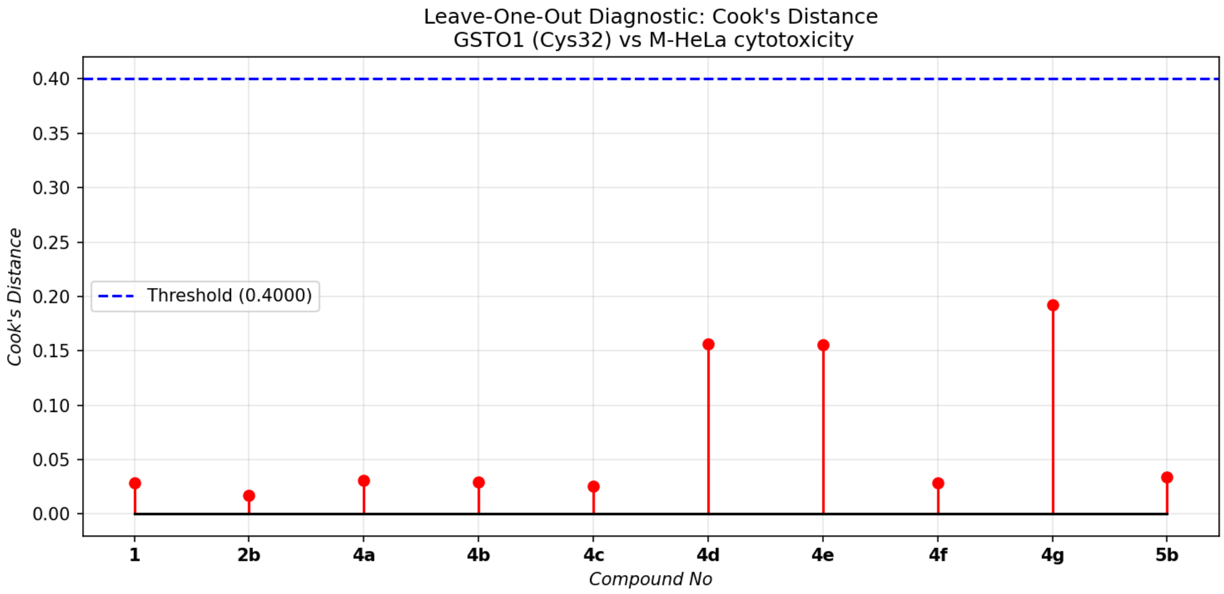

Figure S 17 Leave-one-out sensitivity analysis of the GAPDH docking score *vs* M-HeLa cytotoxicity correlation. Cook's Distance values were calculated after sequential omission of each compound. The dashed horizontal line indicates the critical threshold of 4/N (N = 10)

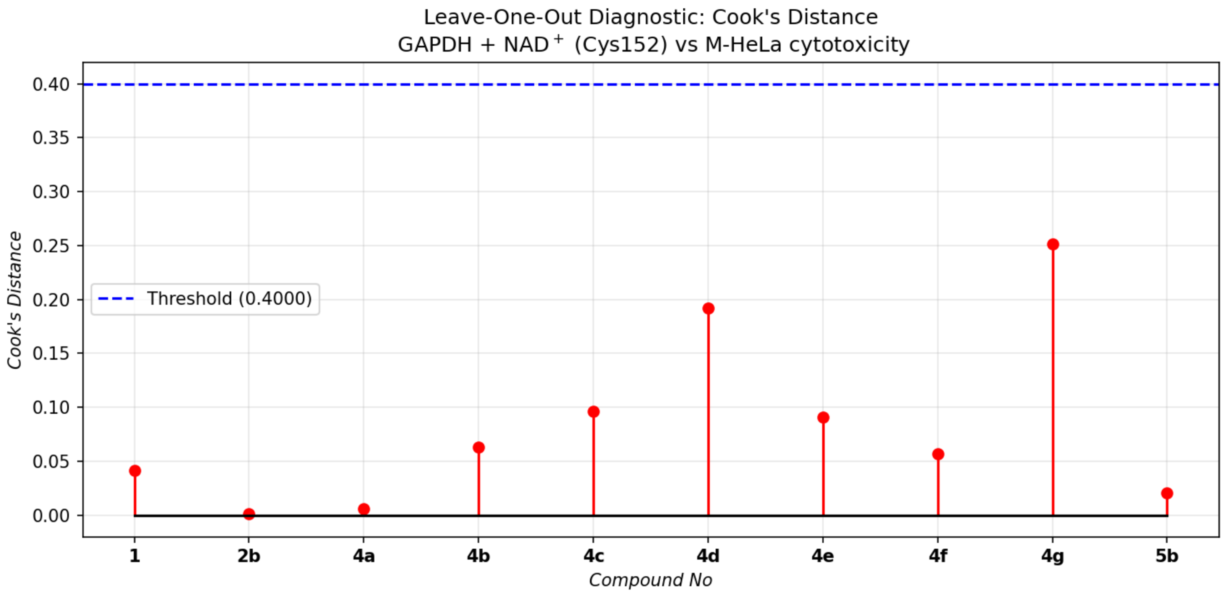

Figure S 18 Leave-one-out sensitivity analysis of the GAPDH (NAD$^+$) docking score *vs* M-HeLa cytotoxicity correlation. Cook's Distance values were calculated after sequential omission of each compound. The dashed horizontal line indicates the critical threshold of 4/N (N = 10)

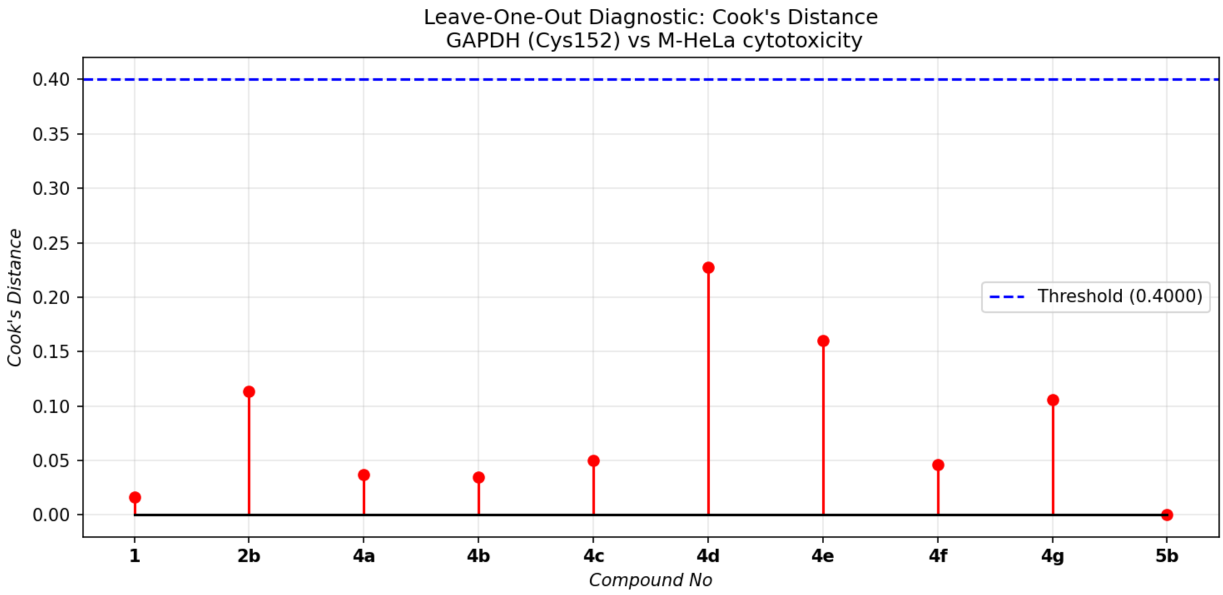

Figure S 19 Leave-one-out sensitivity analysis of the GSTO1 docking score *vs* M-HeLa cytotoxicity correlation. Cook's Distance values were calculated after sequential omission of each compound. The dashed horizontal line indicates the critical threshold of 4/N (N = 10)

Potential hits prediction

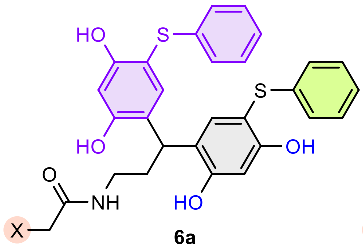

6a

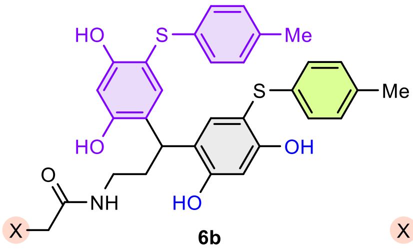

6b

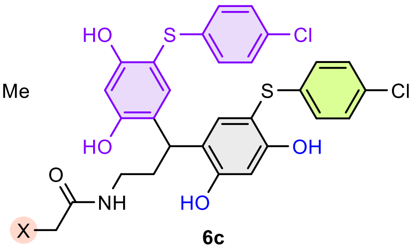

6c

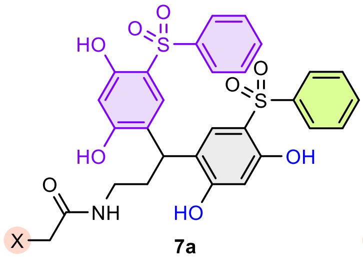

7a

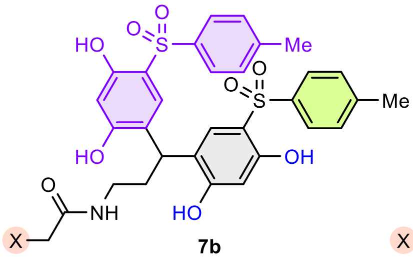

7b

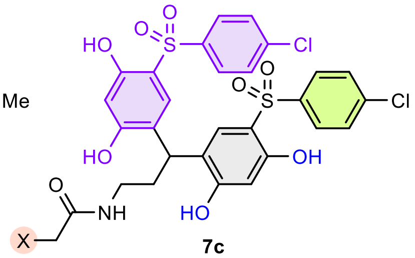

7c

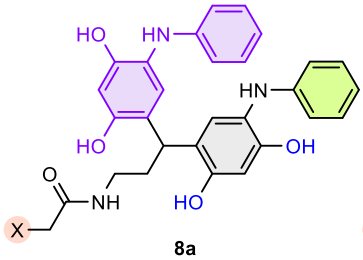

8a

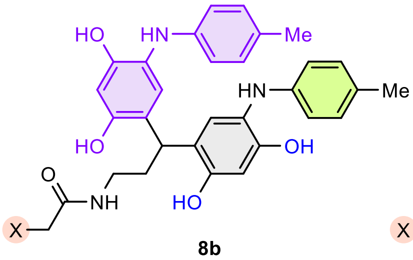

8b

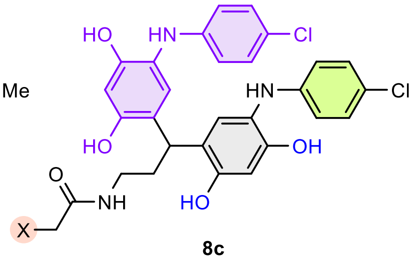

8c

Figure S20 Type **I** compounds used in covalent molecular docking studies

Table S4 Covalent docking scores of type **I** compounds docked to glyceraldehyde 3-phosphate dehydrogenase (GAPDH, PDB code 6M61) and glutathione S-transferase omega 1 (GSTO1, PDB code 4YQU)[a]

**Cmpd**	**GAPDH (CYS152)**			**GSTO1 (CYS32)**		
	docking score (mean)	***RMSD (mean)***	***RMSD (max)***	***docking score (mean)***	***RMSD (mean)***	***RMSD (max)***
**6a**	-14.72	0.068	0.093	-14.25	0.966	1.196
**6b**	-14.80	0.788	1.129	-15.13	0.536	0.739
**6c**	-16.16	0.311	0.364	-14.88	0.972	1.138
**7a**	-16.80	0.171	0.228	-16.14	0.041	0.055
**7b**	-17.04	0.481	0.687	-16.13	0.999	1.146
**7c**	-17.08	0.945	1.145	-16.19	0.761	0.948
**8a**	-13.70	0.124	0.152	-14.48	0.626	0.791
**8b**	-14.63	0.475	0.691	-15.18	1.159	1.471
**8c**	-14.33	0.178	0.221	-14.79	1.118	1.301
**4e**[b]	-9.78	0.097	0.129	-9.34	0.618	0.917
**1**[b,c]	-10.75	0.089	0.121	-10.46	0.054	0.069
**4b''**[b,c]	-12.92	0.503	0.744	-12.48	0.611	0.834
**4c''**[b,c]	-12.94	0.512	0.747	-12.54	0.772	0.933

[a] all docking runs were performed in triplicate; [b] provided for comparison; [c] hit compounds

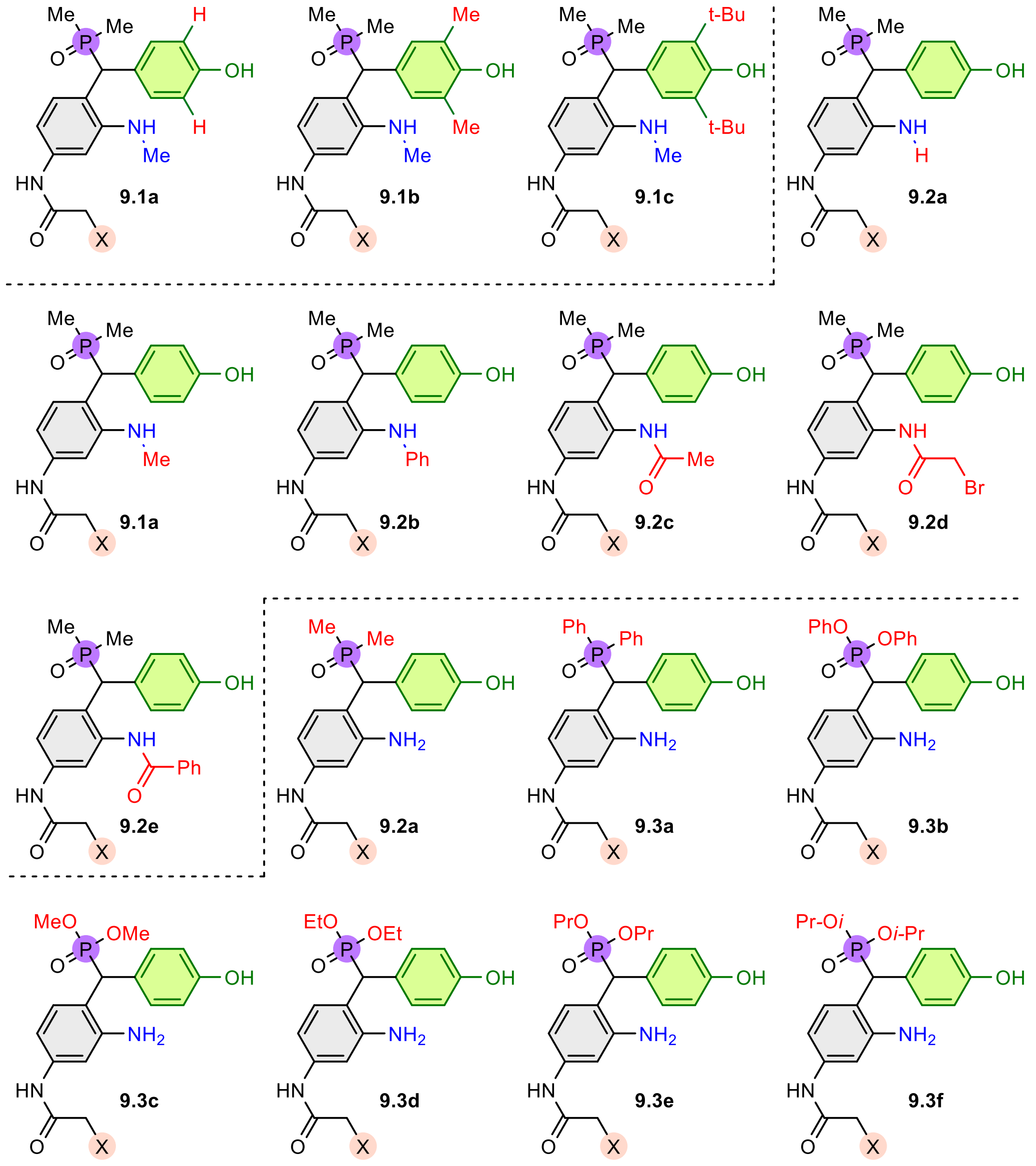

Figure S21 Type **II** compounds used in covalent molecular docking studies. Both enantiomers were modelled; the variable moiety in each series is highlighted in red

Table S5 Covalent docking scores of type **II** compounds **9.1**-**9.3** docked to glyceraldehyde 3-phosphate dehydrogenase (GAPDH, PDB code 6M61) and glutathione S-transferase omega 1 (GSTO1, PDB code 4YQU)[a]

Cmpd	GAPDH (CYS152)			GSTO1 (CYS32)		
	docking score (mean)	*RMSD (mean)*	*RMSD (max)*	*docking score (mean)*	*RMSD (mean)*	*RMSD (max)*
**9.1a-*R***	-8.60	0.914	1.339	-8.02	0.016	0.02
**9.1a-*S***	-8.26	0.037	0.052	-8.46	0.06	0.078
**9.1b-*R***	-8.44	0.783	1.121	-7.99	0.056	0.075
**9.1b-*S***	-8.80	0.031	0.041	-7.92	1.138	1.649
**9.1c-*R***	-10.55	0.702	0.71	-10.30	0.538	0.725
**9.1c-*S***	-10.36	0.502	0.711	-10.23	0.037	0.051
**9.2a-*R***	-8.18	0.041	0.052	-7.58	0.593	0.878
**9.2a-*S***	-8.26	0.061	0.069	-7.63	0.316	0.447
**9.2b-*R***	-11.45	0.043	0.059	-9.93	0.06	0.077
**9.2b-*S***	-11.11	0.033	0.044	-10.11	0.972	1.191
**9.2c-*R***	-9.87	0.061	0.074	-8.91	0.032	0.046
**9.2c-*S***	-9.28	0.045	0.058	-8.24	0.037	0.053
**9.2d-*R***	-10.33	0.019	0.021	-9.29	0.034	0.05

Cmpd	GAPDH (CYS152)			GSTO1 (CYS32)		
	docking score (mean)	*RMSD (mean)*	*RMSD (max)*	*docking score (mean)*	*RMSD (mean)*	*RMSD (max)*
**9.2d-*S***	-9.71	0.587	0.853	-9.72	0.142	0.206
**9.2e-*R***	-10.84	0.069	0.093	-10.91	0.067	0.091
**9.2e-*S***	-10.35	0.197	0.273	-9.76	0.817	1.192
**9.3a-*R***	-12.11	0.052	0.074	-11.38	0.549	0.782
**9.3a-*S***	-10.96	0.035	0.046	-11.28	0.461	0.615
**9.3b-*R***	-13.33	0.537	0.751	-11.69	0.034	0.039
**9.3b-*S***	-12.27	0.53	0.768	-12.33	0.515	0.750
**9.3c-*R***	-9.28	0.028	0.034	-8.59	0.045	0.048
**9.3c-*S***	-9.30	0.072	0.088	-8.27	0.045	0.049
**9.3d-*R***	-10.91	0.397	0.591	-9.78	0.732	0.967
**9.3d-*S***	-10.42	0.176	0.223	-9.96	0.147	0.182
**9.3e-*R***	-12.16	0.066	0.082	-10.16	0.346	0.453
**9.3e-*S***	-11.19	0.69	0.871	-11.06	0.726	0.988
**9.3f-*R***	-11.63	0.125	0.179	-10.63	0.275	0.403
**9.3f-*S***	-11.05	0.072	0.09	-9.56	0.478	0.681

[a] *all docking runs were performed in triplicate*

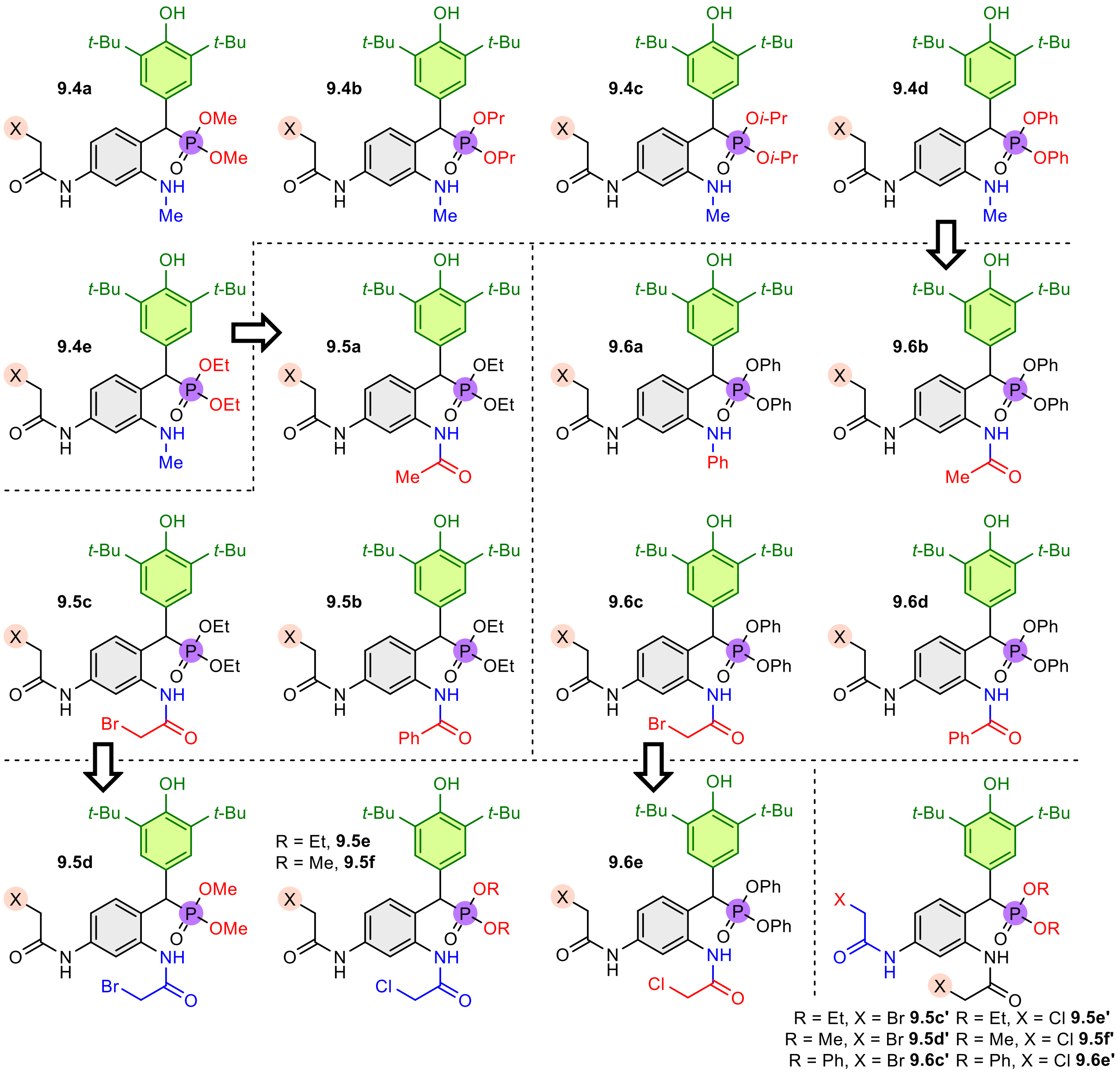

Figure S22 Expansion of the type **II** compound series used in covalent molecular docking studies. Both enantiomers were modelled; the variable moiety in each series is highlighted in red

Table S6 Covalent docking scores of type **II** compounds **9.4**-**9.6** docked to glyceraldehyde 3-phosphate dehydrogenase (GAPDH, PDB code 6M61) and glutathione S-transferase omega 1 (GSTO1, PDB code 4YQU)[a]

Cmpd	GAPDH (CYS152)			GSTO1 (CYS32)		
	docking score (mean)	*RMSD (mean)*	*RMSD (max)*	*docking score (mean)*	*RMSD (mean)*	*RMSD (max)*
**9.4a-*R***	-12.21	0.046	0.064	-10.56	2.114	2.832[b]
**9.4a-*S***	-10.93	0.176	0.250	-10.45	0.791	1.036
**9.4b-*R***	-14.34	0.786	1.131	-12.54	0.747	1.057
**9.4b-*S***	-13.28	0.456	0.663	-12.50	0.750	0.796
**9.4c-*R***	-14.01	0.412	0.610	-12.46	0.112	0.125
**9.4c-*S***	-12.58	1.100	1.275	-12.11	1.148	1.255
**9.4d-*R***	-15.74	0.917	1.147	-14.37	2.092	2.626[b]
**9.4d-*S***	-14.29	1.925	2.558[b]	-13.91	0.657	0.793
**9.4e-*R***	-12.97	0.077	0.085	-11.73	0.068	0.089

Cmpd	GAPDH (CYS152)			GSTO1 (CYS32)		
	docking score (mean)	*RMSD (mean)*	*RMSD (max)*	*docking score (mean)*	*RMSD (mean)*	*RMSD (max)*
**9.4e-*S***	-12.73	0.464	0.668	-11.85	2.174	2.873[b]
**9.5a-*R***	-12.92	2.053	2.739[b]	-12.21	0.711	0.893
**9.5a-*S***	-13.56	0.761	0.939	-12.66	0.184	0.228
**9.5b-*R***	-15.37	0.315	0.435	-14.12	0.480	0.646
**9.5b-*S***	-14.34	0.022	0.026	-14.18	0.762	0.945
**9.5c-*R***	-14.92	0.646	0.914	-12.94	0.459	0.653
**9.5c-*S***	-13.86	0.757	0.941	-13.42	2.030	2.686[b]
**9.5d-*R***	-13.03	0.927	1.018	-12.10	0.726	0.772
**9.5d-*S***	-13.67	0.757	0.929	-11.76	0.468	0.660
**9.5e-*R***	-13.91	0.383	0.528	-13.06	1.275	1.884
**9.5e-*S***	-13.67	0.366	0.499	-13.17	0.761	0.907
**9.5f-*R***	-13.20	0.119	0.130	-12.01	0.421	0.615
**9.5f-*S***	-12.27	0.268	0.375	-11.63	0.972	1.170
**9.6a-*R***	-17.95	2.010	2.521[b]	-16.62	0.896	1.100
**9.6a-*S***	-16.40	0.058	0.069	-15.94	0.443	0.650
**9.6b-*R***	-15.70	0.682	0.848	-14.76	0.928	1.086
**9.6b-*S***	-15.41	0.709	0.844	-14.15	1.714	2.505[b]
**9.6c-*R***	-16.16	1.752	2.535[b]	-15.60	0.617	0.899
**9.6c-*S***	-16.40	0.802	1.093	-15.50	0.058	0.065
**9.6d-*R***	-18.16	0.790	0.884	-16.31	1.259	1.460
**9.6d-*S***	-17.64	0.591	0.858	-16.14	0.981	1.067
**9.6e-*R***	-15.94	1.848	2.482	-15.12	0.913	0.938
**9.6e-*S***	-16.06	0.802	0.899	-14.59	0.500	0.730
**9.5c'-*R***	-14.07	0.739	0.917	-16.27	0.464	0.658
**9.5c'-*S***	-14.76	0.787	0.978	-14.85	0.075	0.084
**9.5d'-*R***	-13.32	0.112	0.160	-15.24	0.755	0.938
**9.5d'-*S***	-13.50	0.870	0.984	-13.89	0.268	0.397
**9.5e'-*R***	-15.15	1.800	2.668[b]	-15.69	0.653	0.665
**9.5e'-*S***	-14.81	0.544	0.705	-14.73	0.119	0.136
**9.5f'-*R***	-13.20	0.460	0.664	-14.84	0.950	1.188
**9.5f'-*S***	-13.27	0.689	0.735	-13.17	0.732	1.074
**9.6c'-*R***	-17.17	0.703	0.955	-17.64	1.033	1.131
**9.6c'-*S***	-17.59	0.438	0.614	-18.41	0.728	0.897
**9.6e'-*R***	-16.90	2.011	2.597[b]	-17.96	0.511	0.680
**9.6e'-*S***	-16.77	0.493	0.635	-17.93	0.776	0.860

[a] all docking runs were performed in triplicate; [b] the high RMSD value results from rotation of the tert-butyl group

Full cytotoxicity data for compounds 9.5c-f and 9.6c,e

Table S7 Cytotoxicity (IC_{50}, μM) of synthesized compounds **9.5c-f** and **9.6c,e** against cancer and normal cell lines[a]

Compound	IC50 (μM)										
	Cancer cell lines									Normal cell lines	
	**M-HeLa**	**MCF-7**	HuTu-80	**Du-145**	**Hep G2**	**A549**	**PC3**	**T98G**	**PANC-1**	**WI38**	**Chang liver**
**9.5f**	16.8±1.3	70.4±6.4	-	-	-	-	-	-	-	-	39.8±2.8
**9.6e**	31.8±2.8	>100	-	-	-	-	-	-	-	-	>100
**9.5d**	0.9	1.4±0.1	4.5±0.4	3.4±0.3	2.7±0.2	5.6±0.4	2.3±0.1	3.1±0.2	1.8±0.1	1.7±0.1	5.2±0.4
**9.5c**	1.9±0.1	1.2±0.1	5.8±0.5	2.4±0.2	3.7±0.3	5.1±0.4	2.6±0.2	4.5±0.4	1.8±0.1	2.3±0.1	4.5±0.4
**9.6c**	2.3±0.2	2.0±0.2	4.3±0.4	2.7±0.2	5.1±0.4	7.8±0.7	3.6±0.3	3.8±0.3	2.1±0.1	2.7±0.2	2.8±0.3
**Sorafenib**	25.0±1.9	14.3±1.3	5.0±0.5	14.9±0.7	14.7±9.0	22.3±1.8	11.3±0.9	12.9±1.4	35.0±2.8	17.1±1.4	35.0±1.7
**Doxorubicin**	2.1±0.1	2.5±0.2	3.0	1.3±0.1	2.9±0.1	0.9	1.4±0.1	5.7±0.4	2.4±0.2	3.5±0.1	2.1±0.1

[a] *Three independent experiments were carried out; "–" means not tested; all compounds were tested as racemic mixtures*

References

(1) Meng, E. C.; Goddard, T. D.; Pettersen, E. F.; Couch, G. S.; Pearson, Z. J.; Morris, J. H.; Ferrin, T. E. <scp>UCSF ChimeraX</scp> : Tools for structure building and analysis. *Protein Sci.* **2023**, *32* DOI: 10.1002/pro.4792.

(2) Meeko 0.6.1 https://github.com/forlilab/meeko.

(3) O'Boyle, N. M.; Banck, M.; James, C. A.; Morley, C.; Vandermeersch, T.; Hutchison, G. R. Open Babel: An open chemical toolbox. *J. Cheminform.* **2011**, *3*, 33 DOI: 10.1186/1758-2946-3-33.

(4) Bannwarth, C.; Ehlert, S.; Grimme, S. GFN2-xTB—An Accurate and Broadly Parametrized Self-Consistent Tight-Binding Quantum Chemical Method with Multipole Electrostatics and Density-Dependent Dispersion Contributions. *J. Chem. Theory Comput.* **2019**, *15*, 1652–1671 DOI: 10.1021/acs.jctc.8b01176.

(5) Neese, F. Software update: The <scp>ORCA</scp> program system—Version 5.0. *WIREs Comput. Mol. Sci.* **2022**, *12* DOI: 10.1002/wcms.1606.

(6) Neese, F.; Wennmohs, F.; Becker, U.; Riplinger, C. The ORCA quantum chemistry program package. *J. Chem. Phys.* **2020**, *152*, 224108 DOI: 10.1063/5.0004608.

(7) Santos-Martins, D.; Solis-Vasquez, L.; Tillack, A. F.; Sanner, M. F.; Koch, A.; Forli, S. Accelerating A <scp>uto</scp> D <scp>ock</scp> 4 with GPUs and Gradient-Based Local Search. *J. Chem. Theory Comput.* **2021**, *17*, 1060–1073 DOI: 10.1021/acs.jctc.0c01006.

(8) AutoGrid https://github.com/ccsb-scripps/autogrid (accessed Aug 6, 2025).

(9) Adasme, M. F.; Linnemann, K. L.; Bolz, S. N.; Kaiser, F.; Salentin, S.; Haupt, V. J.; Schroeder, M. PLIP 2021: expanding the scope of the protein–ligand interaction profiler to DNA and RNA. *Nucleic Acids Res.* **2021**, *49*, W530–W534 DOI: 10.1093/nar/gkab294.

(10) Ramkumar, K.; Samanta, S.; Kyani, A.; Yang, S.; Tamura, S.; Ziemke, E.; Stuckey, J. A.; Li, S.; Chinnaswamy, K.; Otake, H.; et al. Mechanistic evaluation and transcriptional signature of a glutathione S-transferase omega 1 inhibitor. *Nat. Commun.* **2016**, *7*, 13084 DOI: 10.1038/ncomms13084.

(11) Yan, Y.; Zang, X.; Jamieson, C. S.; Lin, H.-C.; Houk, K. N.; Zhou, J.; Tang, Y. Biosynthesis of the fungal glyceraldehyde-3-phosphate dehydrogenase inhibitor heptelidic acid and mechanism of self-resistance. *Chem. Sci.* **2020**, *11*, 9554–9562 DOI: 10.1039/D0SC03805A.

(12) Feeney, J.; Newman, G. A.; Pauwels, P. J. S. A study of pyrazolin-5-one tautomerism. Part IV. Natural abundance carbon-13 nuclear magnetic resonance spectra. *J. Chem. Soc. C Org.* **1970**, 1842 DOI: 10.1039/j39700001842.

(13) Bell, S. J.; Mazzola, E. P.; Dinovi, M. J.; Reynolds, W. F.; Nielsen, K. W. 13 C NMR investigation of tautomeric and acid-base equilibria of pyrazolone T and three analogs. *J. Heterocycl. Chem.* **1991**, *28*, 641–645 DOI: 10.1002/jhet.5570280318.

(14) Tanaka, M.; Sugimura, N.; Fujisawa, A.; Yamamoto, Y. Stabilizers of edaravone aqueous solution and their action mechanisms. 1. Sodium bisulfite. *J. Clin. Biochem. Nutr.* **2017**, *61*, 159–163 DOI: 10.3164/jcbn.17-61.
